# Kinetic and Thermodynamic Modulation of Dynamic Imine Libraries Driven by the Hexameric Resorcinarene Capsule

**DOI:** 10.1021/jacs.0c04705

**Published:** 2020-08-07

**Authors:** Stefania Gambaro, Carmen Talotta, Paolo Della Sala, Annunziata Soriente, Margherita De Rosa, Carmine Gaeta, Placido Neri

**Affiliations:** Laboratory of Supramolecular Chemistry, Dipartimento di Chimica e Biologia “A. Zambelli”, Università di Salerno, Via Giovanni Paolo II, I-84084 Fisciano, Salerno, Italy

## Abstract

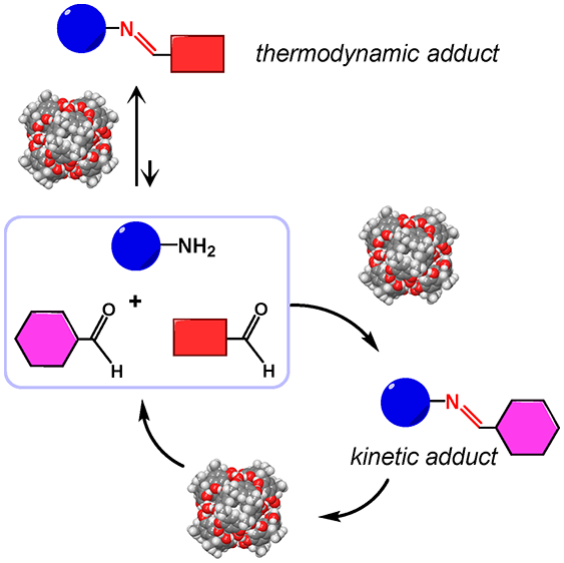

The composition of dynamic covalent imine libraries (DCL) adapts to the presence of the hexameric resorcinarene capsule. In the presence of the self-assembled capsule, a kinetic and thermodynamic modulation of the imine constituents of the DCLs was observed, which was induced by an unusual predatory action of the capsule on specific imine constituents. More complex 2 × 2 DCLs also adapt to the presence of the hexameric capsule, showing a thermodynamic and kinetic modulation of the constituents induced by the predatory action of the capsule. By cross-referencing experimental data, a good selectivity (up to 66%) for one constituent can be induced in a 2 × 2 DCL.

## Introduction

Nature is a continual source of inspiration for those scientists interested in mimicking the strict level of selectivity and efficiency that are the basis of living systems.^1^ Biomimicry^2,3^ starts from the inspiration of natural processes which include the modus operandi of natural enzymes, one of the most amazing phenomena in biological chemistry.^3^ Enzymes are able to work selectively in the presence of complex mixture of substrates, leading to the selective formation of specific products at once. On this basis, one of the aims of enzyme mimicry^3^ is the synthesis of artificial systems able to work selectively in the presence of a complex mixture of reagents.

**Figure 1:**
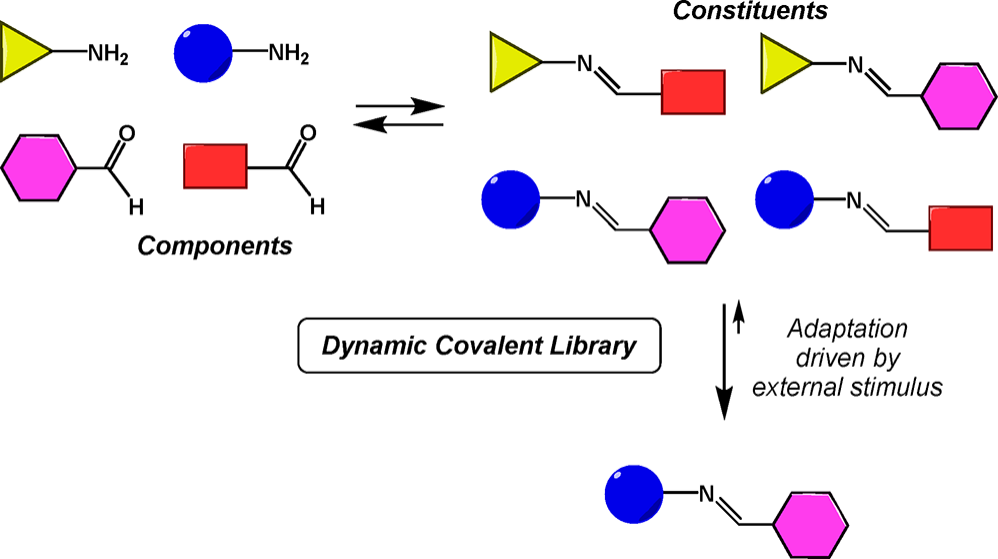
Adaptation of a dynamic covalent library of imines to an external stimulus.

In the past decades, dynamic covalent chemistry (DCC)^4−6^ has aroused a particular interest. In DCC, simple building blocks are held together by reversible covalent bonds to form a library of products that, under thermodynamic equilibrium conditions, are continuously interconverting.^6^ Under these conditions, the library is usually able to respond to an external stimulus (Figure 1) by changing its equilibrium composition according to Le Châtelier’s principle. Examples were reported in the literature in which dynamic covalent libraries (DCL) undergo reorganization as a response to a physical stimulus such as temperature,^6a^ crystallization,^6b,6c^ distillation,^7^ or phase separation.^8^ Interesting examples of supramolecular modulation of DCLs have been also reported in the literature.^9a^ Sanders and Pantoş pointed out that a dynamic library of naphthalenediimide-based macrocycles responded to the presence of different complementary naphthalene guests.^9b^ Another example of supramolecular modulation of a DCL was reported by Sanders and co-workers in which a dynamic library of hydrazone-based pseudopeptides changed in the product distribution after addition of acetylcholine.^9c^ In the presence of the ammonium guest, the equilibrium shifted toward the cyclic trimer, which was able to selectively bind acetylcholine.^9c^ Analogously, a dynamic library of cyclic pseudopeptide receptors changed its equilibrium distribution in the presence of a Li^+^ guest, which was able to convert a complex mixture of about 10 macrocycles into one that contains 98% of the Li^+^ receptor.^10^

Among the major subjects explored in DCC, surely the imines have attracted particular attention.^11^ Formation of an imine bond is a dynamic process where a carbonyl compound reacts in a reversible manner with an amino group with the loss of water (Figure 1). Usually, upon response to an external stimulus, an imine-based dynamic library reorganizes the composition of its constituents and drives it toward the preferential formation of only selected members (Figure 1).

In particular, the reversible formation of imine bonds is affected by external factors such as temperature, pH, and concentration but also by internal factors such as steric and electronic features of the substrates. Dynamic imines have been used in different applications, including the synthesis of complex molecular architectures, such as cages,^12^ and in self-sorting systems.^13^

**Figure 2:**
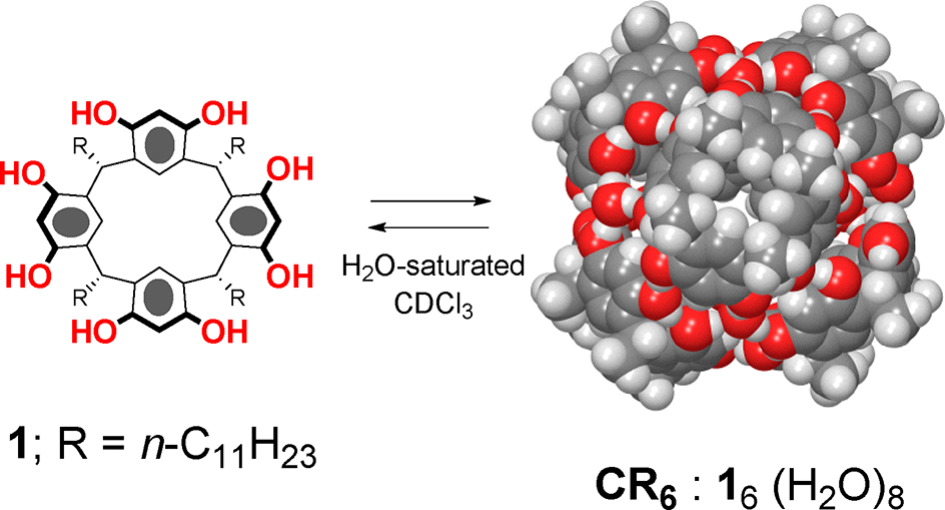
Self-assembly of resorcinarene **1** in water-saturated CDCl_3_ forming the hexameric resorcinarene capsule **CR**_**6**_.

Recently, in biomimicry, much attention has been devoted to catalytic processes in a nanoconfined space using self-assembled capsules.^14^ The confined space inside the self-assembled containers looks like an enzyme pocket and provides interesting catalytic features. Among the self-assembled architectures, the hexameric resorcinarene capsule **CR**_**6**_ (Figure 2) has become increasingly important in catalysis.^15^ The formation of a hexameric resorcin[4]arene capsule **CR**_**6**_ (Figure 2) in the solid state was originally reported by Atwood,^16^ whereas evidence for its formation in water-saturated chloroform or wet benzene solution was provided by Cohen^17^ and co-workers by diffusion NMR experiments. The capsule is obtained by self-assembly of six resorcinarene **1** and eight water molecules, sealed by 60 H-bonding interactions. The container **CR**_**6**_ shows some features that make it a useful tool in biomimetic catalysis:^3^ (a) the internal π···electron-rich cavity of 1375 Å^3^ is able to recognize neutral and cationic species and to stabilize transition states, due to secondary interactions; (b) the capsule **CR**_**6**_ behaves as a mild Brønsted acid with a p*K*_a_ value of about 5.5–6.0;^18a^ (c) four bridging water molecules show a H-bond-donating free valence, which is catalytically relevant.^18b,19^ In addition, previously reported data^20^ show that the **CR**_**6**_ capsule is able to exert a substrate selectivity, whereas stereo- and regioselectivity toward the products are also generally observed.

Tiefenbacher and co-workers reported the first example of catalysis of formation of an iminium group inside **CR**_**6**_,^21^ exploiting its mild acidity and its ability to stabilize cationic intermediates and transition states. Successively, our group reported experimental and computational evidence of the formation of an iminium specie inside **CR**_**6**_.^22^

These considerations prompted us to investigate the behavior of dynamic imine libraries in the presence of **CR**_**6**_. As stated by Lehn,^7a^ “changes in expression of the different constituents as a factor of external parameters represent an adaptation of the system to environmental conditions”. On this basis, we wonder if the composition of dynamic imine libraries adapts to the presence of **CR**_**6**_, which, in addition to catalytic abilities, usually also shows a substrate and product selectivity.

## Results and Discussion

### Adaptation of the DCL of Imines **A2a** and **A2b** to the Presence of the Hexameric Capsule

**Scheme 1:**
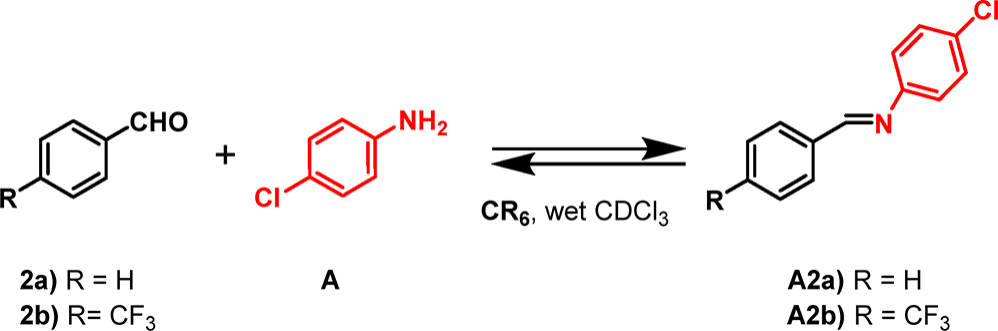
Synthesis of Imines **A2a** and **A2b** in the Presence of Capsule **CR**_**6**_

We start this study by investigating the formation of imines **A2a** and **A2b** in single experiments (Scheme 1) in the presence or in the absence of the hexameric capsule **CR**_**6**_.

**Figure 3:**
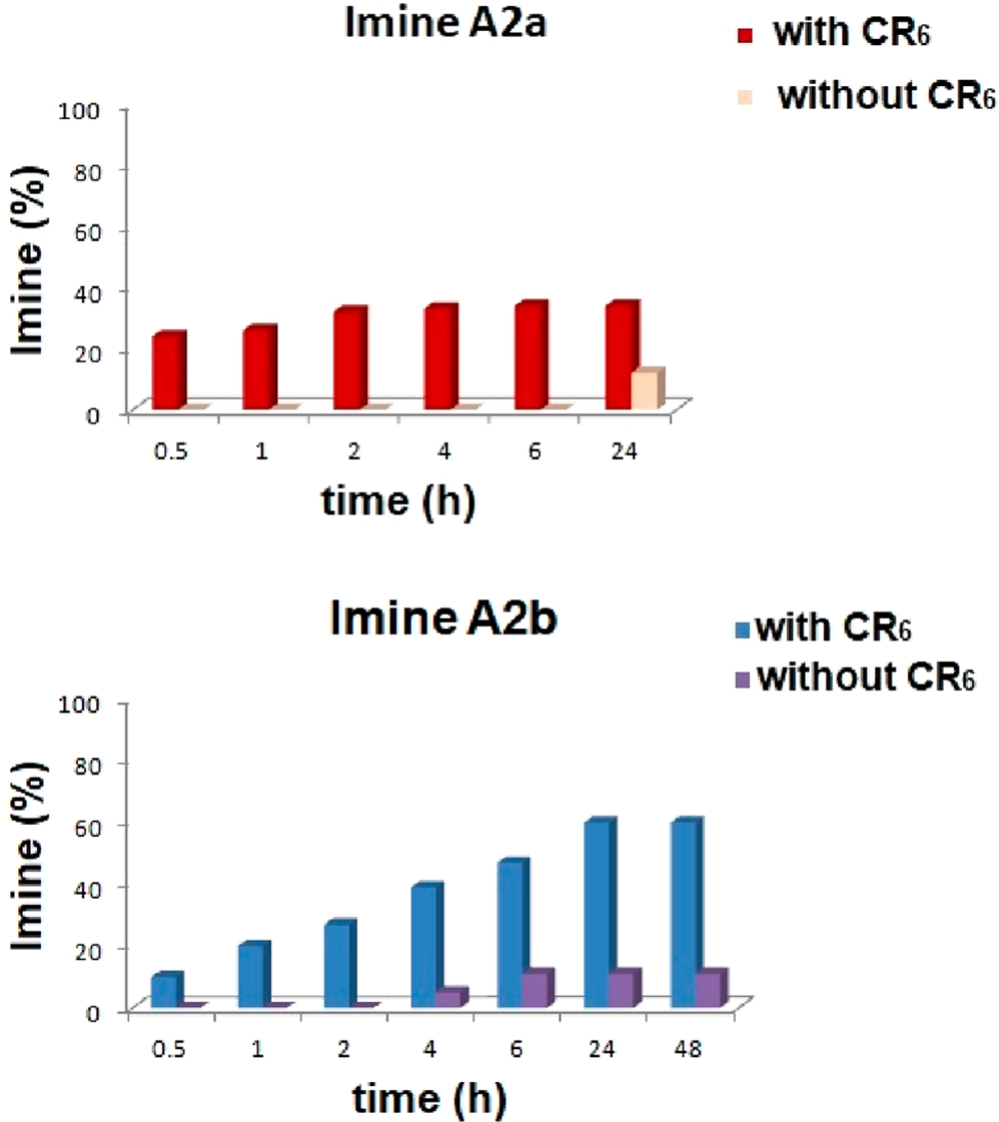
Formation of imines **A2a** (top) and **A2b** (bottom) during the single experiments in the presence or in the absence of capsule **CR**_**6**_ (Scheme 1).

When benzaldehyde **2a** and *p*-chloroaniline **A** were mixed in an equimolar ratio (42.3 mM) in water-saturated CDCl_3_ at 30 °C in the presence of capsule **CR**_**6**_ (1 equiv), the formation of imine **A2a** was detected in the reaction mixture after 30 min (Figure 3). The equilibrium was reached after 2 h, leading to 34% of **A2a** (Figure 3).

When the reaction in Scheme 1 was performed under the same conditions but in the absence of capsule **CR**_**6**_, the formation of **A2a** was slowed and only 12% of it was detected in the reaction mixture after 24 h.

When *p*-trifluoromethylbenzaldehyde **2b** was used with *p*-chloroaniline **A** in the presence of capsule **CR**_**6**_, imine **A2b** reached an equilibrium value of 60% after 24 h (Figure 3). Also, in this case, the formation of **A2b** was slowed in the absence of **CR**_**6**_ (Figure 3).

**Scheme 2:**
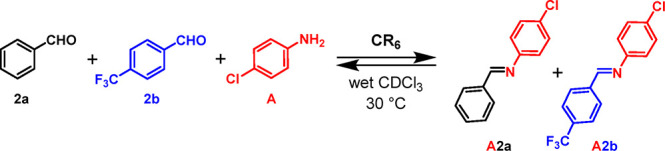
Dynamic Library Formed by the Three Components **2a**, **2b**, and **A** and by the Two Constituents **A2a** and **A2b** in the Presence of **CR**_**6**_

**Figure 4:**
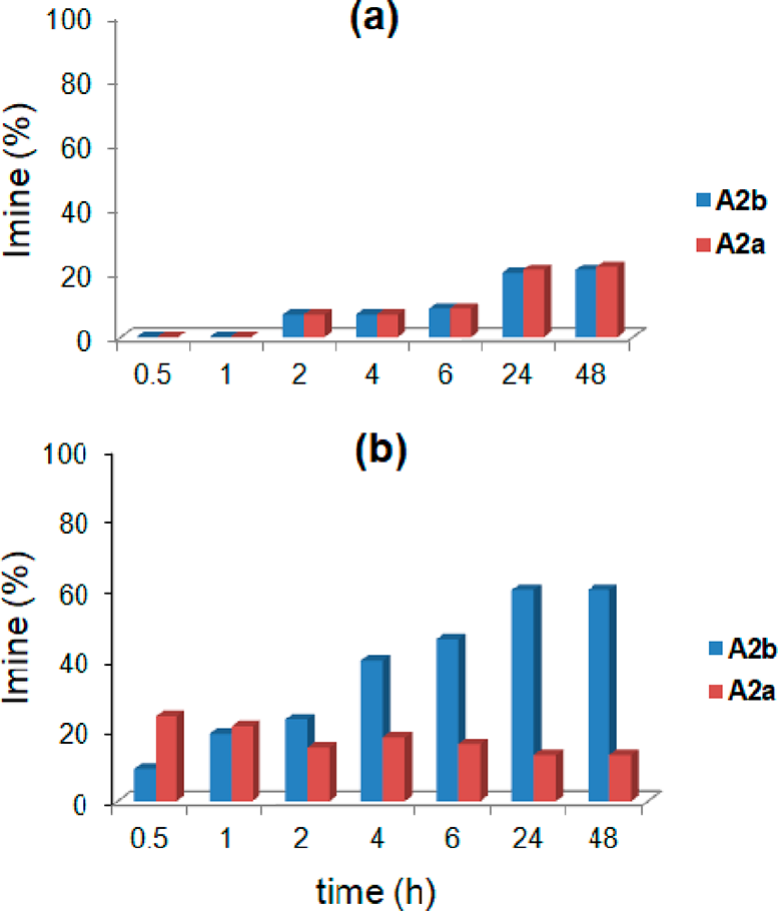
Distribution of imine constituents **A2a** and **A2b** in the DCL in Scheme 2, without (a) and with (b) capsule **CR**_**6**_.

With these results in hand, we then investigated an imine-based DCL of two imine constituents, **A2a** and **A2b** (Scheme 2), formed by the three components of benzaldehyde **2a**, *p*-trifluoromethylbenzaldehyde **2b**, and *p*-chloroaniline **A** (in an equimolar ratio, Scheme 2). Experiments were performed either in the presence or in the absence of capsule **CR**_**6**_ in water-saturated CDCl_3_ using a concentration of 42.3 mM each of **2a**/**2b**/**A/CR**_**6**_. The reactions were conducted at 30 °C. The formation of imine products was monitored as a function of time by quantitative ^1^H NMR (qNMR) spectroscopy using 1,1,2,2-tetracloroethane (TCE) as an internal standard. Aliquots of the reaction mixtures were added to DMSO (Supporting Information) in order to disaggregate **CR**_**6**_, and the imine signals were integrated with respect to the signal of TCE. In the absence of the **CR**_**6**_ capsule, two imine constituents, **A2a** and **A2b**, were formed in equal quantities up to 48 h (Figure 4a) when the conversion was about 20% for each.

Interestingly, the DCL in Scheme 2 adapts to the presence of the capsule **CR**_**6**_ (Figure 4b). In fact, imines **A2a** and **A2b** were formed immediately after being mixed with a conversion of 30 and 10%, respectively.

Imine **A2a**, obtained by benzaldehyde **2a** and *p*-chloroaniline **A**, was formed faster than **A2b**, but after 1 h, **A2a** started to decrease as **A2b** increased. This trend continued up to 24 h, when the **A2a**/**A2b** ratio reached a value of 15/60 and remained constant (48 h). The results in Figure 4 showed that **A2a** was kinetically favored, whereas **A2b** was the thermodynamic product under these conditions.

**Scheme 3:**
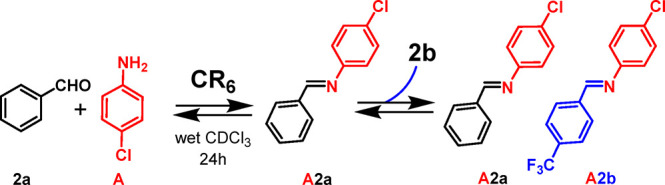


**Figure 5:**
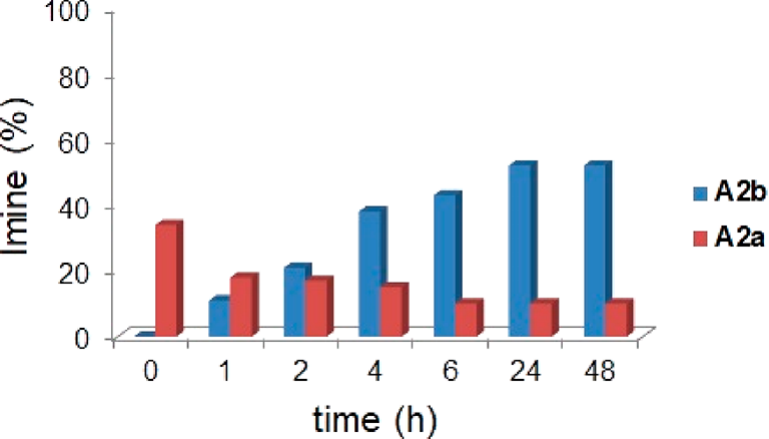
Imine constituents distributions in DCL in Scheme 3.

As known, in an imine-based DCL, the imine constituents exchange their components between them by reversible formation of chemical bonds. Usually, these processes are under thermodynamic control, and in this way, the most stable constituent prevails. Thus, we have envisioned a new experiment, reported in Scheme 3, in which benzaldehyde **2a** and *p*-chloroaniline **A** were reacted in the presence of **CR**_**6**_ in order to form only imine **A2a**. After 24 h, **A2a** was formed in 34% yield (Scheme 3). At this point, the mixture was added to 1 equiv of aldehyde **2b**, and 1 h later, **A2b** started to increase as **A2a** decreased (Scheme 3 and Figure 5). The equilibrium was reached 24 h later (Figure 5), showing a distribution pattern close to that obtained in the experiment in Figure 4b.

The evolution of the imine composition in Figure 4 clearly indicates that the capsule **CR**_**6**_ shows two effects:

(a) **CR**_**6**_ acts as a catalyst by accelerating the formation of imine constituents **A2a** and **A2b**, due to its mild acidity and capability to stabilize cationic intermediates.
(b) **CR**_**6**_ acts as an external stimulus because the DCL composition of **A2a** and **A2b** adapts to its presence. The formation of imine **A2a** is initially favored, whereas **A2b** prevails at longer time. Thus, under these conditions, **A2a** and **A2b** represent the kinetic and the thermodynamic adducts, respectively.

In order to get more insights on the mechanism of this kinetic and thermodynamic modulation of the DCL in Scheme 2, we performed uptake experiments.^23^ In detail, a competition experiment was carried out in which benzaldehyde **2a** and *p*-trifluoromethylbenzaldehyde **2b** were in competition to occupy the inner cavity of **CR**_**6**_. The uptake of **2a**/**2b** inside **CR**_**6**_ was measured by quantitative ^1^H NMR experiments, in which the aldehydes **2a** and **2b** (42.3 mM each one) were mixed in the presence of 1 equiv of **CR**_**6**_ in water-saturated CDCl_3_. The quantity of encapsulated aldehyde was obtained by determining the difference between its initial concentration and the concentration of the free aldehyde in solution. The ^1^H NMR signal of the free aldehyde was integrated with respect to the signal of the internal standard (TCE). After equilibration, a 52% uptake of benzaldehyde **2a** inside **CR**_**6**_ was measured, a value significantly higher than that obtained for the aldehyde **2b** (5%). Thus the hexameric capsule **CR**_**6**_ shows a higher affinity for benzaldehyde **2a** with respect to *p*-CF_3_-benzaldehyde **2b**. Clearly, this result is in accord with the finding that **A2a** is preferentially formed in the early stage of the reaction, where the capsule is filled to a greater extent with benzaldehyde **2a**.

Proof of the encapsulation of benzaldehyde **2a** inside **CR**_**6**_ was obtained by 1D and 2D NMR studies and, in particular, by HSQC experiments (Supporting Information, Figures S48–S54). From these studies, it emerges that the benzaldehyde is encapsulated inside **CR**_**6**_ with slow kinetics with respect to the NMR time scale (600 MHz). Analogously, the encapsulation of the aldehyde **2b** inside **CR**_**6**_ was studied by 1D and 2D NMR experiments (Supporting Information, Figures S55–S59). Analogous studies were reported in the Supporting Information in order to show the encapsulation of *p*-chloroaniline **A** inside **CR**_**6**_ (Supporting Information, Figures S60–S63).

The fate of the two imines **A2a** and **A2b** remains to be understood. In detail, we wonder why **A2b** prevails for a long time whereas **A2a** decreases with respect to its initial percentage.

**Figure 6:**
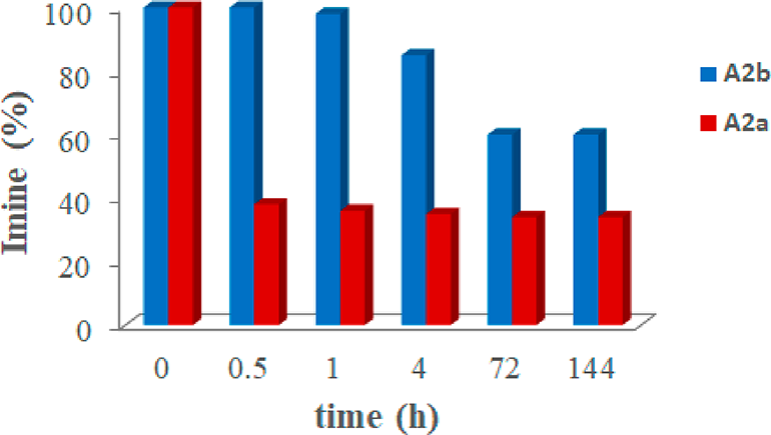
Hydrolysis of imines **A2a** and **A2b** in the presence of capsule **CR**_**6**_.

When **A2a** was dissolved in water-saturated CDCl_3_ solution in the presence of **CR**_**6**_ (1 equiv), after 30 min, 62% of **A2a** was hydrolyzed to **2a** and **A** (Figure 6). After 4 h, the hydrolysis of **A2a** was close to the equilibrium (Figure 6), with a 65% conversion of **A2a** to constituents **2a** and **A**. Interestingly, with respect to the total quantity of benzaldehyde **2a** obtained by hydrolysis of **A2a** after 4 h, a 29% uptake of **2a** inside **CR**_**6**_ was measured. The uptake of **2a** inside **CR**_**6**_ suggests that probably the benzaldehyde **2a** behaves like a reversible inhibitor for the capsule **CR**_**6**_, slowing down its catalytic activity. Under the same conditions but in the absence of **CR**_**6**_, imine **A2a** was stable over time and no hydrolysis products were detected (Supporting Information). These data strongly indicate that the hydrolysis of **A2a** occurs inside **CR**_**6**_ due to its catalytic activity. This was confirmed by the finding that, in the presence of DMSO, a solvent able to break down the capsule,^15^ no conversion of **A2a** into **2a** and **A** was observed. In contrast, the hydrolysis of **A2b** to **2b** and **A** in the presence of **CR**_**6**_ was slower (Figure 6); in fact, the equilibrium was reached after 72 h with a 40% of conversion of **A2b** to **2b** and **A**.

**Figure 7:**
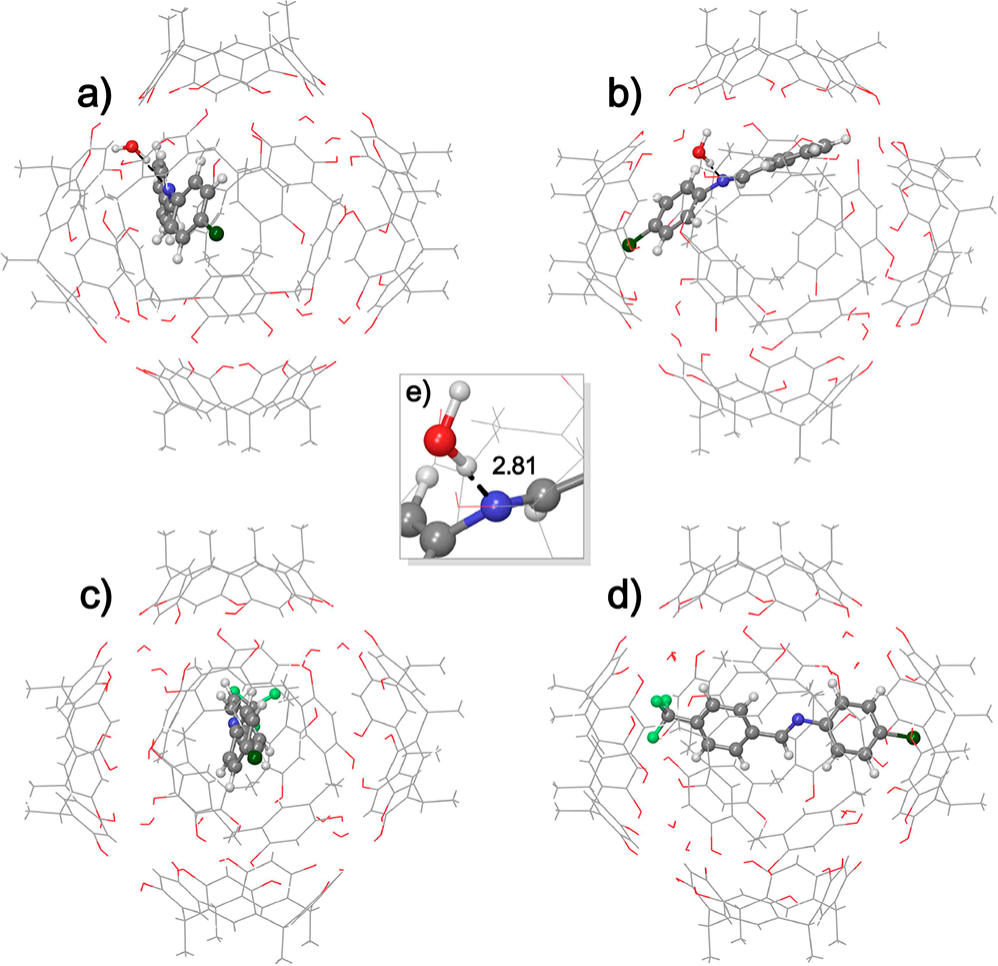
Different views of the optimized geometries of complexes (a,b) **A2a**⊂**CR**_**6**_ and (c,d) **A2a**⊂**CR**_**6**_. (e) Particular H-bonding interaction of **A2a** with the bridged water molecule of **CR**_**6**_.

**Figure 8:**
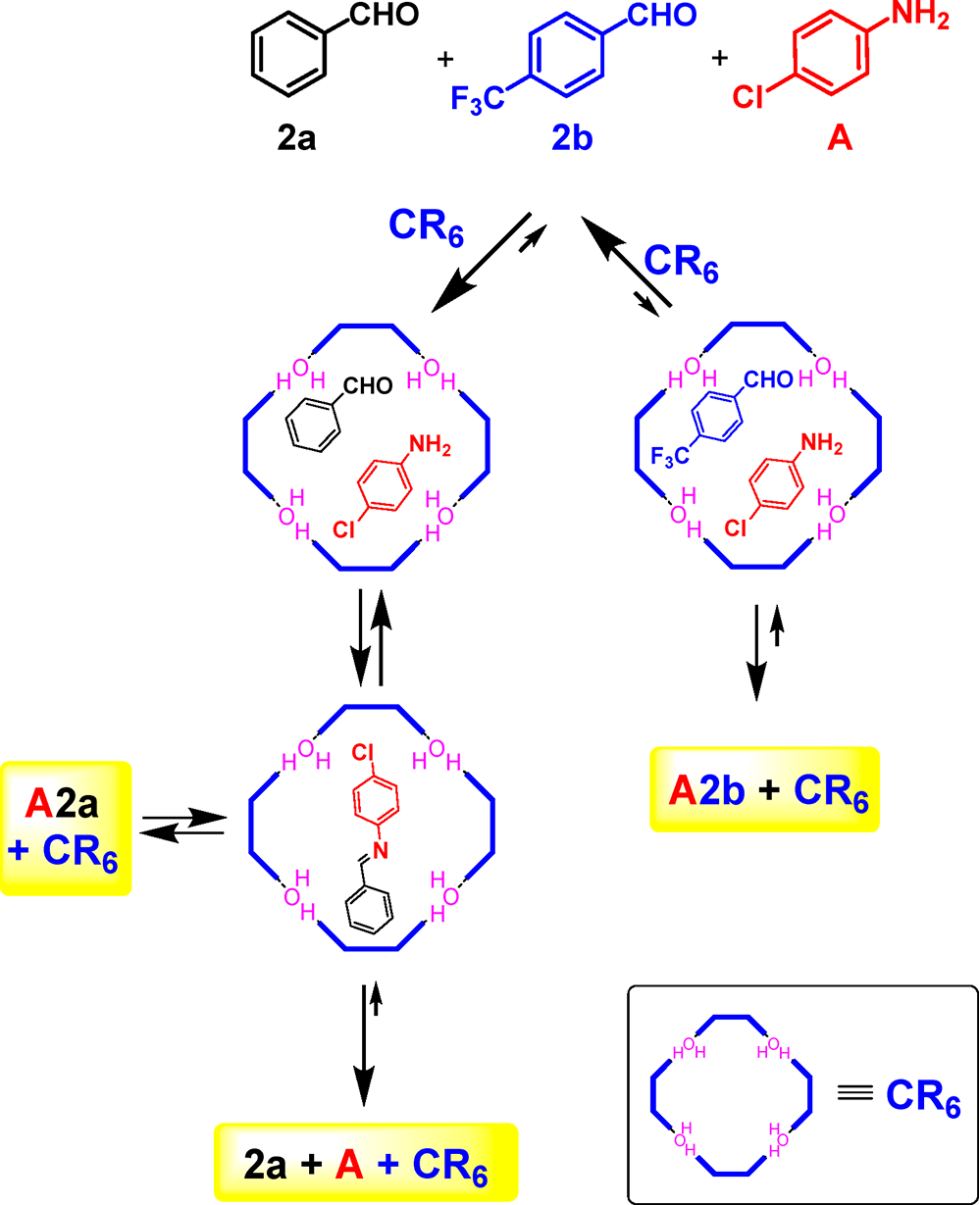
“Predatory” mechanism proposed for the adaptation of imine constituents in the DCL in Scheme 1 and Figure 3b.

On this basis, we can explain the origin of the kinetic and thermodynamic modulation of the DCL in Scheme 2 (Figure 4b). Imine **A2a** is accumulated preferentially during the early stage of the reaction in Scheme 2 (Figures 7 and 8), due to the preferential encapsulation of **2a** inside the cavity of **CR**_**6**_ (Figures 5–8), which catalyzes the formation of **A2a**. In the presence of a significant quantity of **A2a**, its hydrolysis starts quickly inside the **CR**_**6**_ capsule (Figure 8), catalyzed by the inherent Brønsted acidity of the capsule and its ability to stabilize cationic intermediates and transition states.^24^ On the other hand, the hydrolysis of imine **A2b** inside **CR**_**6**_ is less favored, probably because of the lower affinity of the capsule for the imine **A2b**. In fact, qNMR experiments revealed a very low level of uptake of **A2b** inside **CR**_**6**_ of 5%, immediately after mixing of **A2b** and **CR**_**6**_, whereas imine **A2a** is encapsulated to a greater extent (45%). In silico calculations were in accord with these results (Figure 7 and Supporting Information). Quantum-mechanical calculations (Supporting Information) indicate an enthalpic stabilization of −22.14 kcal/mol and a Gibbs free energy stabilization of −8.08 kcal/mol for the formation of the **A2a**⊂**CR**_**6**_^25^ complex. However, the formation of the complex **A2b**⊂**CR**_**6**_ is unfavored in enthaplic as well as Gibbs free energy terms.^25^

Natural bond orbital (NBO)^26^ and noncovalent interaction (NCI)^27^ (see Supporting Information) analyses were performed on complexes **A2a**⊂**CR**_**6**_ and **A2b**⊂**CR**_**6**_ to identify the second-order interactions between the capsule and the imine. Second-order perturbation theory (SOPT) analysis of the FOCK matrix in NBO basis clarified that the better binding affinity of **A2a** is principally due to a strong hydrogen bonding interaction (Figure 7e) between the nitrogen atom of the imine moiety of **A2a** and a bridged water molecule of **CR**_**6**_. This strong^28^ H-bonding interaction shows a N···OH_2_ distance of 2.81 Å (Figure 7e) and a N···H–OH angle of 167° and accounts for 49% of the total stabilization energy of the **A2a**⊂**CR**_**6**_ complex (51% represents the van der Waals interactions). Regarding the **A2b**⊂**CR**_**6**_ complex, because of the steric demand imposed by the trifluoromethyl group, **A2b** is forced to stay on the axis that joins two vertexes of the capsule (Figure 7c,d), with the −CF_3_ group pointing inside the cavity of a resorcinarene macrocycle (Figure 7c,d). In this position, the imine moiety of **A2b** is too far from the bridged water molecules of **CR**_**6**_ and cannot establish any H-bonding interactions.

In summary, these results show that this is a rare example of kinetic and thermodynamic adaptation of a DCL, in which the intraspecific “predatory” effect^29^ of the catalyst (**CR**_**6**_) on one of the constituents (**A2a**) plays a crucial role.

**Figure 9:**
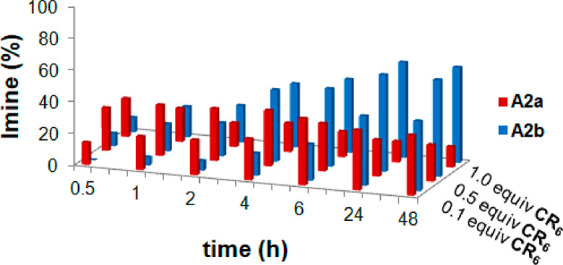
Evolution of the distribution of **A2a** and **A2b** with different amounts of capsule **CR**_**6**_.

Now, in order to corroborate this assumption, we studied the distribution of the constituents **A2a** and **A2b** in the presence of lower quantities of “predator” **CR**_**6**_ (Figure 9). When the reaction in Scheme 2 was performed in the presence of a lower quantity of **CR**_**6**_ (0.5 equiv), the kinetically favored imine **A2a** was prevalent up to 2 h (Figure 9), a time significantly longer than that observed in the presence of 1 equiv of **CR**_**6**_ (0.5 h). Under these conditions, the thermodynamic imine **A2b** began to prevail at 4 h, and finally, the quantity of **A2a** after 48 h was slightly higher than that obtained in the presence of 1 equiv of the capsule (see Figure 9). Decreasing the quantity of capsule **CR**_**6**_ to 0.1 equiv, the imine **A2a** prevailed for up to about 20 h, with a yield of about 40%, higher than that observed in the presence of 0.5 (23%) and 1.0 equiv (15%) of **CR_6_**. This result clearly indicates that the stability of the imine **A2a** in the DCL increases by decreasing the quantity of capsule **CR**_**6**_, showing in this way its predatory effect on **A2a**.

Interestingly, when the *p*-nitrobenzaldehyde **2c** was used instead of **2b**, the DCL of the components **A**/**2a**/**2c** showed an analogous behavior (see Supporting Information). In detail, in the presence of **CR**_**6**_, an adaptation of constituents was thermodynamically driven by the hexameric capsule toward the imine **A2c** derived by aldehyde bearing an electron-withdrawing group on the phenyl ring, whereas the constituent **A2a** remained the kinetically favored one (Figures S20–S23).

In contrast, when the *p*-OMe-benzaldehyde **2d** was used together with **2a** and **A** as a component of the DCL, the formation of imines **A2a** and **A2d** was observed in very low yields in the presence of **CR**_**6**_.

Interestingly, in this case, imine **A2a** was favored over time (Figures S24–S27).

### Adaptation of the 2 × 1 DCL of Imines **B2a** and **B2b** to the Presence of the Hexameric Capsule

**Scheme 4:**
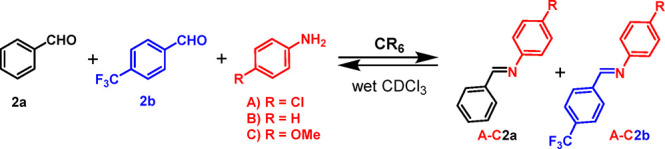
Dynamic Library of Three Components **2a**, **2b**, and **A**–**C** and Two Constituents in the Presence of **CR**_**6**_

**Figure 10:**
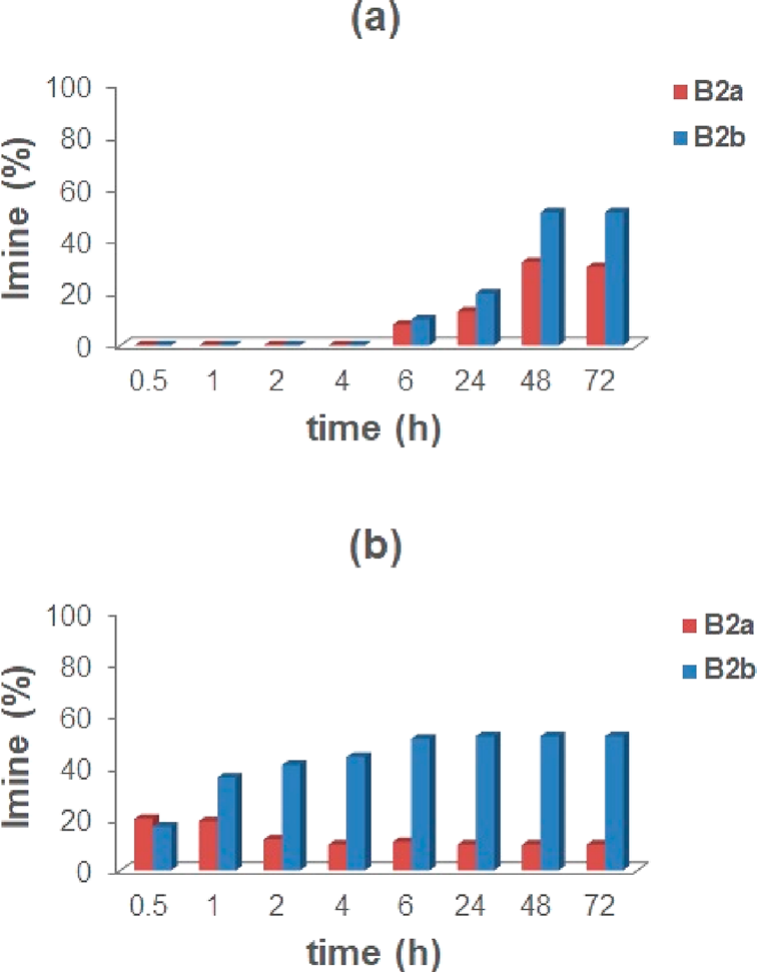
Distribution of imine constituents **B2a** and **B2b** in the DCL in Scheme 4, without (a) and with (b) capsule **CR**_**6**_.

Next, we investigated a DCL starting with aniline **B** and aldehydes **2a** and **2b** (R *=* CF_3_) (Scheme 4) as components. Imine constituents **B2a** and **B2b** were formed immediately after mixing (Figure 10b), whereas in the absence of a capsule, the reaction proceeded more slowly (Figure 10a). With regard to the imine distribution, **B2a** was kinetically favored, reaching 20% conversion after 0.5 h (Figure 10b). After 30 min, **B2a** started to decrease as **B2b** increased, whereas the equilibrium was reached after 6 h with a **B2b**/**B2a** ratio of 52/10. In summary, even when the aniline components are changed, the benzaldehyde-derived imine **B2a** results in the kinetically favored product and the imine obtained by **2b** results in the thermodynamic one.

**Figure 11:**
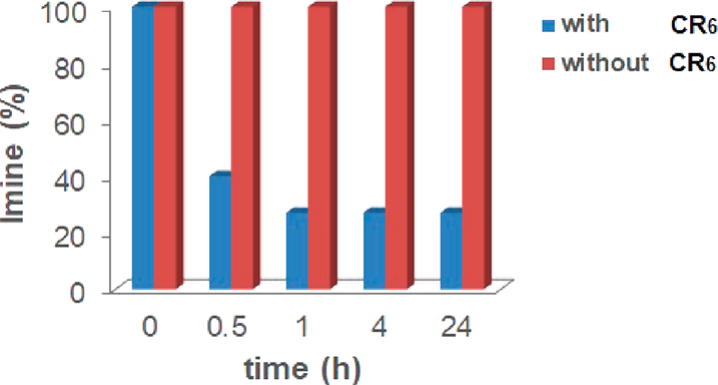
Hydrolysis of imine **B2a** in the presence and in the absence of capsule **CR**_**6**_.

The decrease of the quantity of imine **B2a** in the experiment in Figure 10b suggests a possible predatory action of the capsule **CR**_**6**_ on the imine **B2a**. Encouraged by this hypothesis, we evaluated the stability of **B2a** in water-saturated CDCl_3_ in the presence or in the absence of capsule **CR**_**6**_ (Figure 11). Imine **B2a** was hydrolyzed in the presence of capsule **CR**_**6**_, and the reaction reached equilibrium with a 73% conversion of **B2a** after 1 h (Figure 11), whereas in the absence of capsule **CR**_**6**_, imine **B2a** was stable in water-saturated CDCl_3_ at room temperature.

### Adaptation of the 2 × 1 DCL of Imines **C2a** and **C2b** to the Presence of the Hexameric Capsule

**Figure 12:**
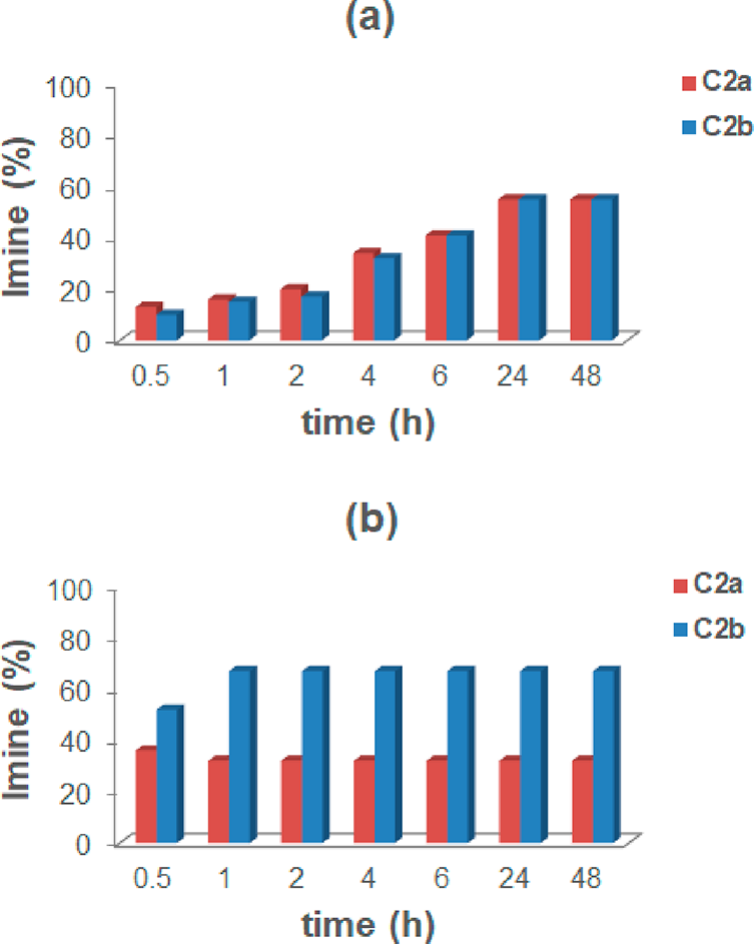
Distribution of imines **C2a** and **C2b** in the dynamic system generated by aldehydes **2a** and **2b** and aniline **C**, without (a) and with (b) capsule **CR**_**6**_.

Starting from *p*-methoxyaniline **C**, which shows a basicity (p*K*_a_ = 5.36) higher than that of **A** and **B**, and aldehydes **2a/2b** in Scheme 4, the DCL adapts in the presence of **CR**_**6**_ (Figure 12), but smaller kinetic effects were observed.

A comparison of the distribution over the time of the constituents **C2a** and **C2b** (Figure 12), with and without capsule, clearly shows that, in the presence of **CR**_**6**_ (Figure 12b), the equilibrium was reached after 1 h with a **C2b**/**C2a** ratio of 67/32.

On the other hand, the reaction without a capsule progressed more slowly and gave an equimolar mixture of imines **C2a** and **C2b** along the reaction time (Figure 12a).

**Figure 13:**
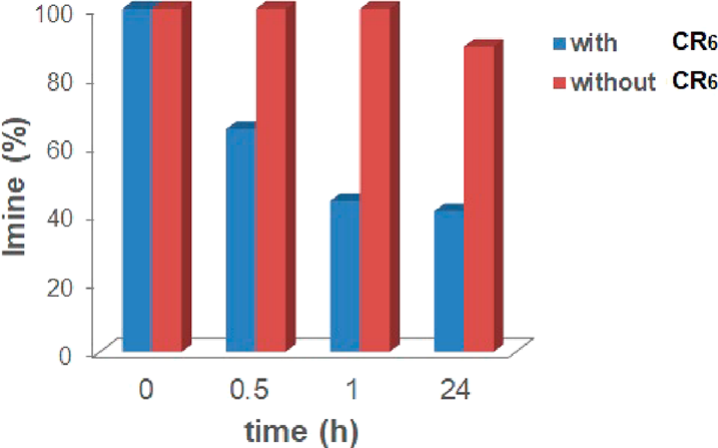
Hydrolysis of imine **C2a** in the presence and in the absence of capsule **CR**_**6**_.

A close inspection of the kinetics in Figure 12a,b indicates that, in the absence of **CR**_**6**_, imine **C2a** increases over the time until it reaches an equilibrium value of 55% after 24 h. In contrast, when capsule **CR**_**6**_ was present, a conversion of 36% of **C2a** was obtained after 30 min, but after 1 h, **C2a** started to decrease until it reached an equilibrium value of 32% after 6 h. Again, this behavior suggests a predatory action of the capsule **CR**_**6**_ on **C2a**. Thus, in order to confirm this assumption, we evaluated the stability of **C2a** in water-saturated CDCl_3_ in the presence or in the absence of capsule **CR**_**6**_ (Figure 13).

Imine **C2a** was rapidly hydrolyzed in the presence of capsule **CR**_**6**_, and the reaction reached the equilibrium with a 59% conversion of **C2a** after 1 h, whereas in the absence of capsule **CR**_**6**_, imine **C2a** was stable in water-saturated CDCl_3_ at room temperature.

In summary, these results (Figures 12 and 13) indicate that the DCL of imines **C2a** and **C2b** also adapts in the presence of capsule **CR**_**6**_ by a predatory effect of the capsule on one of the imine constituent.

### Adaptation of 2 × 1 DCLs of Imines **E2a/E2b** and **A2a/E2a** to the Presence of the Hexameric Capsule. Substrate Selectivity: Aromatic versus Aliphatic Amine

**Scheme 5:**
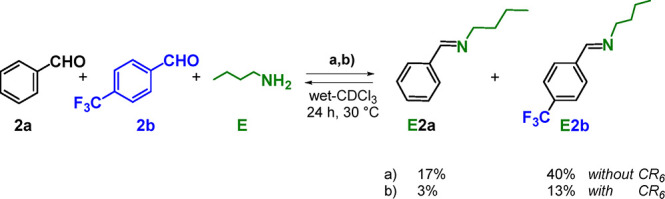
Dynamic Library of Three Components **2a**, **2b**, and **E** and Two Constituents in the Presence or in the Absence of **CR**_**6**_

One of the aims of the enzyme mimicry is to achieve the substrate selectivity typical of natural systems. Thus, we envisioned to study the substrate selectivity of the hexameric capsule in the presence of a mixture constituted by aromatic and aliphatic amines. First, we analyzed the modulation of the DCL in Scheme 5 starting with benzaldehyde **2a**, *p*-trifluoromethylbenzaldehyde **2b**, and *n*-butylamine **E** in the presence or in the absence of a capsule in water-saturated CDCl_3_ using a concentration of 42.3 mM each of **2a**/**2b**/**E/CR**_**6**_ at 30 °C.

The results in Table S9 and Scheme 5 clearly show that the formation of imines **E2a** and **E2b** is favored in the absence of **CR**_**6**_, with 17 and 40% yield, respectively, after 24 h. However, in the presence of **CR**_**6**_, **E2a** and **E2b** were obtained in 3 and 13% yield, respectively. This is in contrast to the results reported in Figure 4, in which the formation of imines **A2a** and **A2b** starting by an aromatic amine such as *p*-chloroaniline **A** and aldehydes **2a** and **2b** is favored in the presence of **CR**_**6**_.

Thus, these results indicate that the capsule suppresses the reactivity of an aliphatic amine such as the *n*-butylamine **E** toward the aldehydes **2a** and **2b**. This conclusion can be explained on the basis of the data previously reported by Tiefenbacher.^18a^ In fact, *n*-butylamine **E** is protonated by the capsule to an extent of 80%, and the resulting *n*-butylammonium cation is stabilized inside the capsule by cation···π interactions.^18a,20a^ Consequently, the percentage of free neutral *n*-butylamine is low, and the imine formation (Scheme 5) is suppressed. In contrast, *p*-chloroaniline **A**, which shows a lower basicity (p*K*_a_ = 3.8), is not protonated by **CR**_**6**_(18a) and consequently shows a remarkable reactivity when co-confined with aldehydes.

**Scheme 6:**
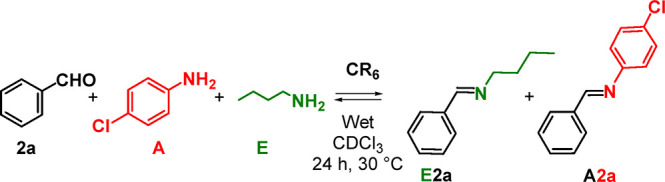
Dynamic Library of Three Components **2a**, **A**, and **E** in the Presence of **CR**_**6**_: *p-*Chloroaniline versus *n*-Butylamine Substrate Selectivity

**Figure 14:**
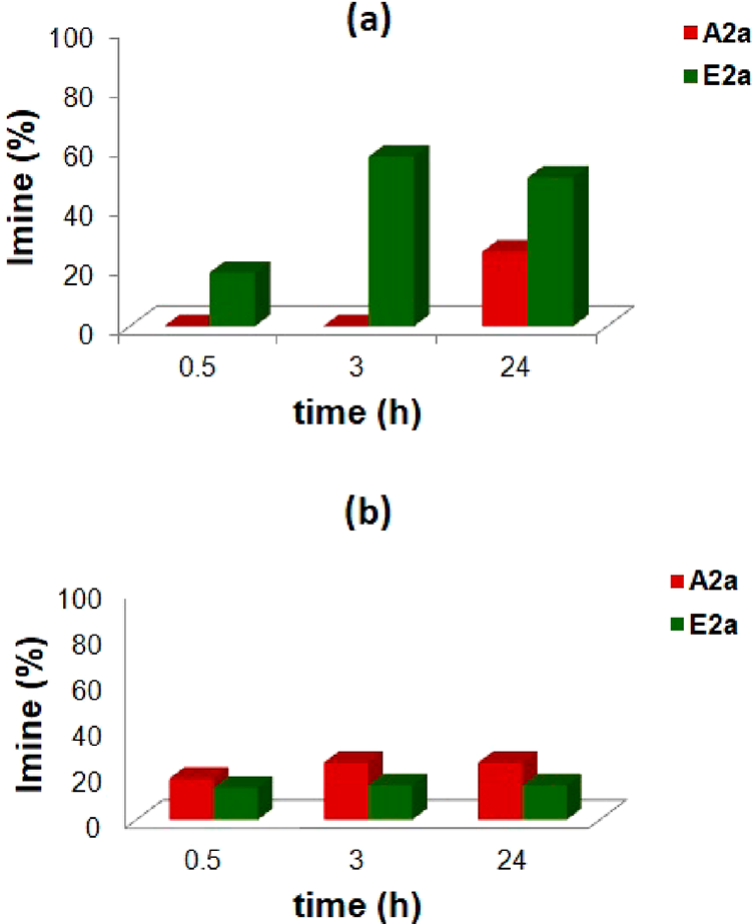
Distribution of imine constituents **A2a** and **E2a** in the DCL, without (a) and with (b) capsule **CR**_**6**_.

With these results in hand, we performed a competition experiment (Scheme 6) in which *p*-chloroaniline **A** and *n*-butylamine **E** compete for benzaldehyde **2a**. In detail, **A**, **E**, and **2a** were mixed in 1/1/1 ratio (42.3 mM) in wet CDCl_3_ in the presence or in the absence of **CR**_**6**_. As reported in Supporting Information (Table S10 and Figures S38–S40) and Scheme 6, in the absence of capsule **CR**_**6**_, imines **E2a** and **A2a** were formed in 50 and 25% yield, respectively (Figure 14). However, in the presence of **CR**_**6**_, the selectivity order was reversed to 15/25 in favor of **A2a** (Figure 14).

In summary, is clear that the **CR**_**6**_ capsule is able to host the scarcely basic *p*-chloroaniline **A** in its neutral form, thus promoting the formation of the corresponding imine in the presence of an aldehyde. When the more basic *n*-butylamine is used, the corresponding ammonium form is obtained after protonation inside the capsule stabilized by cation···π interactions. In this way, the formation of imine is suppressed. This is an intriguing example of substrate selectivity that the **CR**_**6**_ capsule exerts toward aliphatic versus aromatic amines, by decreasing the reactivity of the former toward the formation of imines.

### Adaptation of the 2 × 2 DCL of Imines **A2a**, **A2b**, **B2a**, and **B2b** to the Presence of the Hexameric Capsule

**Scheme 7:**
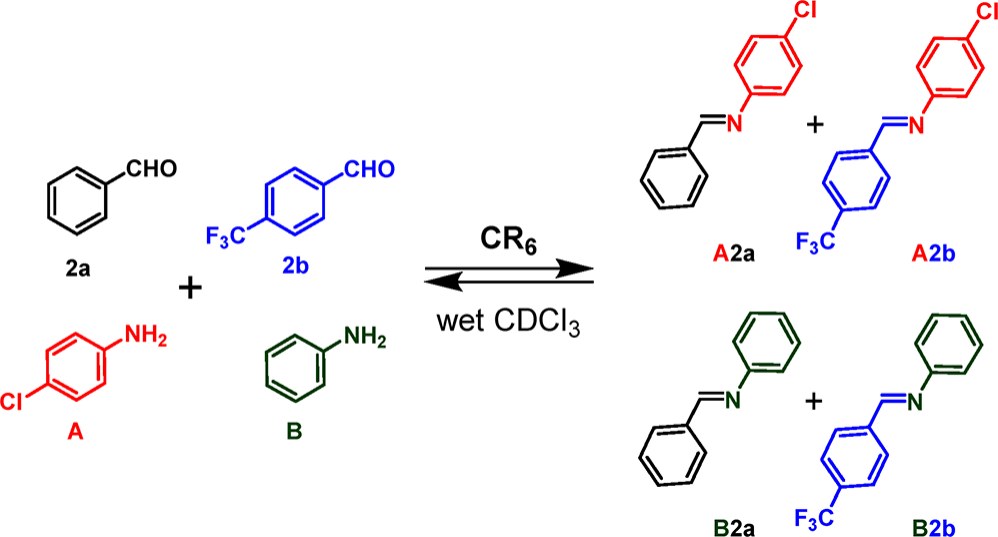
Dynamic Library of Four Components **2a**, **2b**, **A**, and **B** and Four Constituents in the Presence of **CR**_**6**_

At this point, our attention was focused on a more complex DCL formed by four constituents derived by four components (Scheme 7). We mixed equimolar amounts of aldehydes **2a** and **2b** with anilines **A** (*p*-Cl) and **B** (*p*-H) (Scheme 7), and we monitored the adaptation of the DCL of the four imine constituents in the presence of **CR**_**6**_.

**Figure 15:**
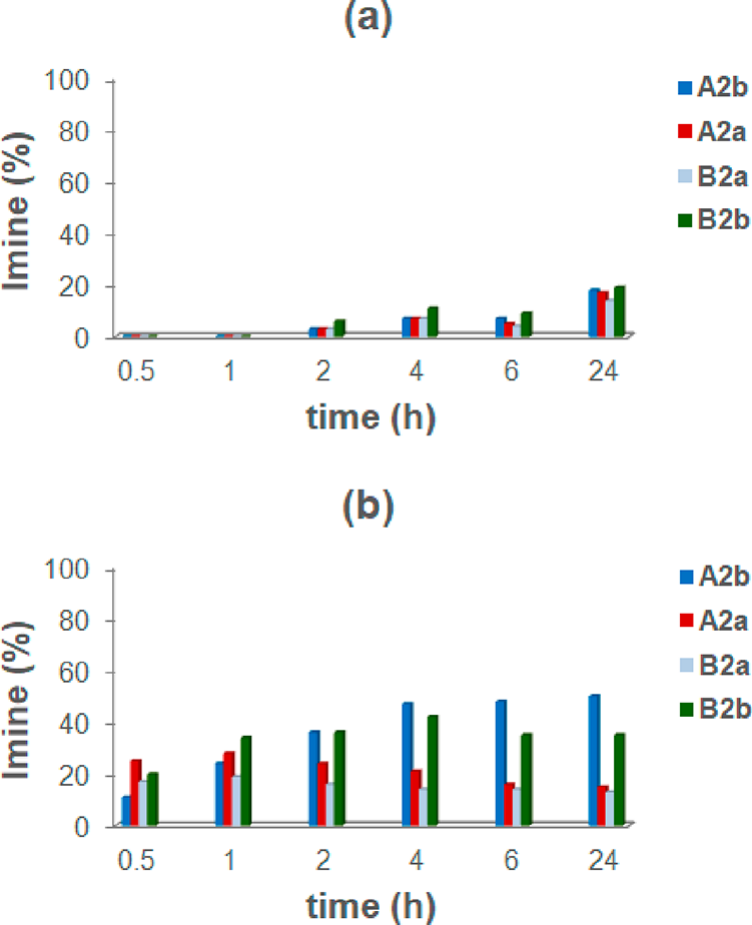
Distribution of imines in the dynamic systems from **2a**, **2b**, **A**, and **B**, without (a) and with (b) capsule **CR**_**6**_.

In the presence of a capsule (Figure 15b), a mixture of all four imines was formed immediately after mixing. Imine constituents **A2a** and **B2b** were the main components, followed by **B2a** and **A2b** in a distribution of 25, 20, 17, and 11%, respectively (Figure 15b). Two hours later, imines **A2b** and **B2b** started to increase as **A2a** and **B2a** decreased. Thus, imines **A2a** and **B2a** resulted in the kinetic products, whereas products **A2b** and **B2b** emerged under thermodynamic conditions.

When the reaction was performed without the capsule, the composition of the library showed no substantial preference for the distribution of the components (Figure 15a). When the reaction in Scheme 7 was performed in the presence of a lower quantity of **CR**_**6**_ (0.5 equiv, Figure S42), the kinetically favored imine **A2a** survived longer, thus also in this case, the predatory effect of the capsule toward **A2a** was suppressed (Figure S42).

In accordance with one of the aims of enzyme mimicry,^3^ to work selectively in the presence of a complex mixture of reagents, the results reported in Figure 15 clearly show that the hexameric capsule **CR**_**6**_ is able to work selectively in the presence of complex mixtures of substrates, due to a fine control of the encapsulated species, leading to the selective formation of specific imines. In order to further corroborate this result, we performed a new 2 × 2 experiment, changing the amine and aldehyde components.

### Adaptation of the 2 × 2 DCL of Imines **C2b**, **C2d**, **D2b**, and **D2d** to the Presence of the Hexameric Capsule

**Scheme 8:**
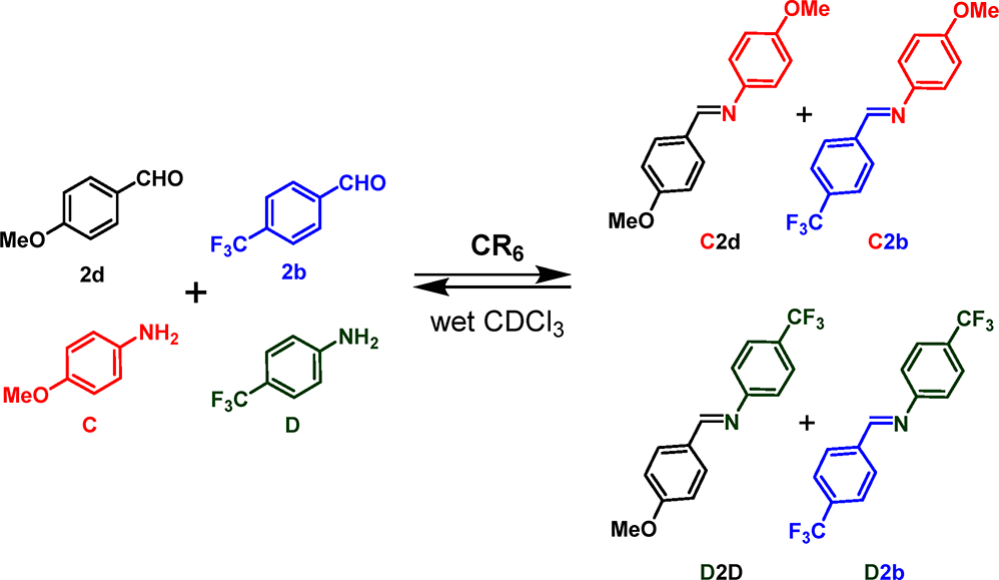
Dynamic Library of Four Components **2d**, **2b**, **C**, and **D** and Four Constituents in the Presence of **CR**_**6**_

**Figure 16:**
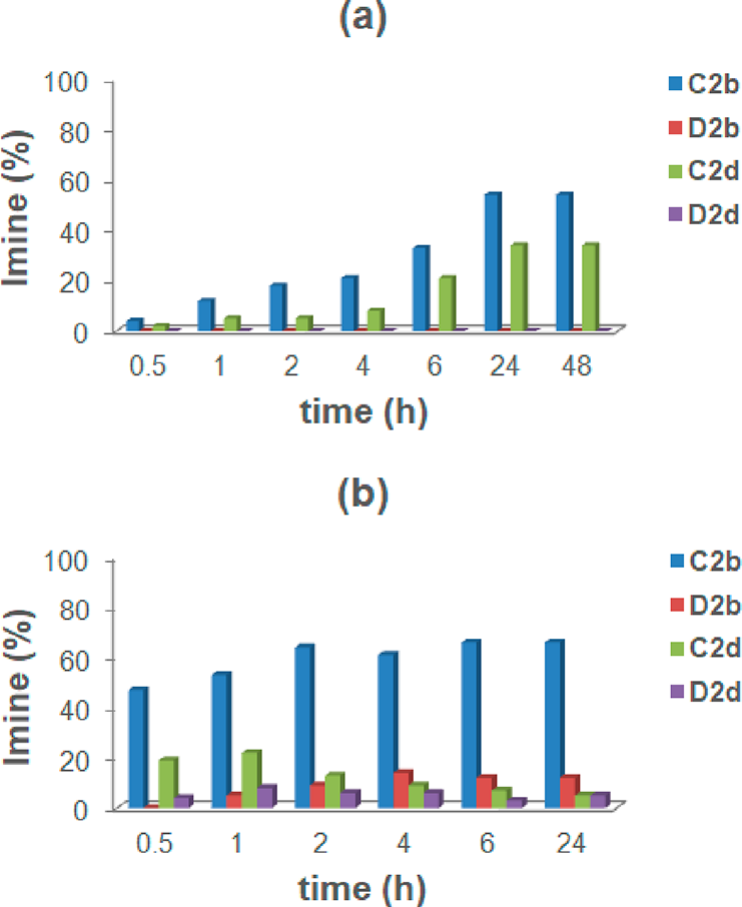
Distribution of imines in the dynamic systems from **2b**, **2d**, **C**, and **D**, without (a) and with (b) capsule **CR**_**6**_.

Finally, we focused our attention toward a 2 × 2 DCL starting with components bearing an electron-donating OMe group (**2d/C**) and an electron-withdrawing trifluoromethyl group (**2b/D**) (Scheme 8). As in all of the above cases, the formation of imines was more efficient in the presence of the **CR**_**6**_ capsule (Figure 16).

After 0.5 h, imine constituents **C2b** and **C2d** from *p*-methoxyaniline **C** were detected as the main components of the mixture in a **C2b**/**C2d** ratio of 47/19, whereas imines **D2b** and **D2d**, obtained from the less reactive *p*-trifluoromethylaniline **D**, were present in almost negligible quantities. Over time, an increase in the quantity of **C2b** was observed, which after 24 h was the most abundant constituent of the mixture, with a composition of **C2b**, **D2b**, **C2d**, and **D2d** of 66, 12, 5, and 5%, respectively. In summary, by cross-referencing the data in Figures 12 and 15, it becomes clear that no kinetic preference was observed in DCL systems in which the *p*-OMe-benzaldehyde **2d** or *p*-OMe-aniline **C** are present as components.

## Conclusions

In conclusion, we have demonstrated that dynamic covalent libraries of imine constituents are able to adapt their composition in response to the presence of the hexameric resorcinarene capsule **CR**_**6**_. The DCL of imines **A2a** and **A2b** formed by benzaldehyde **2a**, *p*-CF_3_-benzaldehyde **2b**, and *p*-chloroaniline **A** adapts its composition in the presence of **CR**_**6**_, showing a kinetic and thermodynamic preference of the constituents. In particular, the kinetically favored constituent **A2a**, obtained from benzaldehyde **2a**, is preferentially formed immediately after mixing, due to the preferred inclusion of **2a** inside **CR**_**6**_. Surprisingly, the capsule shows a predatory behavior toward imine **A2a**, which is quickly hydrolyzed to components **A** and **2a** inside the capsule. On the other hand, imine constituent **A2b**, obtained from *p*-CF_3_-benzaldehyde **2b**, is hydrolyzed slower than **A2a**. Uptake studies show that, after the hydrolysis of imine **A2a**, the benzaldehyde component **2a** remains included in the capsule **CR**_**6**_. Interestingly, the hexameric capsule **CR**_**6**_ shows an analogous predatory action on other benzaldehyde-based imines such as **B2a** and **C2a** derived from aniline **B** and *p*-OMe-aniline **C**, respectively. Finally, more complexes of 2 × 2 DCL systems adapt to the presence of the hexameric capsule, showing a thermodynamic and kinetic modulation of the constituents and leading to a good selectivity (up to 66%) for one of them.

## Supporting Information Available

Synthesis and characterization of imines, experimental procedures for the synthesis of dynamic imine libraries, kinetic experiments on DCLs, ^1^H NMR spectra of DCLs of imines as a function of time, proof of encapsulation of aldehydes by 1D and 2D experiments, ^1^H NMR experiments as proof of the predatory effect of the capsule (PDF)
