# Supplementary material for: Kinetic and Thermodynamic Modulation of Dynamic Imine Libraries Driven by the Hexameric Resorcinarene Capsule
Source: J Am Chem Soc. 2020 Aug 7;142(35):14914–23. doi: 10.1021/jacs.0c04705 (PMC8010792; doi:10.1021/jacs.0c04705)
Supplement: Supplementary file 1 — ja0c04705_si_001.pdf [file ja0c04705_si_001.pdf]

Supporting Information

Kinetic and Thermodynamic Modulation of Dynamic Imine Libraries Driven by the Hexameric Resorcinarene Capsule

Stefania Gambaro, Carmen Talotta, Paolo Della Sala, Annunziata Soriente, Margherita De Rosa,* Carmine Gaeta* and Placido Neri

Laboratory of Supramolecular Chemistry, Dipartimento di Chimica e Biologia "A. Zambelli", Università di Salerno, Via Giovanni Paolo II, I-84084, Fisciano (SALERNO), Italy.

Table of Contents

1. General Remarks **1**

2. General Procedures **1**

2.1 General procedure for synthesis of imines *1*

2.2 General procedure for self-sorting system in the presence of capsule CR_6 *2*

2.3 General procedure for self-sorting system without capsule CR_6 *2*

2.4 Reaction Monitoring *2*

3. Aldehydes, anilines and imines used in the dynamic combinatorial libraries **3**

4. Characterization of novel compounds **3**

5. Two – component reactions **10**

5.1 Formation of A2a by aldehyde 2a and aniline A *10*

5.1.1 Formation of A2a by aldehyde 2a and aniline A in absence of capsule CR_6. *10*

5.1.2 Formation of imine A2a by aldehyde 2a and aniline A in presence of capsule CR_6 *11*

5.2 Formation of A2b by aldehyde 2b and aniline A. *14*

5.2.1 Formation of A2b by aldehyde 2b and aniline A in absence of capsule CR_6 *14*

5.2.2 Formation of A2b by aldehyde 2b and aniline A in presence of capsule CR_6 *15*

6. Evolution of dynamic imine libraries as a function of time **18**

6.1 Competitive reaction between equivalent amounts of 2a, 2b and A without capsule CR_6 *18*

6.2 Competitive reaction between equivalent amounts of 2a, 2b and A in the presence of capsule CR_6 *19*

6.3 Evidence of the capsule effect on the imine distribution in DCL 2a, 2b and A *22*

6.4 Competitive reaction between equivalent amounts of 2a, 2c and A with and without capsule CR_6 *26*

6.5 Competitive reaction between equivalent amounts of 2a, 2d and A with and without capsule CR_6 *31*

6.6 Competitive reaction between equivalent amounts of 2a, 2b and B with and without capsule CR_6 *35*

6.7 Competitive reaction between equivalent amounts of 2a, 2b and C with and without capsule CR_6 *39*

6.8 Competitive reaction between equivalent amounts of 2a, 2b and E with and without capsule CR_6 *43*

6.9 Competitive reaction between equivalent amounts of 2a, 2b, A and B with and without capsule CR_6 *52*

6.10 Competitive reaction between equivalent amounts of 2b, 2d, C and D with and without capsule CR_6 *58*

7. Competitive uptake experiment **62**

8. Proofs of the encapsulation of 2a **63**

9. Proofs of the encapsulation of 2b **68**

10. Proofs of the encapsulation of A **72**

11. Predatory effect of CR_6. Stability of imines in presence of CR_6 **75**

11.1 General experimental conditions *75*

11.2 Stability of imine A2a in presence and in absence of CR_6 *75*

11.3 Uptake of 2a inside the capsule after hydrolysis of A2a in presence of CR_6 *77*

11.4 Stability of imine A2b in presence and in absence of CR_6 *81*

11.5 Uptake of 2b inside the capsule after hydrolysis of A2b in presence of CR_6 *83*

11.6 Stability of imine B2a in presence and in absence of CR_6 *85*

11.7 Stability of imine C2a in presence and in absence of CR_6 *88*

12. Control experiments on concentration influence **91**

13. Computational studies **92**

14. Non-covalent Interactions (NCI studies) **110**

15. References **111**

1. General Remarks

All chemicals were reagent grade and were used without further purification. Solvents were purchased from Aldrich. Reaction temperatures were measured externally; reactions were monitored by ^{1}H NMR spectroscopy and by TLC on Merck silica gel plates (0.25 mm) and visualized by UV light. Flash chromatography was performed on Merck silica gel (60, 40-63 μm). NMR spectra were recorded on Bruker Avance-600 spectrometer [600.13 MHz (^{1}H) and 150.03 MHz (^{13}C)], Bruker Avance-400 spectrometer [400 (^{1}H) and 100.57 MHz (^{13}C)], Bruker Avance-300 spectrometer [300 (^{1}H) and 75.48 MHz (^{13}C)]; chemical shifts are reported relative to the residual solvent peak ($CHCl_3$: δ 7.26, $CDCl_3$: δ 77.23). DOSY experiments were performed on a Bruker Avance-600 spectrometer equipped with 5 mm PABBO BB|19F-1H\D Z-GRD Z114607/0109. The standard Bruker pulse program, ledbpgp2s, employing a double stimulated echo sequence and LED, bipolar gradient pulses for diffusion, and two spoil gradients were utilized. Diffusion times were 150 ms, eddy current delay was 5 ms, gradient recovery delays were 0.2 ms, and gradient pulse was 1400 ms. Individual rows of the quasi-2D diffusion databases were phased and baseline corrected. $CDCl_3$ used for experiments was passed through activated 3Å molecular sieves and alumina basic oxide to remove water and DCl traces and was preserved in a brown glass vial to keep out of the light. The quantitative ^{1}H NMR analysis was performed by using TCE (tetrachloroethane) as internal standard, the optimisation of NMR parameters were performed according to literature data.[1] High-resolution mass spectra (HRMS) were acquired using a Bruker Solaris XR Fourier transform ion cyclotron resonance mass spectrometer equipped with a 7 T refrigerated actively-shielded superconducting magnet. The samples were ionized in positive ion mode using the ESI ion source (Bruker Daltonik GmbH, Bremen, Germany). The mass spectra were calibrated externally using a NaTFA solution in positive ion mode. Low resolution mass spectral analyses were carried out using an electrospray spectrometer Waters 4 micro quadrupole. A linear calibration was applied. All final compounds purity was determined by elemental analysis on a Flash EA 1112 Series with Thermal Conductivity Detector, for C, H, N, and S. The final compounds purity was found to be >95% when analyzed. Water saturated deuterated chloroform was prepared as reported in the literature.[2] Resorcinarene (**1**), was synthesized according to literature procedures.[3]

2. General Procedures

2.1 General procedure for synthesis of imines[4]

Imines were independently prepared by condensation of the aldehydes and amines according to the following procedure. A mixture of the selected aniline (2 mmol) and aldehyde (2 mmol) in ethanol (12 mL) was refluxed upon stirring for 8 h. Then, the reaction mixture was refrigerated and the desired imine was obtained as solid, after filtration.

Spectroscopic data of imines **A2a**, [5] **A2c**,[6] **A2d**,[7] **B2a**,[5] **B2b**,[8] **C2a**,[9] **C2b**,[10] **C2d**[11], **E2a**[16], **E2b**[16] matched with that reported in literature.

2.2 General procedure for self-sorting system in the presence of capsule CR_6

Resorcinarene **1** (281.6 mg, 254.7 μmol, 6 eq.) was weighed in a 4 ml vial. Then, 1 mL of water saturated deuterated chloroform was added and the mixture was warmed at 50°C until clarification (ca 5 min). Next, the components of the system, aldehydes and anilines, were added simultaneously in the same equivalent (0.0423 mmol, 1 equiv.). The reaction mixture was vigorously stirred (1400 rpm) at the reported temperature T and the evolution of the system composition was monitored by ^{1}H NMR as a function of time.

2.3 General procedure for self-sorting system without capsule CR_6

The components of the DCL system were dissolved in equimolar amounts (0.0423 mmol) in water saturated deuterated chloroform (1mL) and stirred (1400 rpm) at the reported temperature T. The distribution of the DCL components was monitored by ^{1}H NMR until no change in composition was detected.

2.4 Reaction Monitoring

Aliquots of the reaction mixture (30 μL) were taken at different times and diluted with 470 μL of $CDCl_3$. After adding TCE (1 μL) as internal standard, the reaction mixture was monitored by ^{1}H NMR spectroscopy. d1 parameter was set to 3 x T1. The ratios of the DCL components were determined by integration of the corresponding resonance signals in the spectrum by comparison with the internal standard TCE. For the reaction in the presence of **CR_6**, the ratios were determined after addition of DMSO (2 μL) to the reaction aliquot, in order to disaggregate the capsule.[2, 12]

3. Aldehydes, anilines and imines used in the dynamic combinatorial libraries

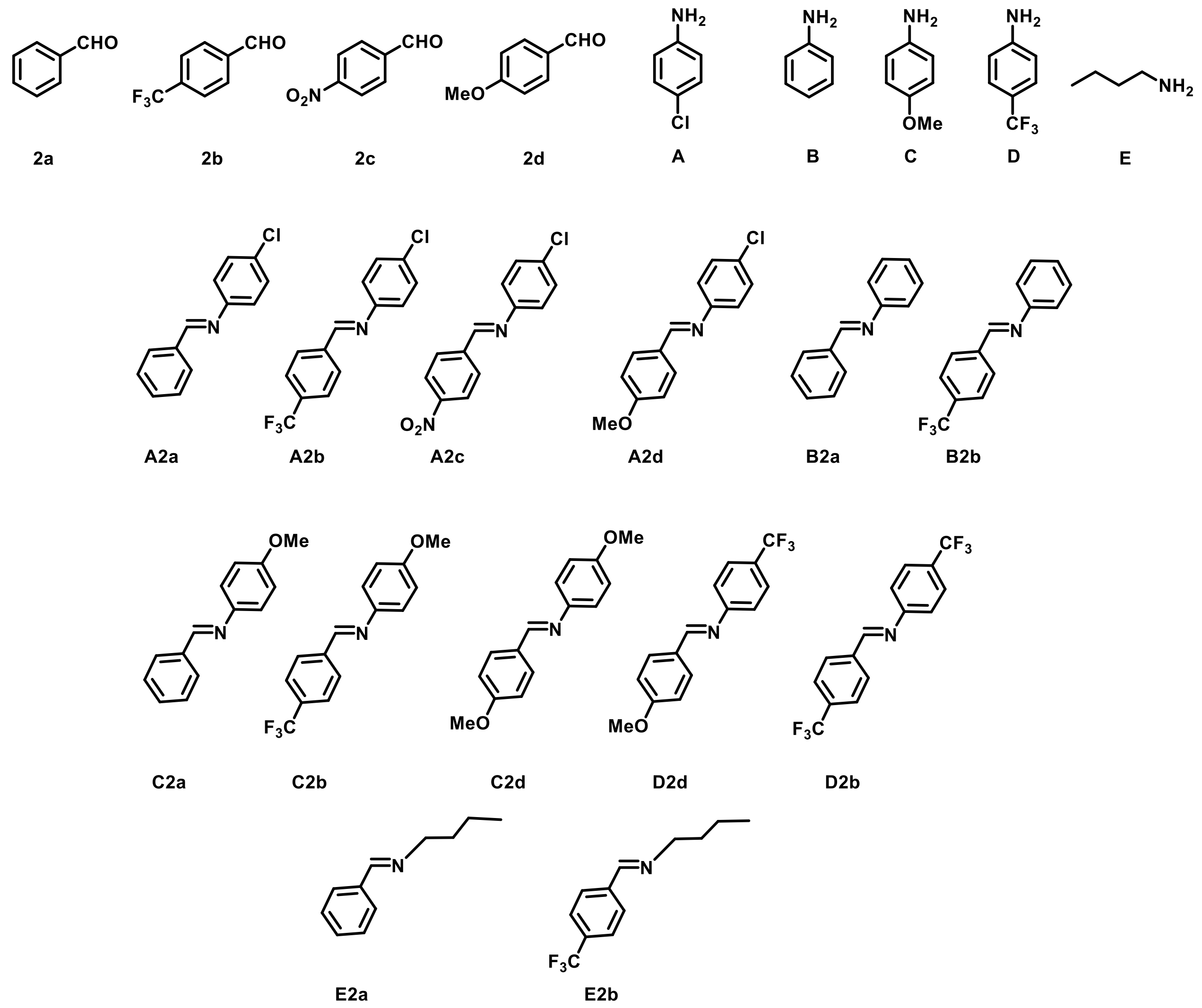

Figure S1. Structures of all aldehydes, anilines and imines used in this study.

4. Characterization of new compounds

N-(4-chlorophenyl)-1-(4-(trifluoromethyl)phenyl)methanimine (A2b)

Obtained as yellow solid (mp 80.1-81.5), following the general procedure described in **2.1** using 4-chloroaniline (**A**) and 4-(trifluoromethyl)benzaldehyde (**2b**) in 95% of yield.

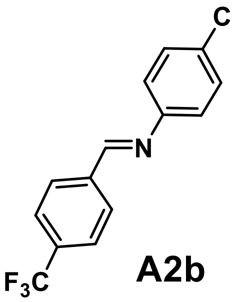

A2b

^{1}H NMR (400 MHz, $CDCl_3$, 298 K): δ 7.17 (d, J = 8.5 Hz, 2H, ArH), 7.38 (d, J = 8.5 Hz, 2H, ArH), 8.01 (d, J = 8.3 Hz, 2H, ArH), 7.73 (d, J = 8.3 Hz, 2H, ArH), 8.48 (s, 1H, CH). ^{13}C NMR (75 MHz, $CDCl_3$, 298 K): δ 122.4, 124.1 (q, $^1J_{CF}$ = 271.7 Hz), 125.8 (q, $^3J_{CF}$ = 3.7 Hz), 129.3, 129.6, 132.4, 133.1 (q, $^2J_{CF}$ = 32.4 Hz), 139.3, 150.1, 159.0. [MALDI-FT-ICR] m/z calcd for $C_{14}H_9ClF_3N$ $[M+H]^+$: 284.04484, found: 284.04479.

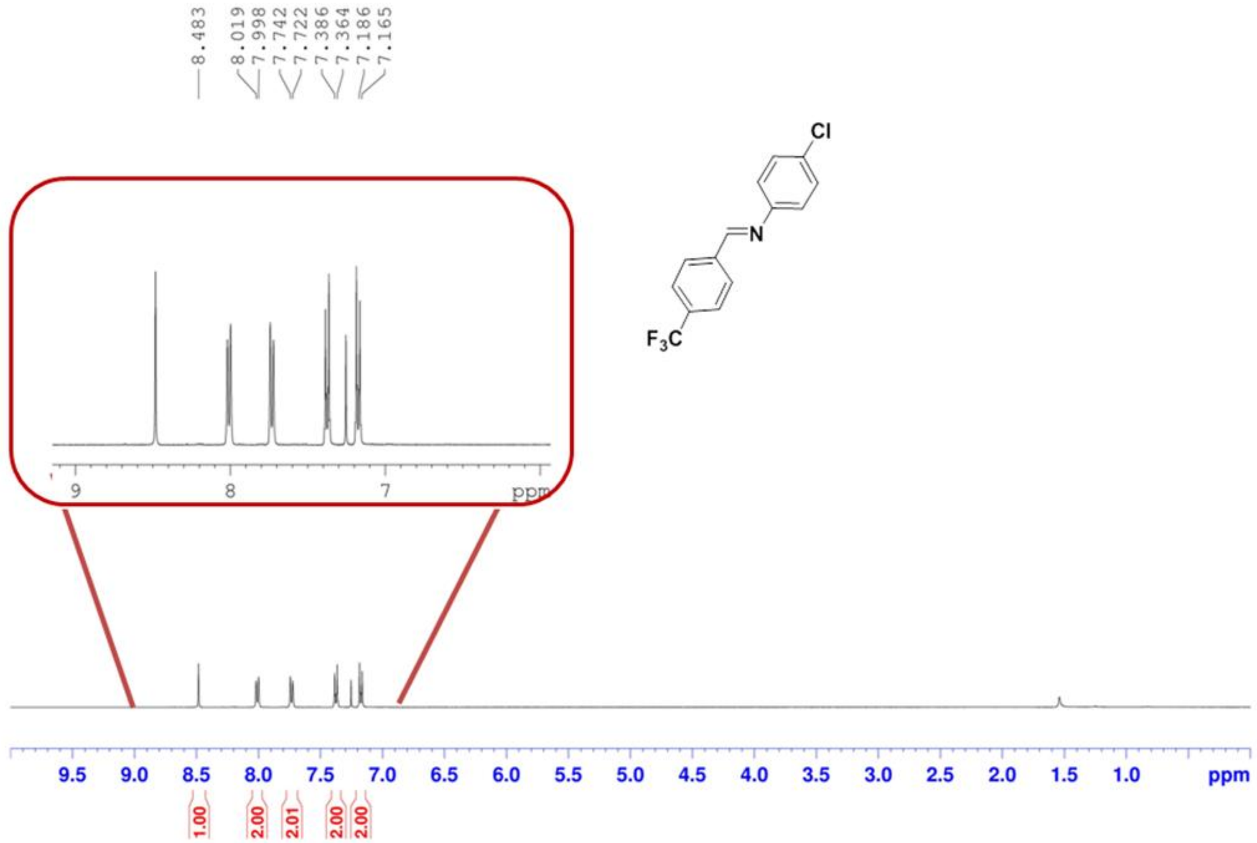

Figure S2. ^{1}H NMR spectrum of **A2b** (400 MHz, $CDCl_3$, 298 K).

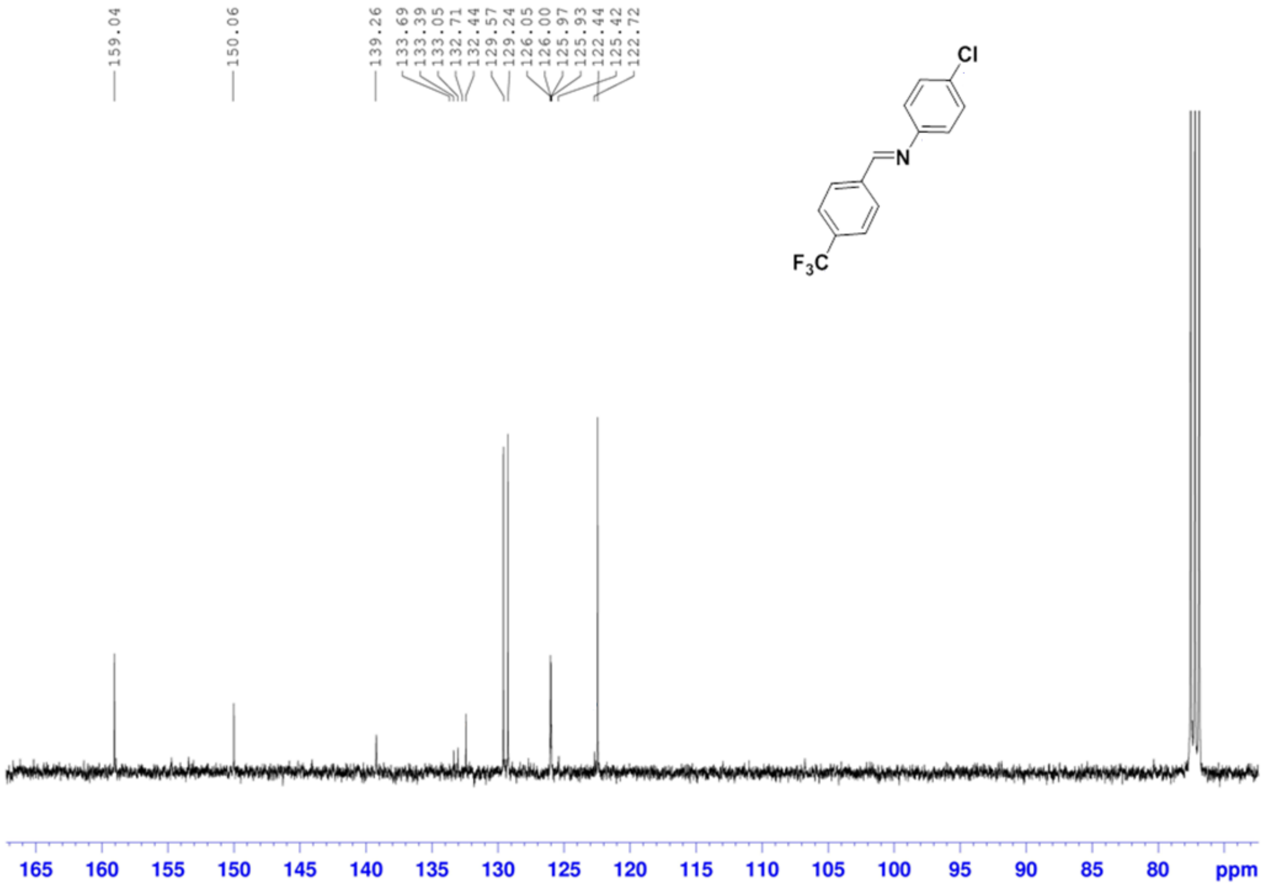

Figure S3. ^{13}C-NMR spectrum of **A2b** (100.57 MHz, $CDCl_3$, 298 K).

1-bis(4-(trifluoromethyl)phenyl)methanimine (D2b)

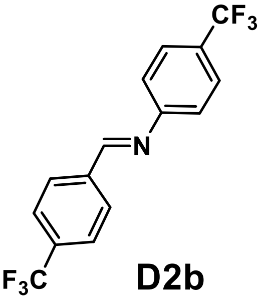

D2b

Obtained as yellow solid (mp 87.5-88.2), following the general procedure described in **2.1** using 4-(trifluoromethyl)aniline (**D**) and 4-(trifluoromethyl)benzaldehyde (**2b**) in 85 % of yield.

^{1}H NMR (400 MHz, $CDCl_3$, 298 K): δ 7.28 (d, J = 8.4, 2H, ArH), 7.67 (d, J = 8.4, 2H, ArH), 7.76 (d, J = 8.3, 2H, ArH), 8.04 (d, J = 8.3, 2H, ArH), 8.48 (s, 1H, CH). ^{13}C NMR (100MHz, $CDCl_3$, 298 K): δ 121.0, 123.8 (q, $^1J_{CF}$ = 270.0 Hz), 124.2 (q, $^1J_{CF}$ = 271.1 Hz), 125.8 (q, $^3J_{CF}$ = 3.5 Hz), 126.4 (q, $^3J_{CF}$ = 3.4 Hz), 128.3 (q, $^2J_{CF}$ = 30.6 Hz), 129.2, 133.29 (q, $^2J_{CF}$ = 34.7 Hz), 138.8, 154.57, 160.3. **[MALDI-FT-ICR]** m/z calcd for $C_{15}H_9F_6N$ $[M+H]^+$: 318.07119, found: 318.07130.

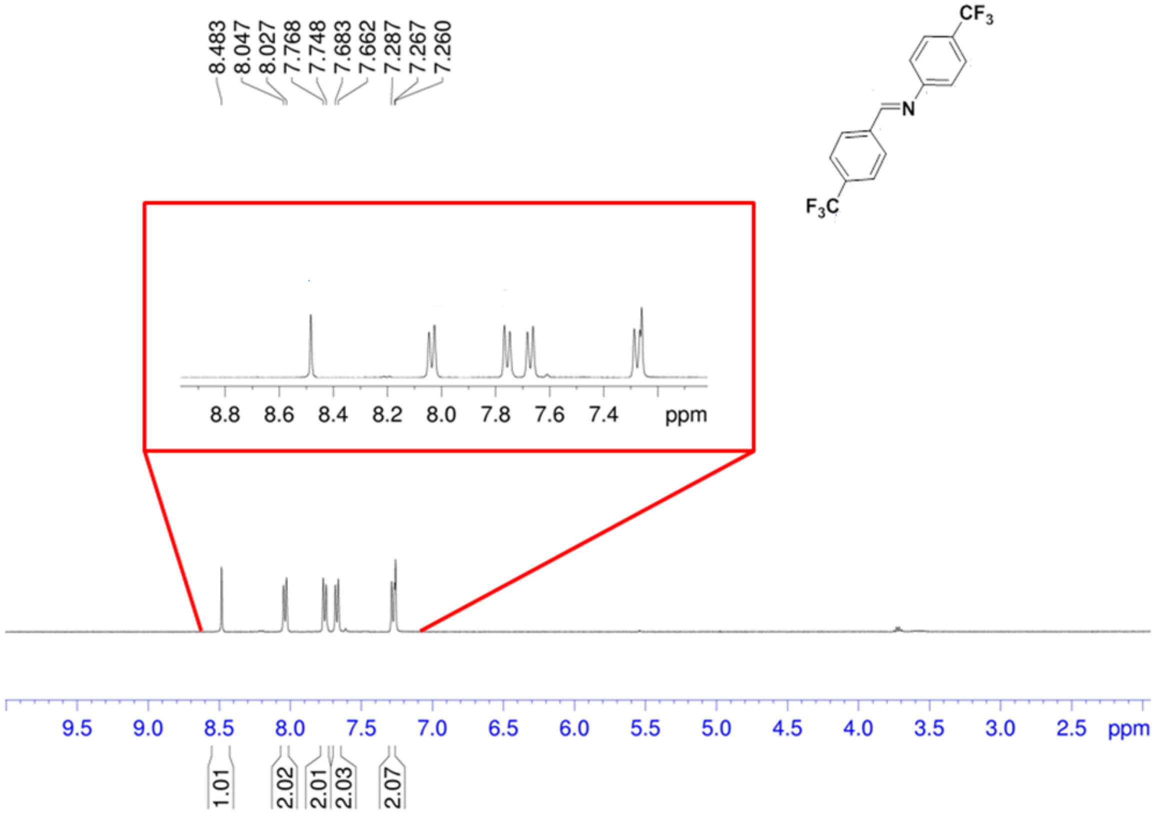

Figure S4. ^{1}H-NMR spectrum of **D2b** (400 MHz, $CDCl_3$, 298 K).

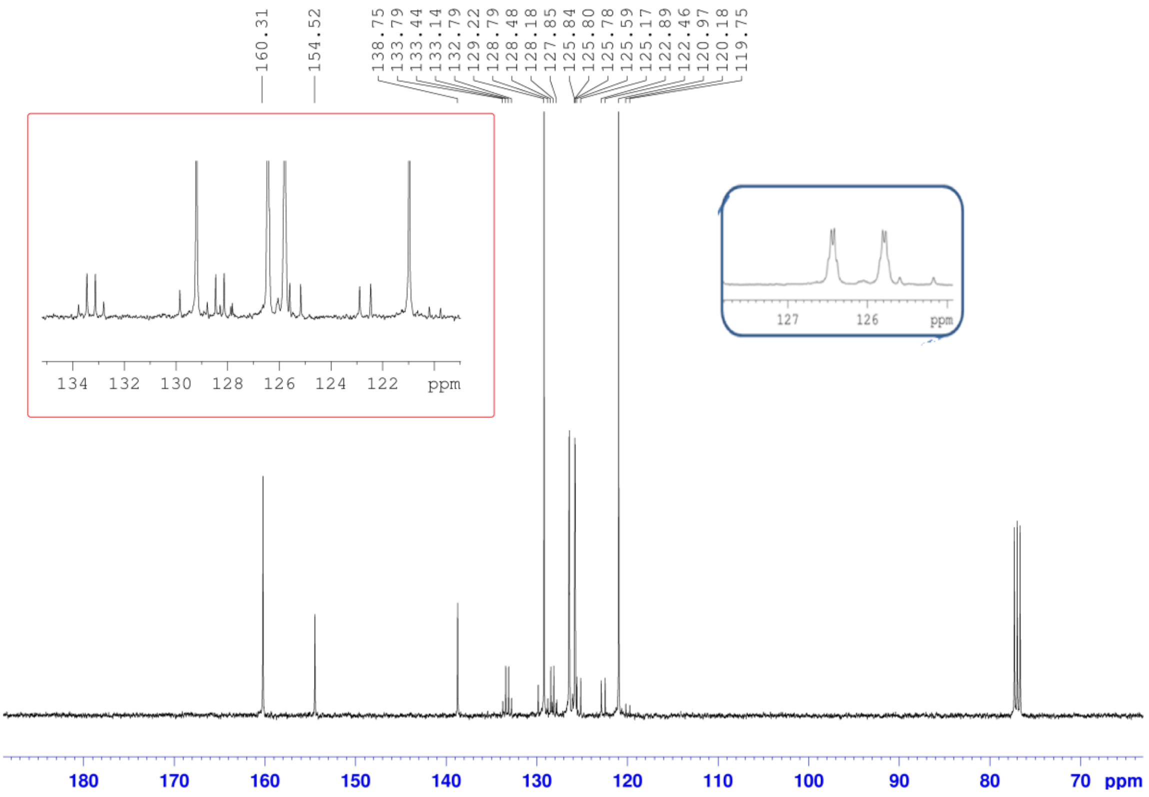

Figure S5. ^{13}C-NMR spectrum of **D2b** (100.57 MHz, $CDCl_3$, 298 K).

1-(4-methoxyphenyl)-N-(4-(trifluoromethyl)phenyl)methanimine (D2d)

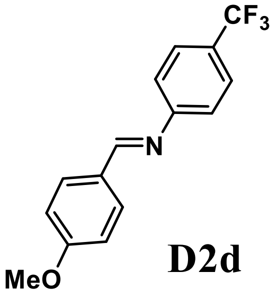

D2d

Obtained as yellow solid (mp 84.5-85.5) following the general procedure described in **2.1** using 4-(trifluoromethyl)aniline (**D**) and 4-methoxybenzaldehyde (**2d**) in 92 % of yield.

^{1}H NMR (300 MHz, $CDCl_3$, 298 K): δ 3.89 (s, 3H, OCH_3), 7.01 (d, J =8.4, 2H, ArH), 7.24 (d, J =8.1, 2H, ArH), 7.63 (d, J =8.1, 2H, ArH), 7.86 (d, J =8.4, 2H, ArH), 8.35 (s, 1H, CH). ^{13}C NMR (100MHz, $CDCl_3$, 298 K): δ 55.6, 114.2, 121.0, 124.6 (q, $^1J_{CF}$ = 271.8), 126.5 (q, $^3J_{CF}$ = 3.9 Hz), 127.4 (q, $^2J_{CF}$ = 32.3 Hz), 128.9, 131.0, 155.4, 161.1, 162.6. Elemental analysis calculated (%) for $C_{15}H_{12}F_3NO$: C, 64.51; H, 4.33; N, 5.02; found: C, 64.45; H, 4.28; N, 5.10.

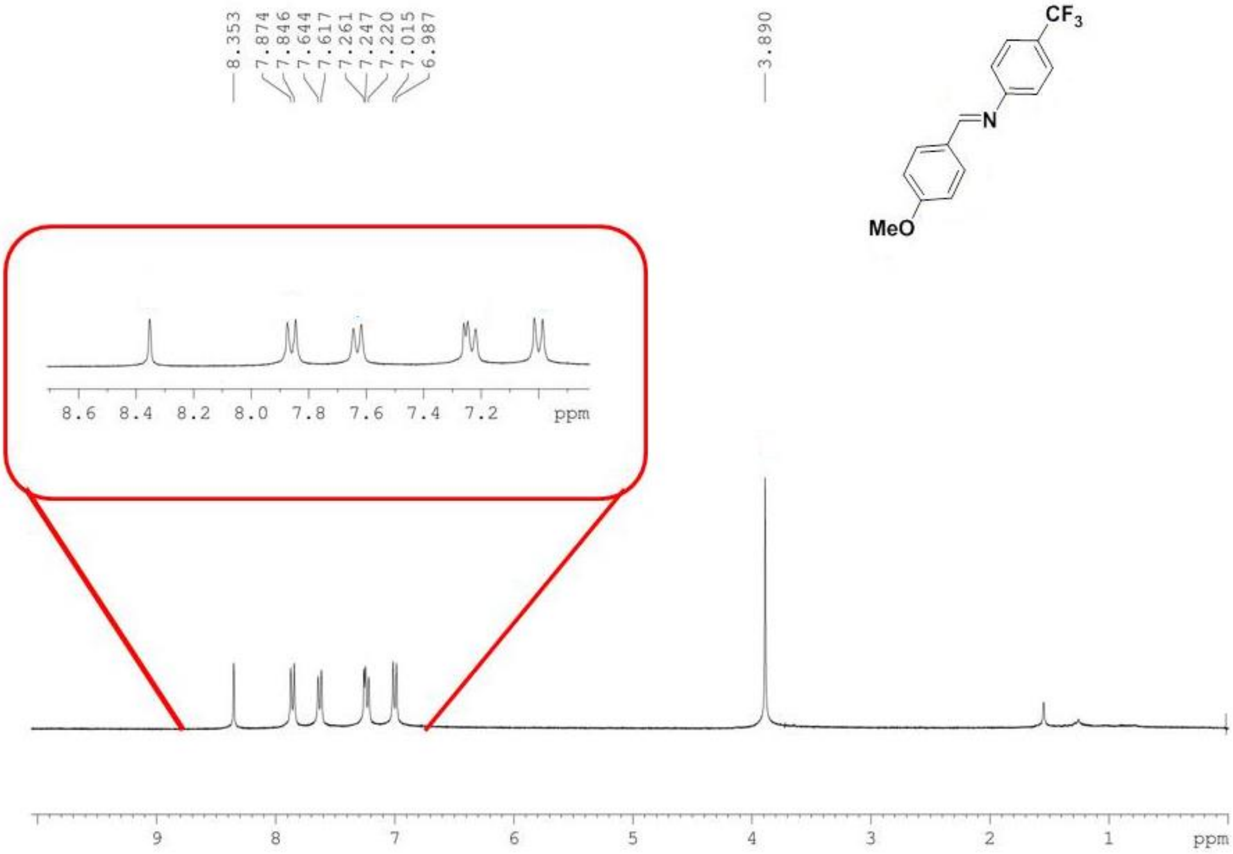

Figure S6. ^{1}H-NMR spectrum of **D2d** (300 MHz, $CDCl_3$, 298 K).

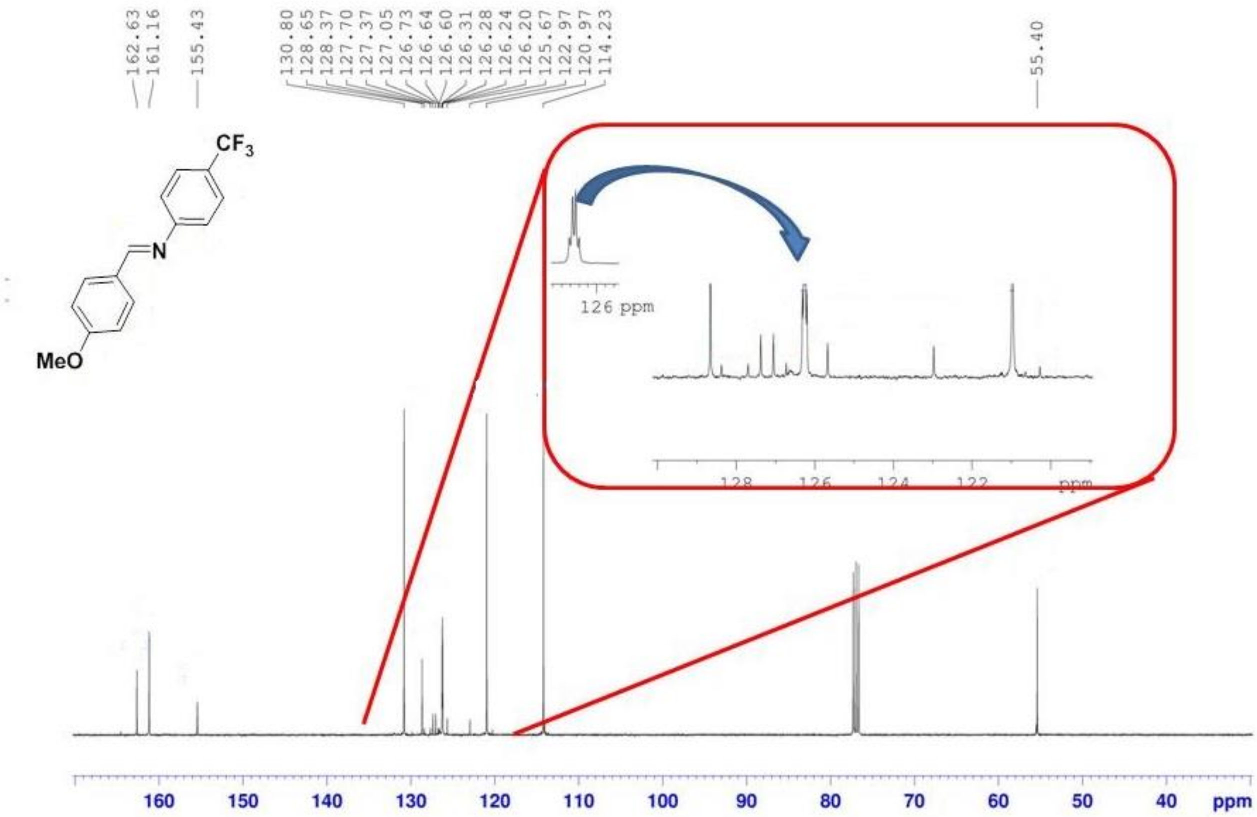

Figure S7. ^{13}C-NMR spectrum of **D2d** (100.57 MHz, $CDCl_3$, 298 K).

5. Two – component reactions (Figure 3 in the main text)

5.1 Formation of A2a by aldehyde 2a and aniline A

*5.1.1 Formation of **A2a** by aldehyde **2a** and aniline **A** in absence of capsule **CR**$_6$* (Figure 3 top)**.**

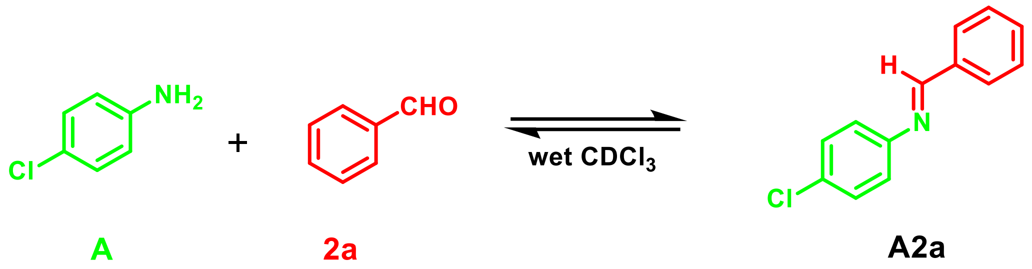

Scheme S1. Synthesis of **A2a** in absence of capsule **CR$_6$**.

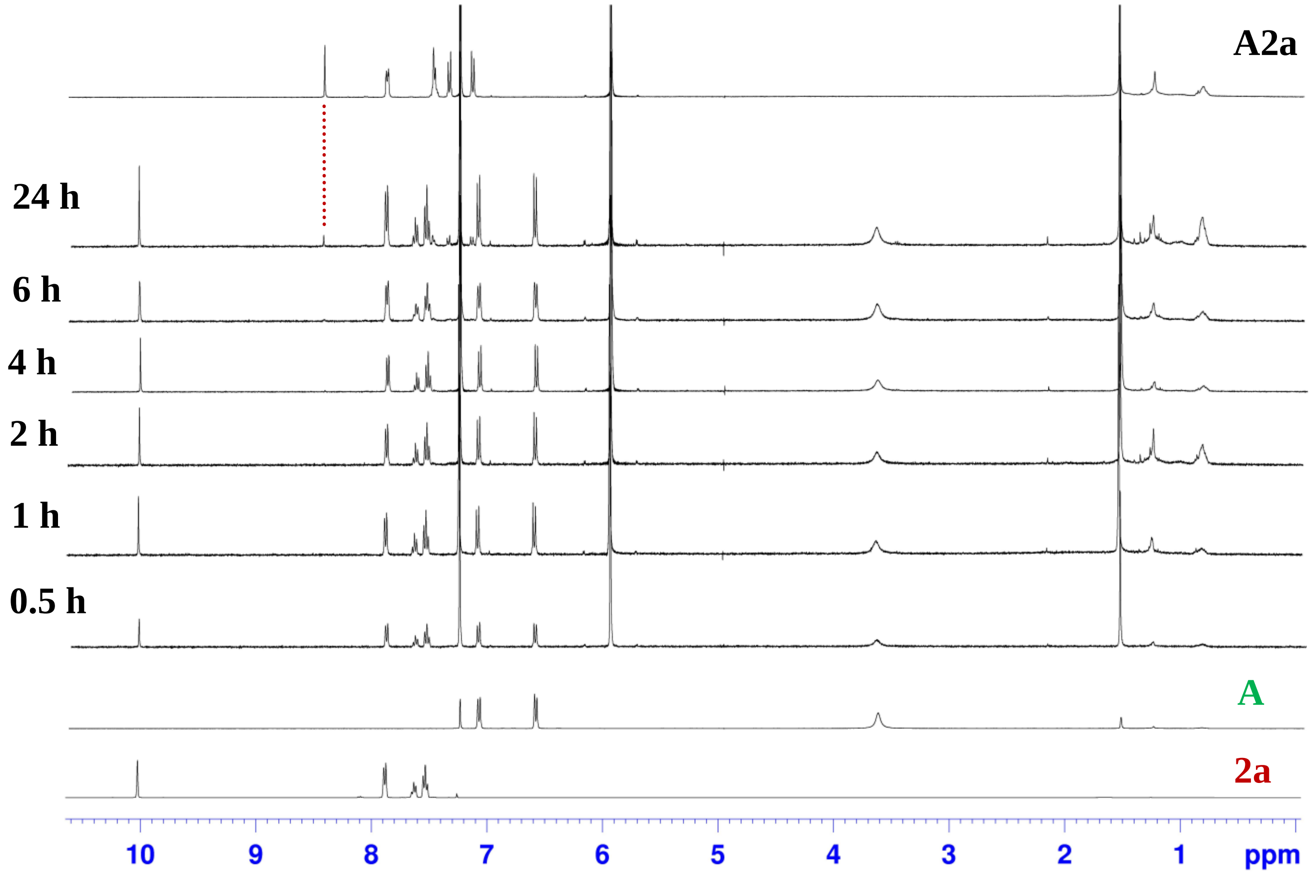

Figure S8. ^{1}H NMR (400 MHz, $CDCl_3$, 298 K) spectra of an equimolar mixture of **A** and **2a** (42.3 mM each, water saturated $CDCl_3$, r.t.) after 0.5, 1, 2, 4, 6 and 24 h. (Bottom), ^{1}H NMR spectra of **A** and **2a**. (Top) ^{1}H NMR spectrum of the isolated imine **A2a**.

*5.1.2 Formation of imine **A2a** by aldehyde **2a** and aniline **A** in presence of capsule **CR_6*** (Figure 3 top)**.**

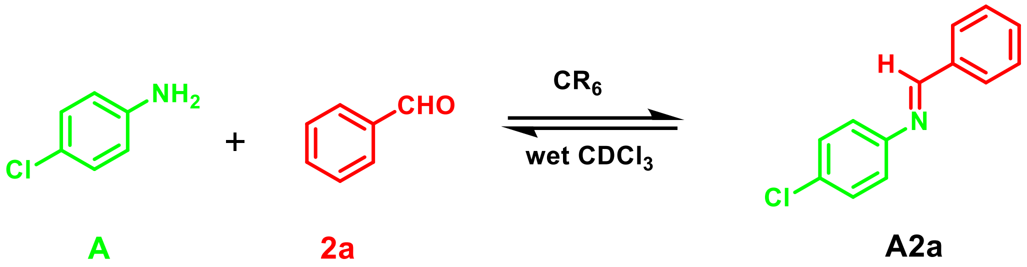

Scheme S2. Synthesis of **A2a** in presence of capsule **CR_6**.

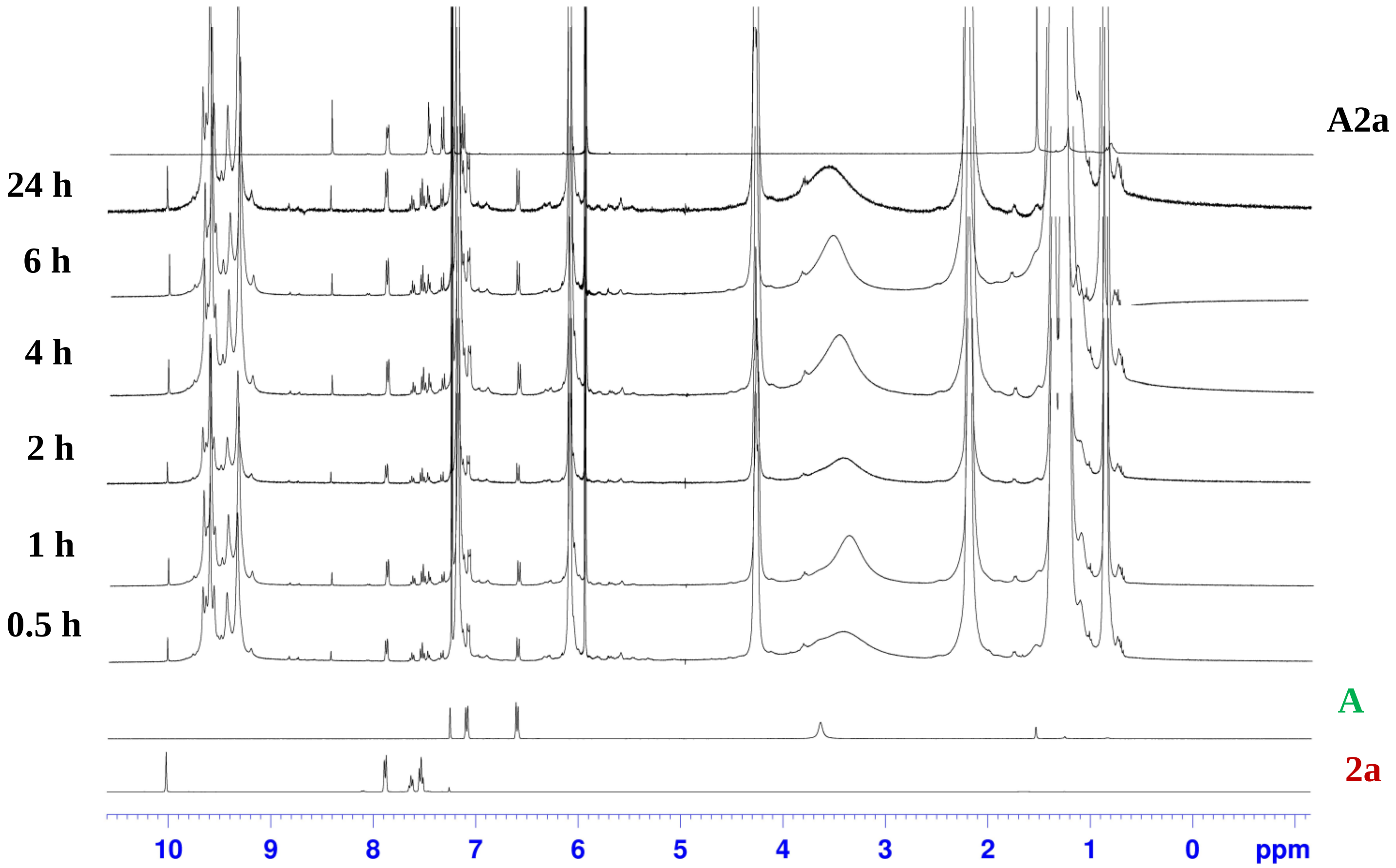

Figure S9. ^{1}H NMR (400 MHz, $CDCl_3$, 298 K) spectra of an equimolar mixture of **A, 2a** and **CR_6** (42.3 mM each, water-saturated $CDCl_3$, r.t.) after 0.5, 1, 2, 4, 6 and 24 h. (Bottom), ^{1}H NMR spectra of **A** and **2a**. (Top) ^{1}H NMR spectrum of the isolated imine **A2a**.

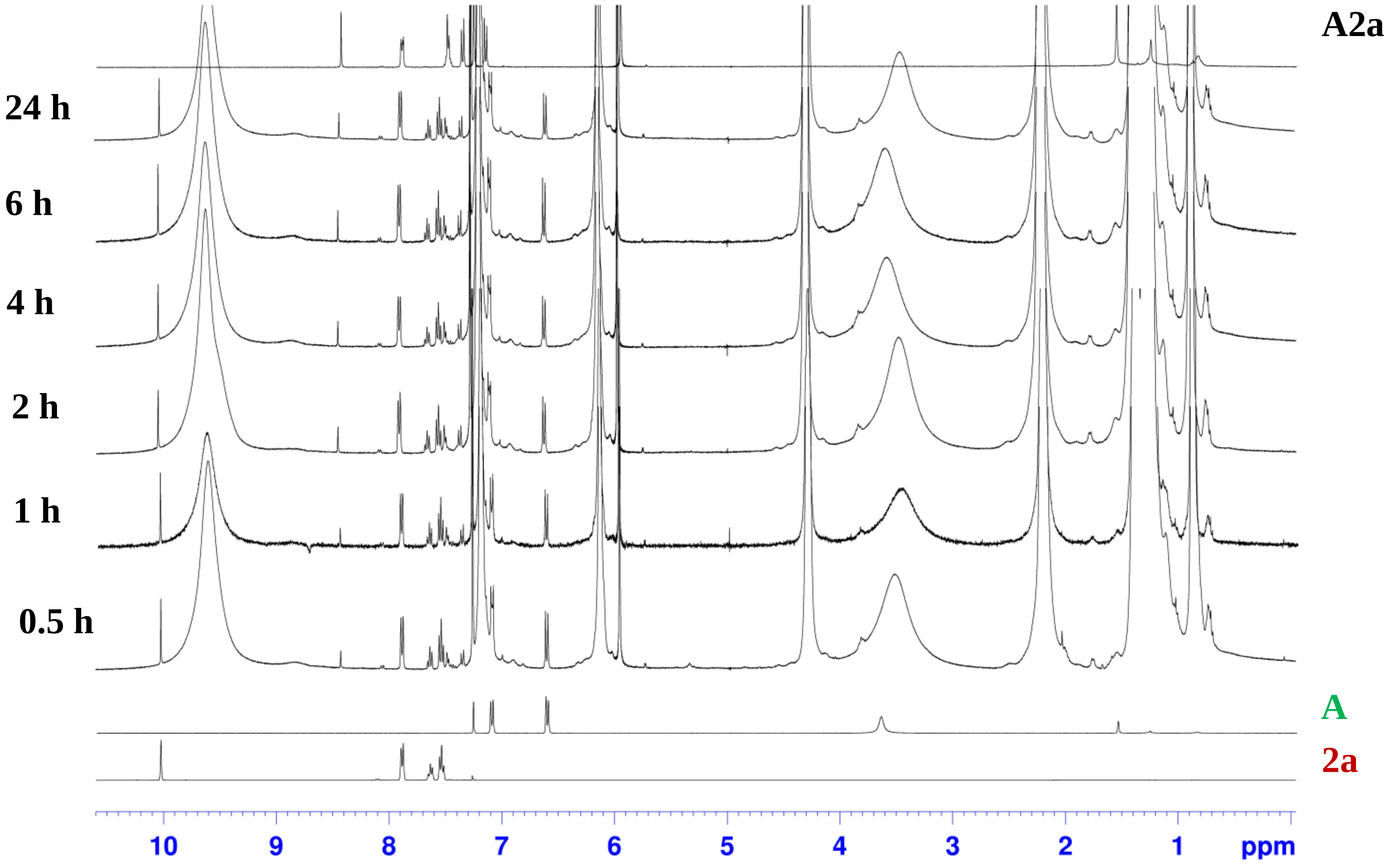

Figure S10. ^{1}H NMR (400 MHz, $CDCl_3$, 298 K) spectra of an equimolar mixture of **A, 2a** and **CR**$_6$ (42.3 mM each, water-saturated $CDCl_3$, r.t.) after 0.5, 1, 2, 4, 6 and 24 h. The spectra are recorded after addition of DMSO (2 μL) to the reaction aliquot, in order to disaggregate the capsule. (Bottom), ^{1}H NMR spectra of **A** and **2a**. (Top) ^{1}H NMR spectrum of the isolated imine **A2a**.

Table S1. Time-dependent conversion in **A2a** of an equimolar mixture of **A** and **2a** in presence and in absence of **CR_6** (section 5.1.1 and 5.2.2, Figure 3).[a]

**Time[b] (h)**	**A2a (%)[c] in absence of CR_6**	**A2a (%)[c,d] in presence of CR_6**
**0.5**	-[e]	24
**1**	-[e]	26
**2**	-[e]	34
**4**	-[e]	34
**6**	-[e]	34
**24**	12	34
**48**	12	34

[a] Reaction conditions: **2a** (0.0423 mmol, 42.3 mM), **A** (0.0423 mmol, 42.3 mM), water-saturated $CDCl_3$ (1 mL), rt. [b] Time at which an aliquot (30 μL) of the reaction mixture was taken and monitored via ^{1}H-NMR spectrum. [c] Conversion was calculated using TCE as internal standard. [d] Conversion was calculated after addition of DMSO (2 μL) to a reaction aliquot, in order to disaggregate the capsule. [e]Below the limit of detection. Error in ^{1}H-NMR signal integration was ± 5%.

5.2 Formation of A2b by aldehyde 2b and aniline A.

*5.2.1 Formation of **A2b** by aldehyde **2b** and aniline **A** in absence of capsule **CR_6*** (Figure 3 bottom).

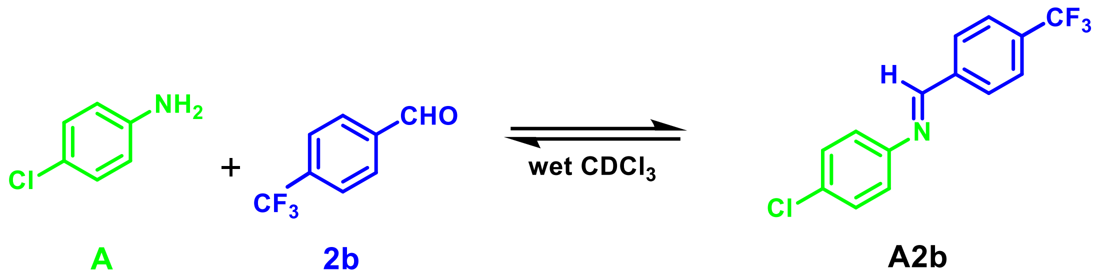

Scheme S3. Synthesis of **A2b** in absence of capsule **CR_6**.

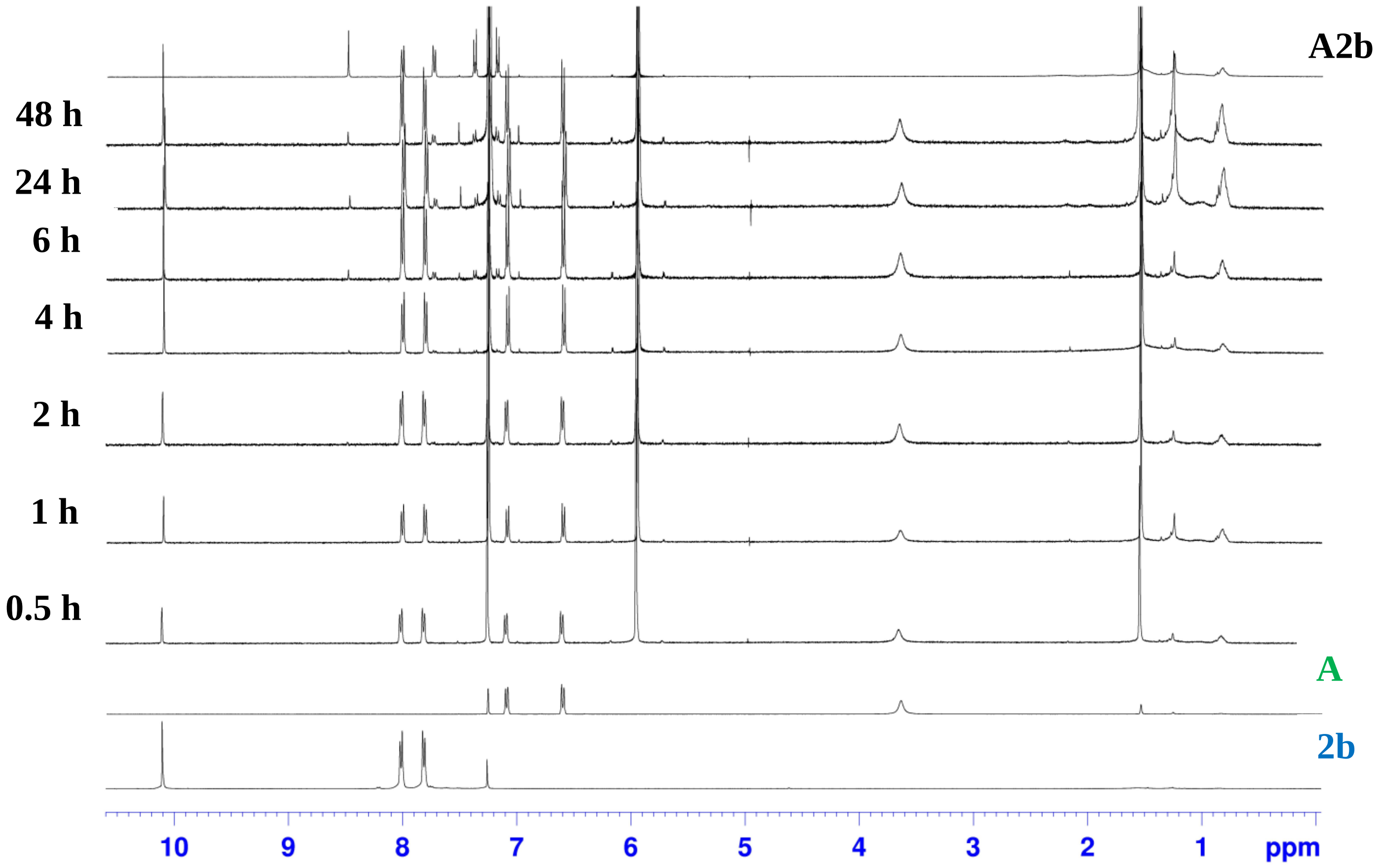

Figure S11. ^{1}H NMR (400 MHz, $CDCl_3$, 298 K) spectra of an equimolar mixture of **A** and **2b** (42.3 mM each, water saturated $CDCl_3$, r.t.) after 0.5, 1, 2, 4, 6 and 24 h. (Bottom), ^{1}H NMR spectra of **A** and **2b**. (Top) ^{1}H NMR spectrum of the isolated imine **A2b**.

*5.2.2 Formation of **A2b** by aldehyde **2b** and aniline **A** in presence of capsule **CR$_6$*** (Figure 3 bottom).

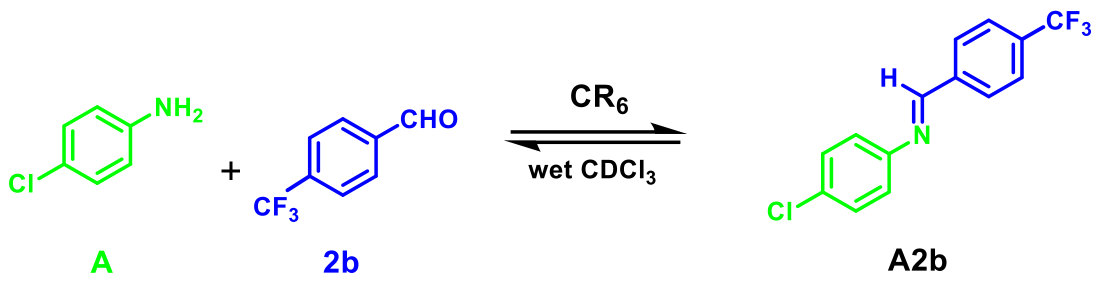

Scheme S4. Synthesis of **A2b** in presence of capsule **CR$_6$**.

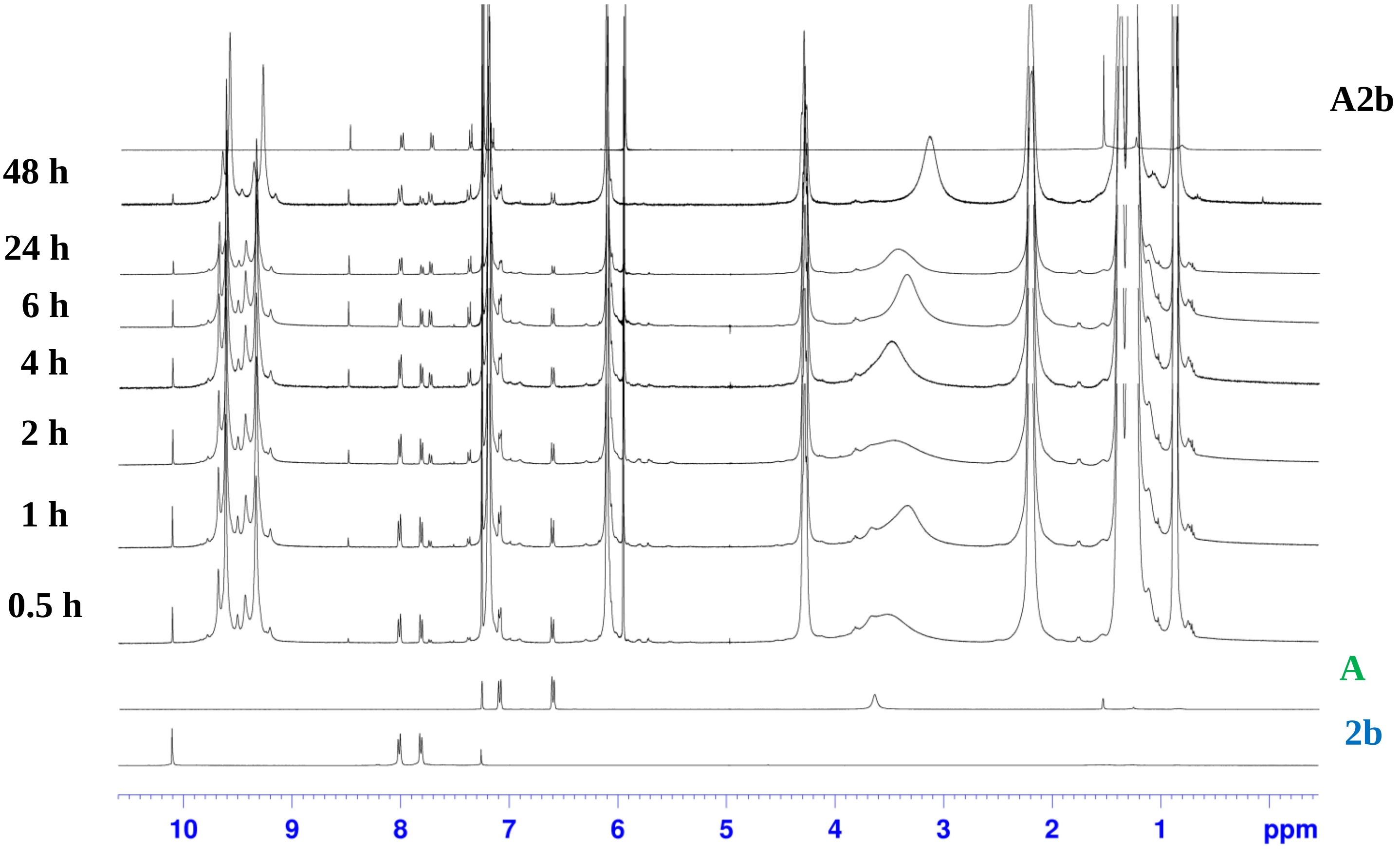

Figure S12. ^{1}H NMR (400 MHz, $CDCl_3$, 298 K) spectra of an equimolar mixture of **A, 2b** and **CR$_6$** (42.3 mM each, water-saturated $CDCl_3$, r.t.) after 0.5, 1, 2, 4, 6 and 24 h. (Bottom), ^{1}H NMR spectra of **A** and **2b**. (Top) ^{1}H NMR spectrum of the isolated imine **A2b**.

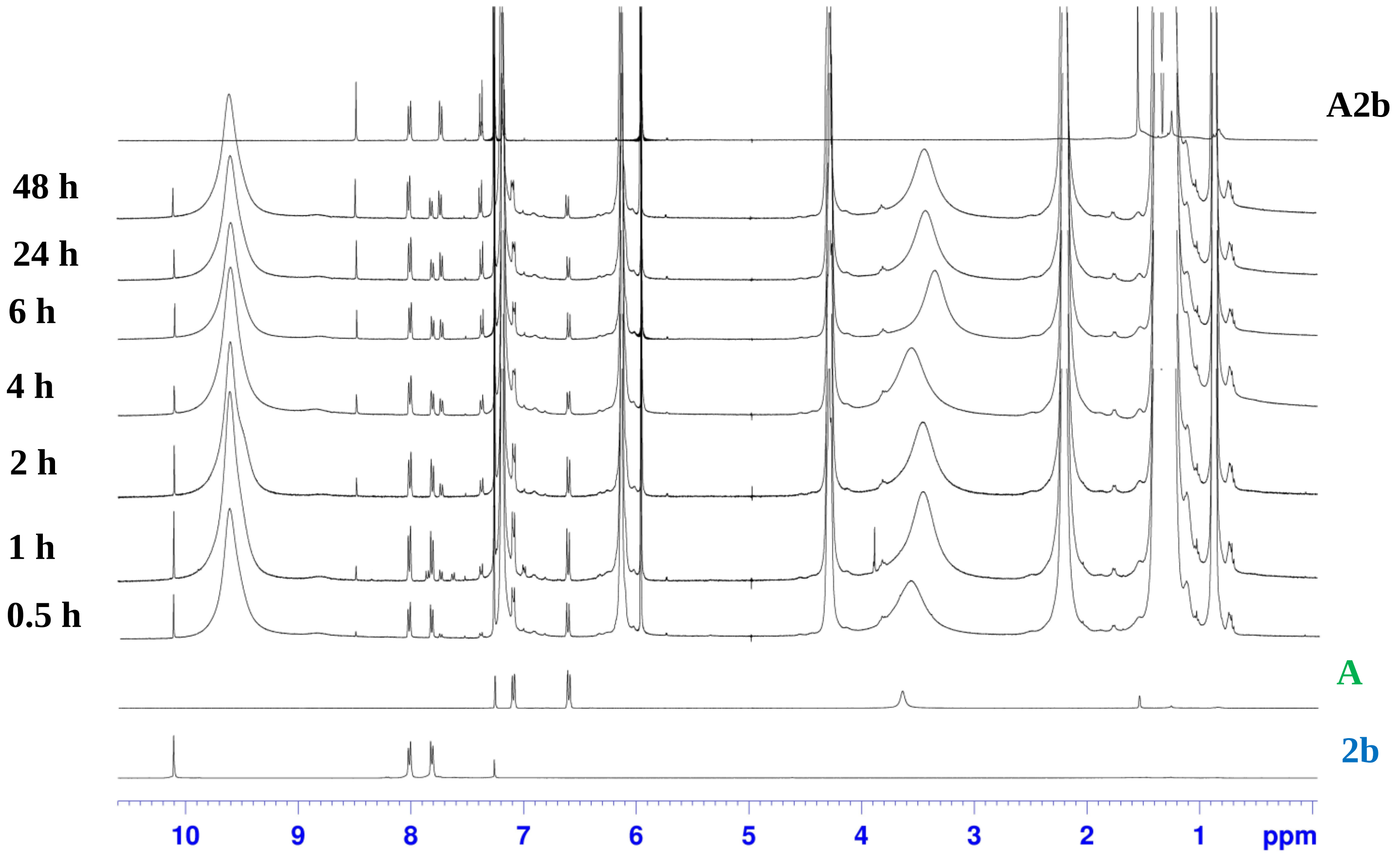

Figure S13. ^{1}H NMR (400 MHz, $CDCl_3$, 298 K) spectra of an equimolar mixture of **A, 2b** and **CR_6** (42.3 mM each, water-saturated $CDCl_3$, r.t.) after 0.5, 1, 2, 4, 6 and 24 h. The spectra are recorded after addition of DMSO (2 μL) to the reaction aliquot, in order to disaggregate the capsule. (Bottom), ^{1}H NMR spectra of **A** and **2b**. (Top) ^{1}H NMR spectrum of the isolated imine **A2a**.

Table S2. Time-dependent conversion in **A2b** of an equimolar mixture of **A** and **2b** in presence and in absence of **CR_6** (section 5.2.1 and 5.2.2, Figure 3).[a]

	**in absence of CR_6**	**in presence of CR_6**
**Time[b] (h)**	**A2b (%)[c]**	**A2b (%)[c,d]**
**0.5**	-[e]	10
**1**	-[e]	20
**2**	-[e]	27
**4**	5	39
**6**	11	47
**24**	11	60
**48**	11	60

[a] Reaction conditions: **2b** (0.0423 mmol, 42.3 mM), **A** (0.0423 mmol, 42.3 mM), water-saturated $CDCl_3$ (1 mL), rt. [b] Time at which an aliquot (30 μL) of the reaction mixture was taken and monitored via ^{1}H-NMR spectrum. [c] Conversion was calculated using TCE as internal standard. [d] Conversion was calculated after addition of DMSO (2 μL) to a reaction aliquot, in order to disaggregate the capsule. [e]Below the limit of detection. Error in ^{1}H-NMR signal integration was ± 5%.

6. Evolution of dynamic imine libraries as a function of time

6.1 Competitive reaction between equivalent amounts of 2a, 2b and A without capsule CR6

(Figure 4a in the main text).

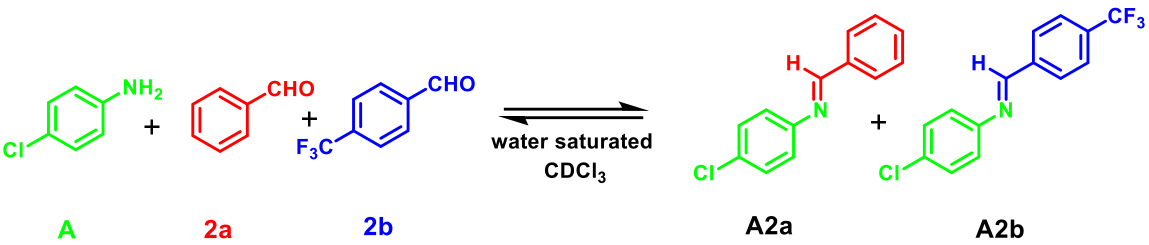

Scheme S5. Synthesis of **A2a** and **A2b** in absence of capsule **CR6**.

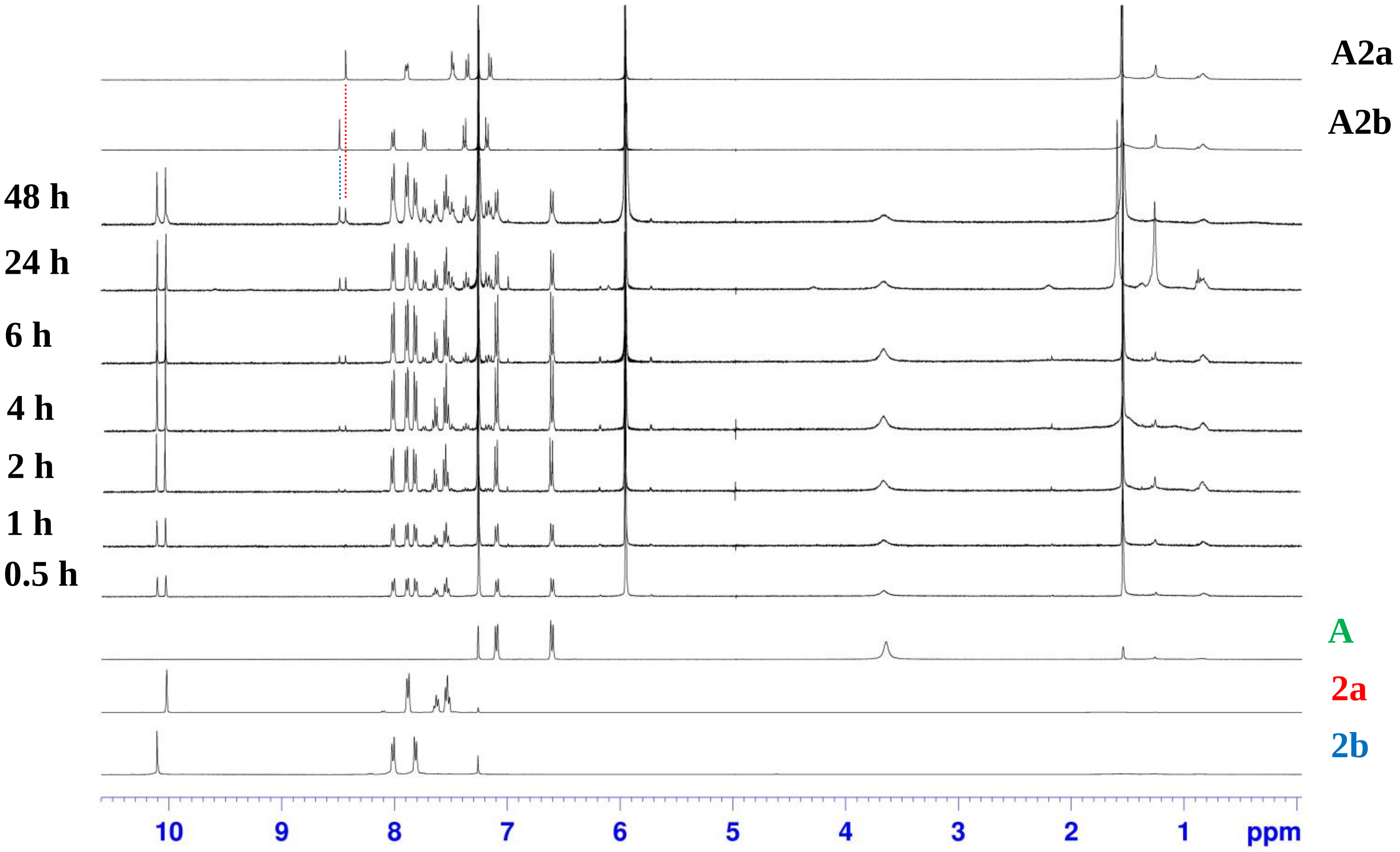

Figure S14. ^{1}H NMR (400 MHz, $CDCl_3$, 298 K) spectra of an equimolar mixture of **A**, **2a** and **2b** (42.3 mM each, water saturated $CDCl_3$, r.t.) after 0.5, 1, 2, 4, 6, 24 and 48 h. (Bottom), ^{1}H NMR spectra of **A, 2a** and **2b**. (Top) ^{1}H NMR spectrum of the isolated imines **A2a** and **A2b**.

6.2 Competitive reaction between equivalent amounts of 2a, 2b and A in presence of capsule CR6 (Figure 4b in the main text).

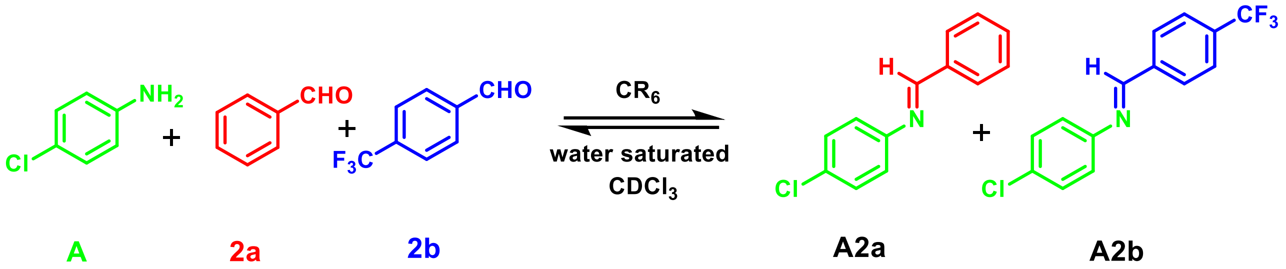

Scheme S6. Synthesis of **A2a** and **A2b** in presence of capsule **CR6**.

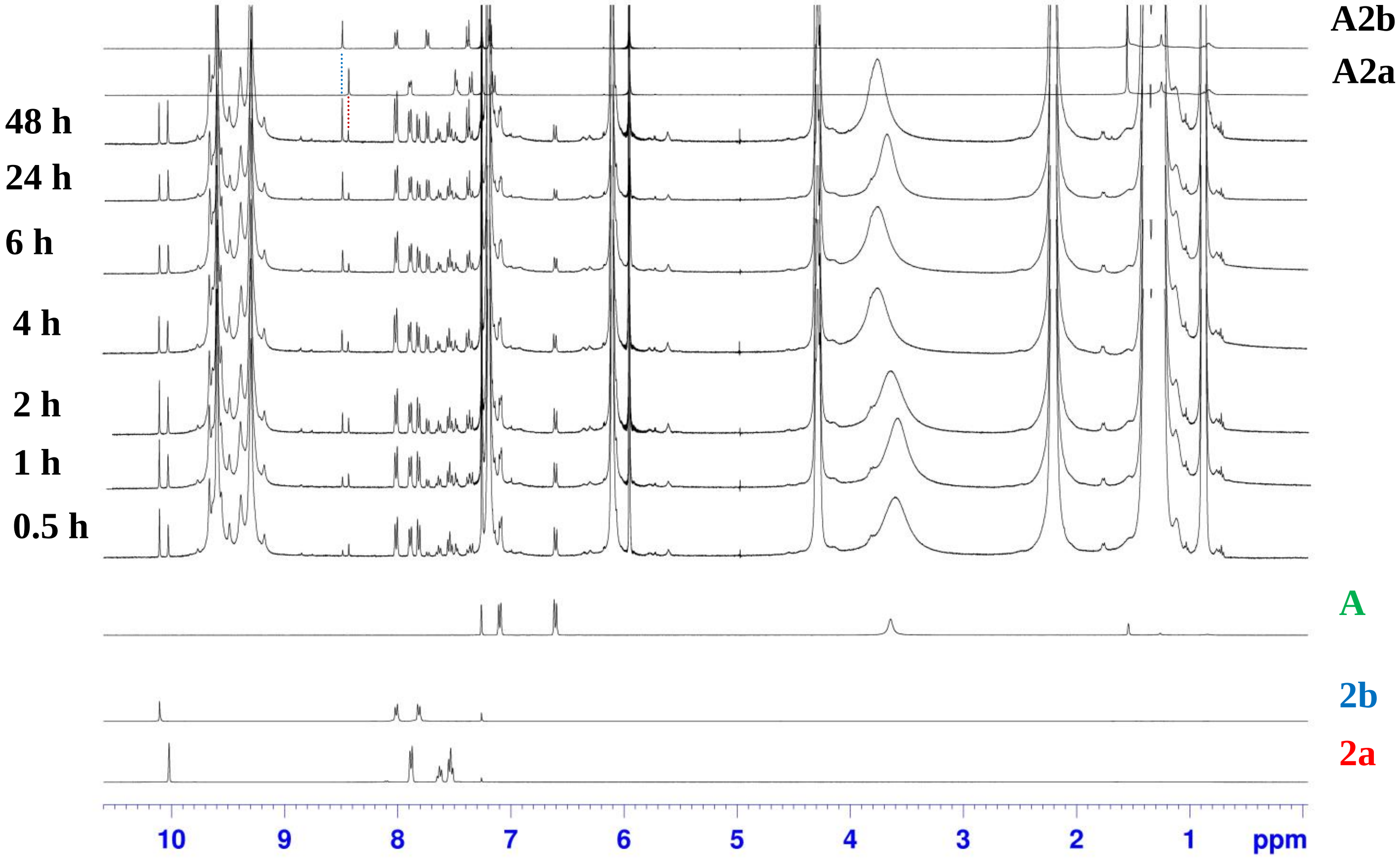

Figure S15. ^{1}H NMR (400 MHz, $CDCl_3$, 298 K) spectra of an equimolar mixture of **A, 2a, 2b** and **CR6** (42.3 mM each, water- saturated $CDCl_3$, r.t.) after 0.5, 1, 2, 4, 6 and 24 h. (Bottom), ^{1}H NMR spectra of **A, 2a** and **2b**. (Top) ^{1}H NMR spectrum of the isolated imines **A2a** and **A2b**.

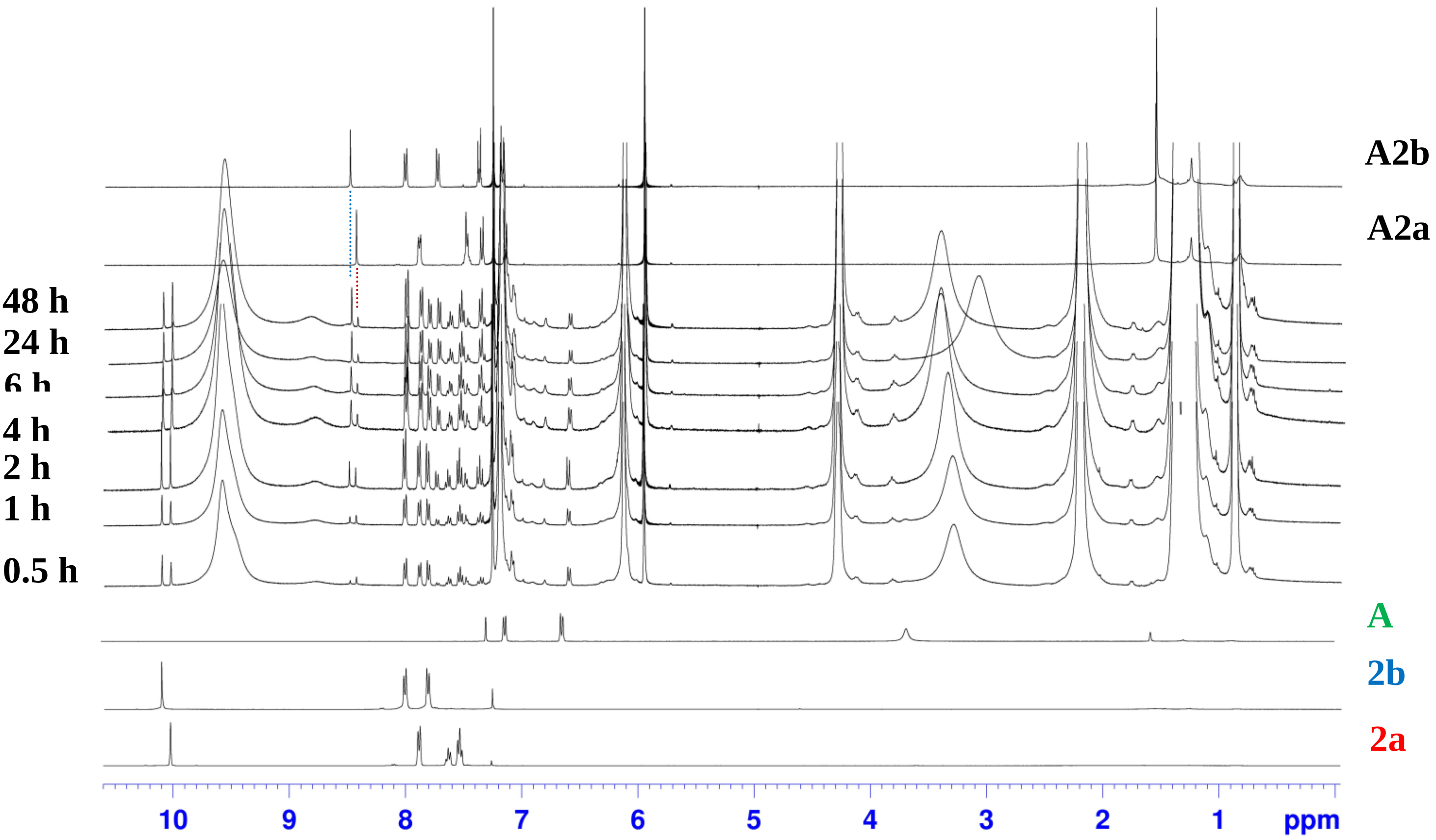

Figure S16. ^{1}H NMR (400 MHz, $CDCl_3$, 298 K) ^{1}H NMR (400 MHz, $CDCl_3$, 298 K) spectra of an equimolar mixture of **A, 2a, 2b** and **CR**$_6$ (42.3 mM each, water- saturated $CDCl_3$, r.t.) after 0.5, 1, 2, 4, 6 and 24 h. The spectra are recorded after addition of DMSO (2 µL) to a reaction aliquot, in order to disaggregate the capsule. (Bottom), ^{1}H NMR spectra of **A, 2a** and **2b**. (Top) ^{1}H NMR spectrum of the isolated imines **A2a** and **A2b**.

Table S3. Time-dependent conversion in **A2a** and **A2b** of an equimolar mixture of **A, 2a** and **2b** in presence and in absence of **CR_6** (section 6.1 and 6.2, Figure 4 in the main text).[a]

	**in absence of CR_6**		**in presence of CR_6**	
**Time[b] (h)**	**A2a (%)[c]**	**A2b (%)[c]**	**A2a (%)[c,d]**	**A2b (%)[c,d]**
**0.5**	-[e]	-[e]	30	10
**1**	-[e]	-[e]	21	19
**2**	7	7	15	23
**4**	7	7	18	40
**6**	9	9	16	46
**24**	21	21	15	60
**48**	21	21	15	60

[a] Reaction conditions: **2a**, **2b** (0.0423 mmol, 42.3 mM), **A** (0.0423 mmol, 42.3 mM), capsule **CR_6** (42.3 mM), water-saturated $CDCl_3$ (1 mL), rt. [b] Time at which an aliquot (30 μL) of the reaction mixture was taken and monitored via ^{1}H-NMR spectrum. [c] Conversion was calculated using TCE as internal standard. [d] Conversion was calculated after addition of DMSO (2 μL) to a reaction aliquot, in order to disaggregate the capsule. [e]Below the limit of detection. Error in ^{1}H-NMR signal integration was ± 5 %.

6.3 Evidence of the capsule effect on the imine distribution in DCL 2a, 2b and A

*6.3.1 Competitive reaction between equivalent amounts of **2a**, **2b** and **A**, in presence of 0.5 and 0.1 equiv of capsule **CR$_6$*** (Figure 9 in the main text).

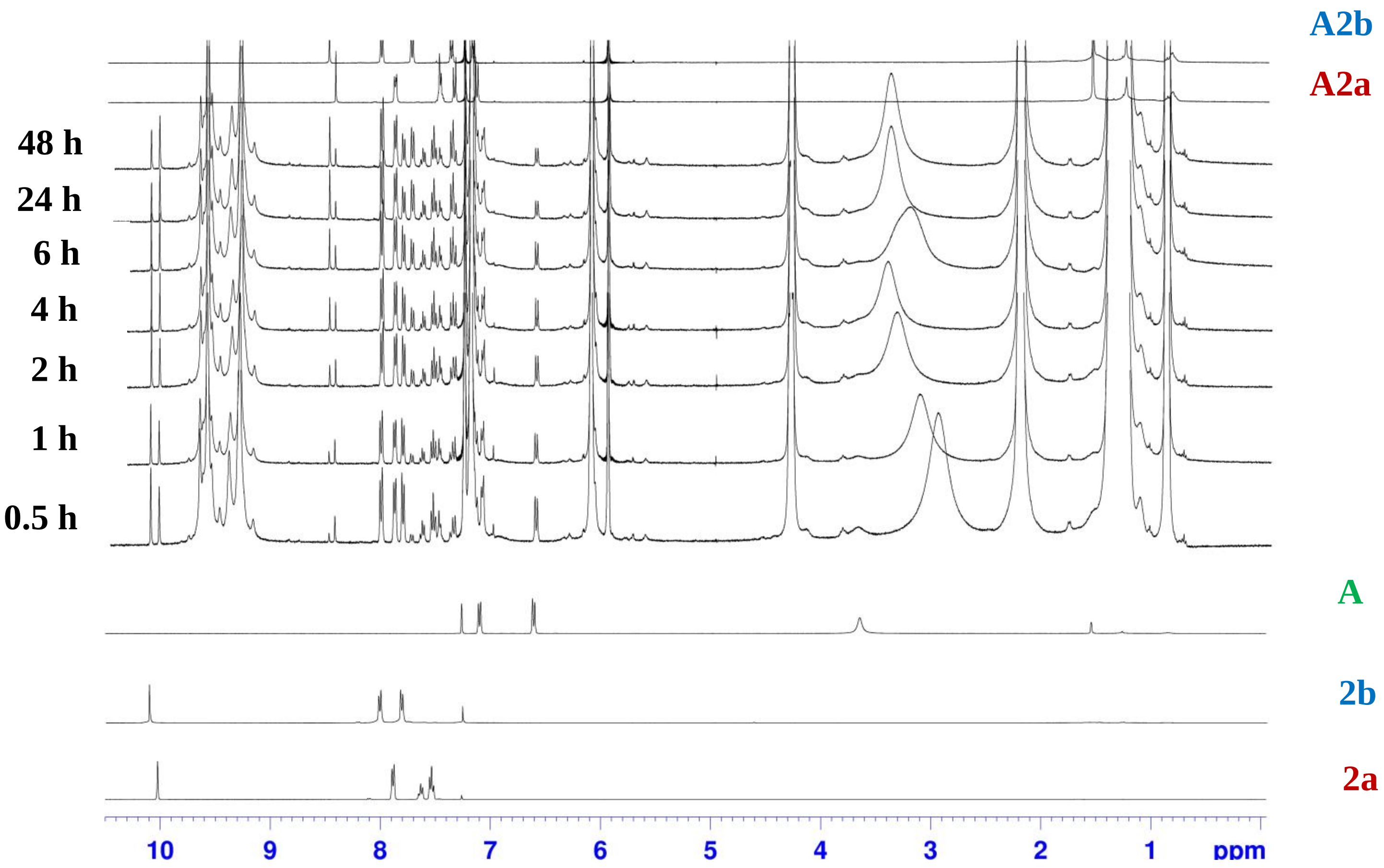

Figure S17. ^{1}H NMR (400 MHz, $CDCl_3$, 298 K) spectra of an equimolar mixture of **A, 2a, 2b** (0.0423 mmol each, water saturated $CDCl_3$, r.t.) and **CR$_6$** (0.0211 mmol) after 0.5, 1, 2, 4, 6 and 24 h. (Bottom), ^{1}H NMR spectra of **A, 2a** and **2b**. (Top) ^{1}H NMR spectrum of the isolated imines **A2a** and **A2b.**

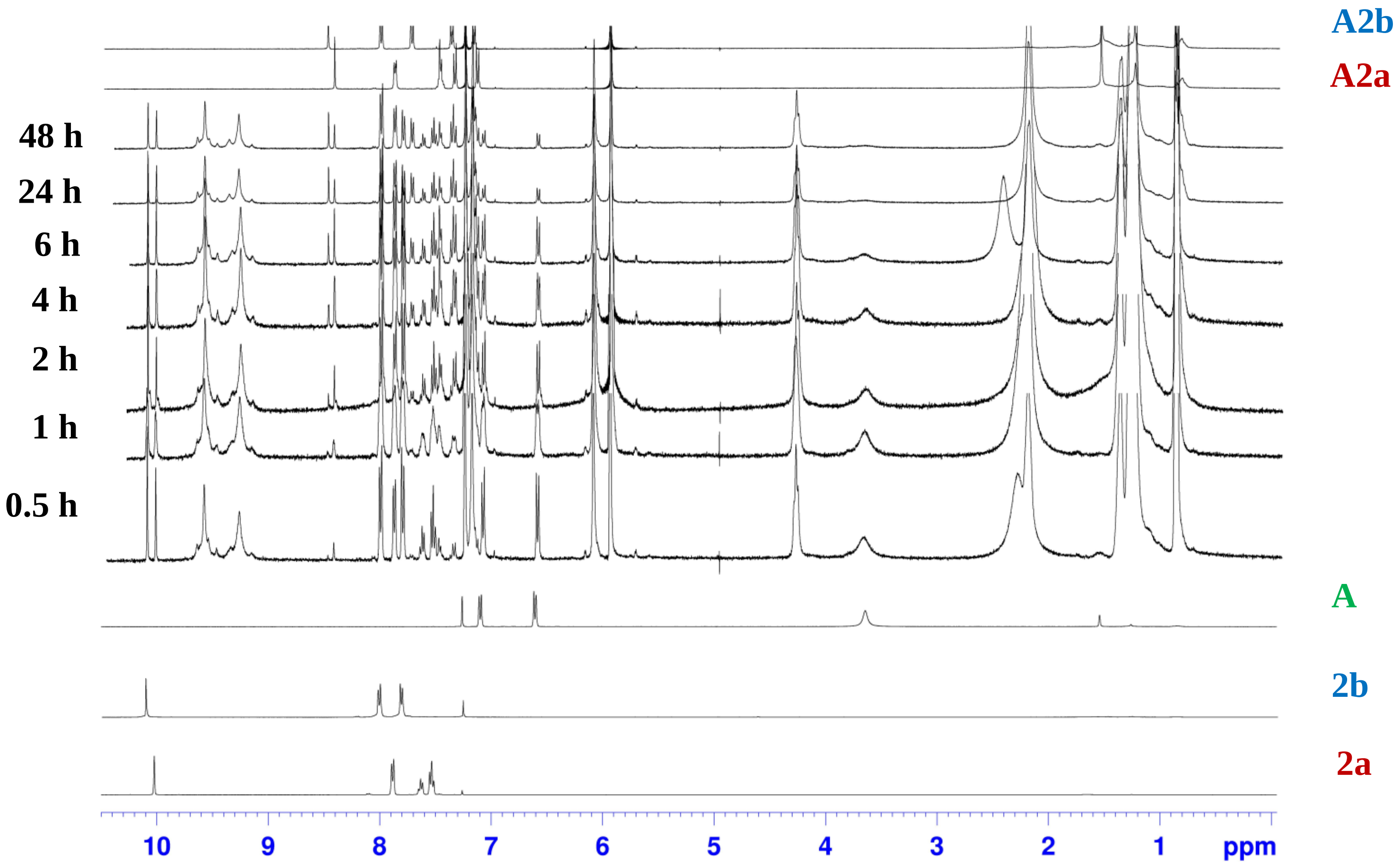

Figure S18. ^{1}H NMR (400 MHz, $CDCl_3$, 298 K) spectra of an equimolar mixture of **A, 2a, 2b** (0.0423 mmol each, water saturated $CDCl_3$, r.t.) and **CR**$_6$ (0.00423 mmol) after 0.5, 1, 2, 4, 6 and 24 h. (Bottom), ^{1}H NMR spectra of **A, 2a** and **2b**. (Top) ^{1}H NMR spectrum of the isolated imines **A2a** and **A2b**.

Table S4. Time-dependent conversion in **A2a** and **A2b** of an equimolar mixture of **A, 2a** and **2b,** in presence of different amount of **CR_6** (Figure 9 in the main text).[a]

	in presence of CR_6 (0.5 equiv)		in presence of CR_6 (0.1 equiv)	
**Time[b] (h)**	**A2a (%)[c]**	**A2b (%)[c]**	**A2a (%)[c,d]**	**A2b (%)[c,d]**
**0.5**	27	8	14	-[e]
**1**	32	17	21	5
**2**	33	21	22	6
**4**	35	45	28	14
**6**	30	49	42	23
**24**	23	61	38	44
**48**	23	61	38	44

[a] Reaction conditions: **2a**, **2b** (0.0423 mmol, 42.3 mM), **A** (0.0423 mmol, 42.3 mM), capsule **CR_6**, water-saturated $CDCl_3$ (1.0 mL), rt. [b] Time at which an aliquot (30 μL) of the reaction mixture was taken and monitored via ^{1}H-NMR spectrum. [c] Conversion was calculated using TCE as internal standard. [d] Conversion was calculated after addition of DMSO (2 μL) to a reaction aliquot, in order to disaggregate the capsule. [e]Below the detection limit. Error in ^{1}H-NMR signal integration was ± 5%.

6.3.2 *Evidence of the capsule effect on the imine distribution in DCL **2a**, **2b** and **A*** (Figure 5 in the main text).

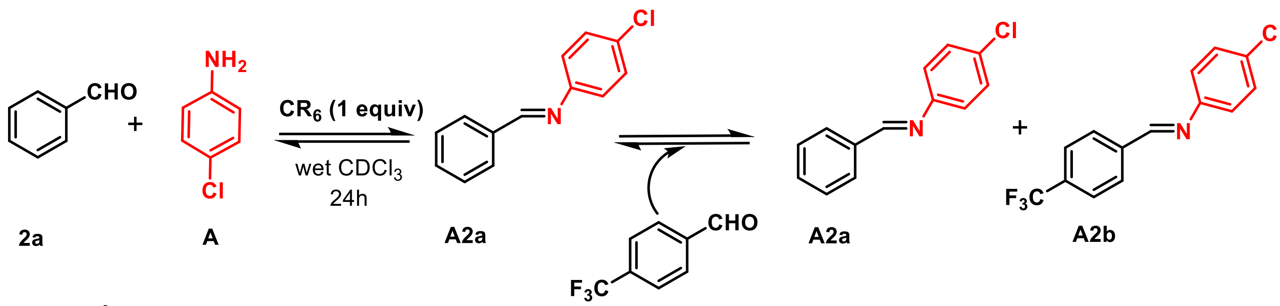

Scheme S7.

Resorcinarene **1** (281.6 mg, 254.7 μmol, 6 equiv) was weighed in a 4 mL vial and 1 mL of water saturated deuterated chloroform was added. The mixture was warmed at 50 °C until clarification (ca 5 min). Then, **2a** (0.0423 mmol, 1 equiv) and **A** (0.0423 mmol, 1 equiv) were added simultaneously, and the reaction mixture was vigorously stirred (1400 rpm) at 30 °C. After 24 h, the mixture was added of **2b** (0.0423 mmol, 1 equiv) and the evolution of the system composition was monitored by ^{1}H NMR as a function of time.

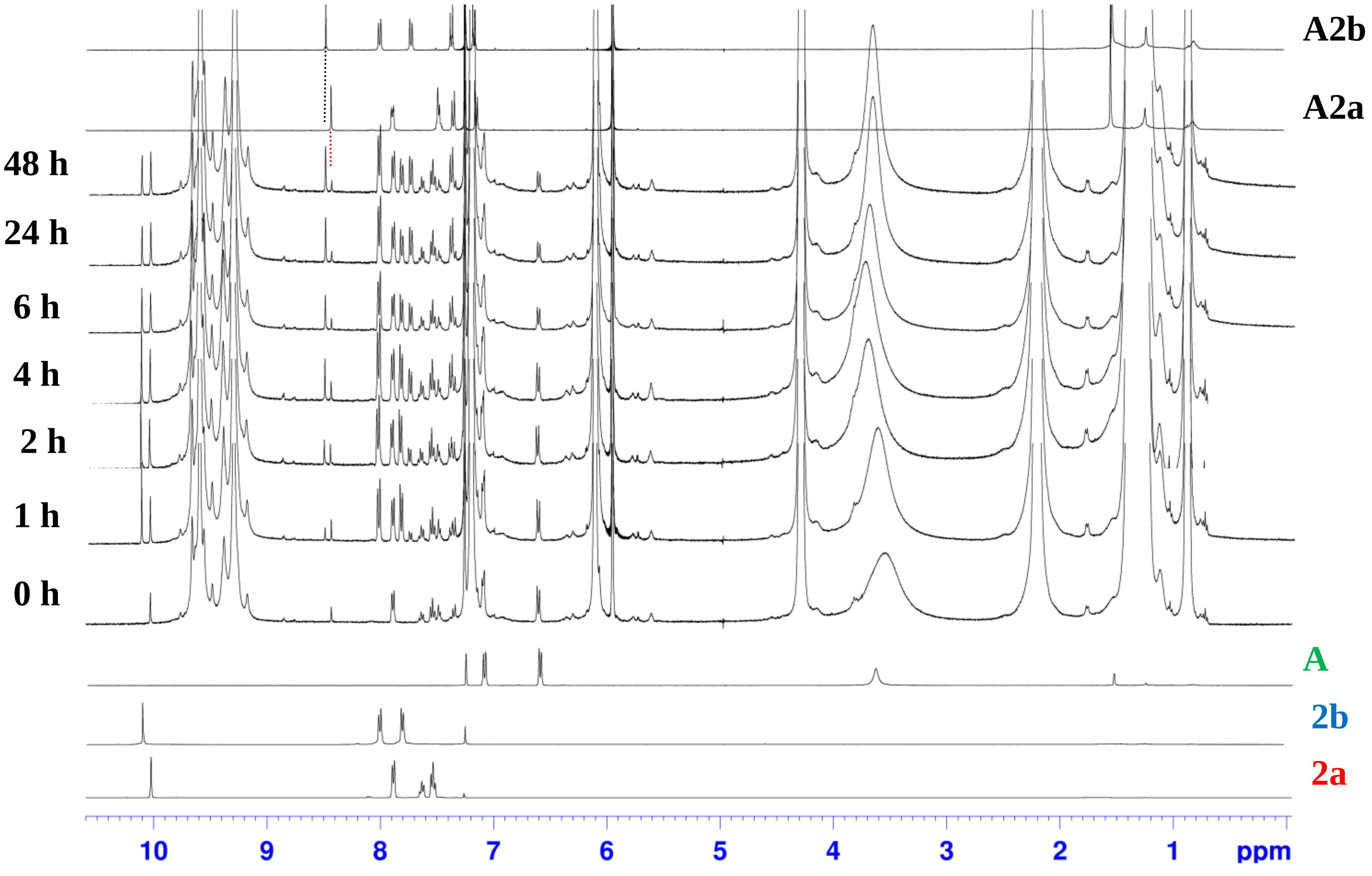

Figure S19. ^{1}H NMR (400 MHz, $CDCl_3$, 298 K) spectra of an equimolar mixture of **A, 2a** and **CR_6** (42.3 mM each, water saturated $CDCl_3$, r.t.) after 1, 2, 4, 6, 24 and 48 h by the addition of **2b** (0.0423 mmol) respectively. (Bottom) ^{1}H NMR spectra of **2a, 2b** and **A**. (Top) ^{1}H NMR spectra of imines **A2a** and **A2b**

6.4 Competitive reaction between equivalent amounts of 2a, 2c and A with and without capsule CR_6

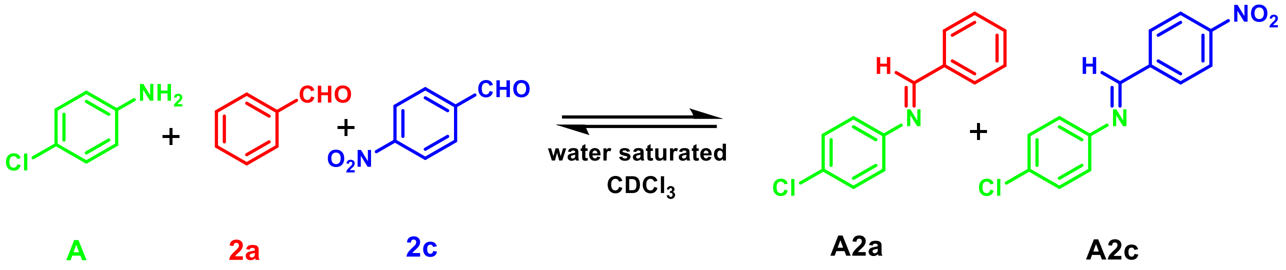

Scheme S8. Synthesis of **A2a** and **A2c** in absence of capsule **CR₆**.

Also, we examined the DCL from benzaldehyde **2a**, *p*-nitrobenzaldehyde **2c**, and *p*-chloroaniline **A**, choosing as standard reaction conditions those used in Scheme 2, main text, (30 °C, 42.3 mM for each reagent including $\mathbf{CR_6}$). When the components **2a** (R = H), **2c** (R = NO_2), and **A** were mixed (Scheme), in equimolar ratio in the presence of $\mathbf{CR_6}$ (1 equiv), **A2a** was formed quickly and prevailed initially over **A2c**.

However, 2h later **A2a** started to decrease as **A2c** increased, and the equilibrium was reached after 24 h with a **A2c/A2a** ratio of 60/13. In the absence of $\mathbf{CR_6}$, the reaction was very slow and the imine **A2c** predominated slightly over **A2a**, after prolonged reaction time (Figure S23a).

In conclusion, an adaptation of constituents was thermodynamically driven by the hexameric capsule toward the imine derived by aldehyde bearing an electron-withdrawing group on the phenyl ring, while the constituent **A2a** remained the kinetically favored one.

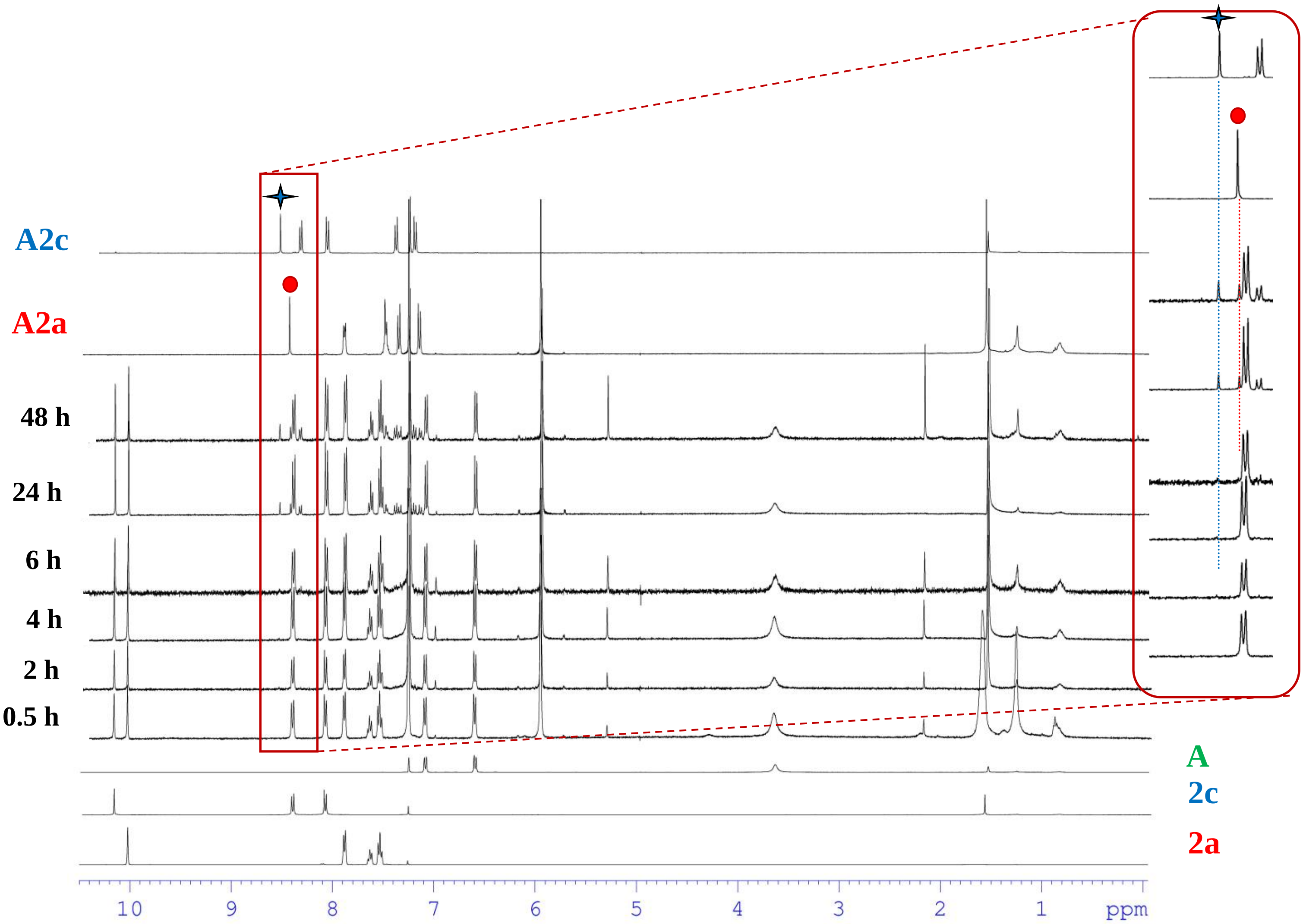

Figure S20. ¹H NMR (400 MHz, $CDCl_3$, 298 K) spectra of an equimolar mixture of **A**, **2a** and **2c** (42.3 mM each, water saturated $CDCl_3$, r.t.) after 0.5, 2, 4, 6, 24 and 48h. (Bottom), ¹H NMR spectra of **2a**, **2c** and **A**. (Bottom) ¹H NMR spectra of imines **A2a** and **A2c**. On the right, in the red panel, the relevant region from 8.4 to 8.6 ppm.

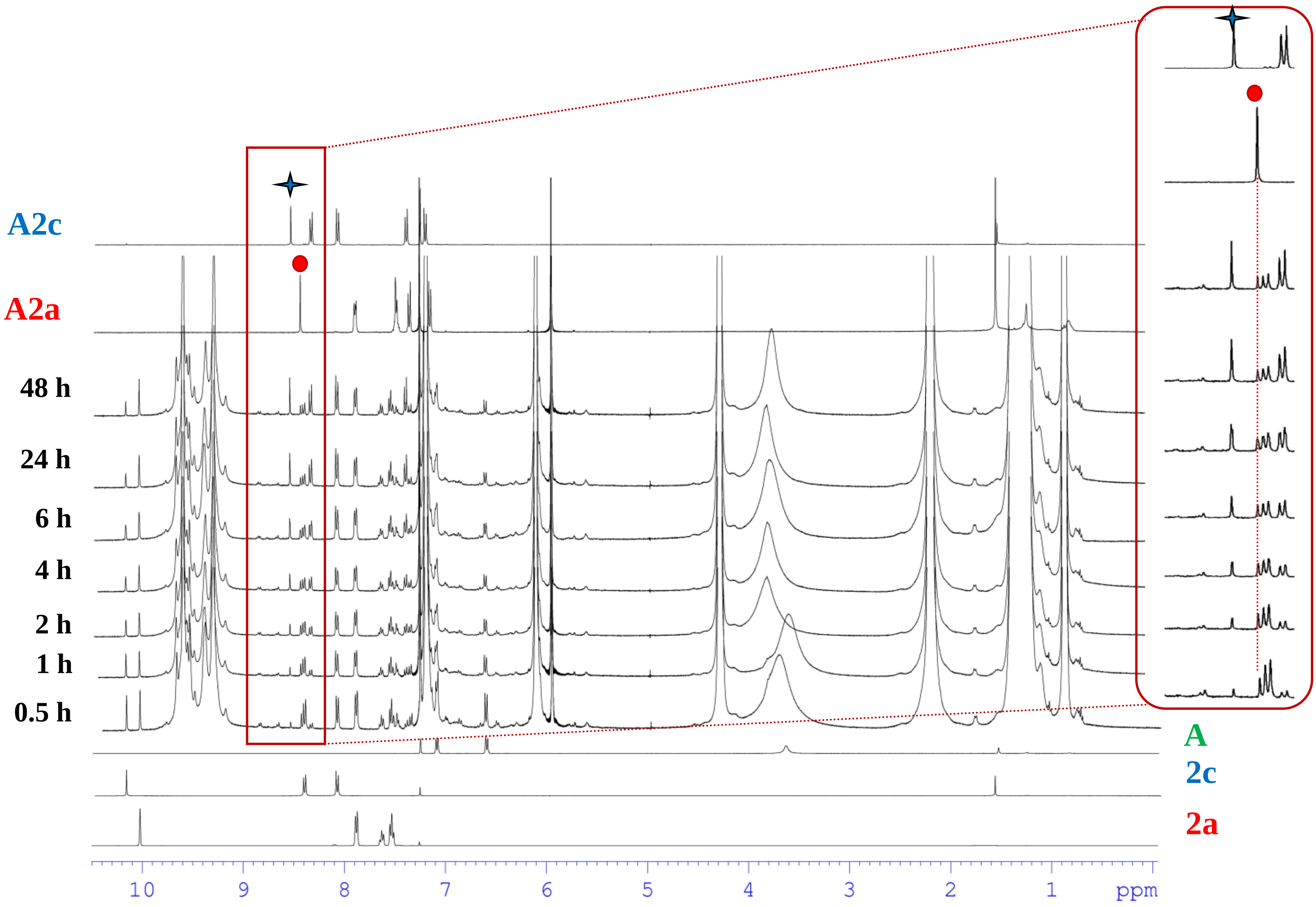

Figure S21. ^{1}H NMR (400 MHz, $CDCl_3$, 298 K) spectra of an equimolar mixture of **A**, **2a, 2c** and **CR_6** (42.3 mM each, water saturated $CDCl_3$, r.t.) after 0.5, 2, 4, 6, 24 and 48 h. (Bottom), ^{1}H NMR spectra of **2a**, **2c** and **A**. (Bottom) ^{1}H NMR spectra of imines **A2a** and **A2c**. On the right, in the red panel, the relevant region from 8.4 to 8.6 ppm.

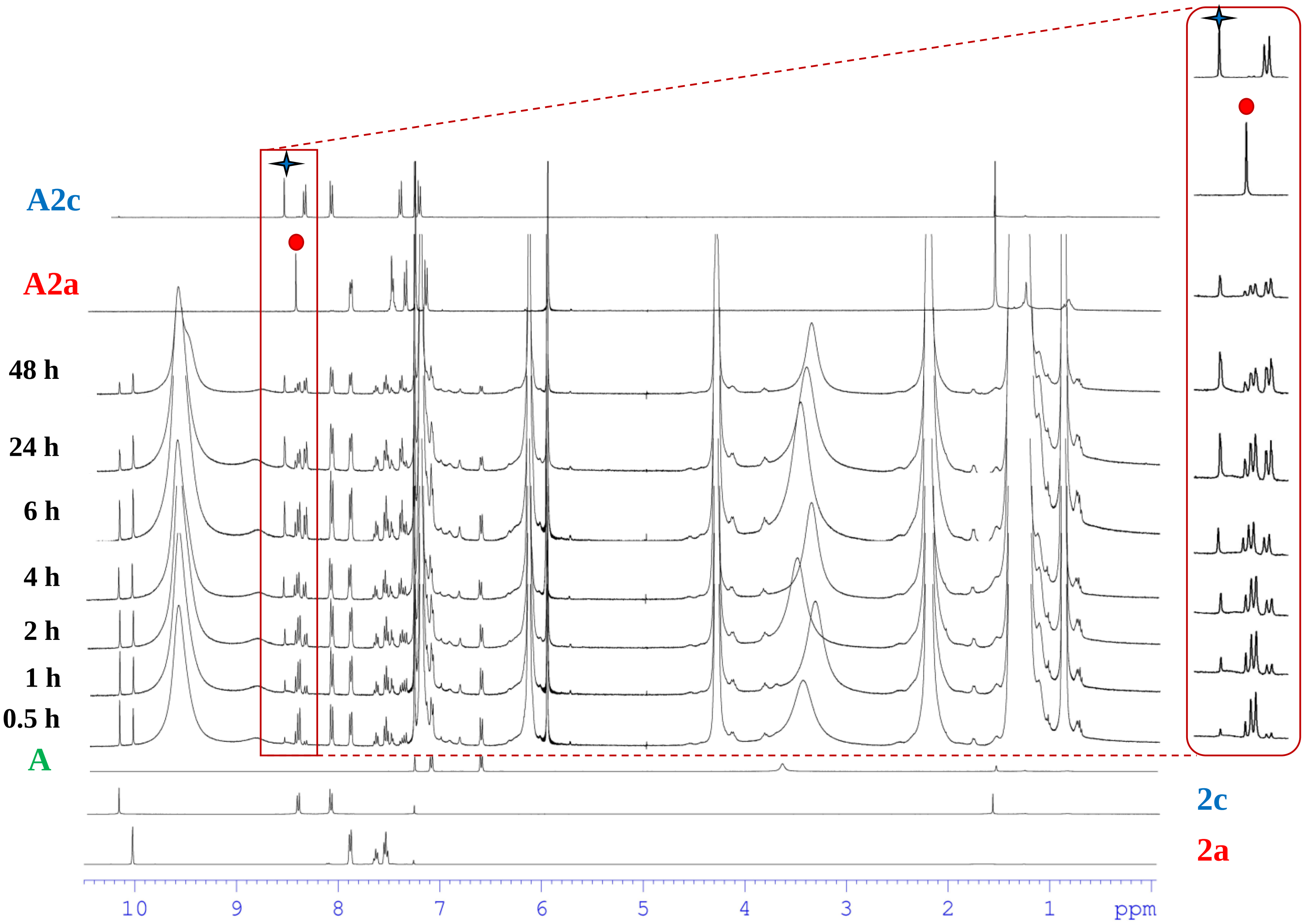

Figure S22. ^{1}H NMR (400 MHz, $CDCl_3$, 298 K) spectra of an equimolar mixture of **A**, **2a, 2c** and $\mathbf{CR_6}$ (42.3 mM each, water saturated $CDCl_3$, r.t.) after 0.5, 2, 4, 6, 24 and 48 h. (Bottom), ^{1}H NMR spectra of **2a**, **2c** and **A**. (Bottom) ^{1}H NMR spectra of imines **A2a** and **A2c**. On the right, in the red panel, the relevant region from 8.4 to 8.6 ppm. The spectra are recorded after addition of DMSO (2 μL) to a reaction aliquot, in order to disaggregate the capsule.

Table S5. Time-dependent conversion in **A2a** and **A2c** of an equimolar mixture of **A, 2a** and **2c,** in presence of different amount of **CR_6** (Figure 9 in the main text).[a]

	in absence of CR_6		in presence of CR_6	
**Time[b] (h)**	**A2a (%)[c]**	**A2c (%)[c]**	**A2a (%)[c,d]**	**A2c (%)[c,d]**
**0.5**	-[e]	-[e]	18	7
**1**	-[e]	-[e]	20	13
**2**	-[e]	-[e]	20	24
**4**	-[e]	-[e]	20	36
**6**	-[e]	-[e]	21	42
**24**	10	13	13	60
**48**	10	13	13	60

[a] Reaction conditions: **2a**, **2c** (0.0423 mmol, 42.3 mM), **A** (0.0423 mmol, 42.3 mM), capsule CR_6 (42.3 mM), water-saturated $CDCl_3$ (1 mL), rt. [b] Time at which an aliquot (30 μL) of the reaction mixture was taken and monitored via ^{1}H-NMR spectrum. [c] Conversion was calculated using TCE as internal standard. [d] Conversion was calculated after addition of DMSO (2 μL) to a reaction aliquot, in order to disaggregate the capsule. [e]Below the limit of detection. Error in ^{1}H-NMR signal integration was ±5%.

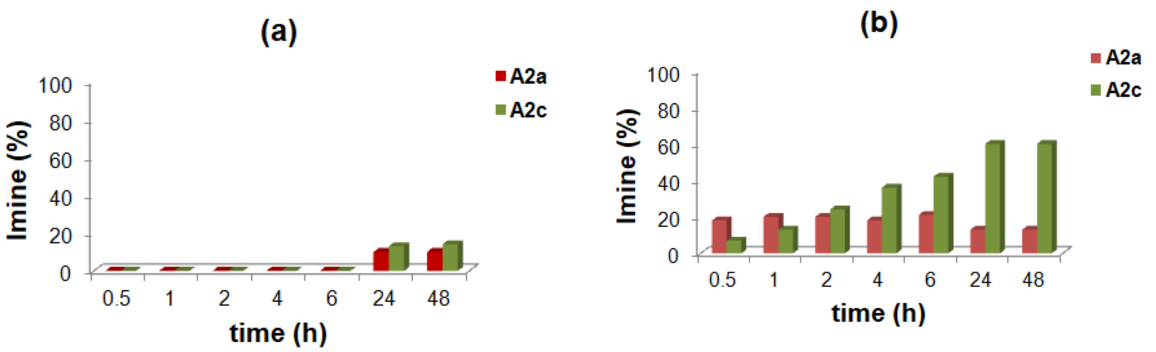

Figure S23. Distribution of imine constituents **A2a** and **A2c** in the DCL, without (**a**) and with (**b**) capsule **CR_6**.

6.5 Competitive reaction between equivalent amounts of 2a, 2d and A with and without capsule CR_6

When the less reactive *p*-OMe-benzaldehyde **2d** was used together with **2a** and **A** as component of the DCL, then the formation of imines **A2a** and **A2d** was observed in very low yields in the presence of $\mathbf{CR_6}$. Interestingly, in this case, the imine **A2a** was favored over the time (Figure s25-26).

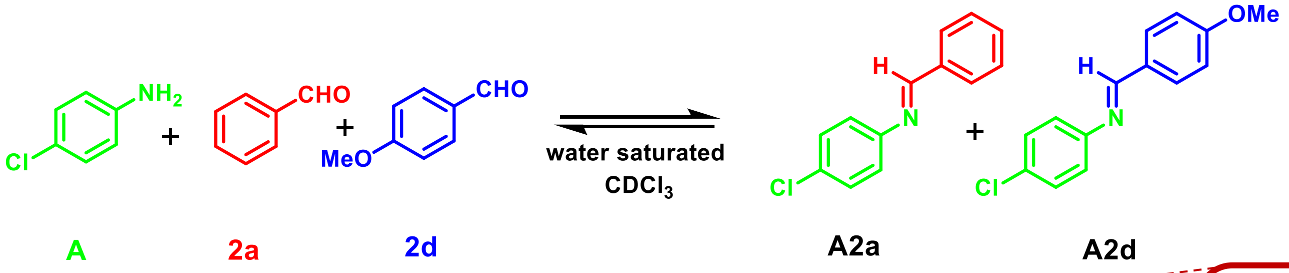

Scheme S9. Synthesis of **A2a** and **A2d** in absence of capsule $\mathbf{CR_6}$.

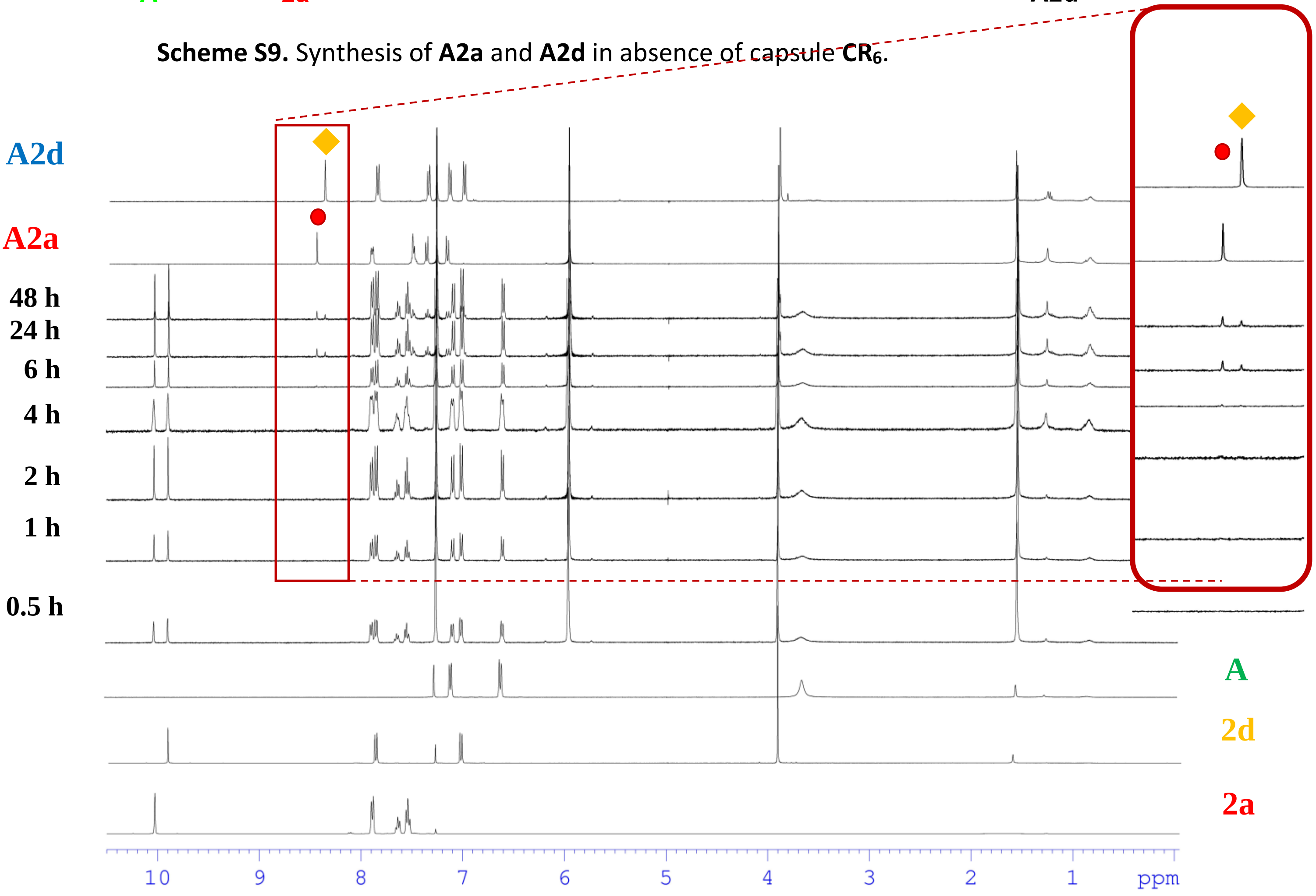

Figure S24. ^{1}H NMR (400 MHz, $CDCl_3$, 298 K) spectra of an equimolar mixture of **A**, **2a** and **2d** (42.3 mM each, water saturated $CDCl_3$, r.t.) after 0.5, 1, 2, 4, 6, 24 and 48 h. (Bottom) ^{1}H NMR spectra of **2a**, **2d** and **A.** (Top) ^{1}H NMR spectra of imines **A2a** and **A2d**. On the right, in the red panel, the relevant region from 8.3 to 8.5 ppm.

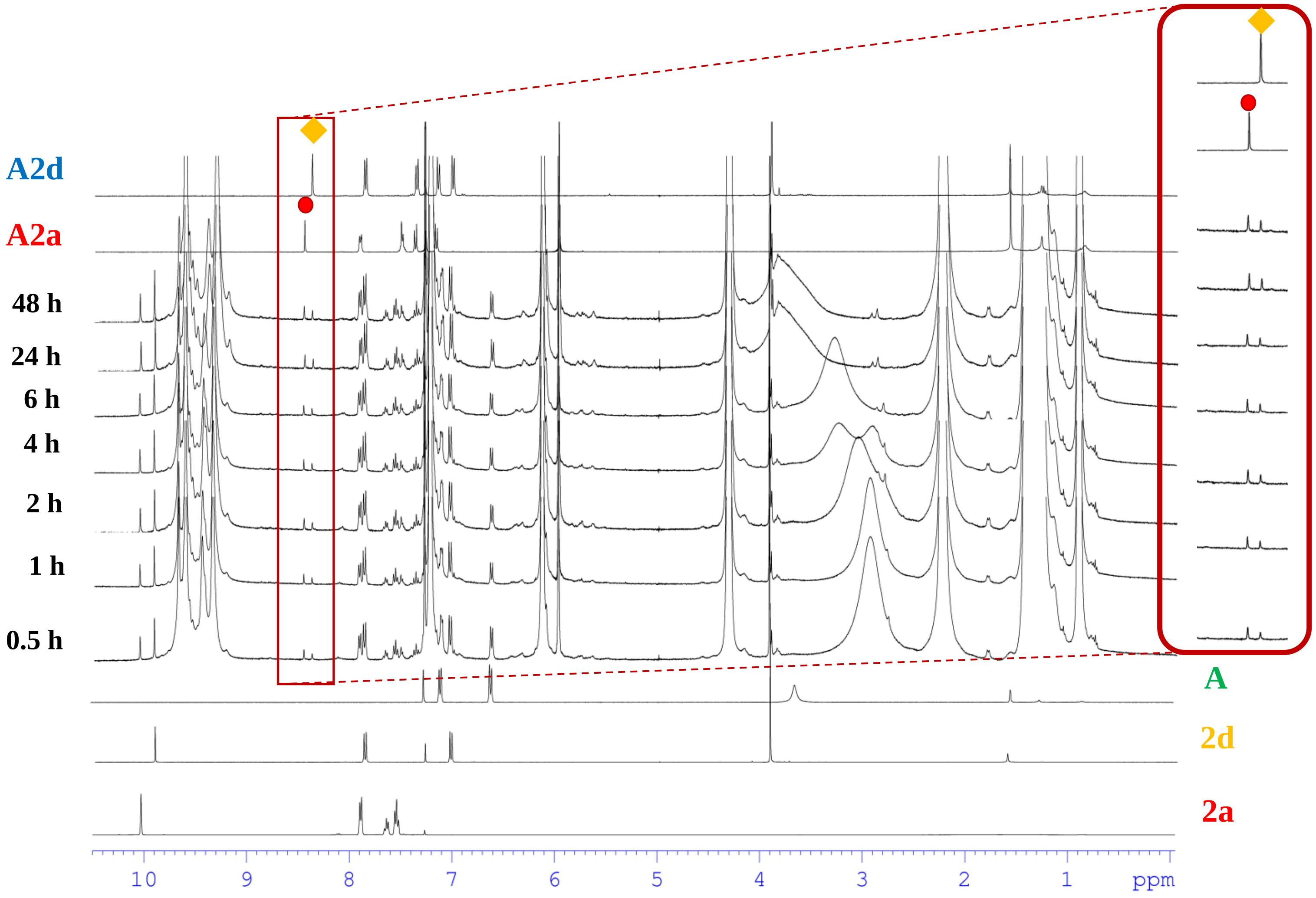

Figure S25. ^{1}H NMR (400 MHz, $CDCl_3$, 298 K) spectra of an equimolar mixture of **A**, **2a, 2d** and **CR₆** (42.3 mM each, water saturated $CDCl_3$, r.t.) after 0.5, 1, 2, 4, 6, 24 and 48 h. (Bottom) ^{1}H NMR spectra of **2a**, **2d** and **A.** (Top) ^{1}H NMR spectra of imines **A2a** and **A2d**. On the right, in the red panel, the relevant region from 8.3 to 8.5 ppm.

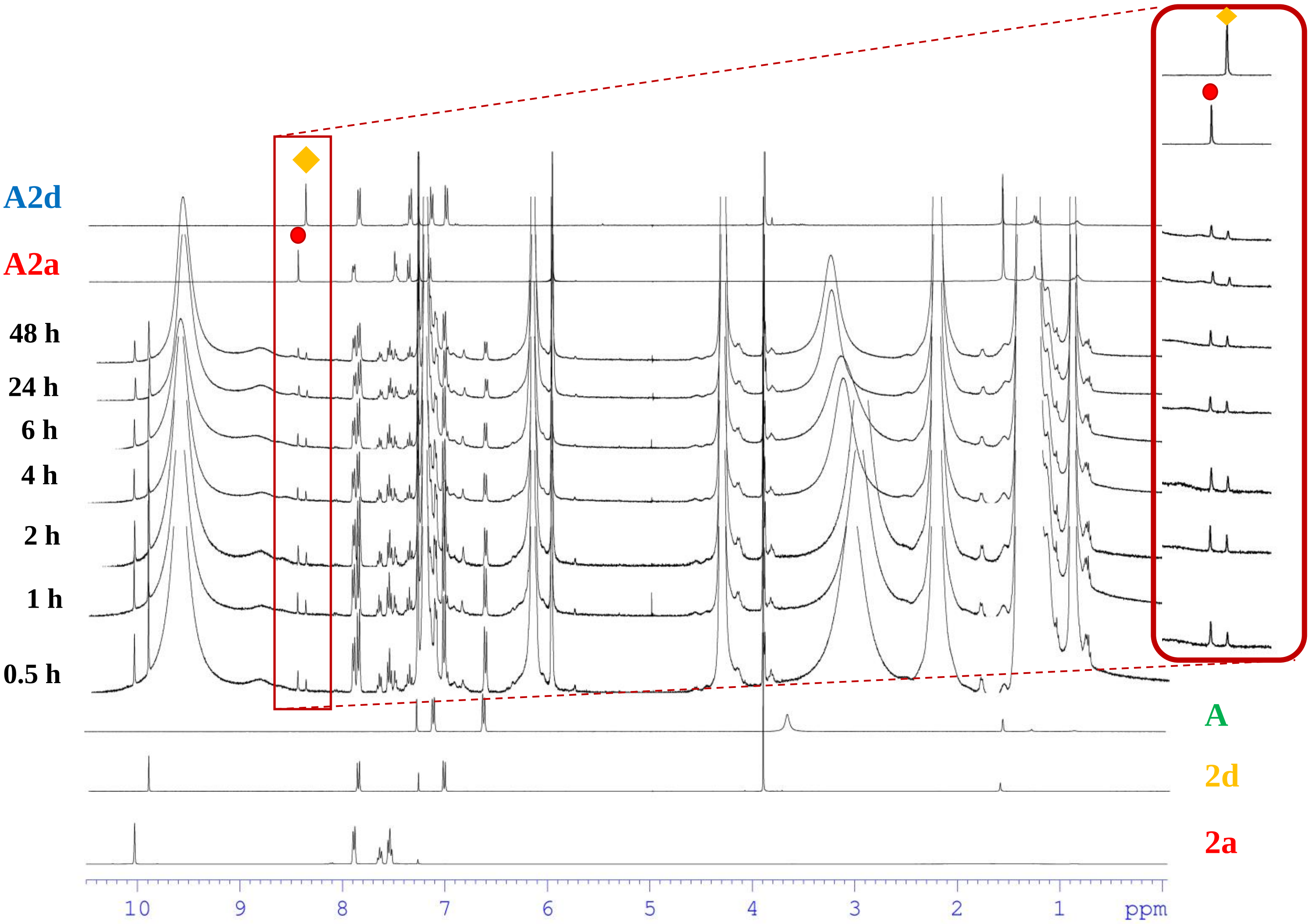

Figure S26. ^{1}H NMR (400 MHz, $CDCl_3$, 298 K) spectra of an equimolar mixture of **A, 2a, 2d** and **CR$_6$** (42.3 mM each, water saturated $CDCl_3$, r.t.) after 0.5, 1, 2, 4, 6, 24 and 48 h. The spectra are recorded after addition of DMSO (2 μL) to a reaction aliquot, in order to disaggregate the capsule. (Bottom) ^{1}H NMR spectra of **2a, 2d** and **A**. (Top) ^{1}H NMR spectra of **A2a** and **A2d**. On the right, in the red panel, the relevant region from 8.3 to 8.5 ppm.

Table S6. Time-dependent conversion in imines **A2a** and **A2d** of an equimolar mixture of **2a, 2d** and **A** in presence and in absence of **CR_6**.[a]

	in absence of CR_6		*in presence of CR_6*	
**Time[b] (h)**	**A2a (%)[c]**	**A2d (%)[c]**	**A2a (%)[c,d]**	**A2d (%)[c,d]**
**0.5**	-[e]	-[e]	19	9
**1**	-[e]	-[e]	20	14
**2**	-[e]	-[e]	25	13
**4**	-[e]	-[e]	28	18
**6**	-[e]	-[e]	31	17
**24**	14	7	31	17
**48**	14	7	31	17

[a] Reaction conditions: **2a**, **2d** (0.0423 mmol, 42.3 mM), **A** (0.0423 mmol, 42.3 mM), capsule CR_6 (42.3 mM), water-saturated $CDCl_3$ (1 mL), rt. [b] Time at which an aliquot (30 μL) of the reaction mixture was taken and monitored via ^{1}H-NMR spectrum. [c] Conversion was calculated using TCE as internal standard. [d] Conversion was calculated after addition of DMSO (2 μL) to a reaction aliquot, in order to disaggregate the capsule. [e]Below the limit of detection. Error in ^{1}H-NMR signal integration was ±5%.

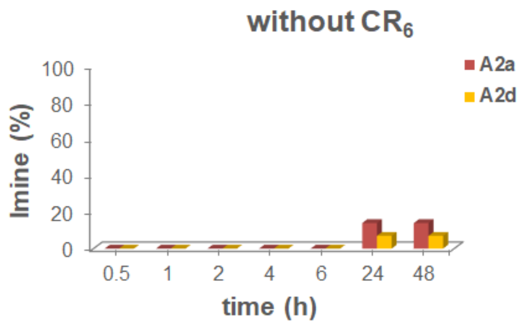

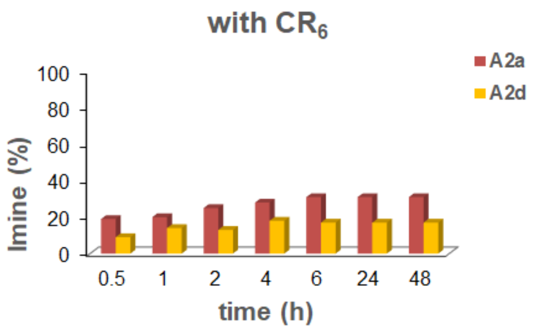

Figure S27. Distribution of imine constituents **A2a** and **A2d** in the DCL, without (a) and with (b) capsule **CR_6**

6.6 Competitive reaction between equivalent amounts of 2a, 2b and B with and without capsule CR6 (Figure 10 in the main text).

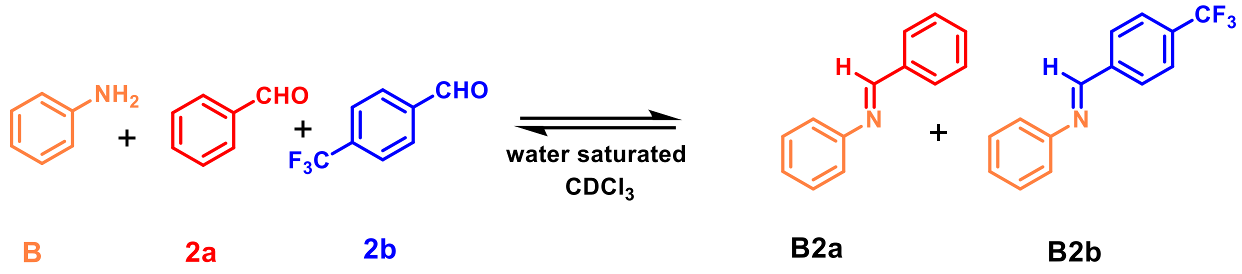

Scheme S10. Synthesis of **B2a** and **B2b** in absence of capsule **CR6**.

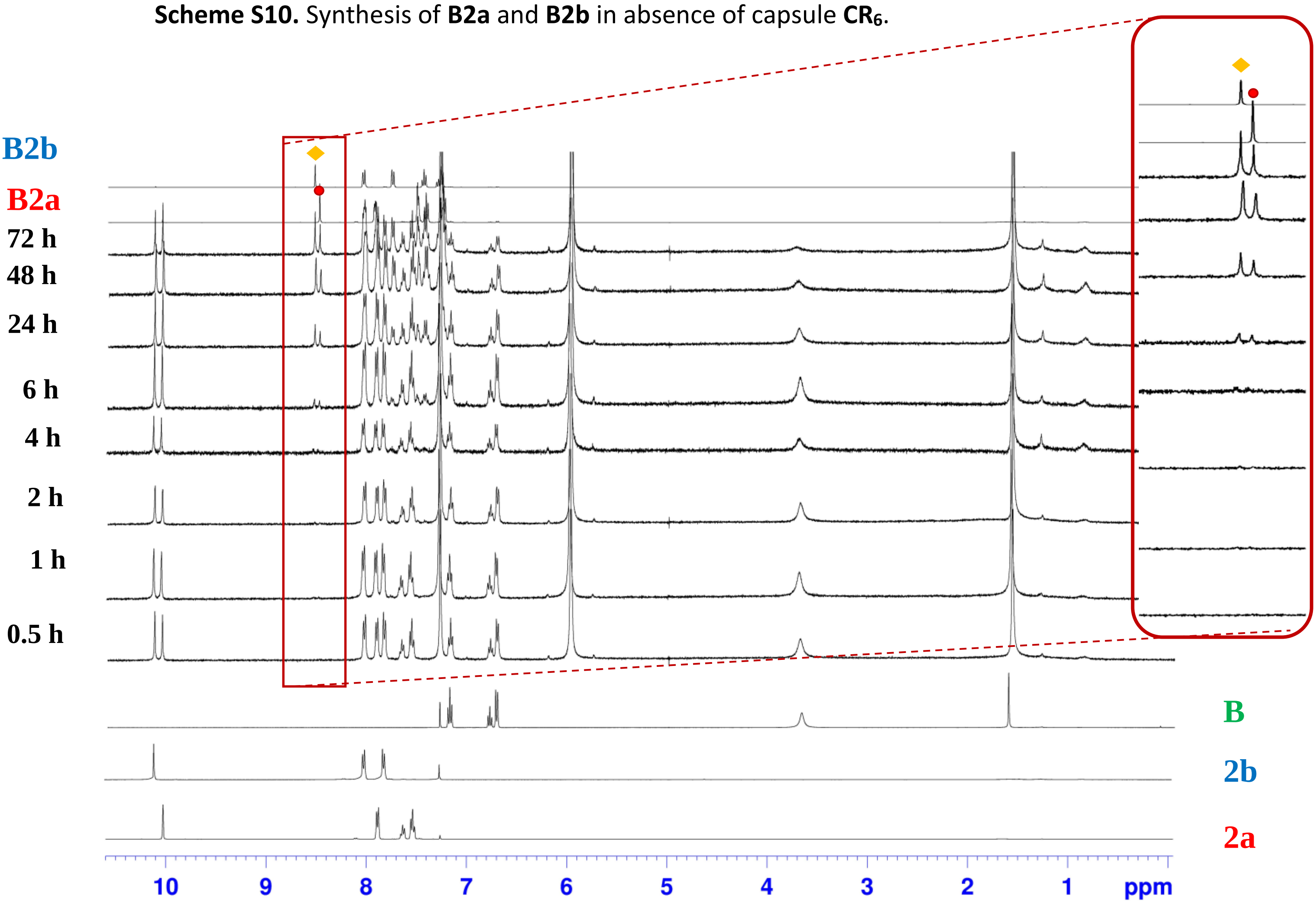

Figure S28. ^{1}H NMR (400 MHz, $CDCl_3$, 298 K) spectra of an equimolar mixture of **B**, **2a** and **2b** (42.3 mM each, water saturated $CDCl_3$, r.t.) after 0.5, 1, 2, 4, 6 , 24, 48 and 72 h. (Bottom) ^{1}H NMR spectra of **2a, 2b** and **B**. (Top) ^{1}H NMR spectra of **B2a** and **B2b**. On the right, in the red panel, a relevant selected region from 8.3 to 8.6 ppm.

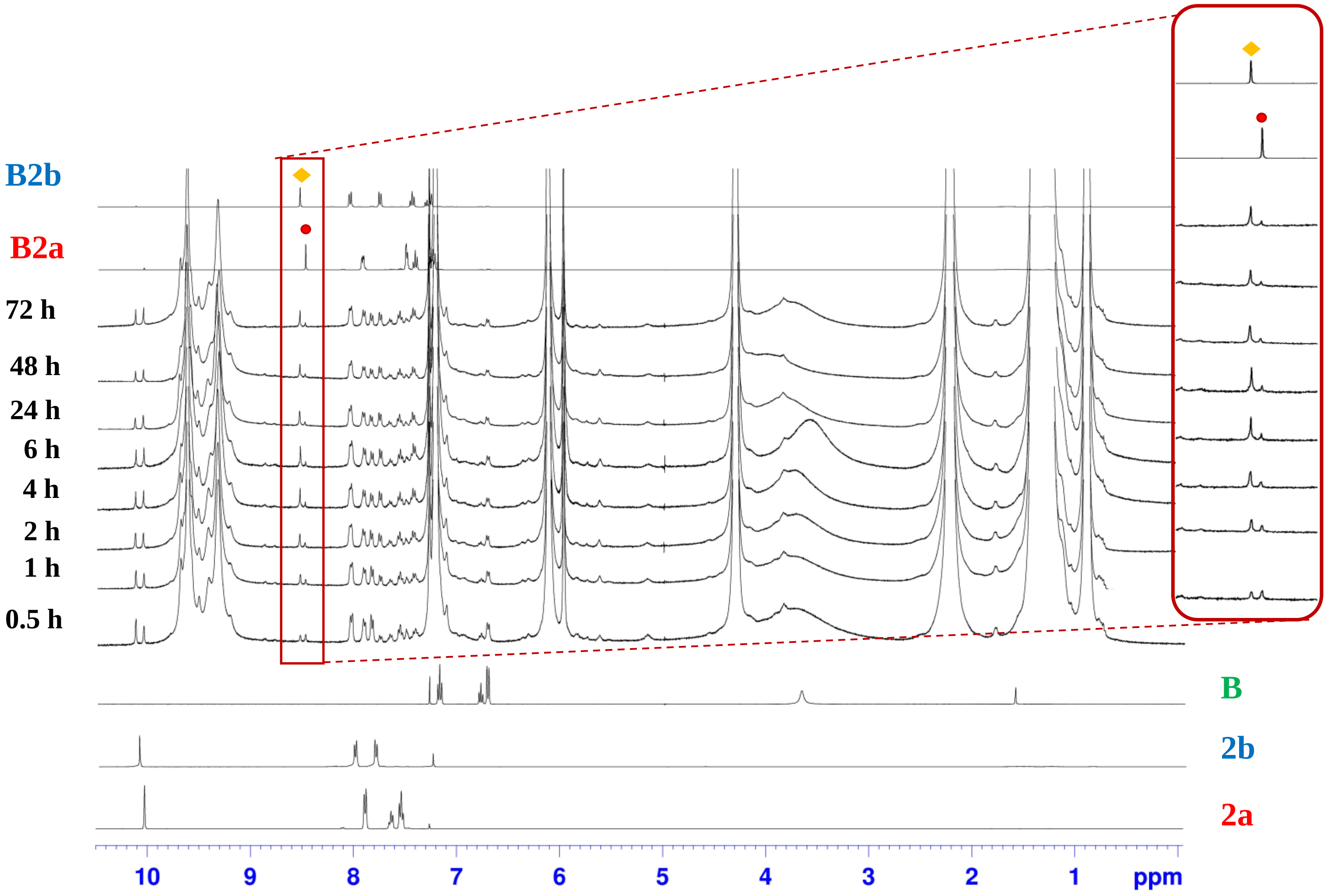

Figure S29. ^{1}H NMR (400 MHz, $CDCl_3$, 298 K) spectra of an equimolar mixture of **B, 2a, 2b** and **CR$_6$** (42.3 mM each, water saturated $CDCl_3$, r.t.) after 0.5, 1, 2, 4, 6, 24, 48 and 72 h. (Bottom) ^{1}H NMR spectra of **2a, 2b** and **B**. (Top) ^{1}H NMR spectra of **B2a** and **B2b**. On the right, in the red panel, a relevant selected region from 8.3 to 8.6 ppm.

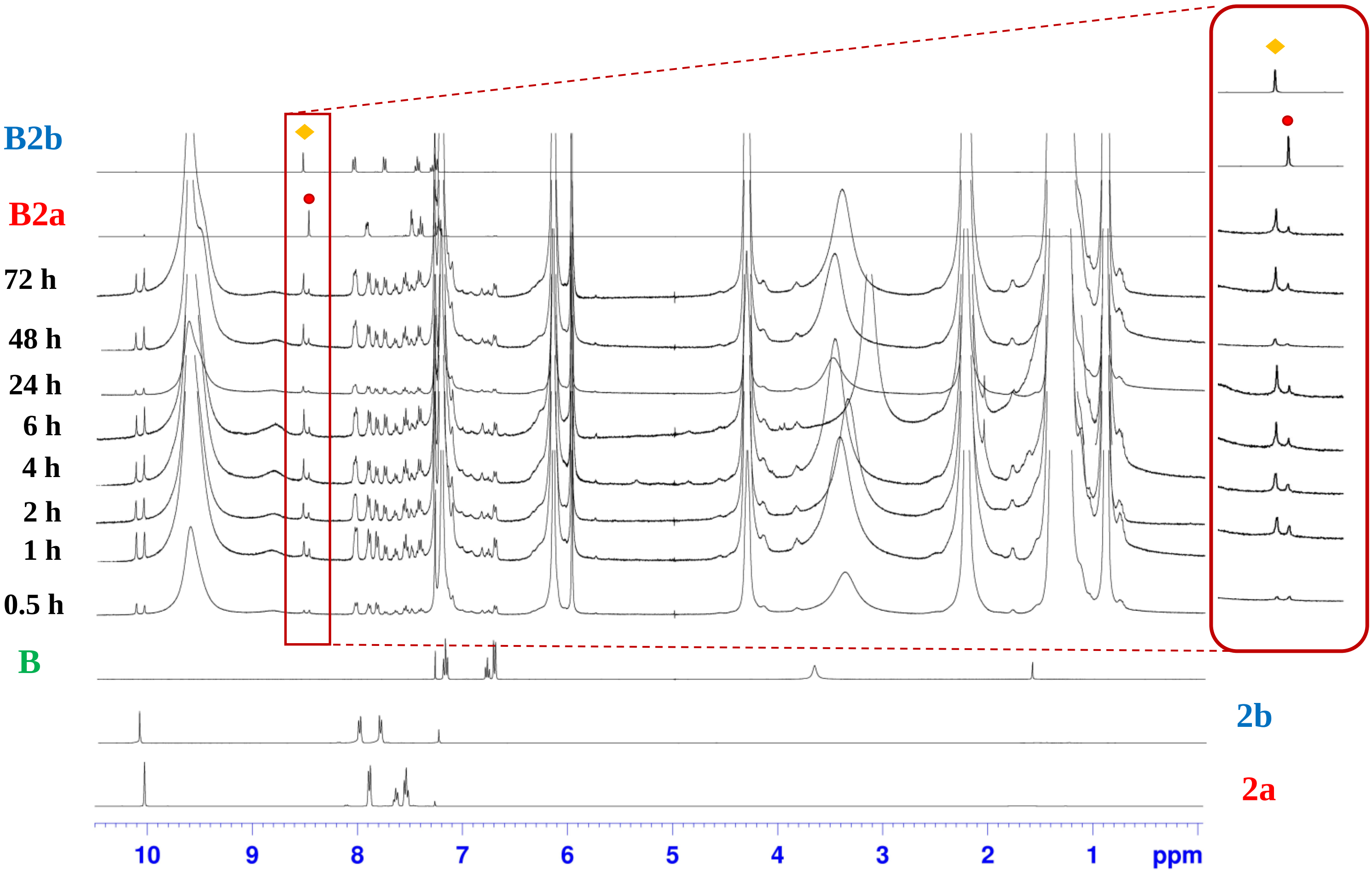

Figure S30. ^{1}H NMR (400 MHz, $CDCl_3$, 298 K) spectra of an equimolar mixture of **B, 2a, 2b** and **CR_6** (42.3 mM each, water saturated $CDCl_3$, r.t.) after 0.5, 1, 2, 4, 6, 24, 48 and 72 h. (Bottom) ^{1}H NMR spectra of **2a, 2b** and **B**. (Top) ^{1}H NMR spectra of **B2a** and **B2b**. On the right, in the red panel, a relevant selected region from 8.3 to 8.6 ppm. The spectra are recorded after addition of DMSO (2 µL) to a reaction aliquot, in order to disaggregate the capsule

Table S7. Time-dependent conversion in imines **B2a** and **B2b** of an equimolar mixture of **2a, 2b** and **B** in presence and in absence of **CR$_6$** (Figure 10 in the main text).[a]

	in absence of CR$_6$		***in presence of CR$_6$***	
**Time[b] (h)**	**B2a (%)[c]**	**B2b (%)[c]**	**B2a (%)[c,d]**	**B2b (%)[c,d]**
**0.5**	-[e]	-[e]	20	17
**1**	-[e]	-[e]	19	36
**2**	-[e]	-[e]	12	41
**4**	-[e]	-[e]	10	44
**6**	8	10	11	51
**24**	13	20	10	52
**48**	32	51	10	52
**72**	32	51	10	52

[a] Reaction conditions: **2a**, **2b** (0.0423 mmol, 42.3 mM), **B** (0.0423 mmol, 42.3 mM), capsule CR$_6$ (42.3 mM), water-saturated $CDCl_3$ (1 mL), rt. [b] Time at which an aliquot (30 μL) of the reaction mixture was taken and monitored via ^{1}H-NMR spectrum. [c] Conversion was calculated using TCE as internal standard. [d] Conversion was calculated after addition of DMSO (2 μL) to a reaction aliquot, in order to disaggregate the capsule. [e]Below the limit of detection. Error in ^{1}H-NMR signal integration was ±5%.

6.7 Competitive reaction between equivalent amounts of 2a, 2b and C with and without capsule CR_6 (Figure 12 in the main text).

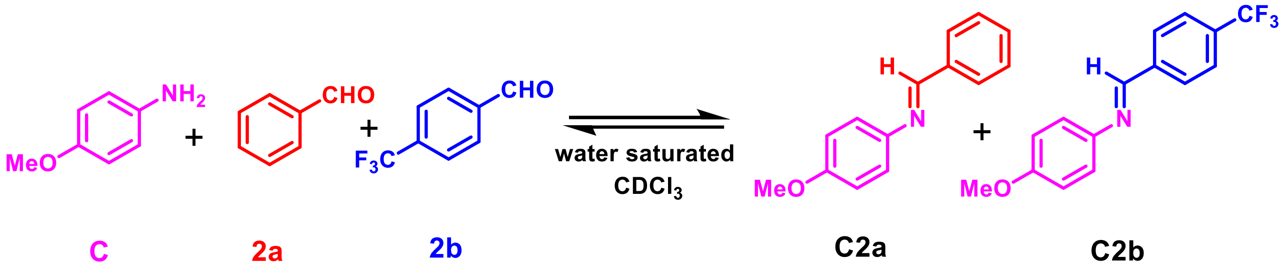

Scheme S11. Synthesis of **C2a** and **C2b** in absence of capsule **CR_6**.

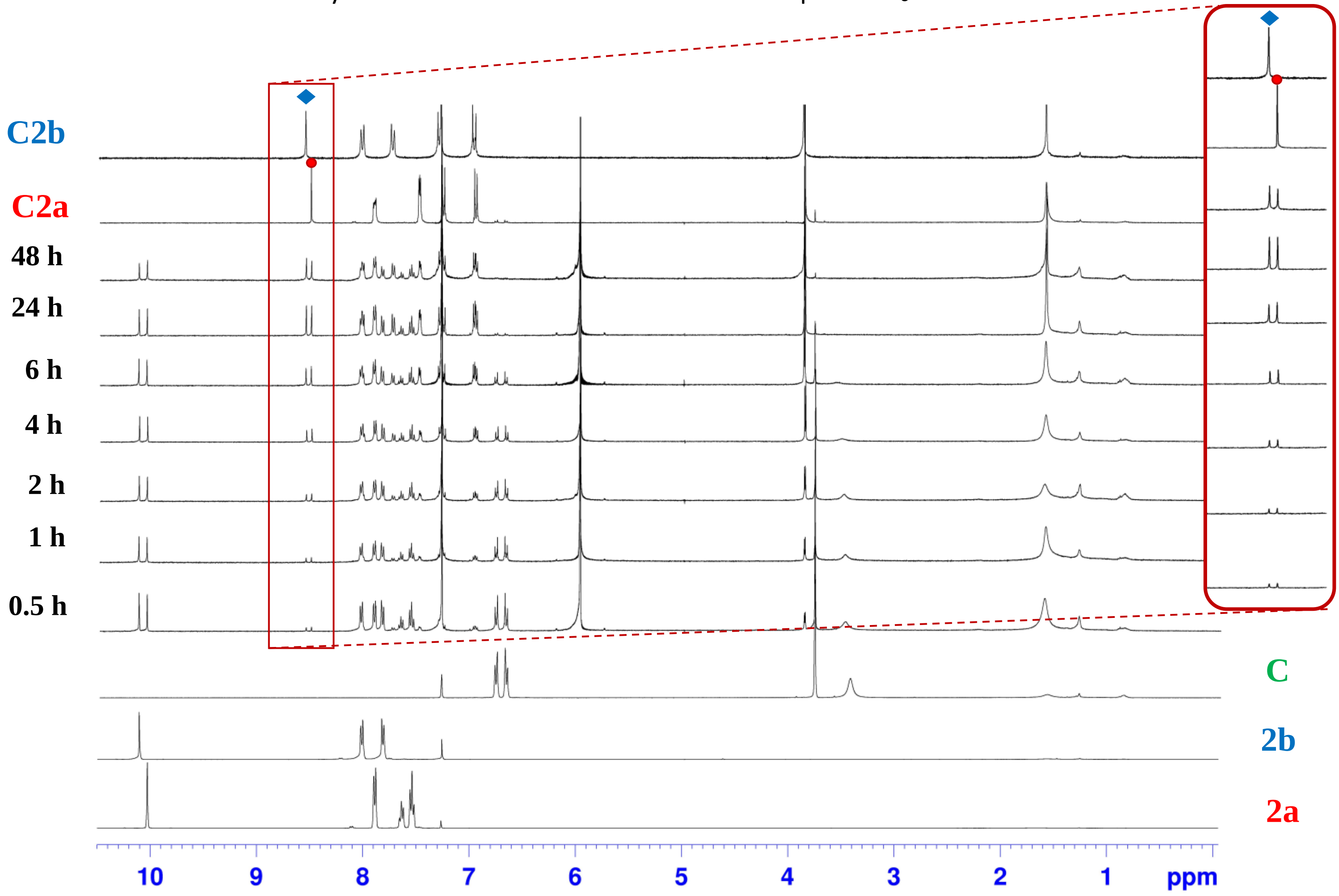

Figure S31. ^{1}H NMR (400 MHz, $CDCl_3$, 298 K) spectra of an equimolar mixture of **C**, **2a** and **2b** (42.3 mM each, water saturated $CDCl_3$, r.t.) after 0.5, 1, 2, 4, 6 , 24 and 48h. (Bottom) ^{1}H NMR spectra of **2a, 2b** and **C**. (Top) ^{1}H NMR spectra of **C2a** and **C2b**. On the right, in the red panel, a relevant selected region from 8.4 to 8.7 ppm.

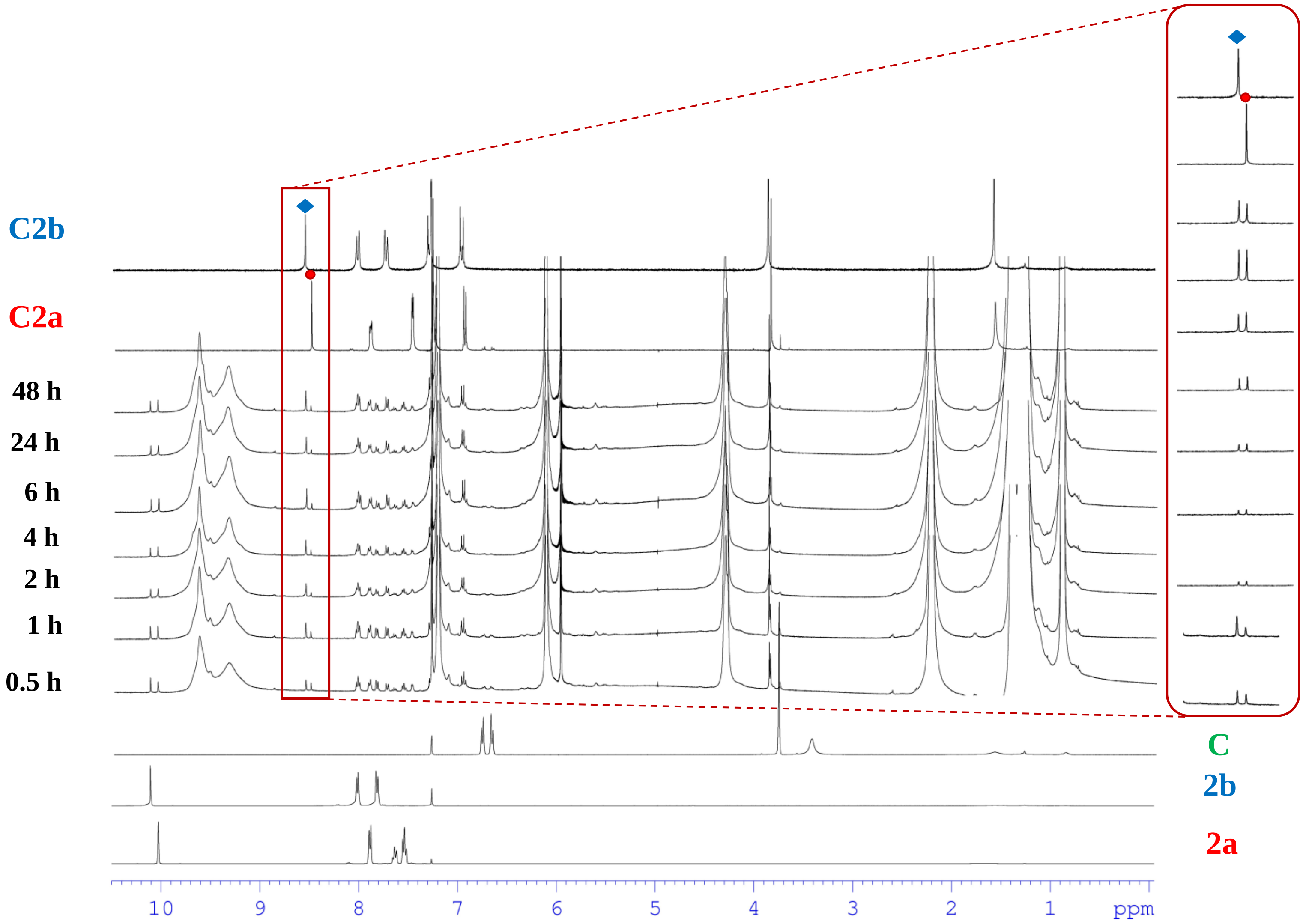

Figure S32. ^{1}H NMR (400 MHz, $CDCl_3$, 298 K) spectra of an equimolar mixture of **C, 2a, 2b** and **CR**$_6$ (42.3 mM each, water saturated $CDCl_3$, r.t.) after 0.5, 1, 2, 4, 6, 24 and 48h. (Bottom) ^{1}H NMR spectra of **2a, 2b** and **C**. (Top) ^{1}H NMR spectra of **C2a** and **C2b**. On the right, in the red panel, a selected region from 8.4 to 8.7 ppm.

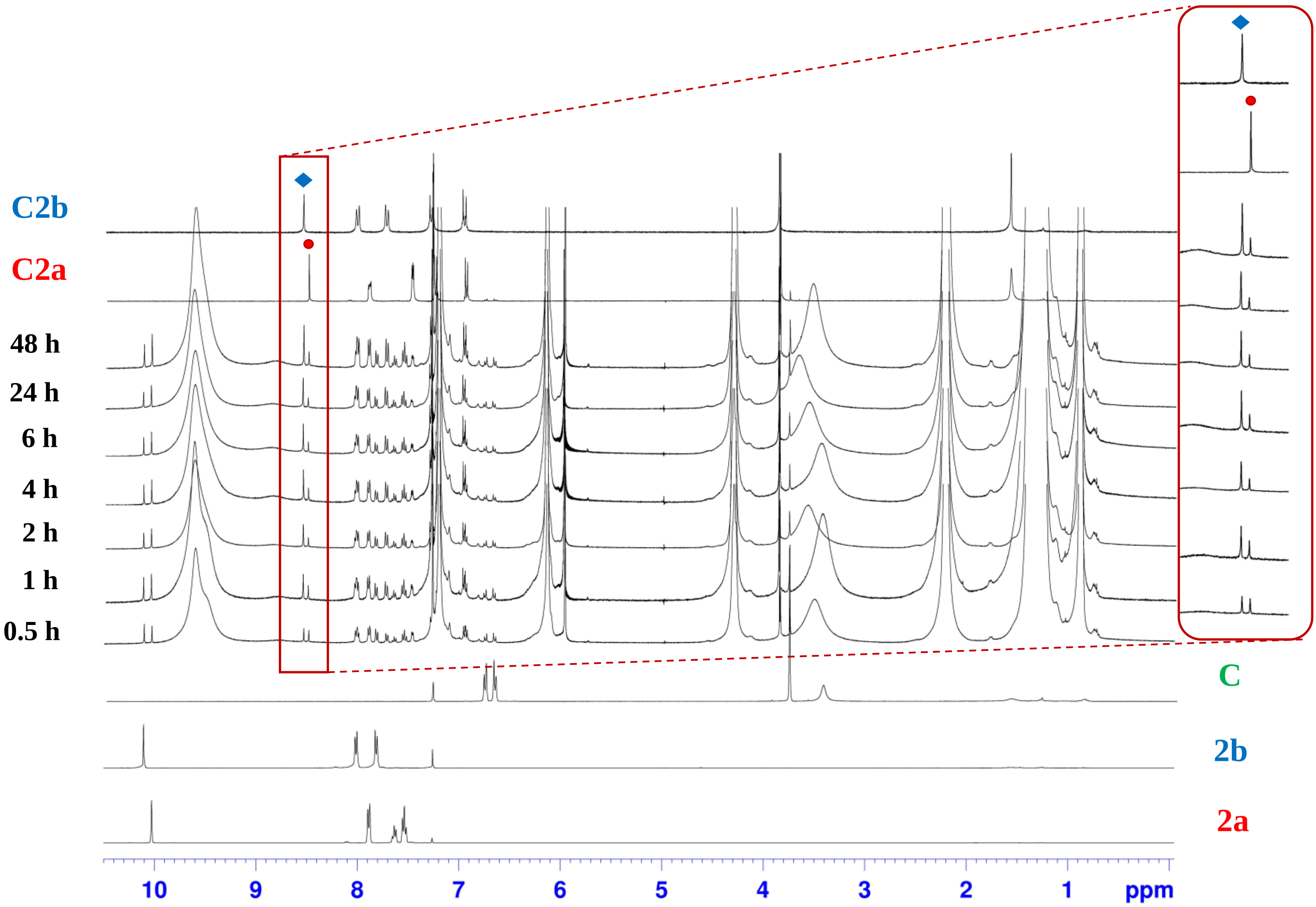

Figure S33. ^{1}H NMR (400 MHz, $CDCl_3$, 298 K) spectra of an equimolar mixture of **C, 2a, 2b** and **CR_6** (42.3 mM each, water saturated $CDCl_3$, r.t.) after 0.5, 1, 2, 4, 6, 24 and 48h. (Bottom) ^{1}H NMR spectra of **2a, 2b** and **C**. (Top) ^{1}H NMR spectra of **C2a** and **C2b**. On the right, in the red panel, a selected region from 8.4 to 8.7 ppm. The spectra are recorded after addition of DMSO (2 μL) to a reaction aliquot, in order to disaggregate the capsule.

Table S8. Time-dependent conversion in imines **C2a** and **C2b** of an equimolar mixture of **2a, 2b** and **C** in presence and in absence of **CR$_6$** (Figure S12 in the main text).[a]

	in absence of CR$_6$		***in presence of CR$_6$***	
**Time[b] (h)**	**C2a (%)[c]**	**C2b (%)[c]**	**C2a (%)[c,d]**	**C2b (%)[c,d]**
**0.5**	13	10	36	52
**1**	16	15	32	67
**2**	20	17	32	67
**4**	34	32	32	67
**6**	41	41	32	67
**24**	55	55	32	67
**48**	55	55	32	67

[a] Reaction conditions: **2a**, **2b** (0.0423 mmol, 42.3 mM), **C** (0.0423 mmol, 42.3 mM), capsule **CR$_6$** (42.3 mM), water-saturated $CDCl_3$ (1 mL), rt. [b] Time at which an aliquot (30 μL) of the reaction mixture was taken and monitored via ^{1}H-NMR spectrum. [c] Conversion was calculated using TCE as internal standard. [d] Conversion was calculated after addition of DMSO (2 μL) to a reaction aliquot, in order to disaggregate the capsule. Error in ^{1}H-NMR signal integration was ± 5%.

6.8 Competitive reaction between equivalent amounts of 2a, 2b and E with and without capsule CR_6

The reaction was performed following the general procedures reported in sections 2.2-2.4.

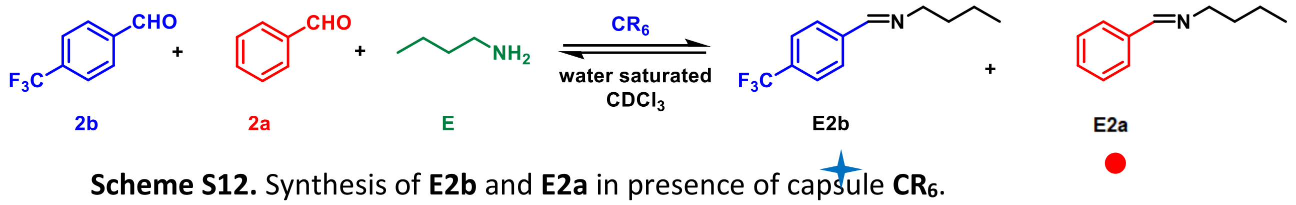

Scheme S12. Synthesis of **E2b** and **E2a** in presence of capsule **CR_6**.

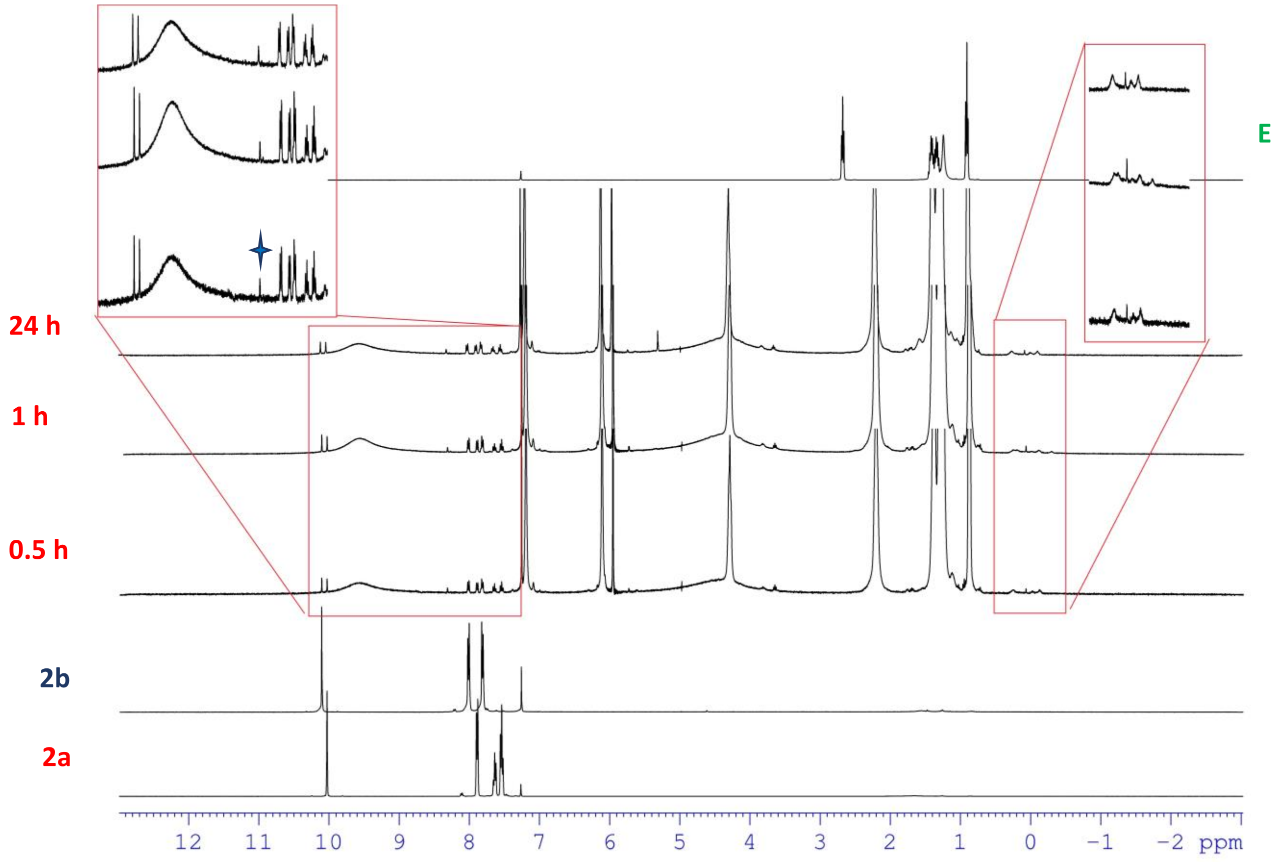

Figure S34. ^1H NMR (400 MHz, $CDCl_3$, 298 K) spectra of an equimolar mixture of **E, 2a, 2b** and **CR_6** (42.3 mM each, water saturated $CDCl_3$, r.t.) after 0.5, 1 and 24 h. (Bottom) ^1H NMR spectra of **2a, 2b** . (Top) ^1H NMR spectra of **E**. On the right, in the red panel, a selected region from -0.50 to 0.41 ppm; on the left, in the red panel, a selected region from 7.44 to 10.22 ppm. The signals of free imines **E2a** and **E2b** were assigned in agreement with reported literature values.[16]

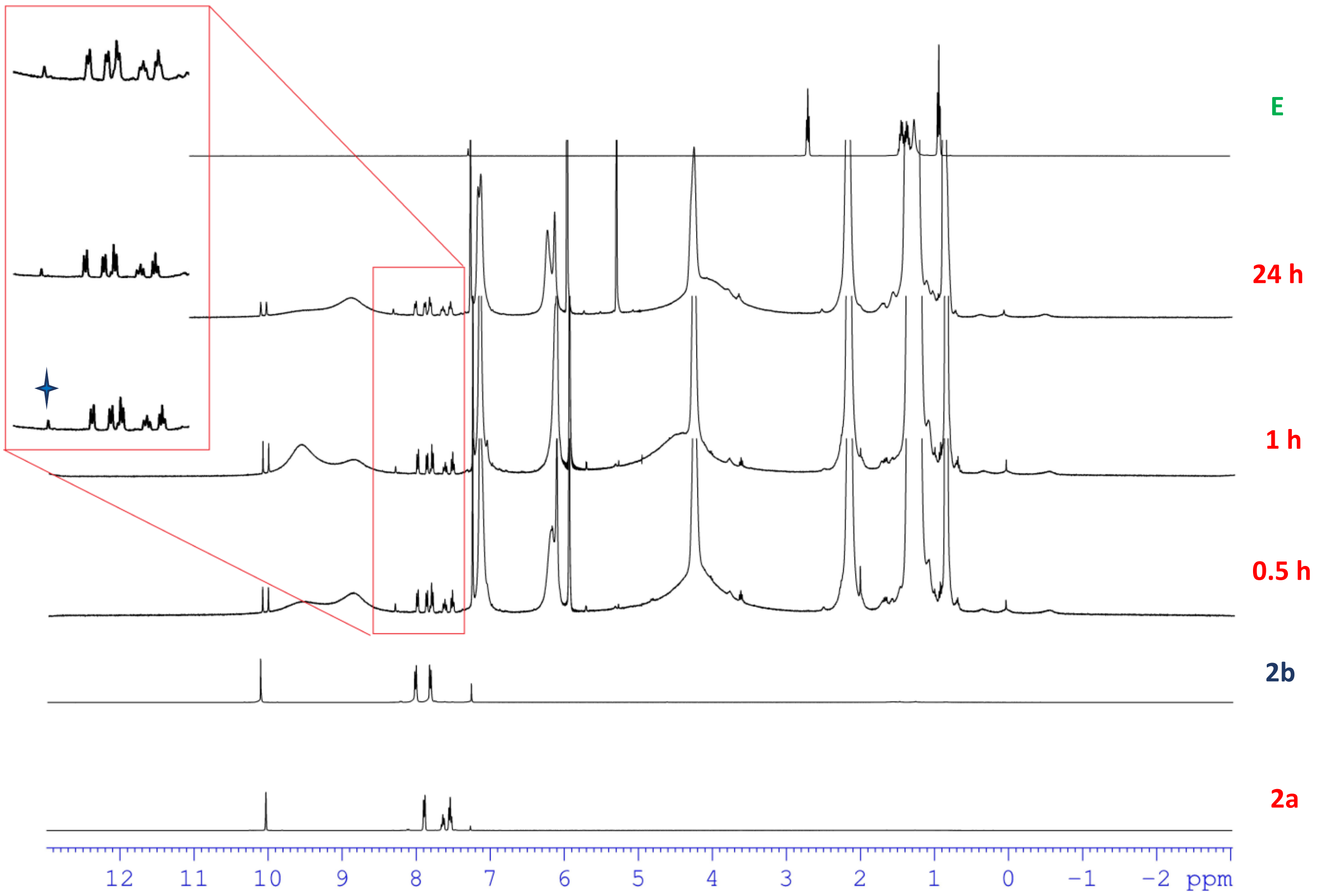

Figure S35. ^{1}H NMR (400 MHz, $CDCl_3$, 298 K) spectra of an equimolar mixture of **E, 2a, 2b** and **CR_6** (42.3 mM each, water saturated $CDCl_3$, r.t.) after 0.5, 1 and 24h. (Bottom) ^{1}H NMR spectra of **2a** and **2b**. (Top) ^{1}H NMR spectra of **E**. On the left, in the red panel, a selected region from 7.4 to 8.4 ppm. The spectra are recorded after addition of DMSO (2 μL) to a reaction aliquot, in order to disaggregate the capsule. The signals of free imines **E2a** and **E2b** were assigned in agreement with reported literature values.[16]

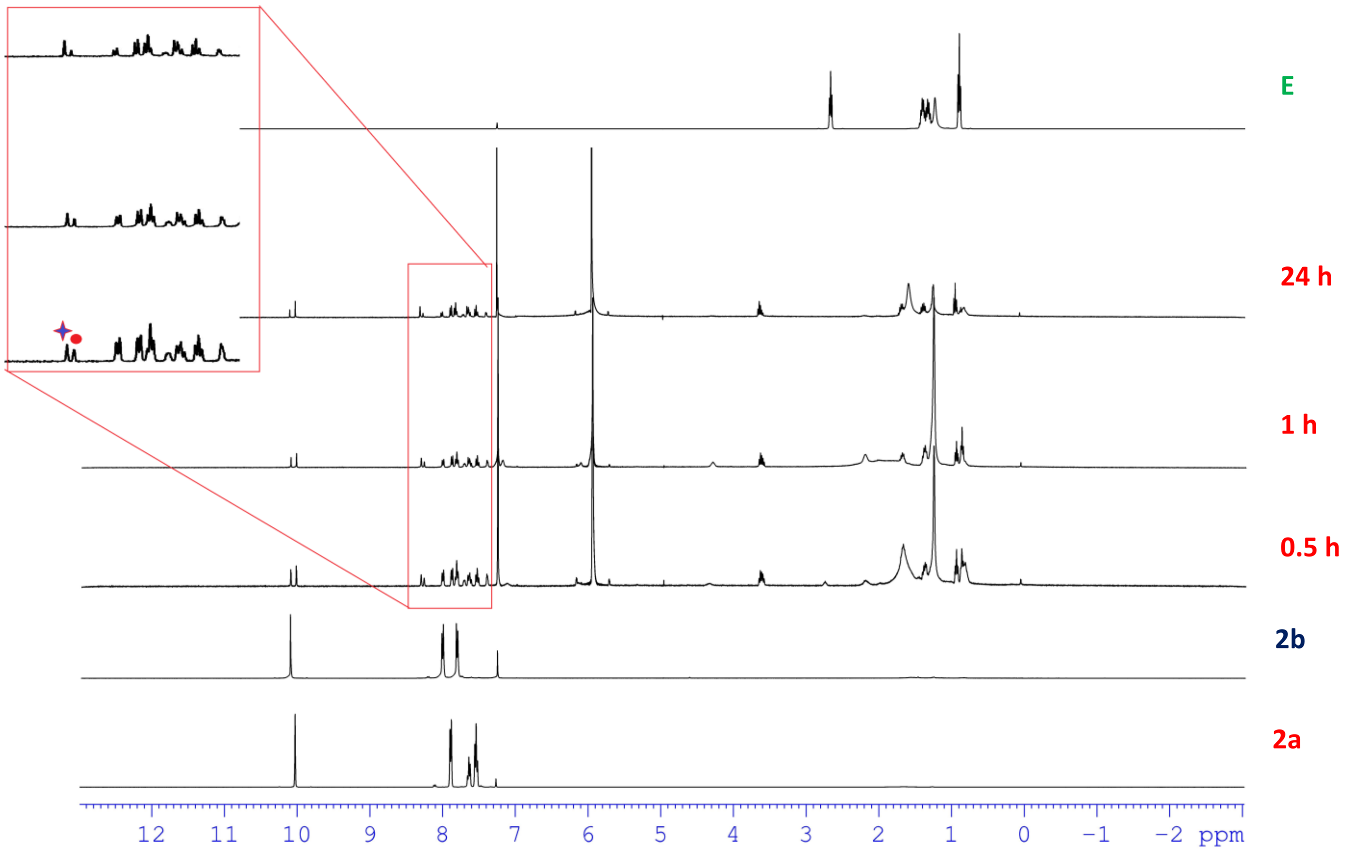

Figure S36 . ^{1}H NMR (400 MHz, $CDCl_3$, 298 K) spectra of an equimolar mixture of **E**, **2a** and **2b** (42.3 mM each, water saturated $CDCl_3$, r.t.) after 0.5, 1 and 24h. (Bottom) ^{1}H NMR spectra of **2a** and **2b**. (Top) ^{1}H NMR spectra of **E**. On the left, in the red panel, a relevant selected region from 7.3 to 8.5 ppm. The signals of free imines **E2a** and **E2b** were assigned in agreement with reported literature values.[16]

Table S9. Time-dependent conversion in imines **E2a** and **E2b** of an equimolar mixture of **2a, 2b** and **E** in presence and in absence of **CR$_6$**.[a]

	in absence of CR$_6$		***in presence of CR$_6$***	
**Time[b] (h)**	**E2a (%)[c]**	**E2b (%)[c]**	**E2a (%)[c,d]**	**E2b (%)[c,d]**
**0.5**	12	13	3	12
**1**	14	26	3	12
**5**	17	40	3	12
**24**	17	40	3	13

[a] Reaction conditions: **2a**, **2b** (0.0423 mmol, 42.3 mM), **E** (0.0423 mmol, 42.3 mM), capsule **CR$_6$** (42.3 mM), water-saturated $CDCl_3$ (1 mL), rt. [b] Time at which an aliquot (30 μL) of the reaction mixture was taken and monitored via ^{1}H-NMR spectrum. [c] Conversion was calculated using TCE as internal standard. [d] Conversion was calculated after addition of DMSO (2 μL) to a reaction aliquot, in order to disaggregate the capsule. Error in ^{1}H-NMR signal integration was ± 5%.

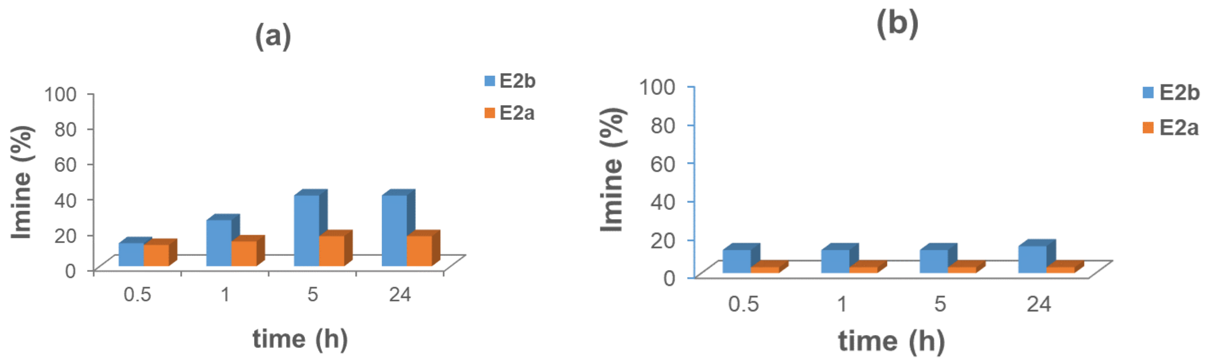

Figure S37. Distribution of imine constituents **E2a** and **E2b** in the DCL, without (a) and with (b) capsule **CR$_6$**

The results in Table S9 (Figure S37 a and b) show clearly that the formation of imines **E2a** and **E2b** starting by *n*-butylamine **E** and aldehydes **2a**/**2b**, is favoured in absence of capsule **CR$_6$**. In fact, in absence of **CR$_6$** the imine **E2a** and **E2b** were obtained in 17 and 40 % of yield respectively after 24 h, while in presence of **CR$_6$** the 3 and 13 % of yields were collected. This is in contrast to the results obtained so far with aromatic amines, in which in presence of **CR$_6$**, the formation of imines was favoured. These results indicate that the capsule depresses the reactivity of *n*-butylamine **E** toward the aldehydes **2a** and **2b**, in accord with the data previously reported by Tiefenbacher in reference 2, in which the authors stated: "*Addition of 0.5 equiv of bases with* pKa *values ranging from 11−6.1 to a solution of capsule in water-saturated CDCl$_3$ (3.3 mM) resulted in approximately 80% of protonation…*". In accord with these results,[2] the *n*-butylammonium cation is encapsuled inside **CR$_6$** as stable ammonium cation, and at this extent of protonation (80 % about) the

percentage of free neutral *n*-butylamine is low and the imine formation is depressed. Tiefenbacher and coworkers, again stated:

"*Beginning with pyridine* (p*K*a *=5.2), we observed a lower degree of protonation (53 ± 1%),* ***which is further decreased to 23 ± 2% in the case of aniline (pKa = 4.6). Amines of lower basicity did not show any degree of protonation as evidenced by*** [1]***H NMR spectroscopy*.**" On the basis of these observations, we can explain the difference in reactivity between the aromatic and aliphatic amines compared to the formation of imines in the confined space of the capsule. The *p*-chloroaniline, that shows a p*K*a of 3.8, is not protonated by **CR_6** as reported in Figure 4 of reference 2 and consequently shows a remarkable reactivity in the confined space inside **CR_6** in presence of aldehydes, while the *n*-butylammine is protonated inside **CR_6** and its reactivity toward the aldehydes is depressed.

In order to corroborate these results, we performed a competition experiment in which benzaldehyde **2a**, *p*-chloroaniline **A** and *n*-butylamine **E** were mixed in 1/1/1 ratio (42.3 mM) in water saturated $CDCl_3$. As reported in Table S10 and Figures S38-S40, in absence of capsule **CR_6** the *n*-butylamine-derived imine **E2a** was formed in 50 % of yield while the aniline-derived imine **A2a** was obtained in 25 % of yield. Differently, in presence of capsule **CR_6**, the selectivity order was reversed to 25/15 in favour of the aniline-derived imine **A2a.**

In conclusion this is an interesting example of substrate selectivity of the hexameric resorcinarene capsule, that is able to discriminate between aromatic and aliphatic amines. In details, the capsule is able to encapsulate the scarcely basic *p*-chloroaniline in a neutral form promoting the formation of the corresponding imine in presence of aldehyde. Differently, when more basic *n*-butylammine is used, the corresponding ammonium form is obtained after protonation inside the capsule and is stabilized by cation···π interactions, in this way in the presence of aldehydes, the formation of imine is depressed.

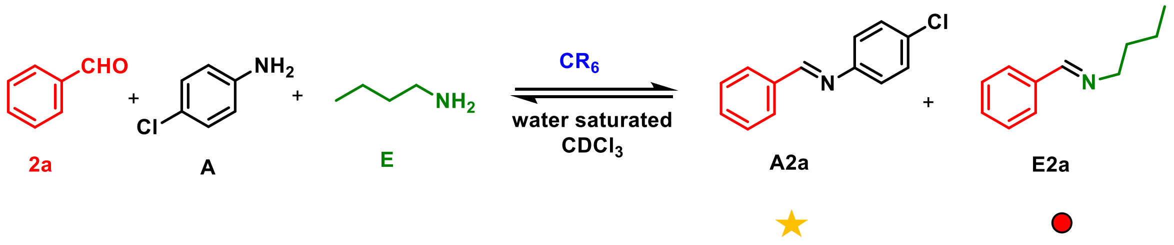

Scheme S13. Synthesis of **A2a** and **E2a** in presence of capsule **CR_6**.

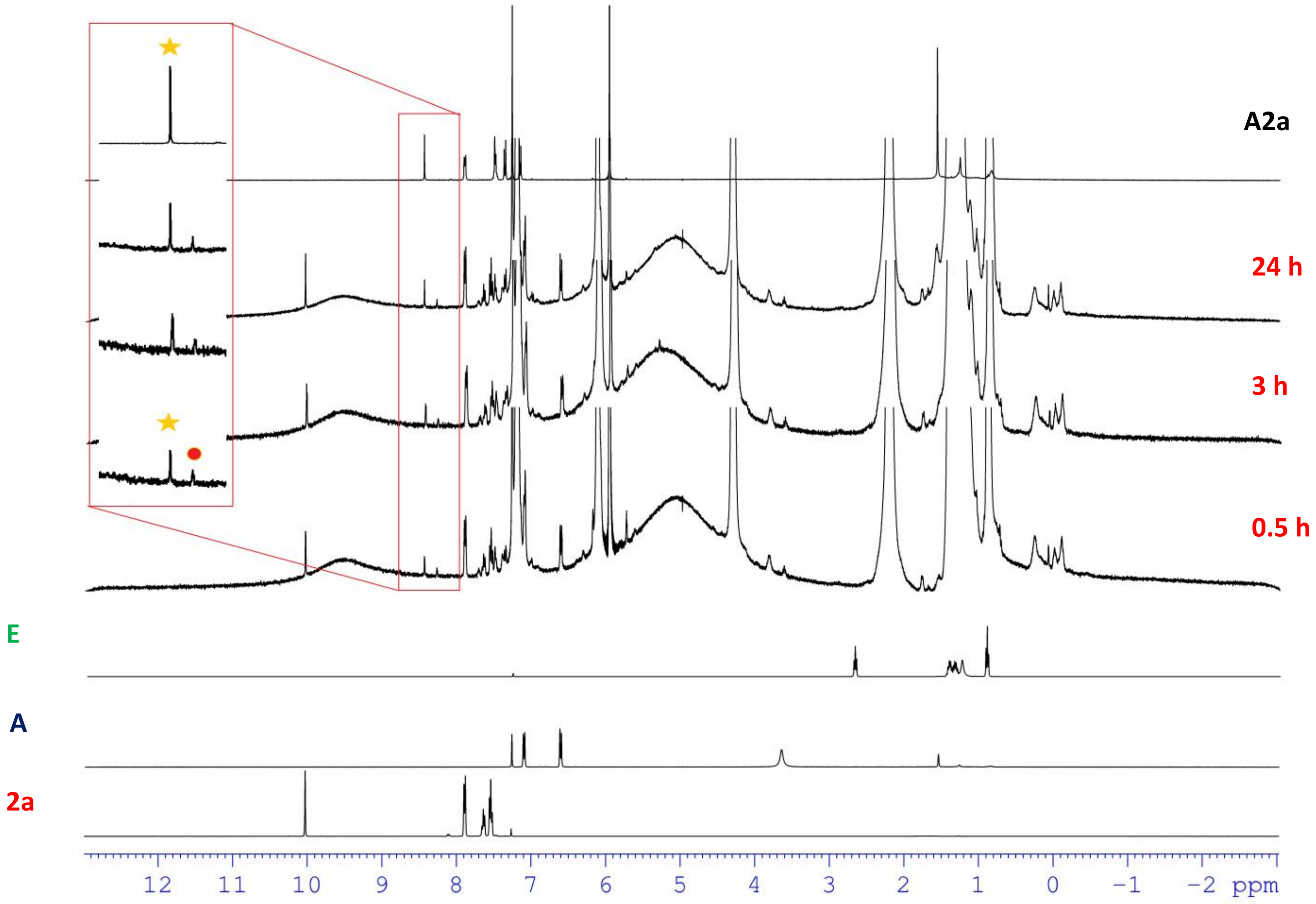

Figure S38. ^{1}H NMR (400 MHz, $CDCl_3$, 298 K) spectra of an equimolar mixture of **E, A, 2a** and **CR_6** (42.3 mM each, water saturated $CDCl_3$, r.t.) after 0.5, 3 and 24. (Bottom) ^{1}H NMR spectra of **2a, A** and **E** . (Top) ^{1}H NMR spectra of **A2a**. On the left, in the red panel, a selected region from -8.0 to 8.6 ppm. The signals of free imines **E2a** were assigned in agreement with reported literature values.[16]

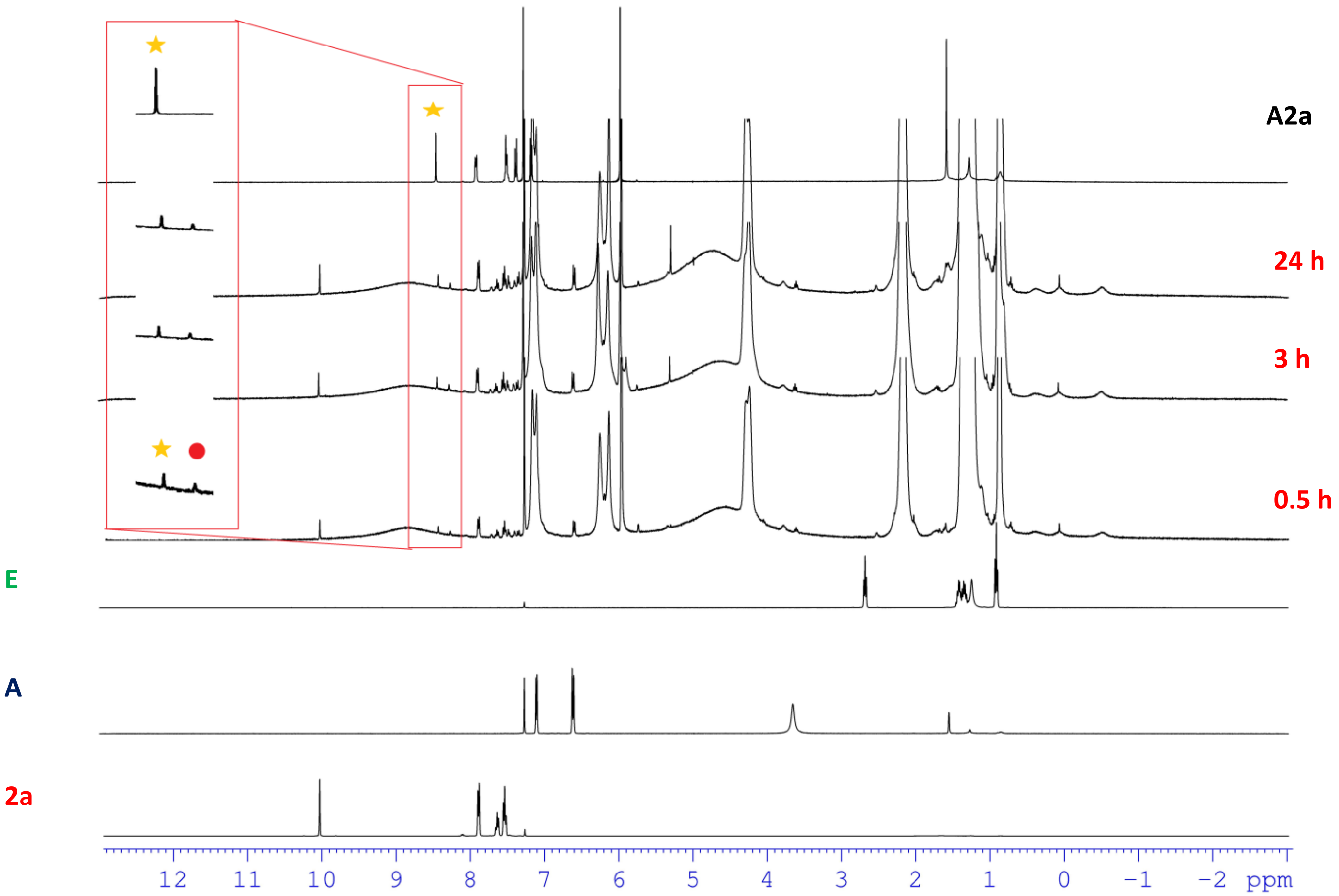

Figure S39. ^{1}H NMR (400 MHz, $CDCl_3$, 298 K) spectra of an equimolar mixture of **A, E, 2a** and **CR$_6$** (42.3 mM each, water saturated $CDCl_3$, r.t.) after 0.5, 3 and 24h. (Bottom) ^{1}H NMR spectra of **2a**, **A** and **E**. (Top) ^{1}H NMR spectra of **A2a**. On the left, in the red panel, a selected region from 8.0 to 8.6 ppm. The spectra are recorded after addition of DMSO (2 µL) to a reaction aliquot, in order to disaggregate the capsule. The signals of free imines **E2a** were assigned in agreement with reported literature values.[16]

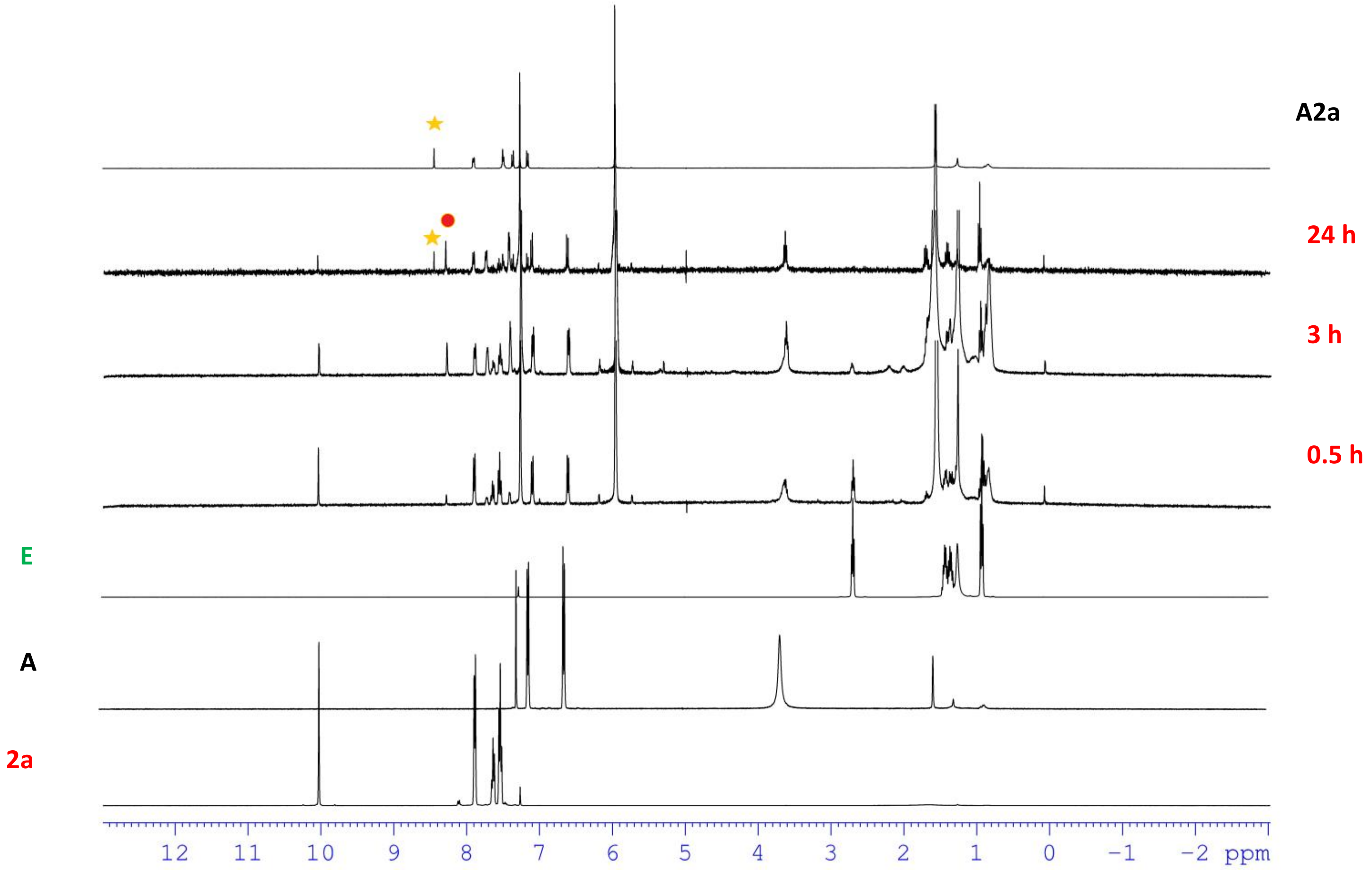

Figure S40. ^{1}H NMR (400 MHz, $CDCl_3$, 298 K) spectra of an equimolar mixture of **A**, **E** and **2a** (42.3 mM each, water saturated $CDCl_3$, r.t.) after 0.5, 1 and 24h. (Bottom) ^{1}H NMR spectra of **2a, A** and **E**. (Top) ^{1}H NMR spectra of **A2a**. The signals of free imines **E2a** were assigned in agreement with reported literature values.[16]

Table S10. Time-dependent conversion in imines **E2a** and **A2a** of an equimolar mixture of **A, E** and **2a** in presence and in absence of **CR_6**.[a]

	in absence of CR_6		*in presence of CR_6*	
**Time[b] (h)**	**A2a (%)[c]**	**E2a (%)[c]**	**A2a (%)[c,d]**	**E2a (%)[c,d]**
**0.5**	-[e]	18	18	14
**3**	-[e]	57	25	15
**24**	25	50	25	15

[a] Reaction conditions: **A**, **E** (0.0423 mmol, 42.3 mM), **2a** (0.0423 mmol, 42.3 mM), capsule **CR_6** (42.3 mM), water-saturated $CDCl_3$ (1 mL), rt. [b] Time at which an aliquot (30 μL) of the reaction mixture was taken and monitored via ^{1}H-NMR spectrum. [c] Conversion was calculated using TCE as internal standard. [d] Conversion was calculated after addition of DMSO (2 μL) to a reaction aliquot, in order to disaggregate the capsule. [e]Below the limit of detection . Error in ^{1}H-NMR signal integration was ± 5%. The signals of free imines **E2a** were assigned in agreement with reported literature values.[16]

6.9 Competitive reaction between equivalent amounts of 2a, 2b, A and B with and without capsule CR_6 (Figure 15 in the main text).

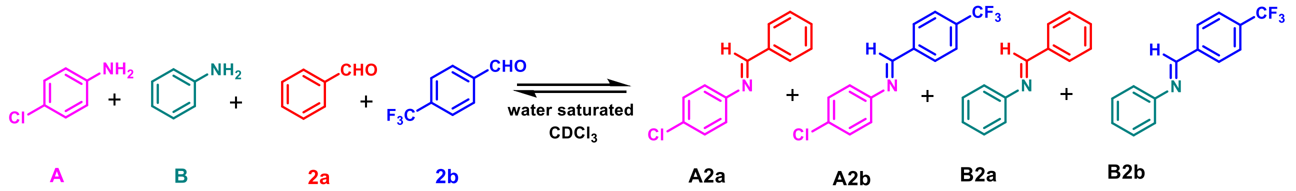

Scheme S14. Dynamic library of four constituents.

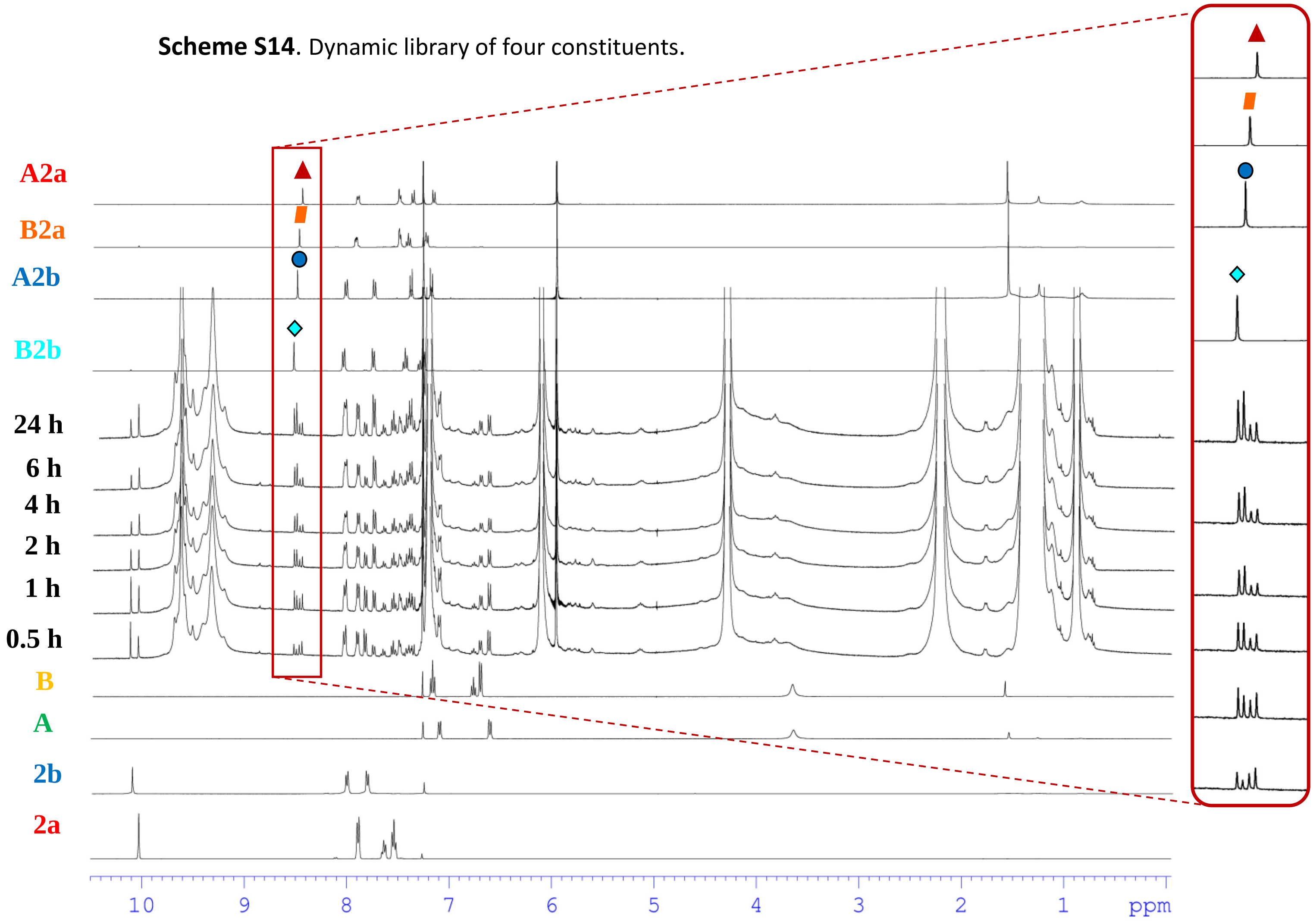

Figure S41. ^{1}H NMR (400 MHz, $CDCl_3$, 298 K) spectra of an equimolar mixture of **A, B, 2a, 2b** and **CR_6** (42.3 mM each) in water saturated $CDCl_3$ (1 mL, r.t.) after 0.5, 1, 2, 4, 6 and 24 h. (Bottom) ^{1}H NMR spectra of **2a, 2b** and **A** and **B**. (Top) ^{1}H NMR spectra of **A2a**, **A2b, B2a** and **B2b**. On the right, in the red panel, a selected region from 8.4 to 8.6 ppm.

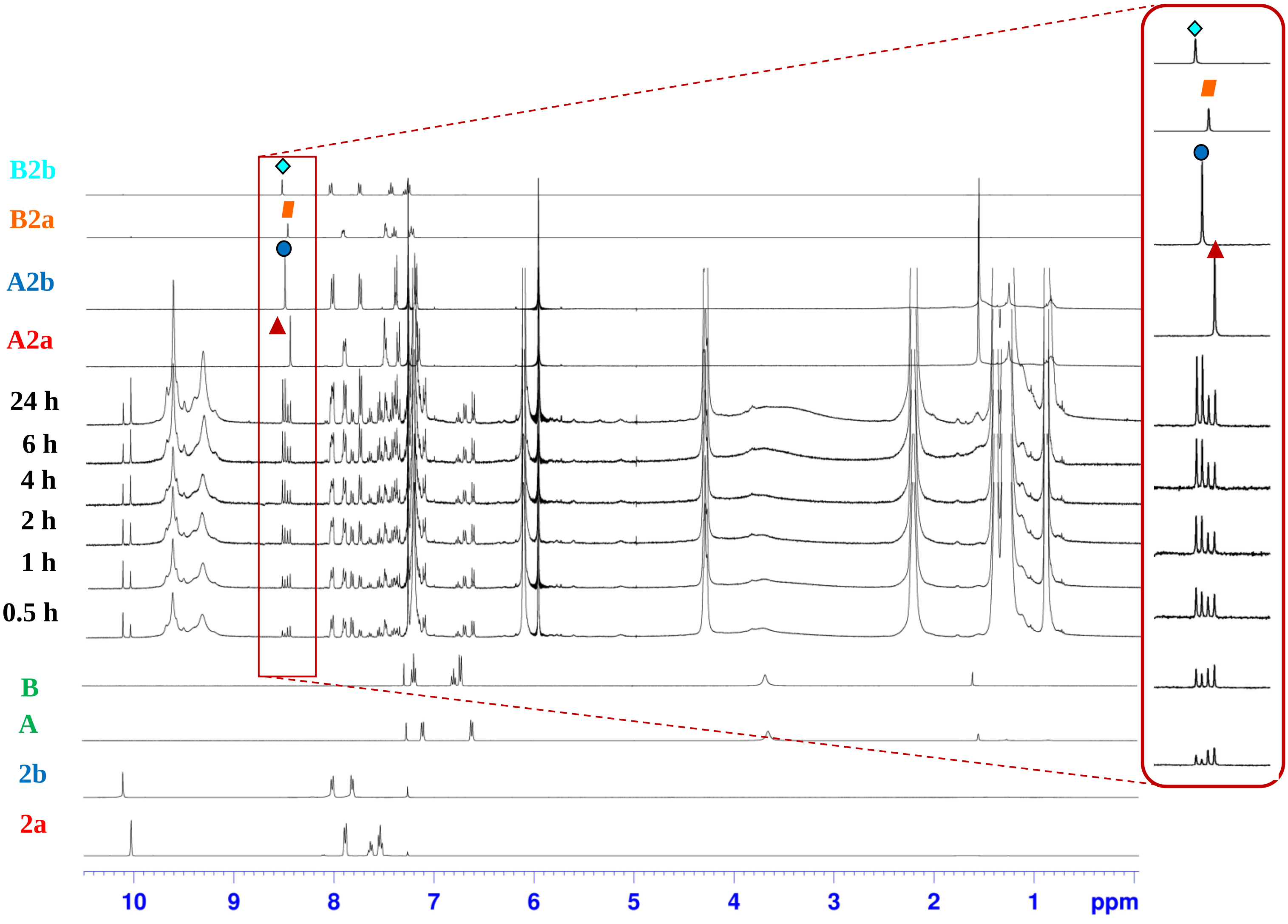

Figure S42. ^{1}H NMR (400 MHz, $CDCl_3$, 298 K) spectra of an equimolar mixture of **A, B, 2a, 2b** (42.3 mM each) and $\mathbf{CR_6}$ (21.1 mM) in water saturated $CDCl_3$ (1 mL, r.t.) after 0.5, 1, 2, 4, 6 and 24 h. (Bottom) ^{1}H NMR spectra of **2a, 2b** and **A** and **B**. (Top) ^{1}H NMR spectra of **A2a**, **A2b, B2a** and **B2b**. On the right, in the red panel, a selected region from 8.4 to 8.6 ppm.

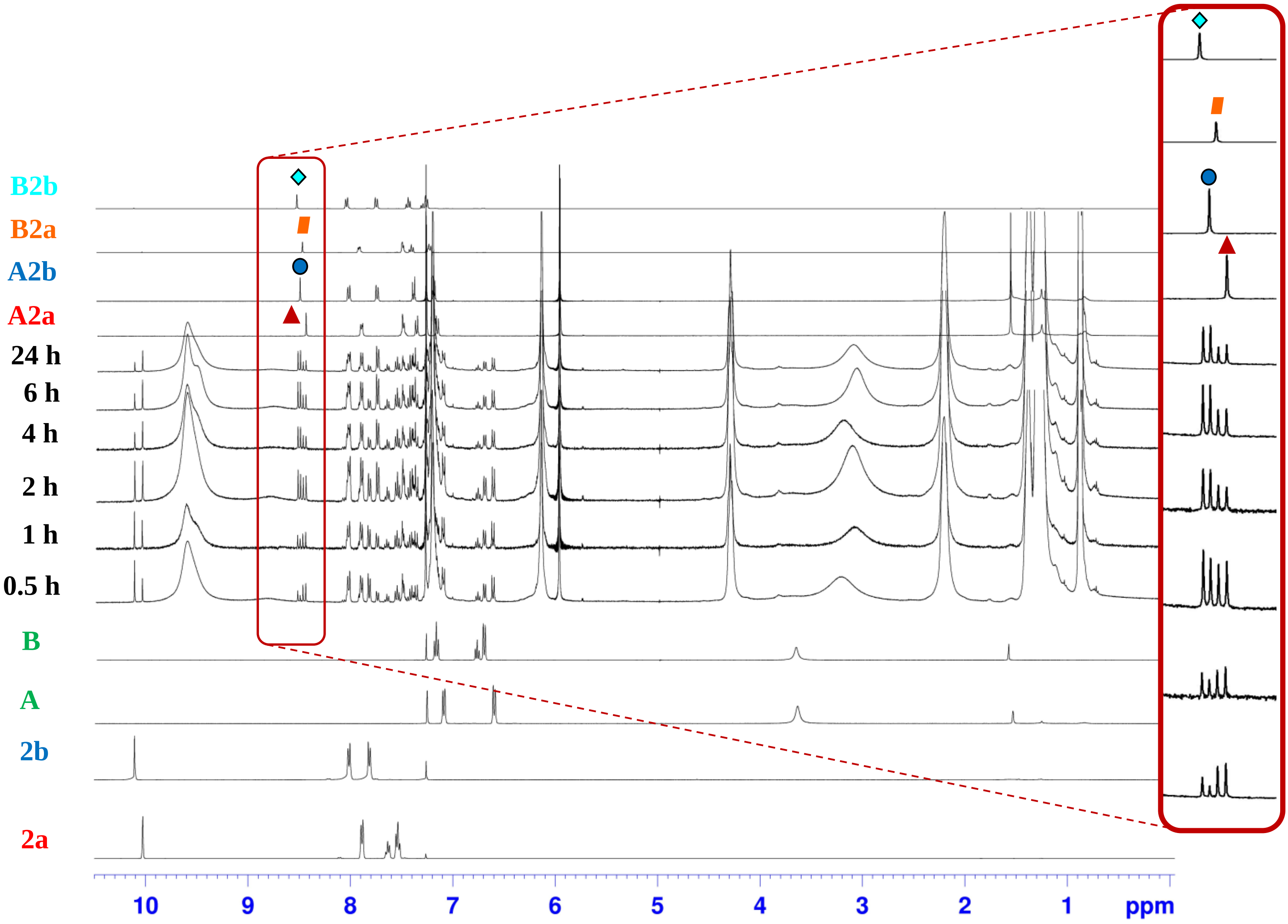

Figure S43. ^{1}H NMR (400 MHz, $CDCl_3$, 298 K) spectra of an equimolar mixture of **A, B, 2a, 2b** and **CR_6** (42.3 mM each) in water saturated $CDCl_3$ (1 mL, r.t.) after 0.5, 1, 2, 4, 6 and 24 h. The spectra are recorded after addition of DMSO (2 μL) to a reaction aliquot, in order to disaggregate the capsule. (Bottom) ^{1}H NMR spectra of **2a, 2b** and **A** and **B**. (Top) ^{1}H NMR spectra of **A2a**, **A2b, B2a** and **B2b**. On the right, in the red panel, a selected region from 8.4 to 8.6 ppm.

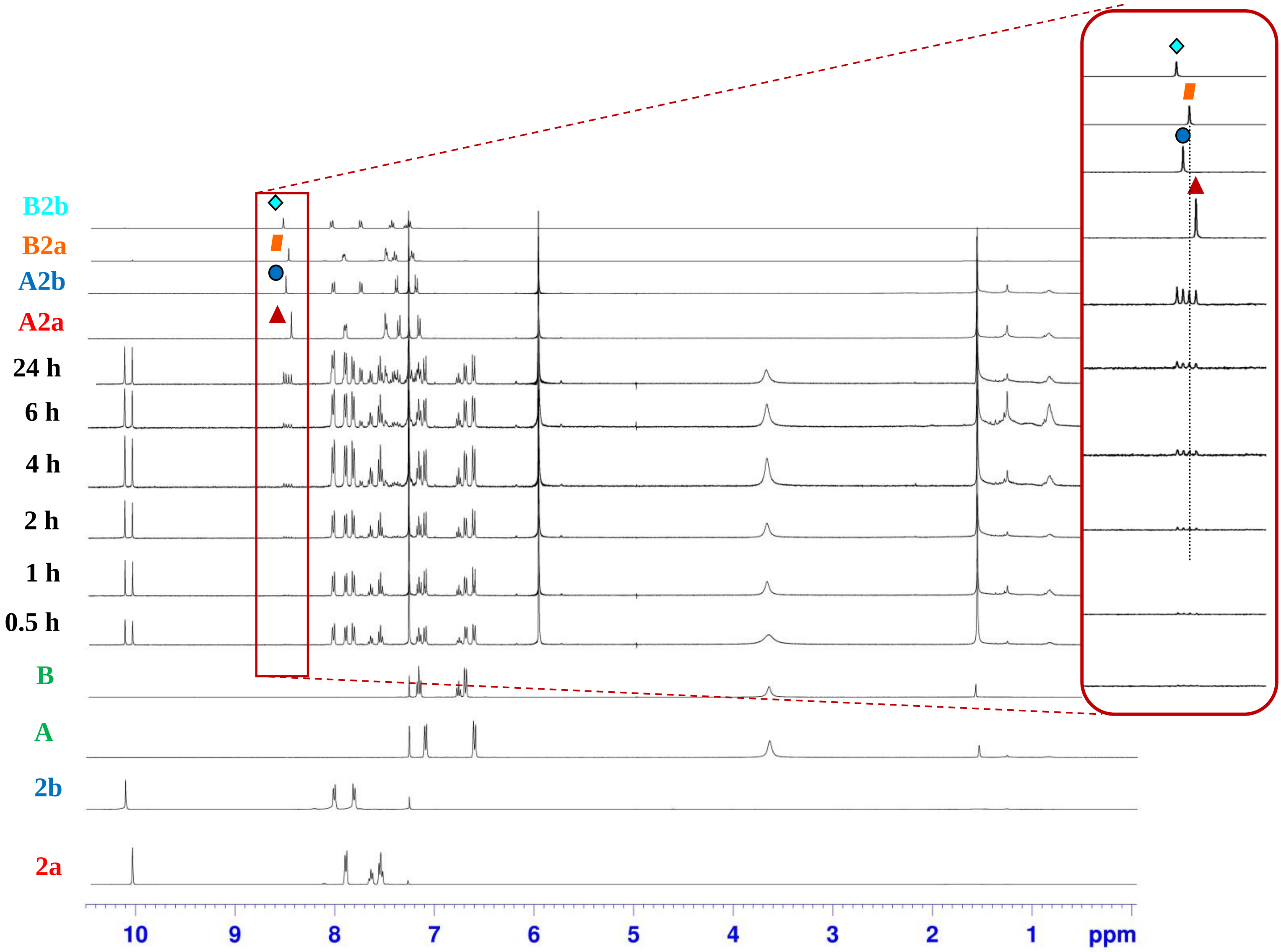

Figure S44. ^{1}H NMR (400 MHz, $CDCl_3$, 298 K) spectra of an equimolar mixture of **A, B, 2a, 2b** (42.3 mM each) in water saturated $CDCl_3$ (1 mL, r.t.) after 0.5, 1, 2, 4, 6 and 24 h. (Bottom) ^{1}H NMR spectra of **2a, 2b** and **A** and **B**. (Top) ^{1}H NMR spectra of **A2a**, **A2b, B2a** and **B2b**. On the right, in the red panel, a selected region from 8.4 to 8.6 ppm.

Table S11. Time-dependent conversion in imines **A2a, A2b, B2a** and **B2b** of an equimolar mixture of **2a, 2b, A** and **B** in presence and in absence of $\mathbf{CR_6}$ (Figure 15 in the main text).[a]

	in absence of CR_6				*in presence of* CR_6			
**Time[b] (h)**	**A2a(%)[c]**	**A2b (%)[c]**	**B2a (%)[c]**	**B2b(%)[c]**	**A2a (%)[d]**	**A2b (%)[d]**	**B2a(%)[d]**	**B2b(%)[d]**
**0.5**	-[e]	-[e]	-[e]	-[e]	25	11	17	20
**1**	-[e]	-[e]	-[e]	-[e]	28	24	19	34
**2**	3	3	3	6	24	36	16	36
**4**	7	7	7	11	21	47	14	42
**6**	5	7	4	9	16	48	14	35
**24**	17	18	14	19	15	50	13	35
**48**	17	18	14	19	15	50	13	35

[a] Reaction conditions: **2a**, **2b** (0.0423 mmol, 42.3 mM), **A, B** (0.0423 mmol, 42.3 mM), capsule $\mathbf{CR_6}$ (42.3 mM), water-saturated $CDCl_3$ (1 mL), rt. [b] Time at which an aliquot (30 μL) of the reaction mixture was taken and monitored via ^{1}H-NMR spectrum. [c] Conversion was calculated using TCE as internal standard. [d] Conversion was calculated after addition of DMSO (2 μL) to a reaction aliquot, in order to disaggregate the capsule. [e]Below the limit of detection. Error in ^{1}H-NMR signal integration was ±5%.

Table S12. Time-dependent evolution of imines **A2a, A2b, B2a** and **B2b** generated from equal amounts of **2a, 2b, A** and **B** in presence of **CR_6** (0.5 equiv).[a]

	in presence of CR_6			
**Time[b] (h)**	**A2a (%)[c]**	**A2b (%)[c]**	**B2a(%)[c]**	**B2b(%)[c]**
**0.5**	32	10	28	18
**1**	25	15	27	28
**2**	28	26	24	30
**4**	22	33	23	37
**6**	25	39	24	40
**24**	22	44	20	40
**48**	22	44	20	40

[a] Reaction conditions: **2a**, **2b** (0.0423 mmol, 42.3 mM), **A, B** (0.0423 mmol, 42.3 mM), capsule **CR_6** (0.0211 mmol, 21.1 mM), water-saturated $CDCl_3$ (1 mL), rt. [b] Time at which an aliquot (30 μL) of the reaction mixture was taken and monitored via ^{1}H-NMR spectrum. [c] Conversion was calculated using TCE as internal standard after addition of DMSO (2 μL) to a reaction aliquot, in order to disaggregate the capsule. Error in ^{1}H-NMR signal integration was ±5%.

6.10 Competitive reaction between equivalent amounts of 2b, 2d, C and D with and without capsule CR6 (Figure 16 in the main text).

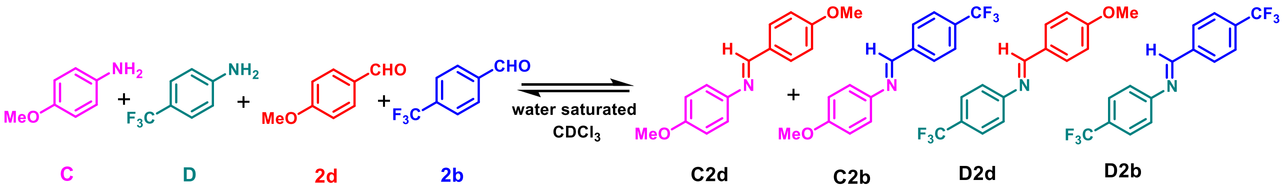

Scheme S15. Dynamic library of four constituents.

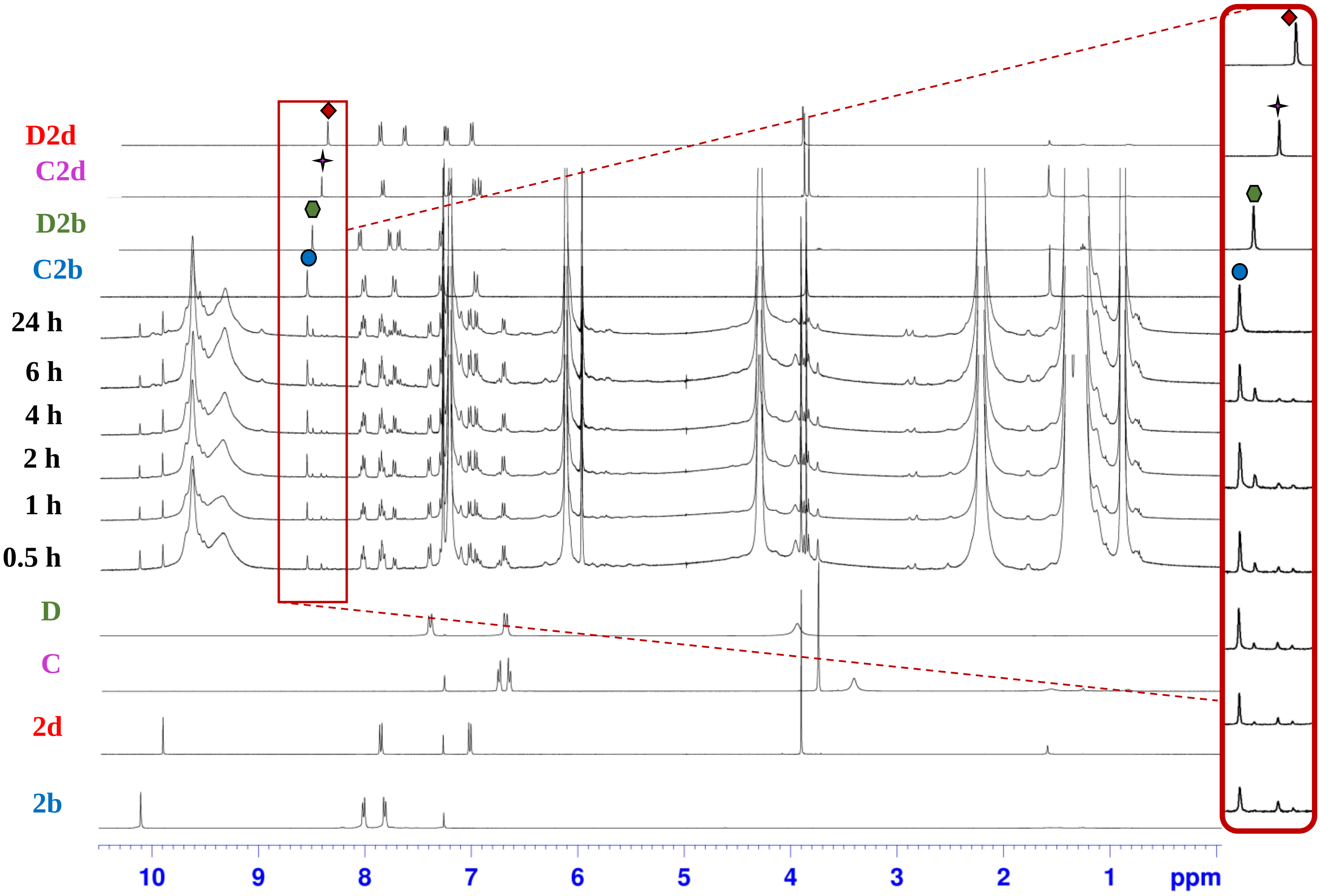

Figure S45. ^{1}H NMR (400 MHz, $CDCl_3$, 298 K) spectra of an equimolar mixture of **C, D, 2b, 2d** and **CR_6** (42.3 mM each) in water saturated $CDCl_3$ (1 mL, r.t.) after 0.5, 1, 2, 4, 6 and 24 h. (Bottom) ^{1}H NMR spectra of **2d, 2b** and **C** and **C**. (Top) ^{1}H NMR spectra of **D2d**, **C2d, D2b** and **C2b**. On the right, in the red panel, a selected region from 8.4 to 8.6 ppm.

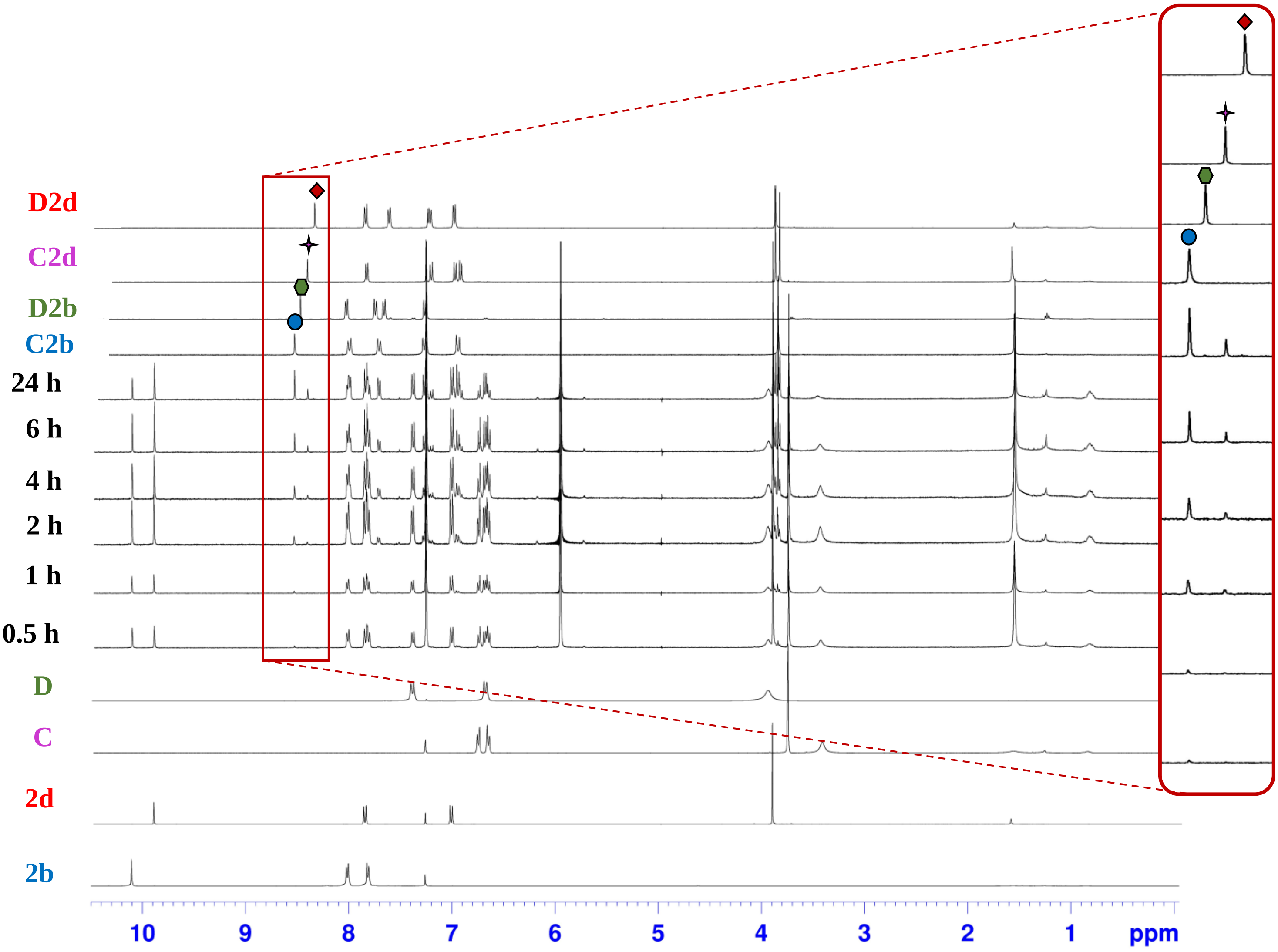

Figure S46. ^{1}H NMR (400 MHz, $CDCl_3$, 298 K) spectra of an equimolar mixture of **C**, **D**, **2b** and **2d** (42.3 mM each, water saturated $CDCl_3$, r.t.) after 0.5, 1, 2, 4, 6 and 24 h. (Bottom) ^{1}H NMR spectra of **2d, 2b** and **C**. (Top) ^{1}H NMR spectra of **D2d**, **C2d, D2b** and **C2b**. On the right, in the red panel, a relevant selected region from 8.4 to 8.6 ppm.

Table S13. Time-dependent conversion in imines **C2b, C2d, D2b** and **D2d** of an equimolar mixture of **2b, 2d, C** and **D** in presence and in absence of $\mathbf{CR_6}$.[a]

	in absence of CR_6				*in presence of CR_6*			
**Time[b] (h)**	**C2b(%)[c]**	**C2d (%)[c]**	**D2b (%)[c]**	**D2d(%)[c]**	**C2b (%)[d]**	**C2d (%)[d]**	**D2b(%)[d]**	**D2d(%)[d]**
**0.5**	4	2	-[e]	-[e]	47	19	-[e]	4
**1**	12	5	-[e]	-[e]	53	22	5	8
**2**	18	5	-[e]	-[e]	64	13	9	6
**4**	21	8	-[e]	-[e]	61	9	14	6
**6**	33	21	-[e]	-[e]	66	7	12	3
**24**	54	34	-[e]	-[e]	66	5	12	5
**48**	54	34	-[e]	-[e]	66	5	12	5

[a] Reaction conditions: **2b**, **2d** (0.0423 mmol, 42.3 mM), **C, D** (0.0423 mmol, 42.3 mM), capsule $\mathbf{CR_6}$ (42.3 mM), water-saturated $CDCl_3$ (1 mL), rt. [b] Time at which an aliquot (30 μL) of the reaction mixture was taken and monitored via ^{1}H-NMR spectrum. [c] Conversion was calculated using TCE as internal standard. [d] Conversion was calculated after addition of DMSO (2 μL) to a reaction aliquot, in order to disaggregate the capsule. [e]Below the limit of detection. Error in ^{1}H-NMR signal integration was ±5%.

7. Competitive uptake experiment

A competition experiment was performed in which aldehydes **2a** and **2b** were in competition to occupy the cavity of capsule **CR6**.

Resorcinarene **1** (281.6 mg, 254.7 µmol, 6 equiv) was weighed in a 4 mL vial and 1mL of water saturated $CDCl_3$ was added. The mixture was warmed at 50 °C until clarification (ca 5 min). To this solution, aldehydes **2a** (0.0423 mmol, 1 equiv) and **2b** (0.0423 mmol, 1 equiv) were added simultaneously and the mixture was kept at 30 °C under stirring for 1 h before being subjected to ^{1}H-NMR spectroscopy. An aliquot portion of this mixture (500 µL) was taken and monitored by ^{1}H NMR spectroscopy. The spectra were recorded before (Figure S47-c) and after addition (Figure S47-d) of DMSO (35 µL).

The uptakes of **2a**/**2b** within **CR6** were measured by quantitative ^{1}H NMR experiments. The quantity of encapsulated aldehyde was obtained by difference between its initial concentration (checked also after disassembly of the hexameric capsule by addition of DMSO) and the concentration of the free aldehyde in solution. The ^{1}H NMR signals of the free aldehyde was integrated with respect to the signal of TCE (5.97 ppm, 2H, 0.019 mmol).

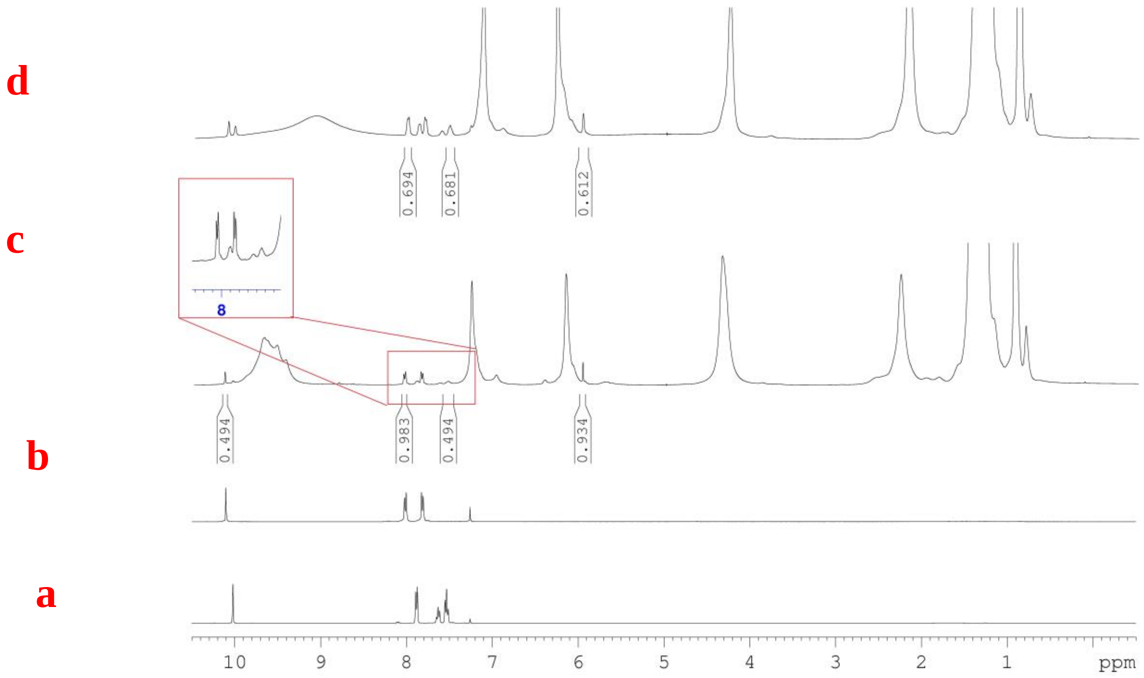

Figure S47. ^{1}H NMR (400 MHz, $CDCl_3$, 298 K) spectra of : **a**) isolated **2a**; **b**) isolated **2b;** **c**) an equimolar mixture of **2a, 2b** and **CR6** (42.3 mM each, water saturated $CDCl_3$, r.t.) and **d**) an equimolar mixture of **2a, 2b** and **CR6** (42.3 mM each, water saturated $CDCl_3$, r.t.) after addition of DMSO.

8. Proofs of the encapsulation of 2a

Resorcinarene **1** (281.6 mg, 254.7 μmol, 6 equiv) was weighed in a 4 mL vial and 1.1 mL of water saturated $CDCl_3$ was added. The mixture was warmed at 50 °C until clarification (ca 5 min). To this solution aldehydes **2a** (0.0423 mmol, 1 equiv) was added and the mixture was kept at 30 °C under stirring for 1 h before being subjected to NMR spectroscopy. An aliquot portion of the mixture prepared (500 μL) was taken and subjected to different NMR experiments.

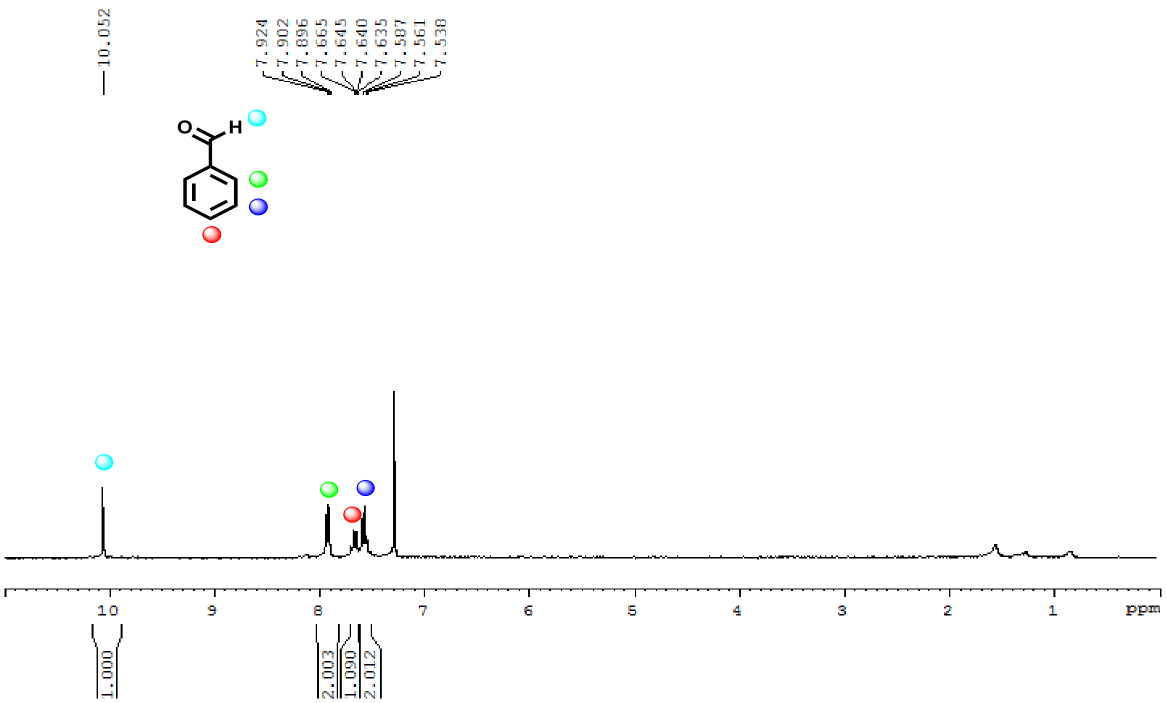

Figure S48. ^{1}H NMR spectrum (400 MHz, $CDCl_3$, 298 K) of benzaldehyde **2a**.

The amount of encapsulated aldehyde **2a** was calculated by the integration of proton signal of free aldehyde at 7.89 ppm (2H). In particular, the equation ($[G]_0 - [G]_t$)/ $[G]_0$ (as reported by Tiefenbacher et al. in *J. Am. Chem. Soc.* **2017,** *139*, 11482-11492) was used to determine the encapsulation degree of **2a**. The terms $[G]_0$ indicates the total concentration of **2a** and $[G]_t$ the remaining free **2a** after the sample was equilibrated for t = 1h.

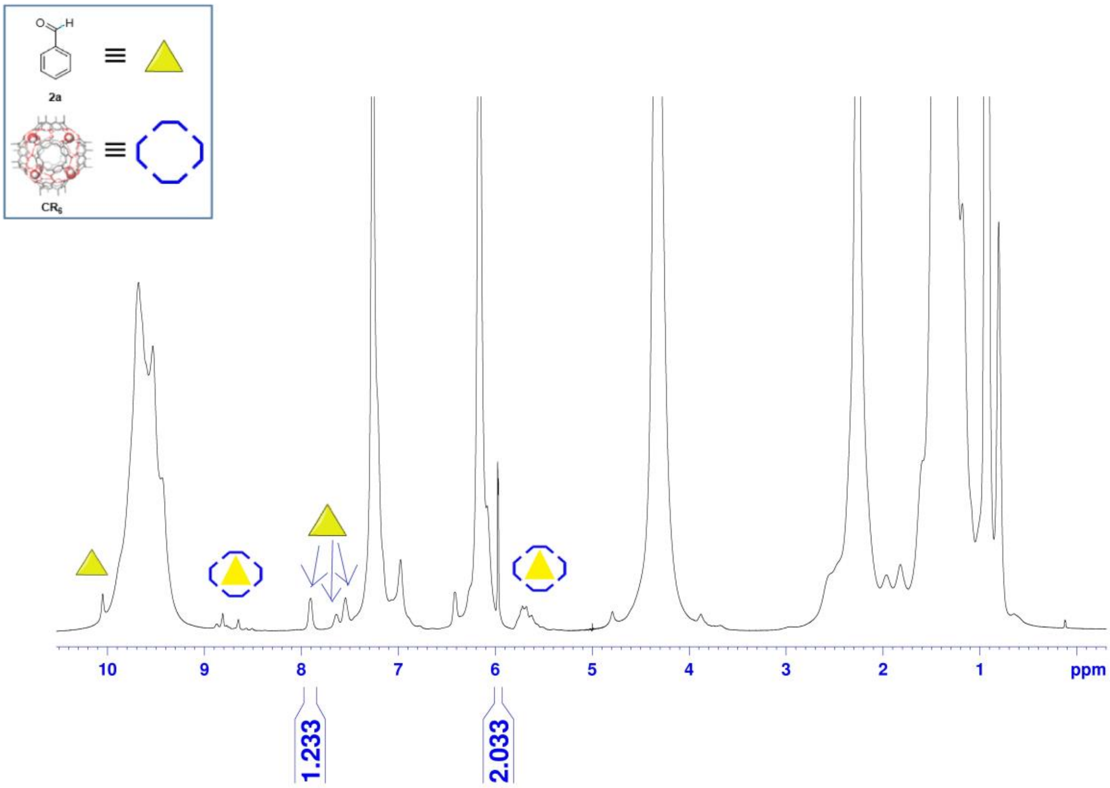

Figure S49. ^{1}H NMR spectrum (400 MHz, $CDCl_3$, 298 K) of the mixture of benzaldehyde **2a** and **CR_6** (42.3 mM of each component). The signal of TCE (5.97 ppm, 2H) used as internal standard.

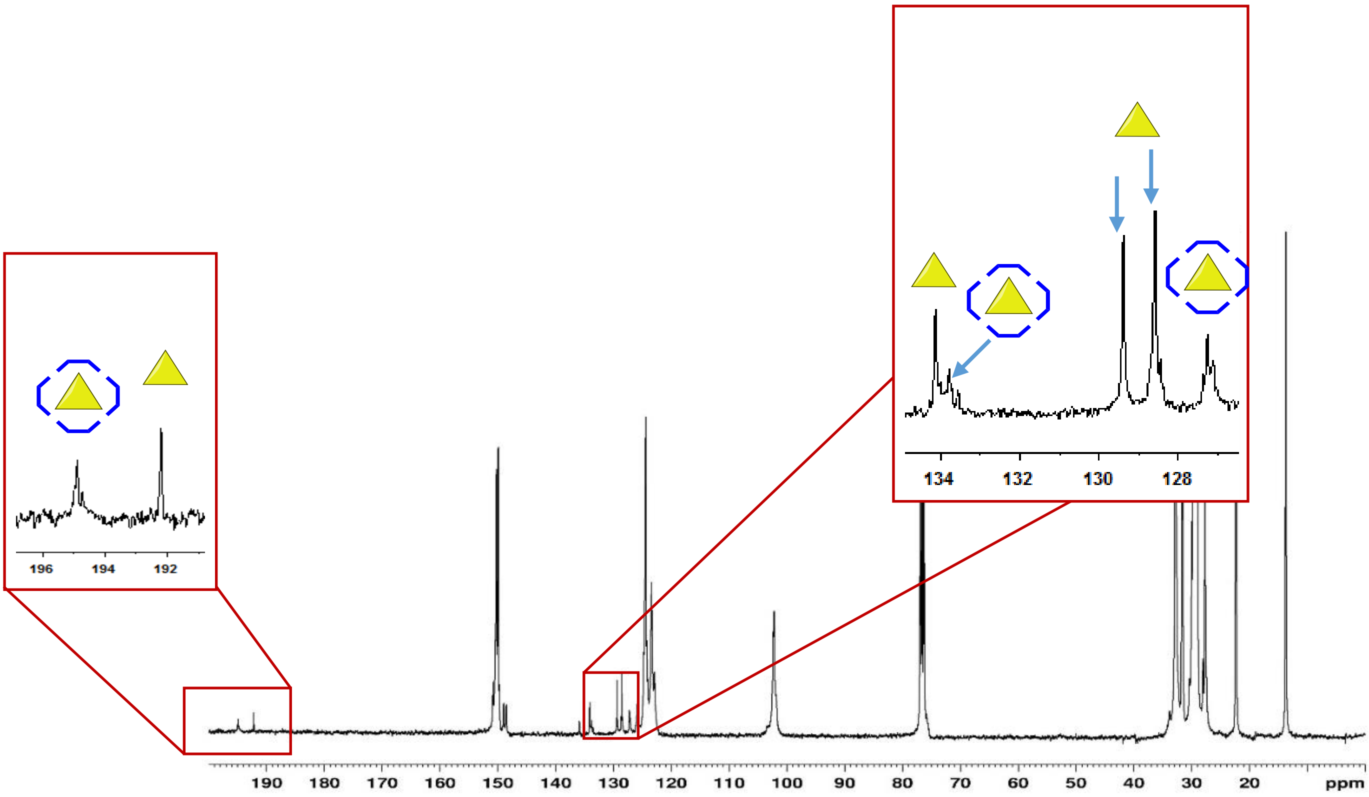

Figure S50. ^{13}C NMR spectrum (100 MHz, $CDCl_3$, 298 K) of the mixture of benzaldehyde **2a** and **CR_6**, 42.3 mM each component.

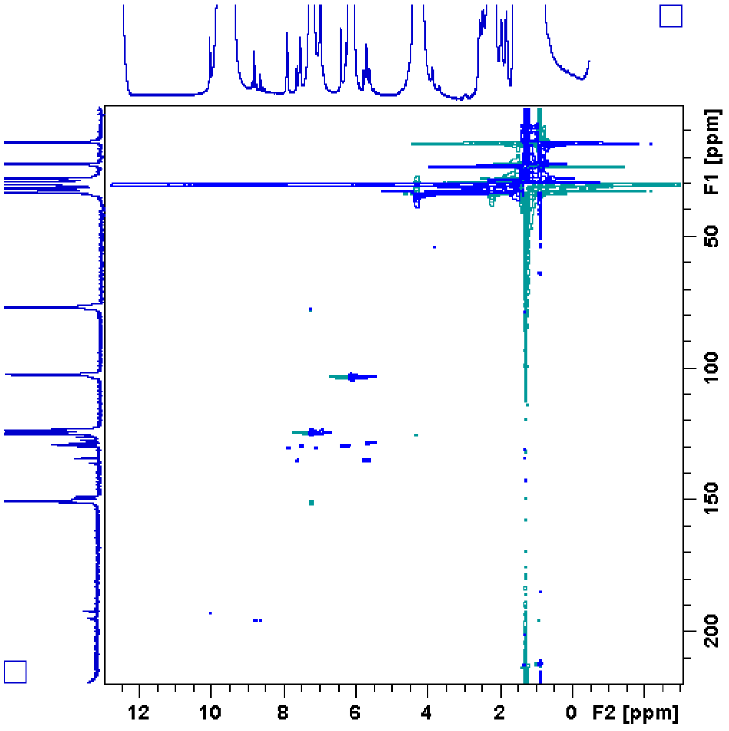

Figure S51. HSQC NMR spectrum (400 MHz, $CDCl_3$, 298 K) of the mixture of benzaldehyde **2a** and capsule **CR$_6$**, 42.3 mM each component.

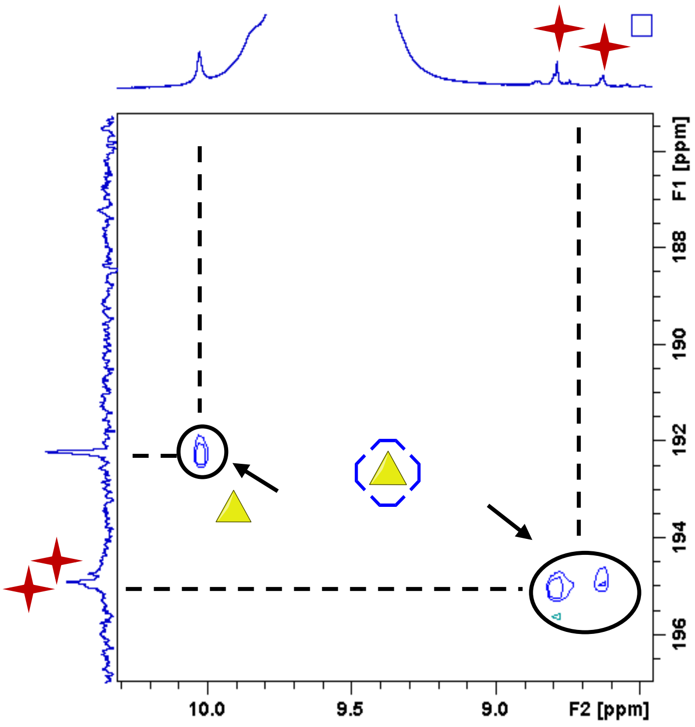

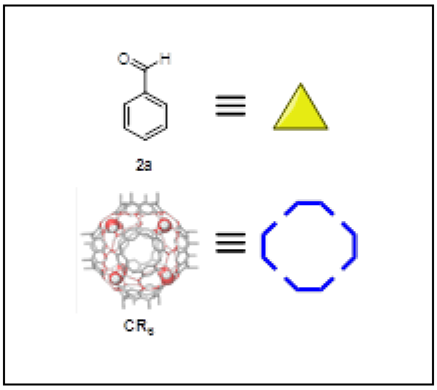

Figure S52. Selected region of the HSQC NMR spectrum in Figure S51. ^{1}J correlations between hydrogen and carbon of the aldehyde group of **2a** attributable to free and encapsulated **2a**. The ^{1} H NMR signals of the encapsulated aldehyde **2a** are very complex, and in accord with the data previously reported by Cohen[13] and by us,[14,15] which indicates that the molecules of **2a** are encapsulated in slightly different nanocontainers.

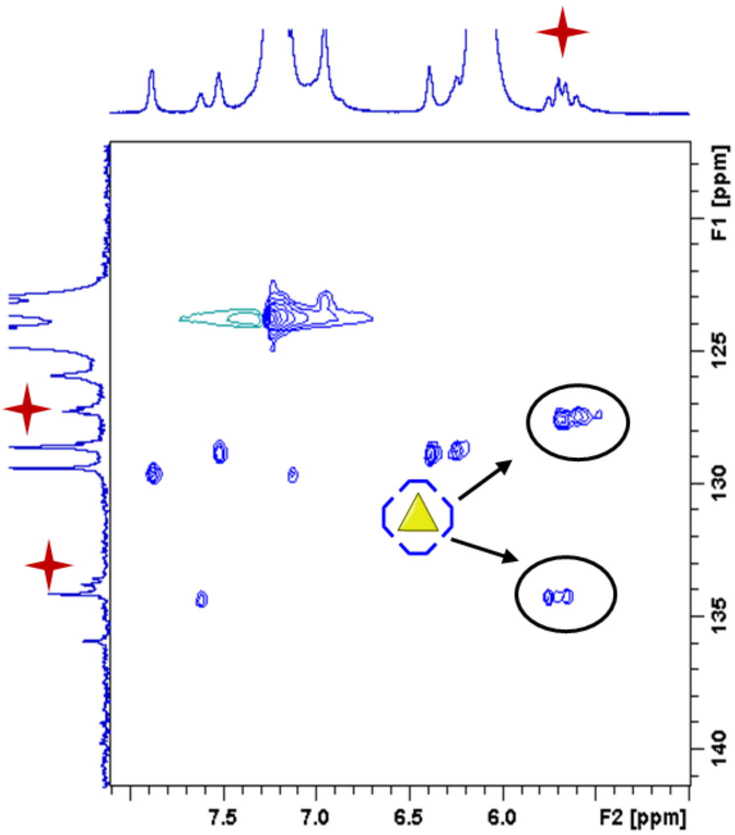

Figure S53. Selected region of the HSQC NMR spectrum in Figure S51. ^1J correlations attributable to the aromatic protons of the benzaldehyde **2a** inside the nanocontainer **CR$_6$** are marked[11,12]

DOSY experiment

DOSY experiments were performed on a Bruker Avance-600 spectrometer equipped with 5 mm PABBO BB|19F-1H\D Z-GRD Z114607/0109. The standard Bruker pulse program, ledbpgp2s, employing a double stimulated echo sequence and LED, bipolar gradient pulses for diffusion, and two spoil gradients were utilized. Diffusion times were 150 ms, eddy current delay was 5 ms, gradient recovery delays were 0.2 ms and gradient pulse was 1400 ms. Individual rows of the quasi-2D diffusion databases were phased and baseline corrected.

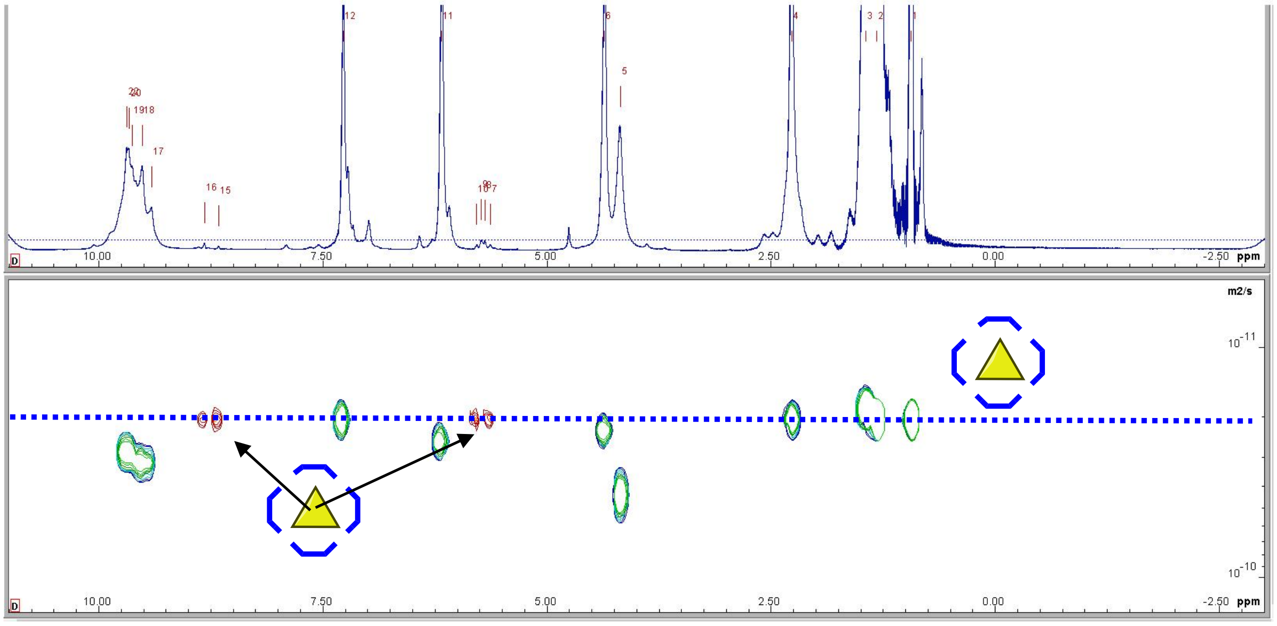

Figure S54. DOSY NMR (600 MHz, $CDCl_3$, 298 K) of the mixture of benzaldehyde **2a** and capsule **CR$_6$**, 42.3 mM of each component.

As indicated in Figure S54, the signals pattern associated to the benzaldehyde **2a** inside the resorcinarene hexameric capsule **CR$_6$** are aligned with the capsule diffusion coefficient.

9. Proofs of the encapsulation of 2b

Resorcinarene **1** (281.6 mg, 254.7 µmol) was weighed in a 4 mL vial and 1 mL of $CDCl_3$ was added. The mixture was warmed at 50 °C until clarification (ca 5 min). To this solution aldehyde **2b** (147.3 mg, 0.846 mmol) was added and the mixture was kept at 30 °C under stirring for 1 h before being subjected to NMR spectroscopy. An aliquot portion of the mixture prepared (500 µL) was taken and subjected to NMR experiments.

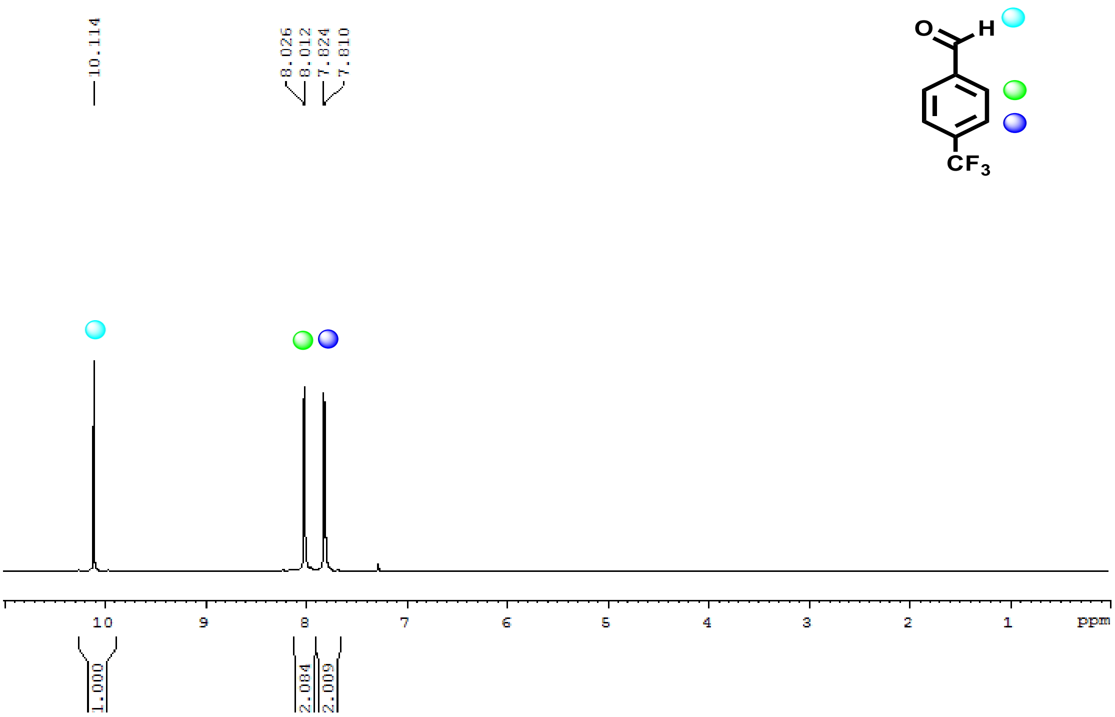

Figure S55. ^1H NMR spectrum (400 MHz, $CDCl_3$, 298 K) of 4-(trifluoromethyl)benzaldehyde **2b**.

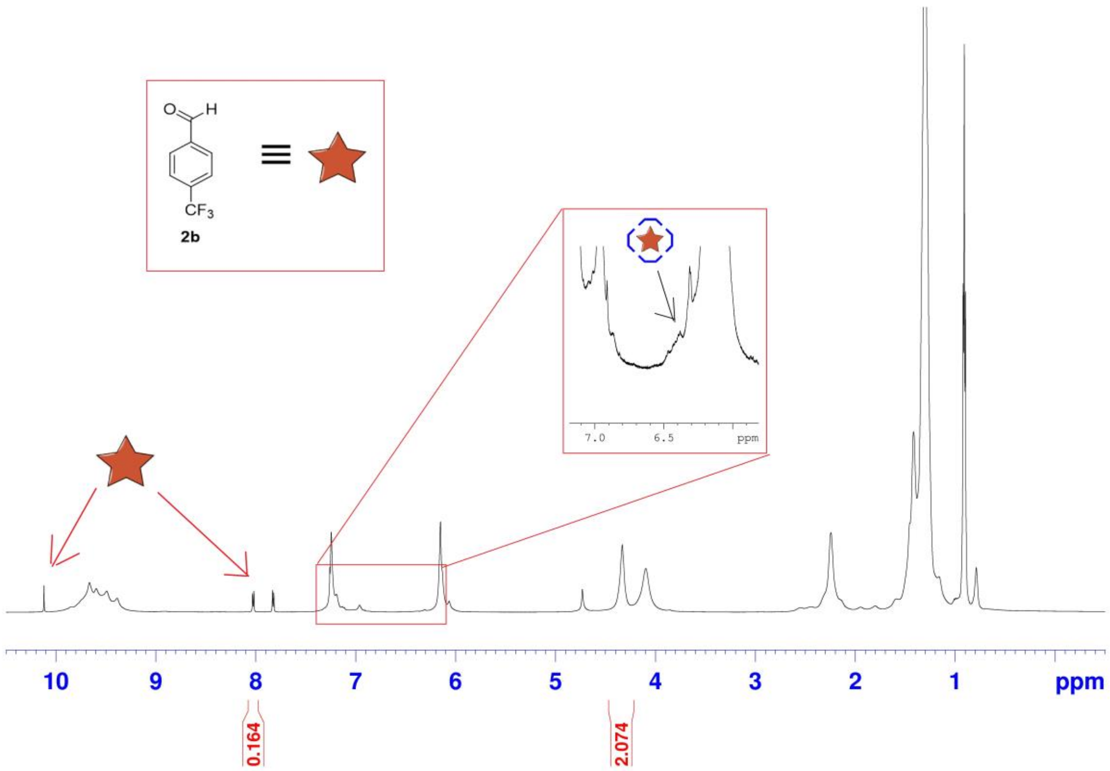

Figure S56. ^{1}H NMR spectrum (400 MHz, $CDCl_3$, 298 K) of the mixture of **2b** (42.3 mM) and **CR_6** (42.3 mM). The methine signal of CR_6 at 4.30 ppm (24H) used as internal standard

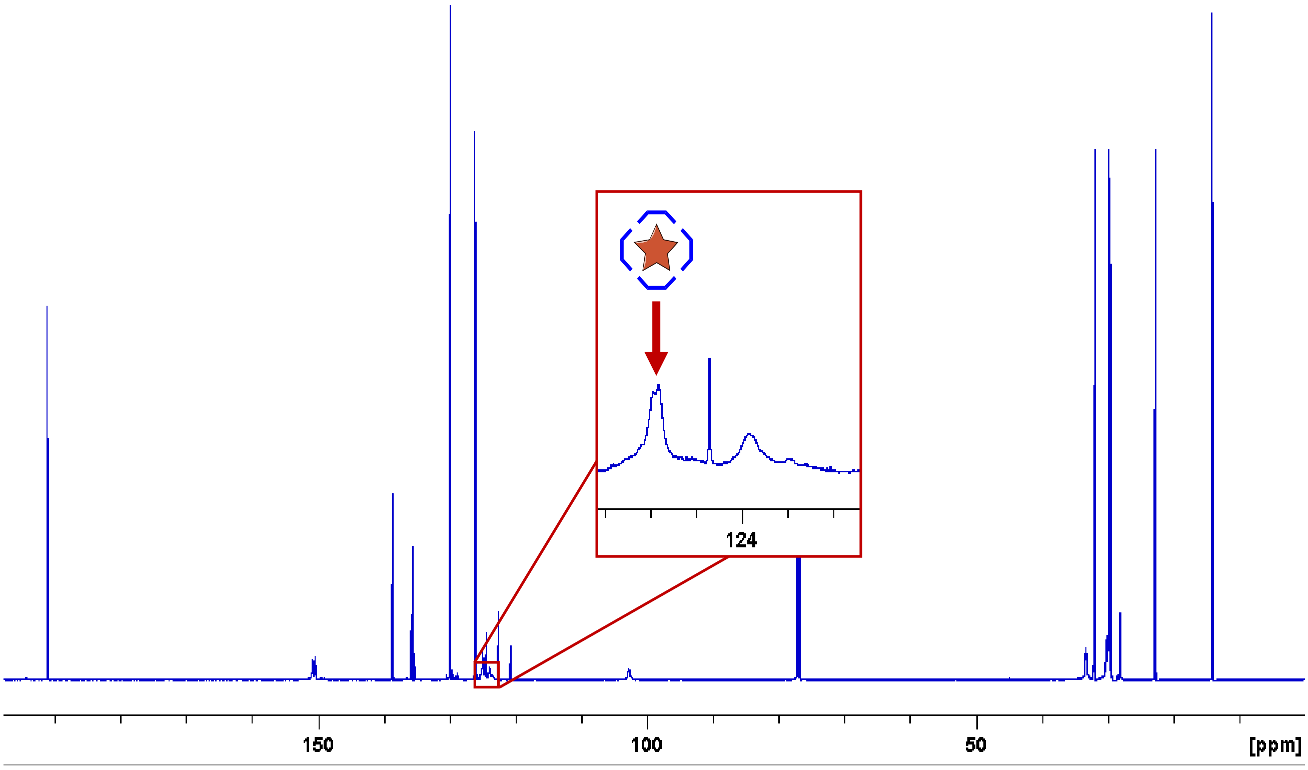

Figure S57. ^{13}C NMR spectrum (100 MHz, $CDCl_3$, 298 K) of the mixture of **2b** (0.846 mmol) and **CR_6** (0.0423 mmol).

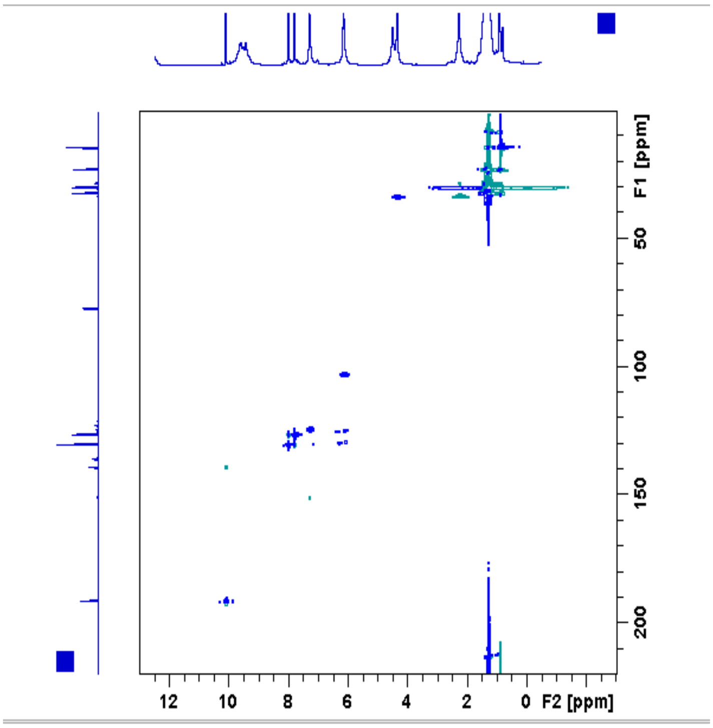

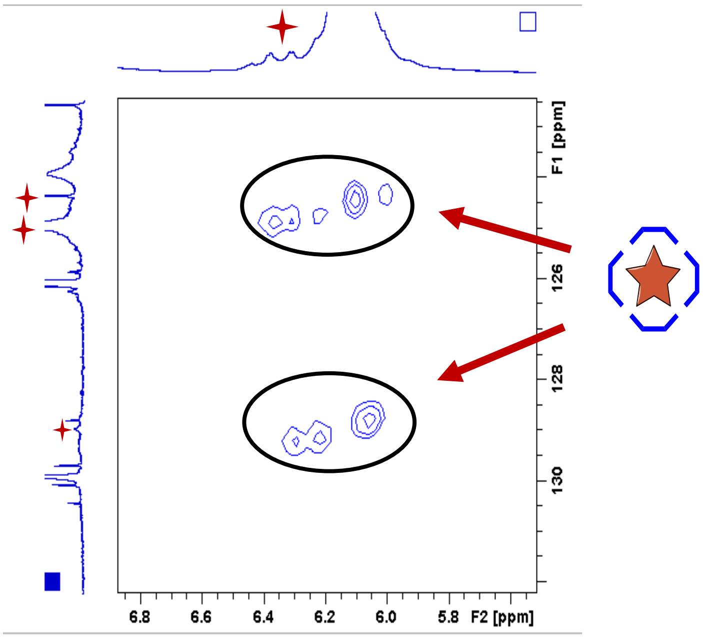

Figure S58. HSQC NMR spectrum (400 MHz, $CDCl_3$, 298 K) of the mixture **2b** (0.846 mmol) and **CR_6** (0.042 mmol). ^1J correlations attributable to the aromatic protons of **2b** inside the nanocontainer **CR_6** are marked.[14]

DOSY experiment

DOSY experiments were performed on a Bruker Avance-600 spectrometer equipped with 5 mm PABBO BB|19F-1H\D Z-GRD Z114607/0109. The standard Bruker pulse program, ledbpgp2s, employing a double stimulated echo sequence and LED, bipolar gradient pulses for diffusion, and two spoil gradients were utilized. Diffusion times were 150 ms, eddy current delay was 5 ms, gradient recovery delays was 0.2 ms and gradient pulse was 1400 ms. Individual rows of the quasi-2D diffusion databases were phased and baseline corrected.

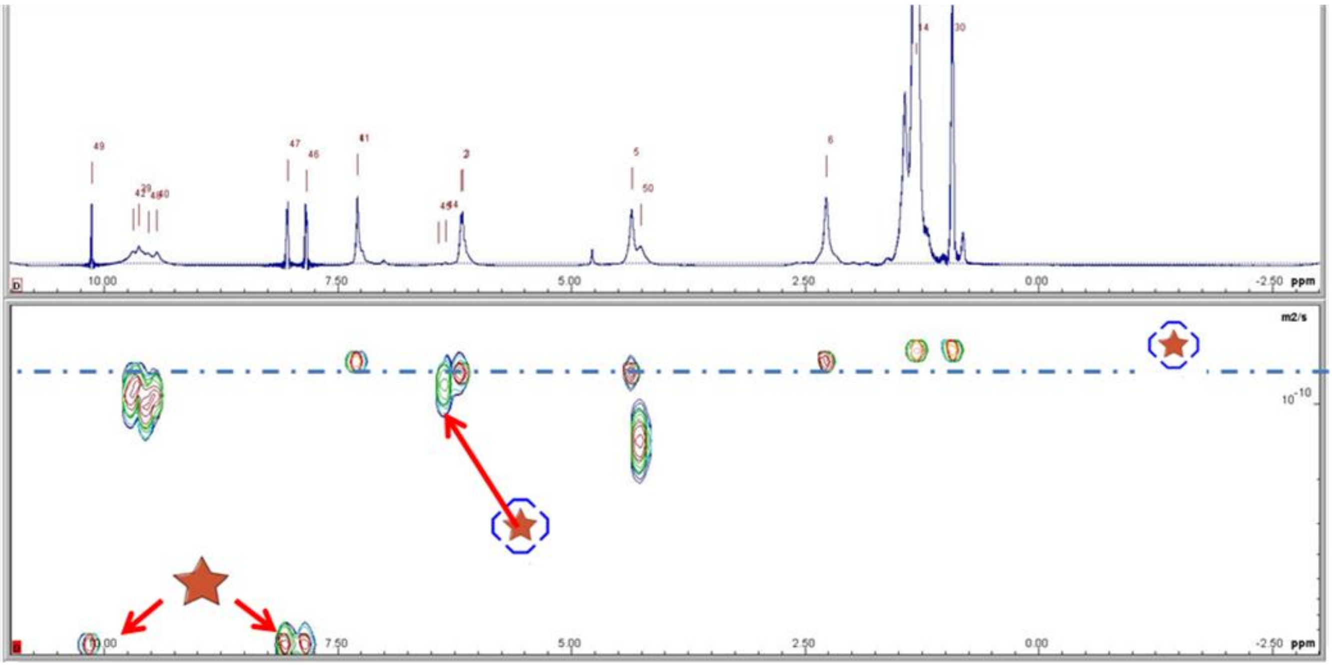

Figure S59. DOSY NMR (600 MHz, $CDCl_3$, 298 K) of the mixture of **2b** (0.846 mmol) and capsule **CR_6** (0.042 mmol).

As indicated in Figure S59, the signals pattern associated to **2b** inside the capsule **CR_6** are aligned with the capsule diffusion coefficient.

10. Proofs of the encapsulation of A

Resorcinarene **1** (281.6 mg, 254.7 μmol) was weighed in a 4 mL vial and 1 mL of $CDCl_3$ was added. The mixture was warmed at 50 °C until clarification (ca 5 min). To this solution p-chloro aniline **A** (0.0423 mmol) was added and the mixture was kept at 30 °C under stirring for 1 h before being subjected to NMR spectroscopy. An aliquot portion of the mixture prepared (500 μL) was taken and subjected to NMR experiments.

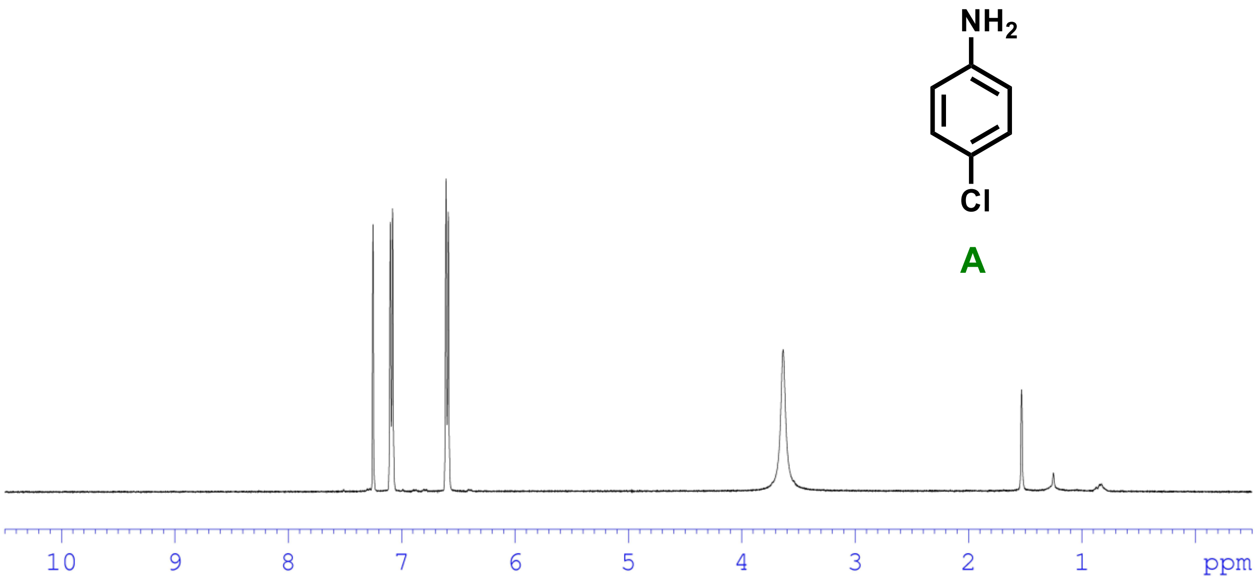

Figure S60. ^{1}H NMR spectrum (400 MHz, $CDCl_3$, 298 K) of *p*-chloroaniline **A**.

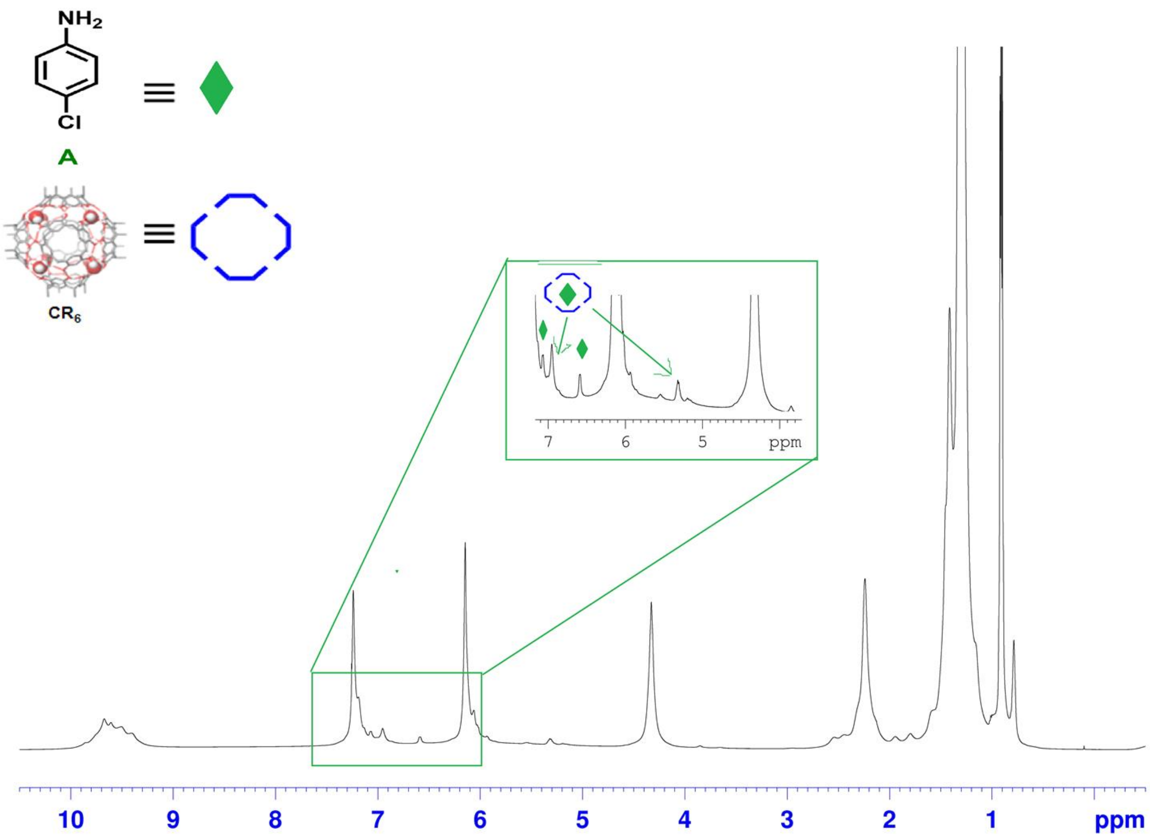

Figure S61. ^{1}H NMR spectrum (600 MHz, $CDCl_3$, 298 K) of the mixture of **A** (42.3 mM) and $\mathbf{CR_6}$ (42.3 mM)

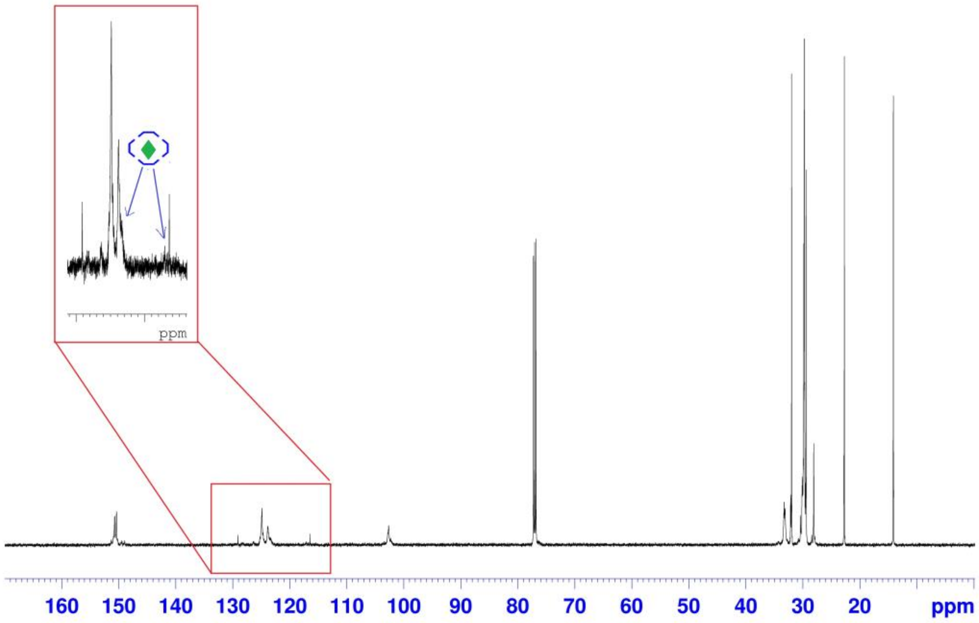

Figure S62. ^{13}C NMR spectrum (150.03 MHz, $CDCl_3$, 298 K) of the mixture of p-chloroanililne **A** and $\mathbf{CR_6}$, 42.3 mM each component.

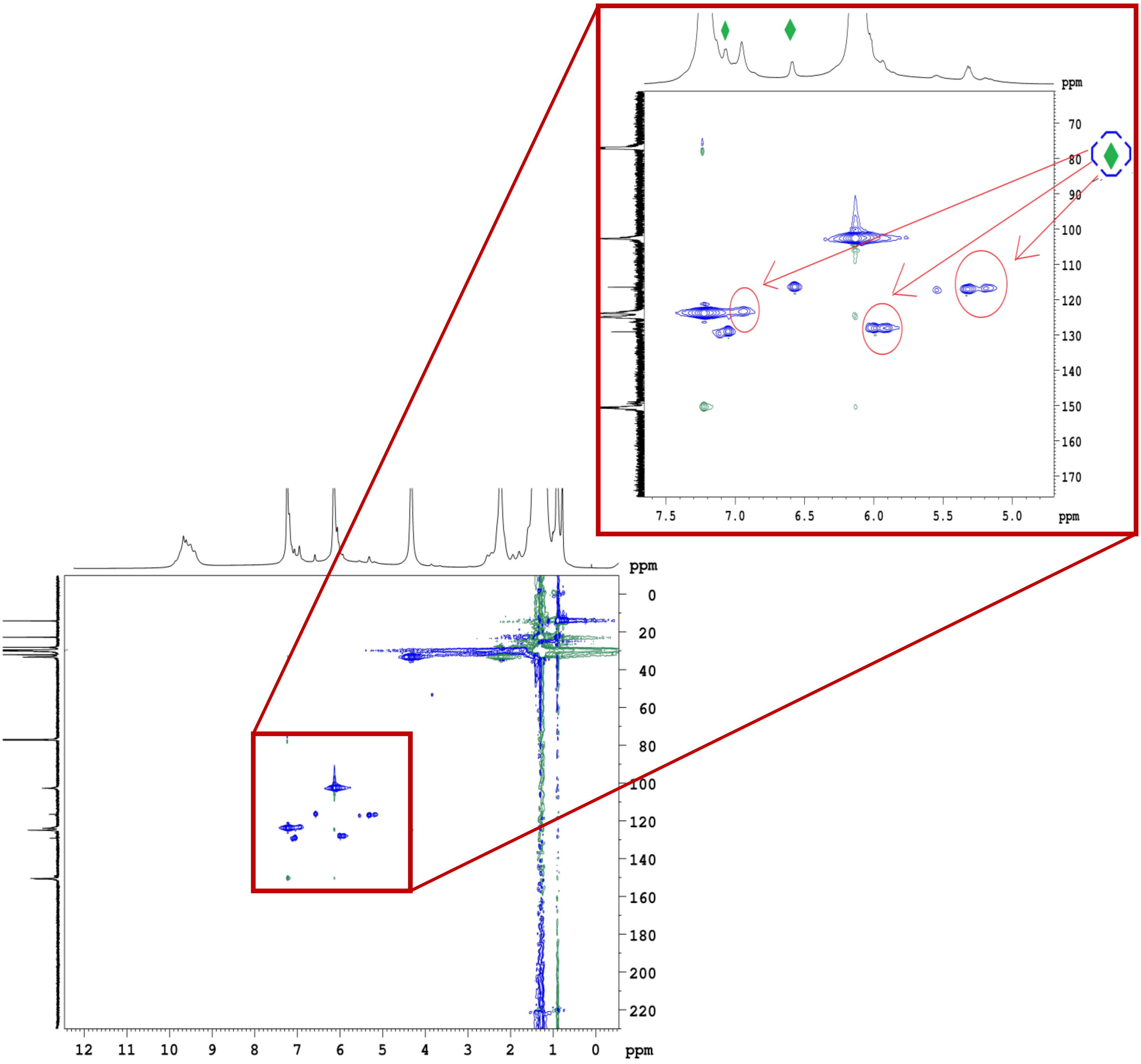

Figure S63. HSQC NMR spectrum (600 MHz, $CDCl_3$, 298 K) of the mixture **A** (42.3 mM) and **CR$_6$** (42.3 mM).

11. Predatory effect of CR₆. Stability of imines in presence of CR₆ (Figure 8 in the main text)

11.1 General experimental conditions

Resorcinarene **1** (281.6 mg, 254.7 μmol, 6 equiv) was weighed in a 4 mL vial and 1 mL of water saturated deuterated chloroform was added. The mixture was warmed at 50 °C until clarification (ca 5 min). To this solution the appropriate imine (0.0423 mmol, 1 equiv) was added at 30 °C under stirring. The mixture was monitored by taking aliquots over time and recording ^{1}H-NMR spectra. An aliquot portion of the mixture (30 μL) was taken and diluted with 470 μL of $CDCl_3$ (freshly filtered through activated 3Å molecular sieves and basic aluminium oxide) and, after by adding TCE (1 μL) as internal standard, the mixture was monitored by ^{1}H NMR spectroscopy. The spectra were recorded before and after addition of DMSO (2 μL) to the aliquot. The stability of imines was monitored by ^{1}H-NMR spectra as a function of time.

11.2 Stability of imine A2a in presence and in absence of CR₆

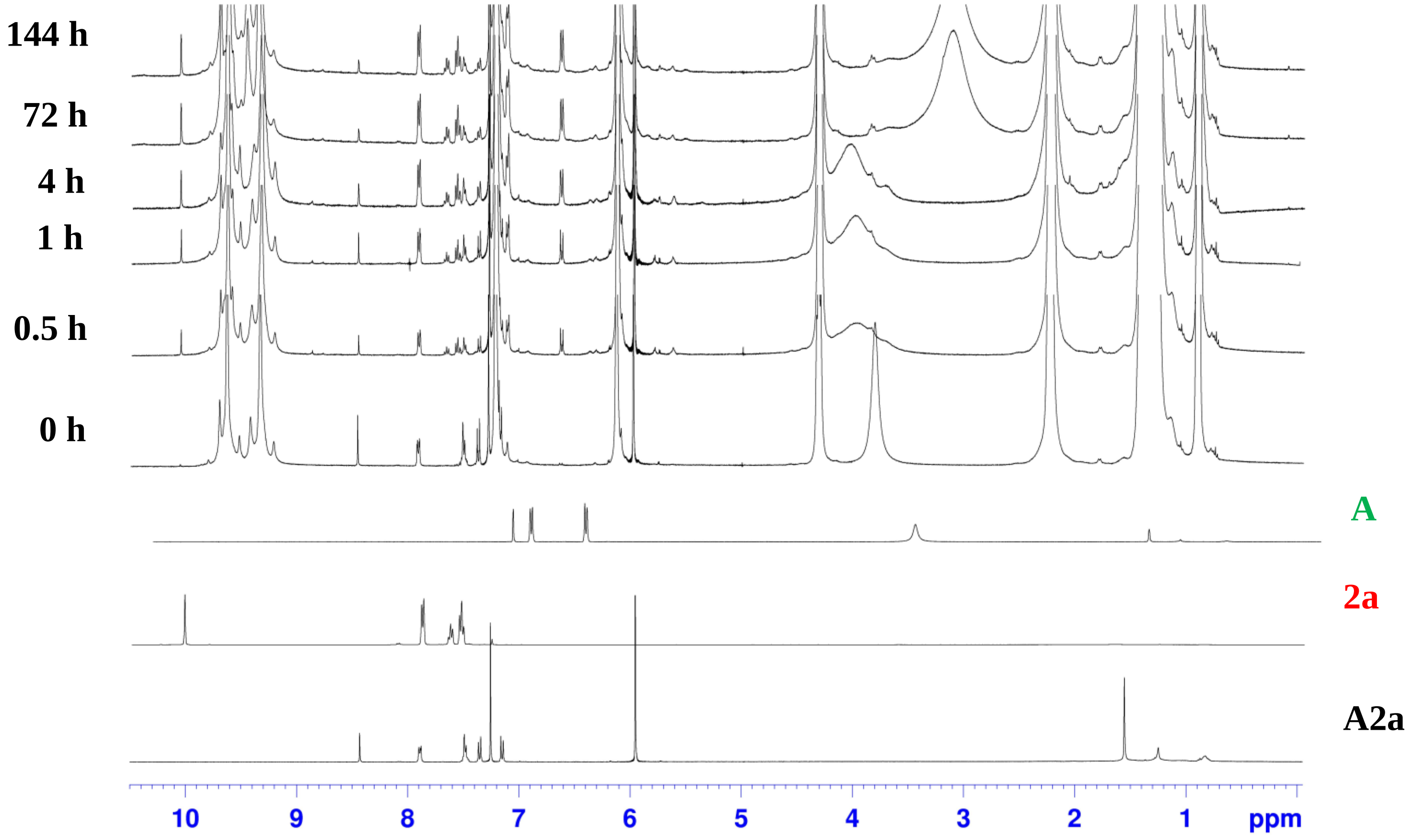

Figure S64. ^{1}H NMR (400 MHz, $CDCl_3$, 298 K) spectra of an equimolar mixture of **A2a** and **CR₆** immediately after preparation and after, 0.5, 1, 4, 72 and 144 h. (Bottom) ^{1}H NMR spectra of **A2a, 2a** and **A**.

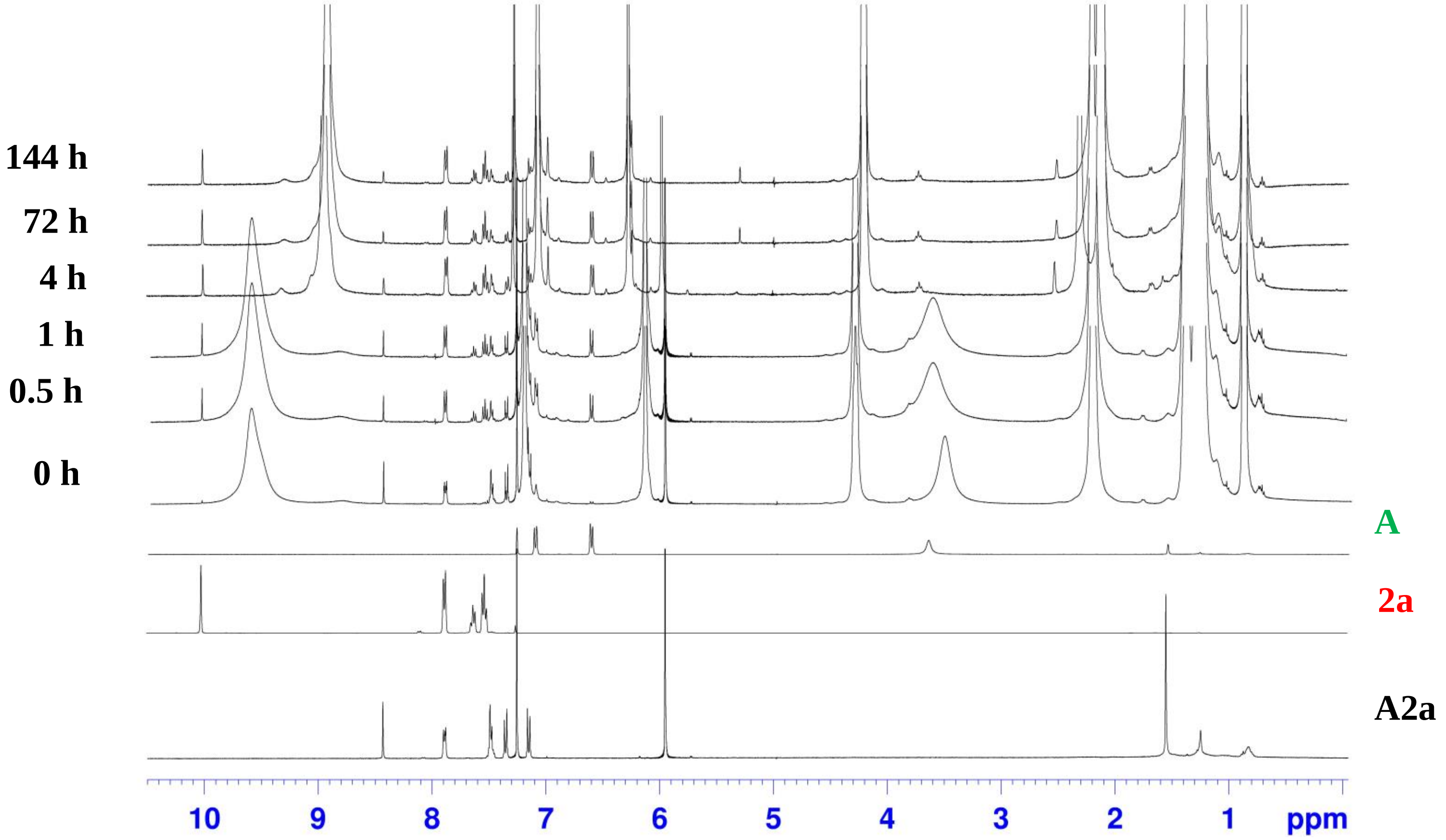

Figure S65. ^{1}H NMR (400 MHz, $CDCl_3$, 298 K) spectra of an equimolar mixture of **A2a** and **CR$_6$** immediately after preparation and after 0.5, 1, 4, 72 and 144 h. The spectra of the mixture were recorded after addition of DMSO to the collected aliquots. (Bottom) ^{1}H NMR spectra of **A2a, 2a** and **A**.

Table S14. Hydrolysis of **A2a** in presence of **CR$_6$**.[a]

Time[a] (h)	A2a (%)[b]	2a (%)[b]
**0**	100	-[c]
**0.5**	38	62
**1**	36	64
**4**	35	65
**72**	34	66
**144**	34	66

[a] Time at which an aliquot (30 μL) of the mixture was taken and monitored via ^{1}H-NMR spectrum. [b] Conversion calculated after addition of DMSO. [c]Below the limit of detection. Error in ^{1}H-NMR signal integration was ± 5%.

11.3 Uptake of 2a inside the capsule after hydrolysis of A2a in presence of CR_6.

With respect to the total quantity of aldehyde **2a** obtained by hydrolysis of **A2a**, the uptake of aldehyde **2a** within **CR_6** was measured by quantitative ^{1}H NMR experiments following the experimental conditions reported in paragraph *11.1*. The quantity of encapsulated aldehyde was obtained by difference between the concentration of the free aldehyde in solution with **CR_6** and that measured after disassembly of the hexameric capsule by adding DMSO. The ^{1}H NMR signal of the free aldehyde was integrated with respect to the signal of the internal standard (TCE).

Table S15. Uptake of aldehyde 2a inside CR_6 (Table 1 in the main text)

time (h)	Integral [a] (before adding DMSO)	mmol of free aldehyde (**before adding DMSO**)	Integral [a] (after addition DMSO)	mmol free aldehyde (**after addition DMSO**)	Uptake (%)
0.5	0.0589	0.0187	0.0825	0.0261	28
1	0.0610	0.0193	0.0853	0.0270	29
4	0.0603	0.019	0.0866	0.0270	29

[a] Integral of free aldehyde proton signal at 10.0 ppm.

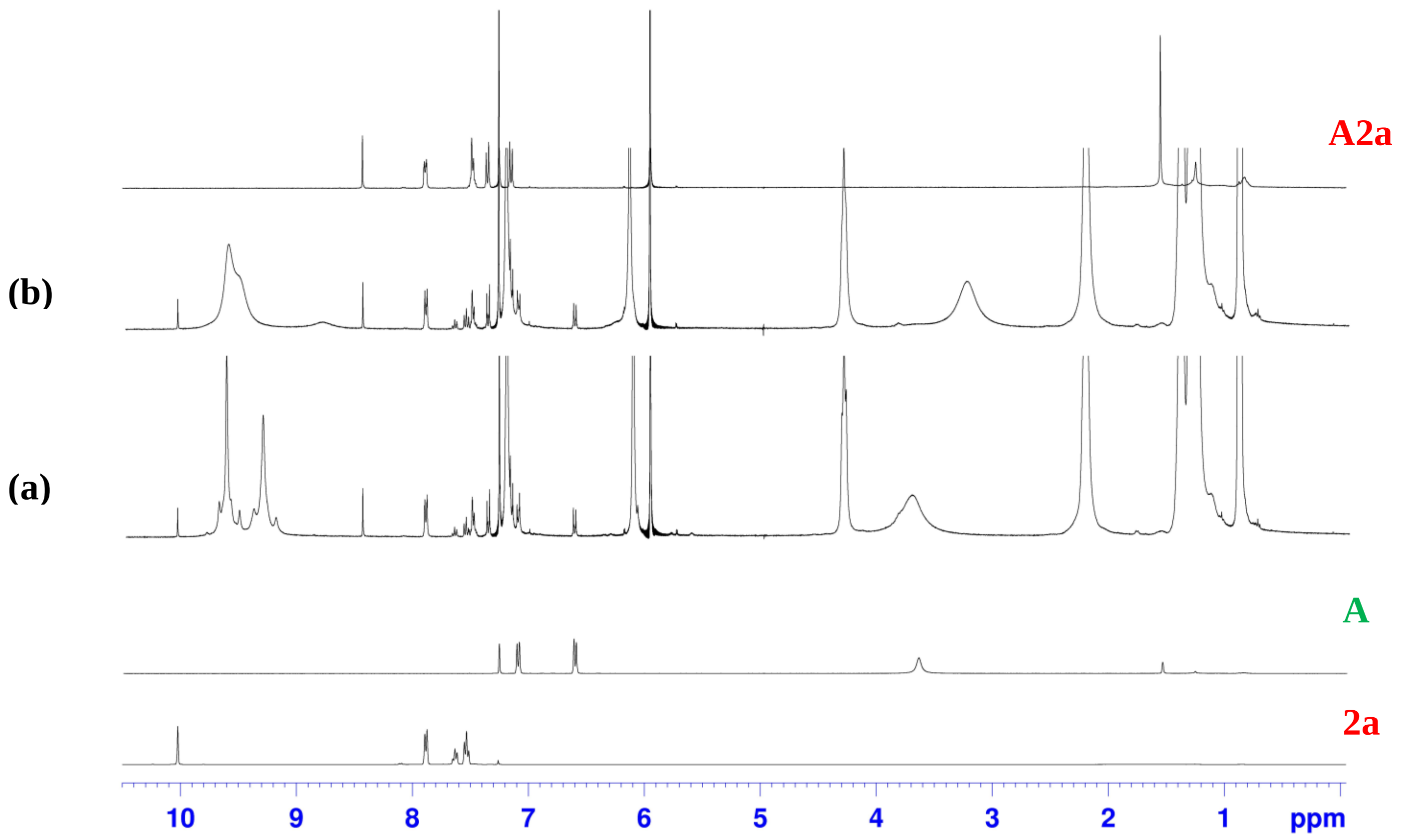

Figure S66. ^{1}H NMR (400 MHz, $CDCl_3$, 298 K) spectra of the mixture of **A2a** (0.0423 mmol) and **CR_6** (0.0211 mmol) after 1 h. The spectra of the mixture were recorded before (a) and after (b) addition of DMSO to the collected aliquot. (Bottom), ^{1}H NMR spectra of **2a** and **A**, and (top) ^{1}H NMR spectrum of **A2a**.

The hydrolysis of **A2a** to **2a** and **A** in the presence of 0.5 equiv. of **CR_6** was slower than in the presence of 1.0 equiv. of **CR_6** ((cfr. figure S66 with S65 and S64 at 1h). In fact, after 1 h the conversion of **A2a** to **2a** and **A** was about 38 %.

Stability of imine A2a in absence of CR_6

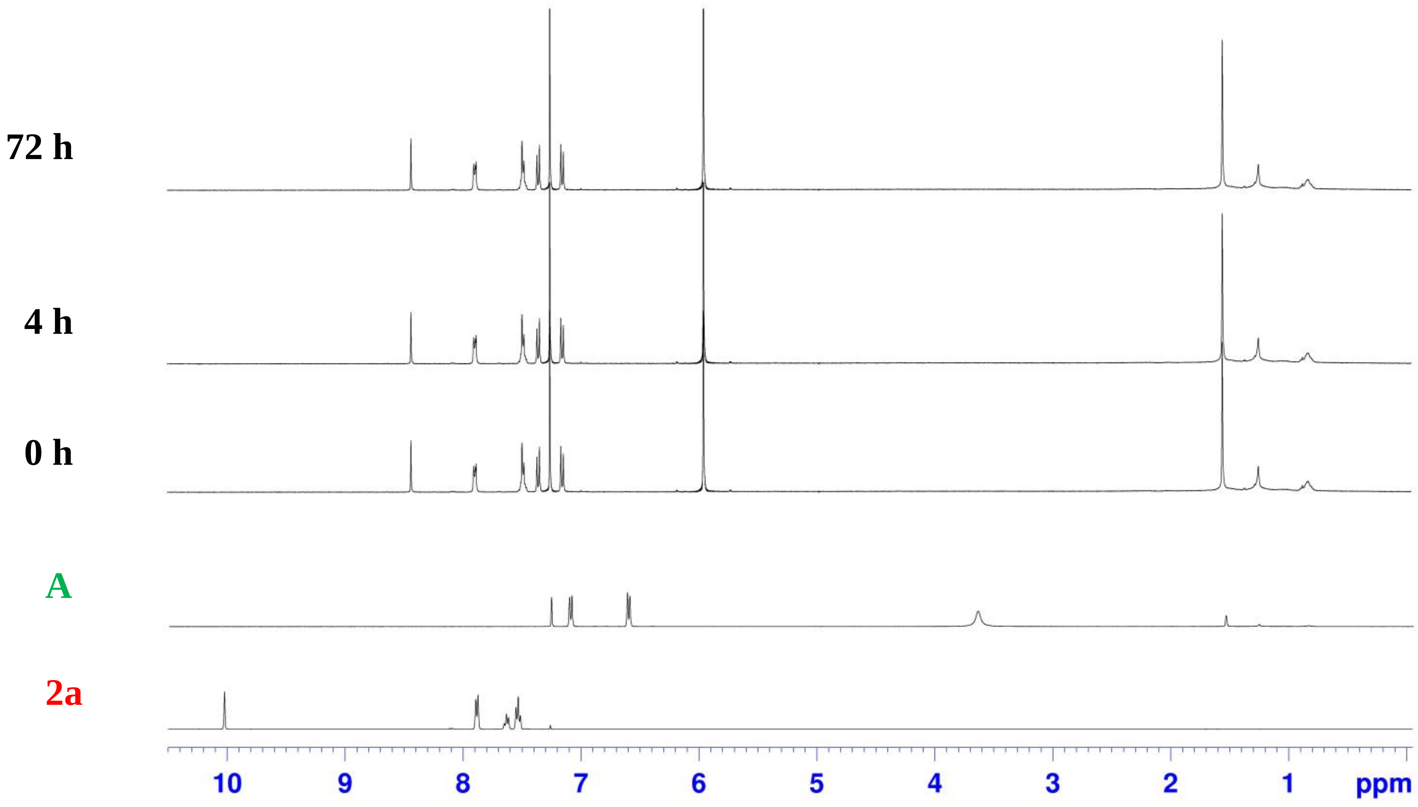

Figure S67a. ^{1}H NMR (400 MHz, $CDCl_3$, 298 K) spectra of the evolution of the solution of **A2a** (0.0423 mmol) in water saturated $CDCl_3$ (1.0 mL) immediately after preparation and after 4 h and 72 h. On the bottom, the spectra corresponding to isolated **2a** and **A** are reported.

A control experiment was performed in the presence of DMSO, a polar additive able to destroy the capsule.[17] To a solution of CR_6, prepared as described in *par. 11.1*, DMSO (300 µL, 100 eq. per capsule $\mathbf{CR_6}$) was added, followed by imine **A2a** (0.0423 mmol). The evolution of the mixture was followed over time and compared with that in the presence of CR_6 (cfr. FigS67b with FigS64). The results confirmed that the addition of DMSO, and so the destruction of the capsule, prevented the hydrolysis of **A2a**.

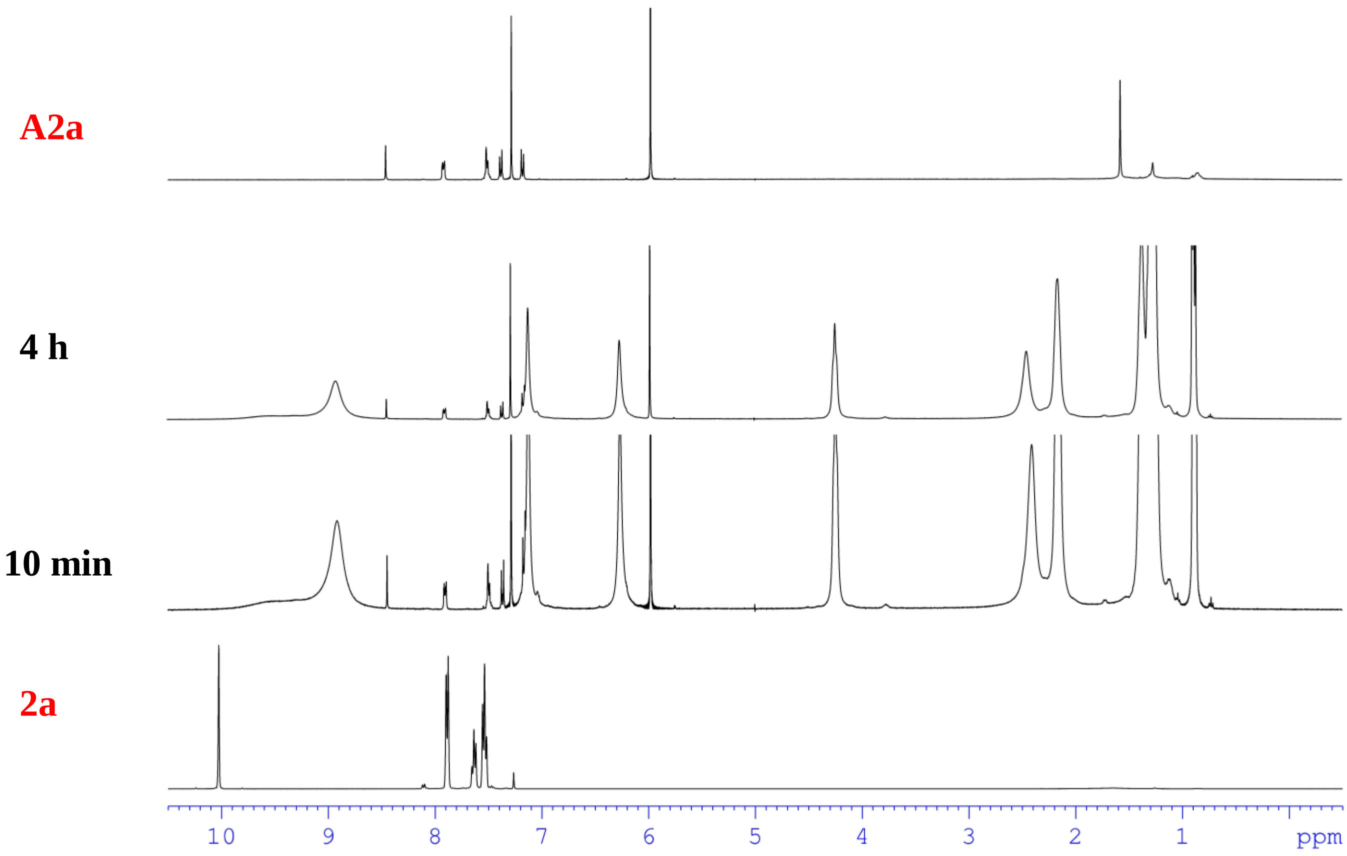

Figure S67b. ^{1}H NMR (400 MHz, $CDCl_3$, 298 K) spectra of the evolution of the solution of **A2a** (0.0423 mmol) in water saturated $CDCl_3$ (1.0 mL) in the presence of $\mathbf{CR_6}$ (0.0423 mmol) and DMSO (0.3 mL) after 10 min. and 4 h. (Bottom), ^{1}H NMR spectrum corresponding to isolated **2a** and (top), ^{1}H NMR spectrum of **A2a.**

11.4 Stability of imine A2b in presence and in absence of CR_6

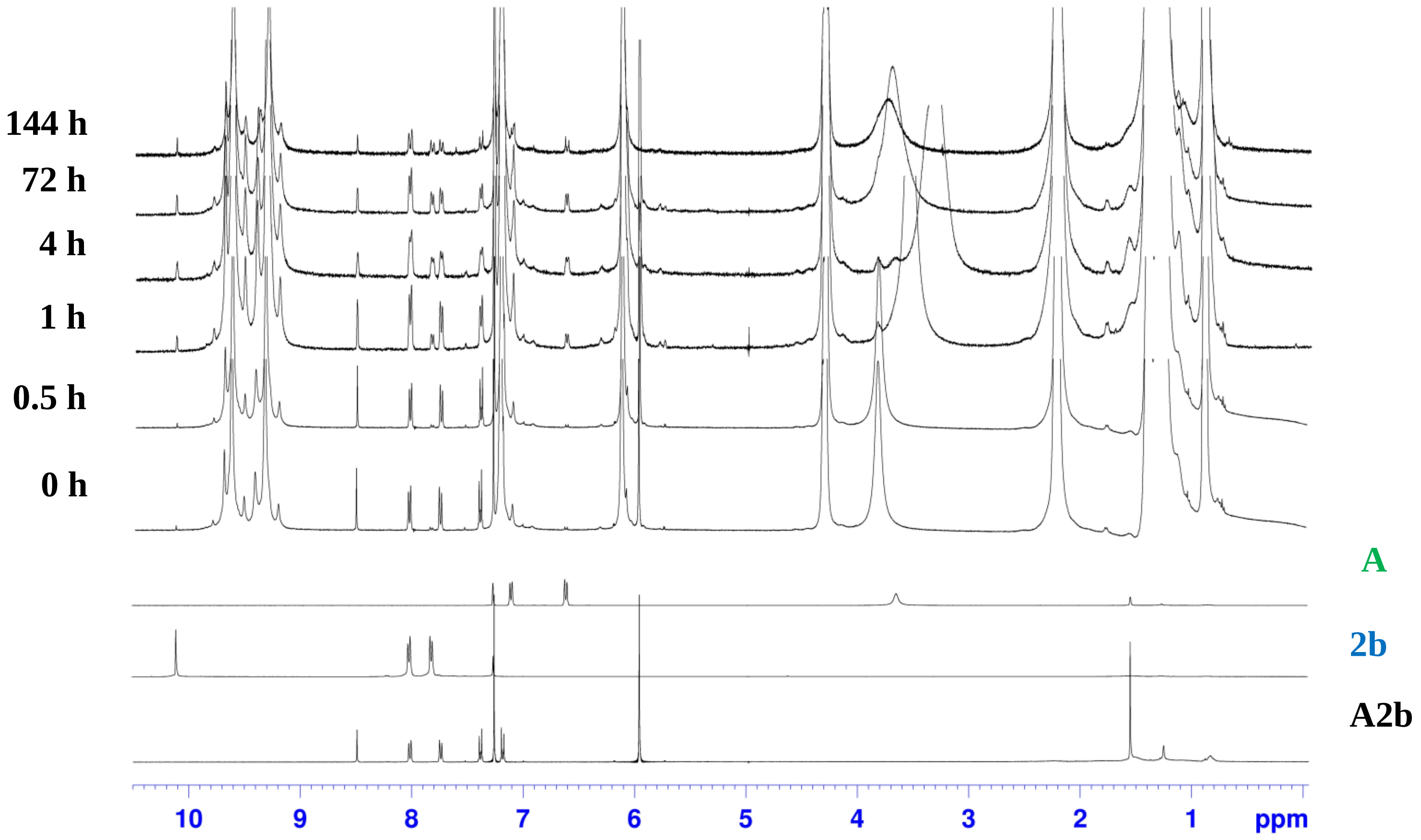

Figure S68. ^{1}H NMR (400 MHz, $CDCl_3$, 298 K) spectra of an equimolar mixture of **A2b** and **CR_6** immediately after preparation and after 0.5, 1, 4, 72 and 144 h. (Bottom) ^{1}H NMR spectra of **A2b, 2b** and **A**.

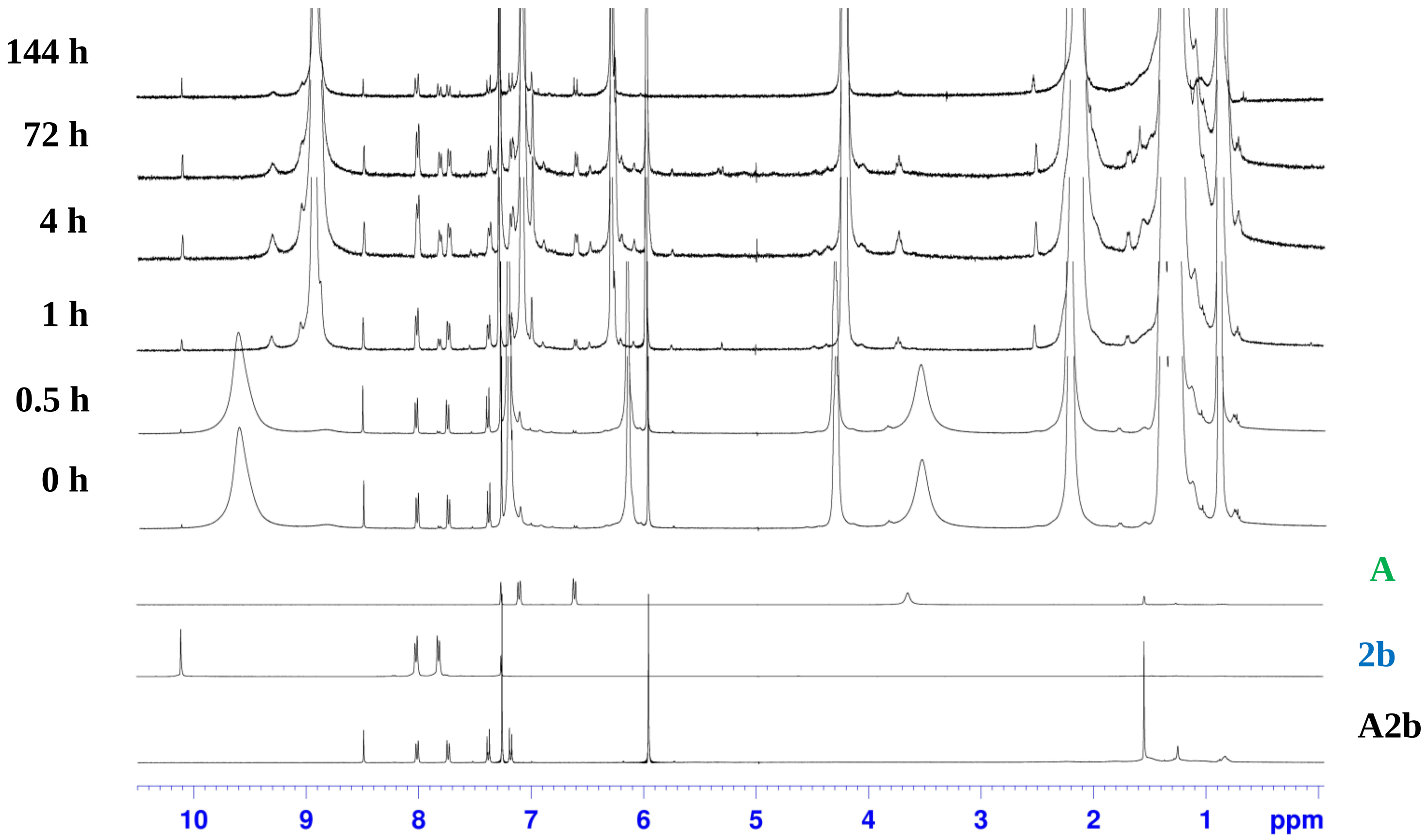

Figure S69. ^{1}H NMR (400 MHz, $CDCl_3$, 298 K) spectra of an equivalent mixture of **A2b** and **CR_6** immediately after preparation and after 0.5, 1, 4, 72 and 144 h. (Bottom) ^{1}H NMR spectra of **A2b, 2b** and **A**. The spectra of the mixture were recorded after addition of DMSO to the collected aliquots.

Table S16. Hydrolysis of **A2b** in presence of **CR_6**.

Time[a] (h)	A2b (%)[b]	2b (%)[b]
**0**	100	-[c]
**0.5**	100	-[c]
**1**	98	2
**4**	85	15
**72**	60	40
**144**	60	40

[a] Time at which an aliquot (30 μL) of the mixture was taken and monitored via ^{1}H-NMR spectrum. [b] Conversion calculated after addition of DMSO. [c]Below the limit of detection. Error in ^{1}H-NMR signal integration was ± 5%.

11.5 Uptake of 2b inside the capsule after hydrolysis of A2b in presence of CR_6.

With respect to the total quantity of aldehyde **2b** obtained by hydrolysis of **A2b**, the uptake of aldehyde **2b** within **CR_6** was measured by quantitative ^{1}H NMR experiments following the experimental conditions reported in paragraph *10.1*. The quantity of encapsulated aldehyde was obtained by difference between the concentration of the free aldehyde in solution with **CR_6** and that calculated after disassembly of the hexameric capsule by adding DMSO. The ^{1}H NMR signal of the free aldehyde was integrated with respect to the signal of the internal standard (TCE).

Table S17. Uptake of aldehyde 2b inside CR_6 (Table 1 in the main text)

time (h)	Integral[a] (before adding DMSO)	mmol free aldehyde **(before adding DMSO)**	Integral[a] (after addition DMSO)	mmol free aldehyde **(after addition DMSO)**	Uptake (%)
4	0.0199	0.006	0.0199	0.006	-
72	0.0534	0.0169	0.0534	0.0169	-

[a] Integral of free aldehyde proton signal at 10.1 ppm

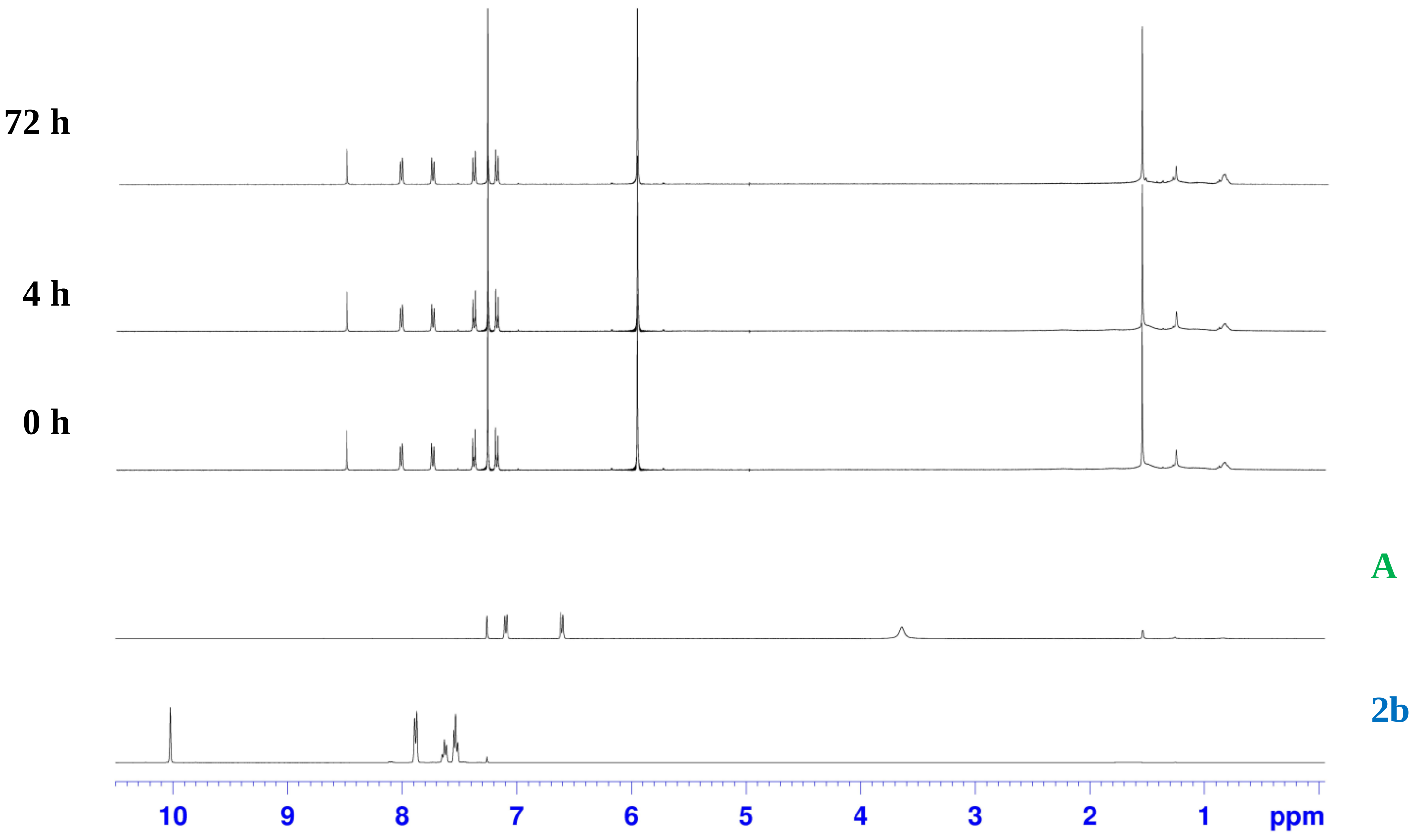

Figure S70. ^{1}H NMR (400 MHz, $CDCl_3$, 298 K) spectra of a mixture of **A2b** (0.0423 mmol) in water - saturated $CDCl_3$ (1mL) immediately after preparation and after 4 h and 72 h. On the bottom, the spectra corresponding to isolated **2b** and **A** are reported.

11.6 Stability of imine B2a in presence and in absence of CR$_6$ (Figure 11 in the main text).

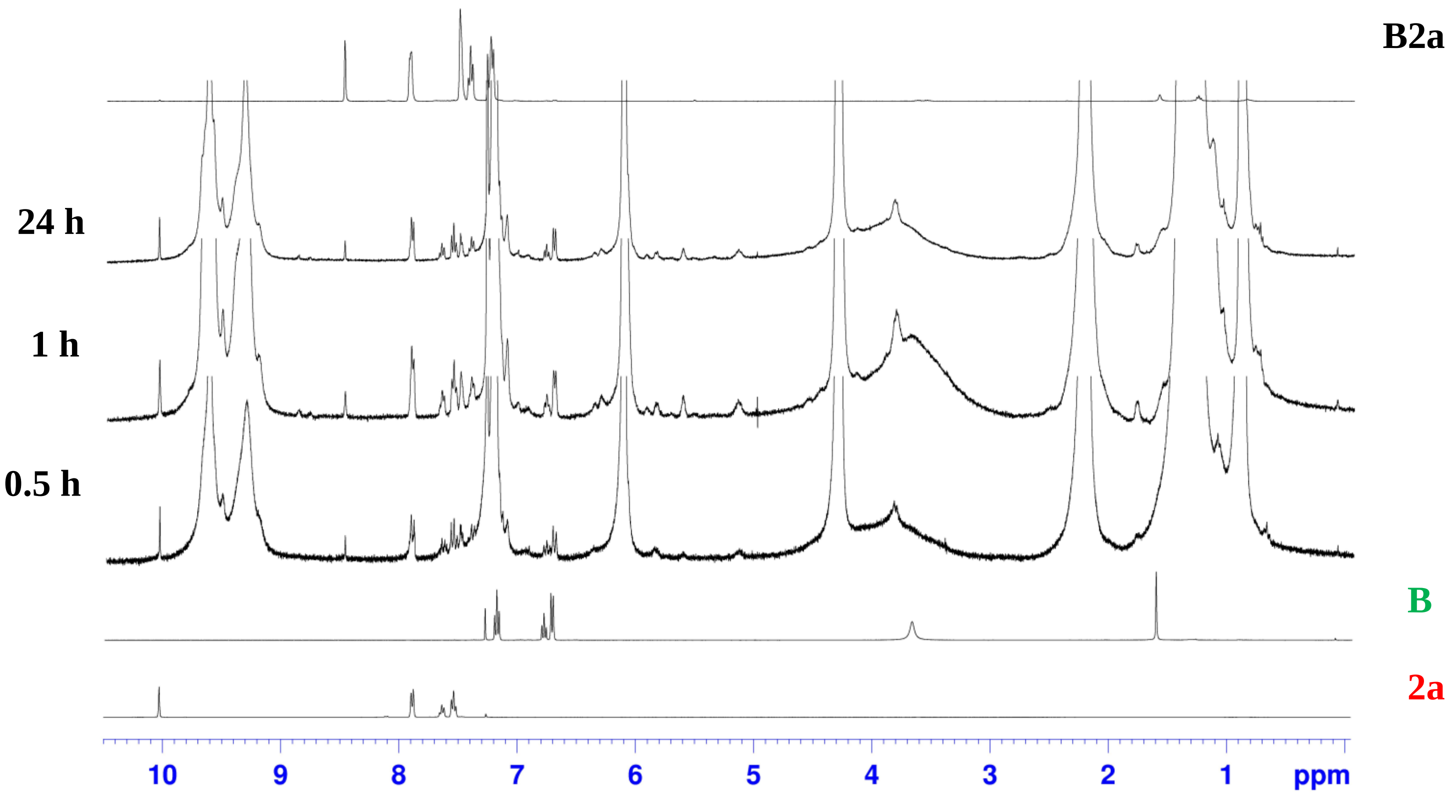

Figure S71. ^{1}H NMR (400 MHz, $CDCl_3$, 298 K) spectra of an equimolar mixture of **B2a** and **CR$_6$** (prepared following the procedure 11.1), after 0.5, 1, and 24 h. (Bottom), ^{1}H NMR spectra of **2a** and **B**. (Top) ^{1}H NMR spectrum of the imine **B2a**.

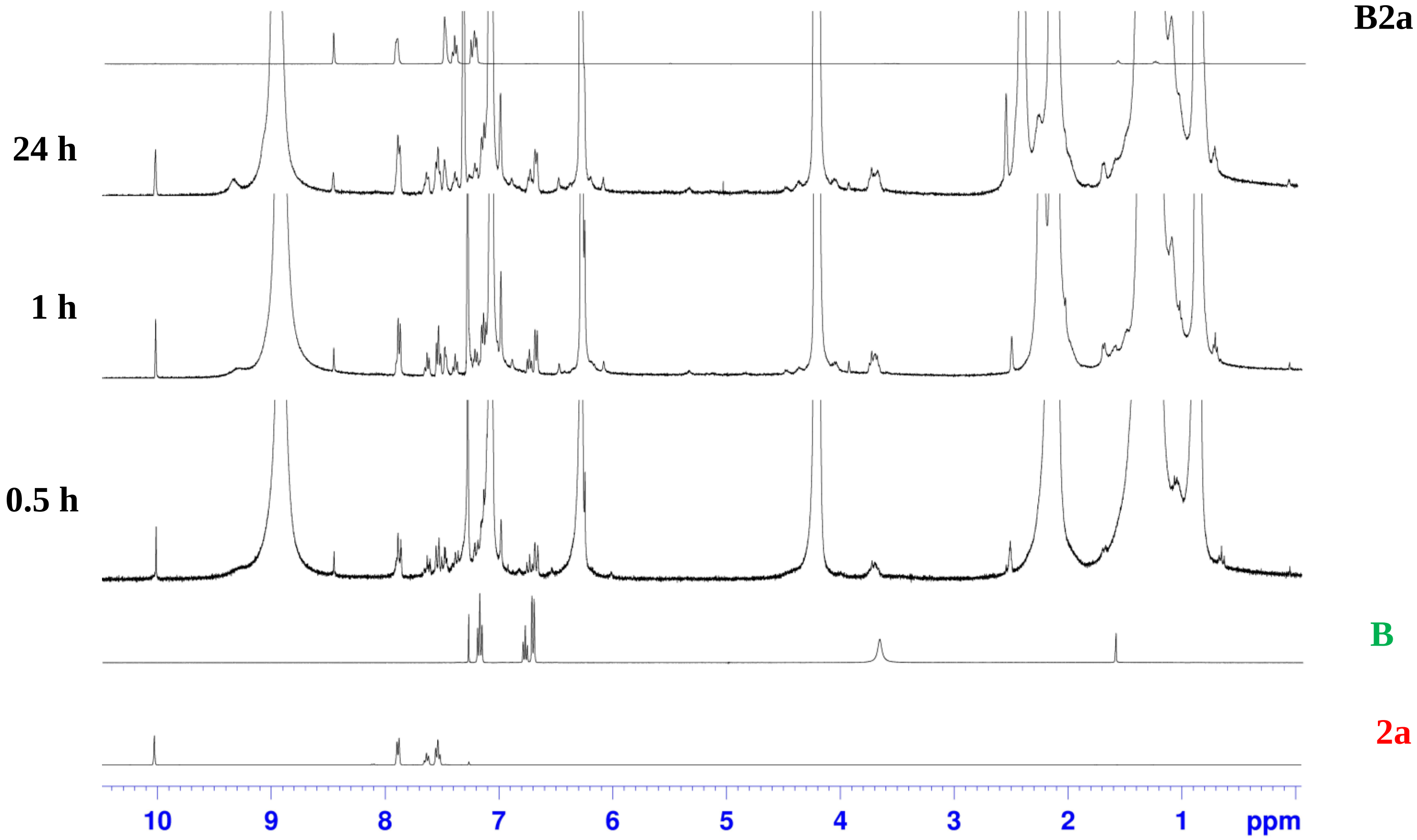

Figure S72. ^{1}H NMR (400 MHz, $CDCl_3$, 298 K) spectra of an equimolar mixture of **B2a** and **CR_6** (prepared following the procedure 11.1), after 0.5, 1, and 24 h. (Bottom), ^{1}H NMR spectra of **2a** and **B**. (Top) ^{1}H NMR spectrum of the imine **B2a**. The spectra of the mixture were recorded after addition of DMSO to the collected aliquots.

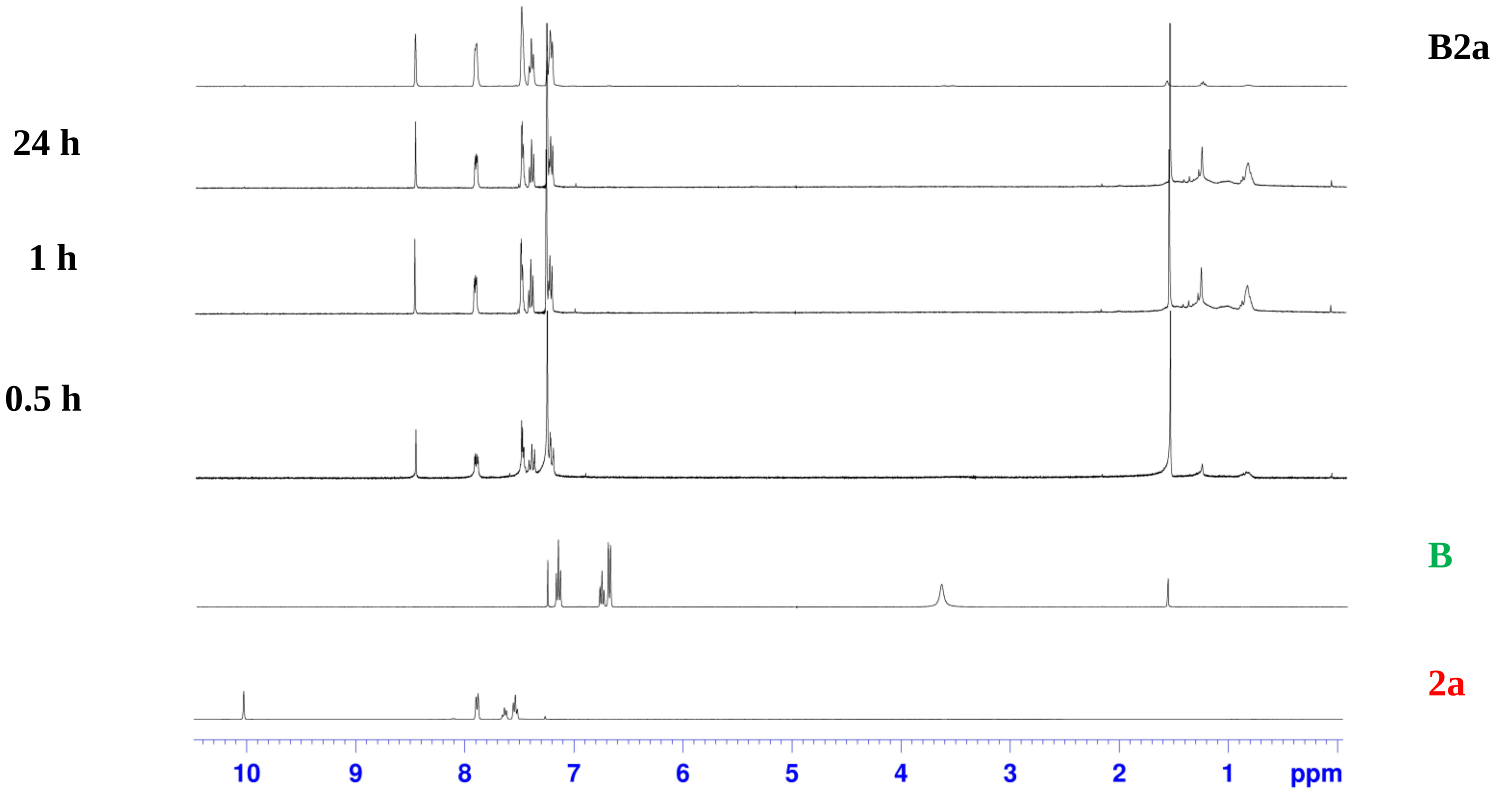

Figure S73. ^{1}H NMR (400 MHz, $CDCl_3$, 298 K) spectra of **B2a**, after 0.5, 1, and 24 h. (Bottom), ^{1}H NMR spectra of **2a** and **B**. (Top) ^{1}H NMR spectrum of the imine **B2a**.

Table S18. Hydrolysis of **B2a** in presence of $\mathbf{CR_6}$.[a]

**Time[a] (h)**	**B2a (%)[b]**	**2a (%)[b]**
**0**	100	-[c]
**0.5**	40	60
**1**	27	73
**4**	27	73
**24**	27	73

[a] Time at which an aliquot (30 μL) of the mixture was taken and monitored via ^{1}H-NMR spectrum. [b] Conversion calculated after addition of DMSO. [c]Below the limit of detection. Error in ^{1}H-NMR signal integration was ± 5%.

11.7 Stability of imine C2a in presence and in absence of CR_6 (Figure 13 in the main text).

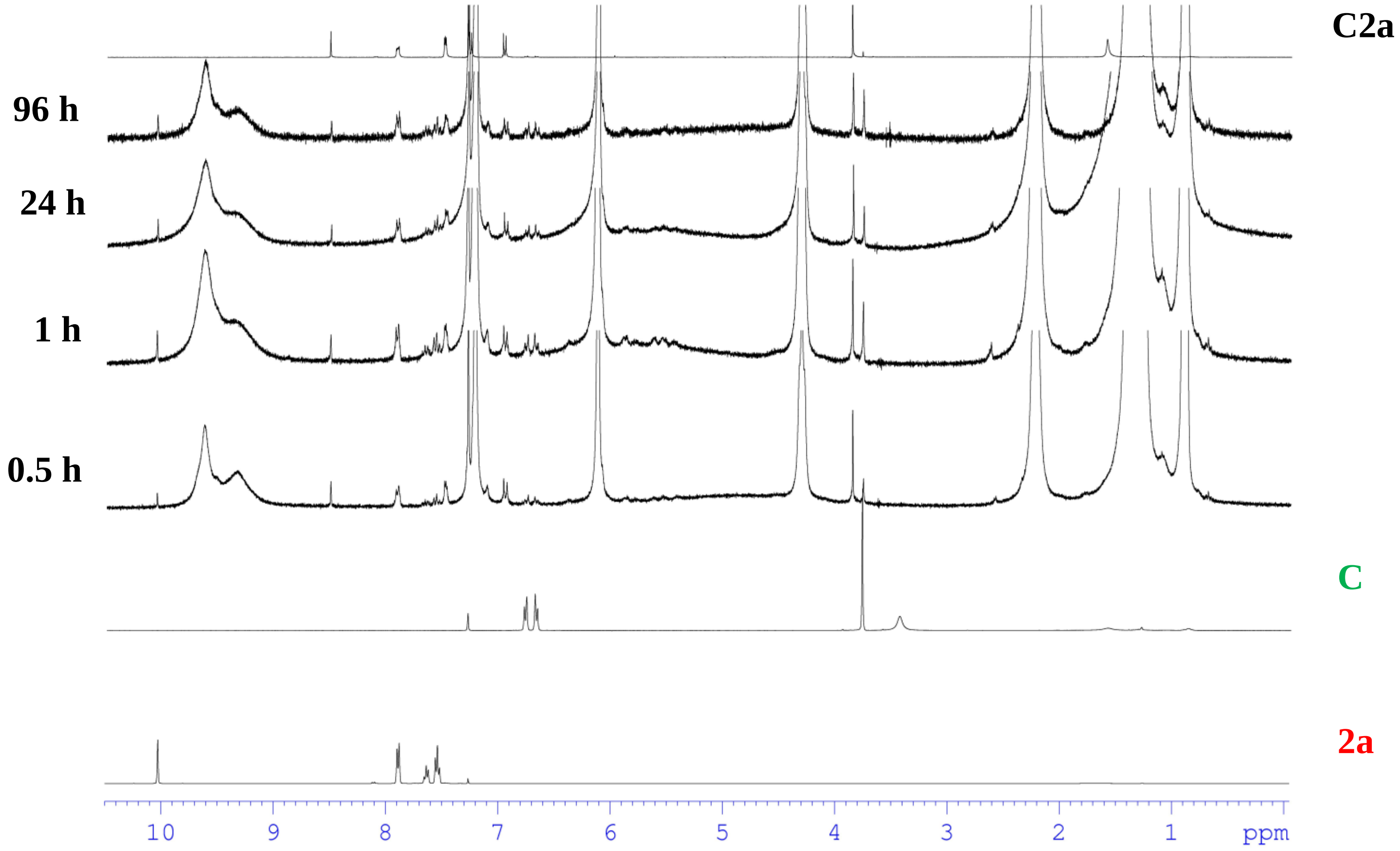

Figure S74. ^{1}H NMR (300 MHz, $CDCl_3$, 298 K) spectra of an equimolar mixture of **C2a** and **CR_6** (prepared following the procedure in 11.1) after 0.5, 1, 24 and 96 h. Bottom), ^{1}H NMR spectra of **2a** and **C**. (Top) ^{1}H NMR spectrum of the imine **C2a**.

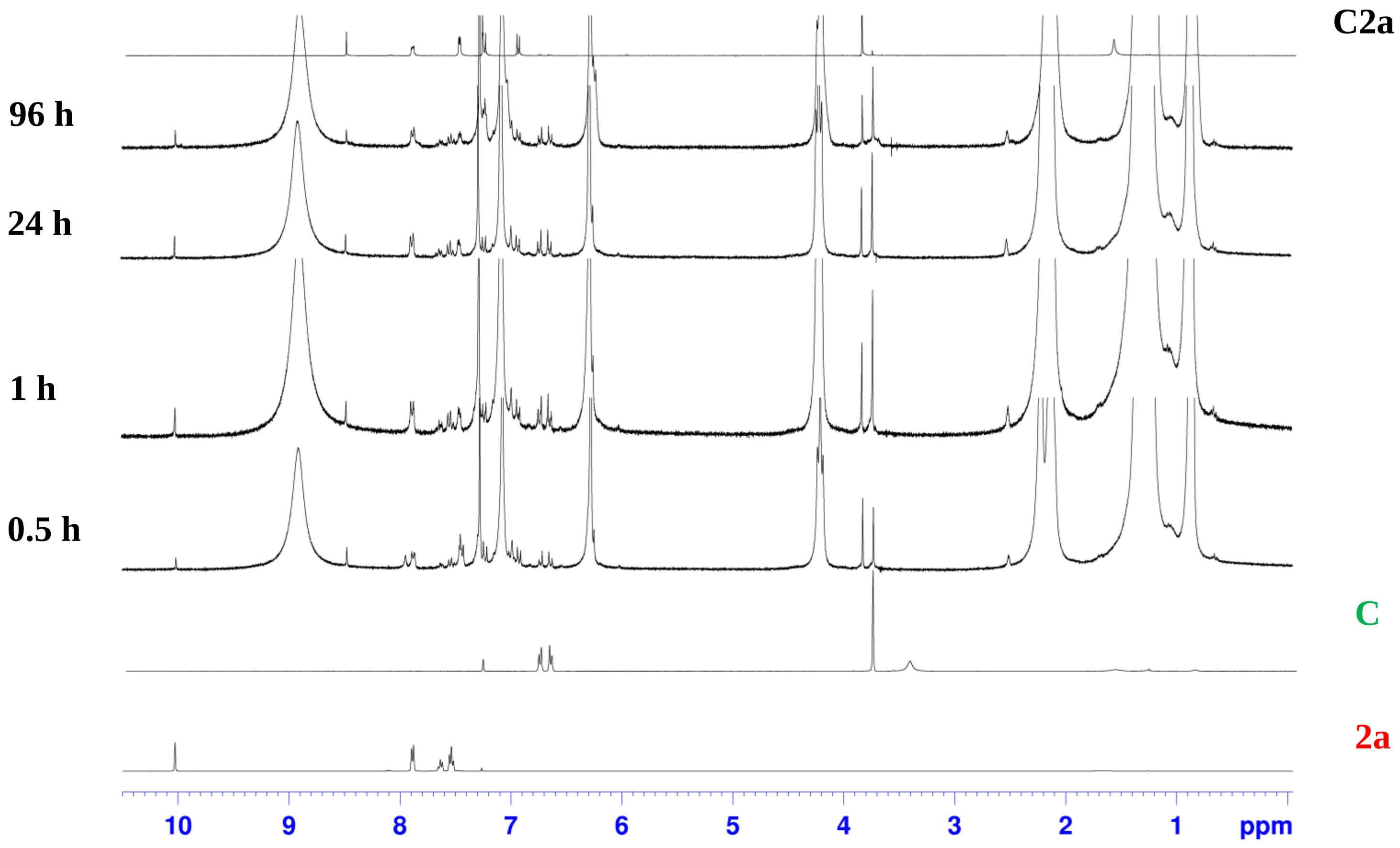

Figure S75. ^{1}H NMR (300 MHz, $CDCl_3$, 298 K) spectra of an equimolar mixture of **C2a** and **CR₆** (prepared following the procedure in 11.1) after 0.5, 1, 24 and 96 h. Bottom), ^{1}H NMR spectra of **2a** and **C**. (Top) ^{1}H NMR spectrum of the imine **C2a**. The spectra of the mixture were recorded after addition of DMSO to the collected aliquots.

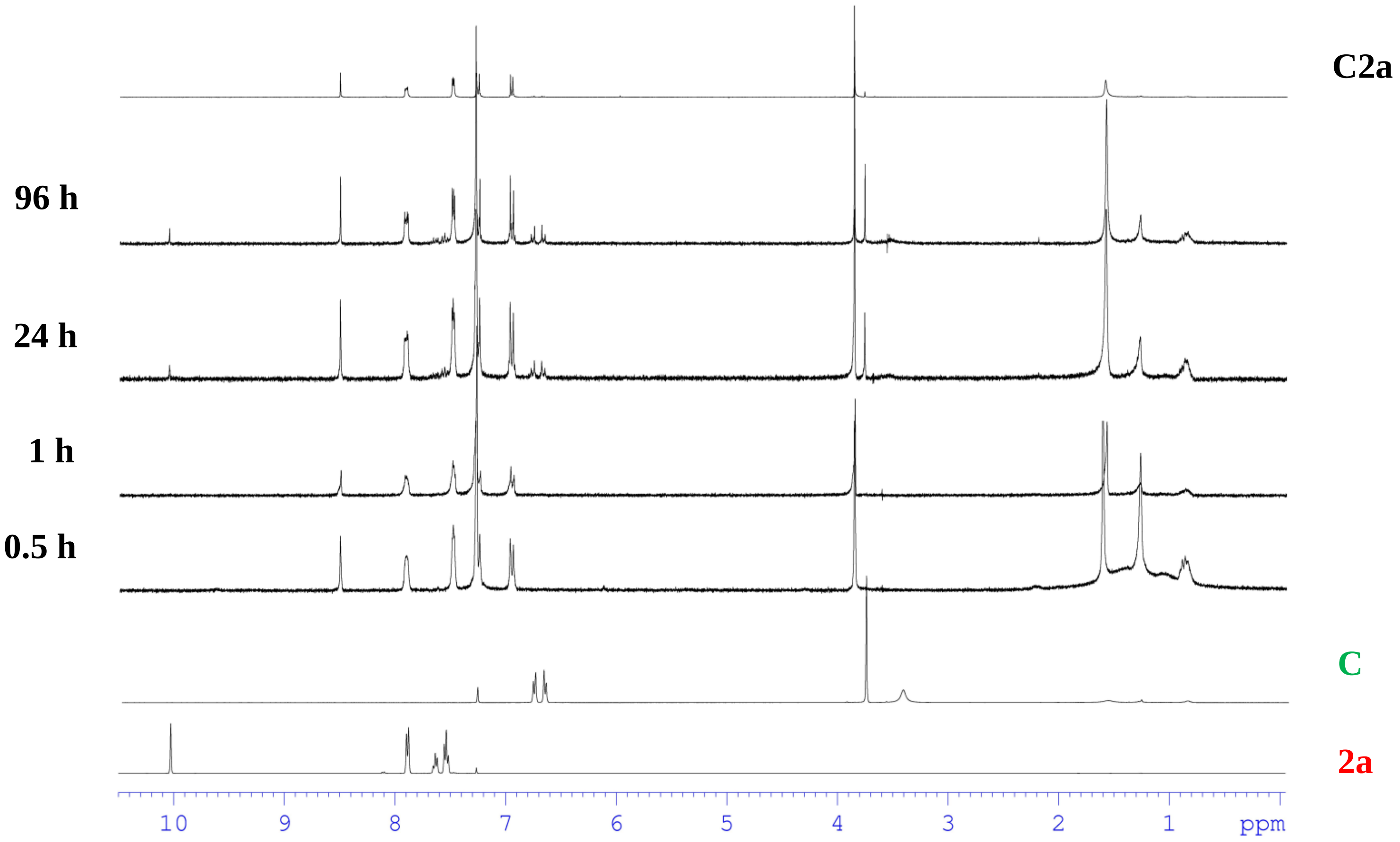

Figure S76. ^{1}H NMR (300 MHz, $CDCl_3$, 298 K) spectra of a solution of **C2a** after 0.5, 1, 24 and 96 h. Bottom), ^{1}H NMR spectra of **2a** and **C**. (Top) ^{1}H NMR spectrum of the imine **C2a**.

Table S19. Hydrolysis of **C2a** with and without **CR$_6$** (Figure 13 in the main text).[a]

	with CR$_6$		*without CR$_6$*	
**Time[a] (h)**	**C2a (%)[b]**	**2a (%)[b]**	**C2a (%)**	**2a (%)**
**0**	100	-[c]	100	-[c]
**0.5**	65	35	100	-[c]
**1**	41	59	100	-[c]
**24**	41	59	89	11
**96**	41	59	89	11

[a] Time at which an aliquot (30 µL) of the mixture was taken and monitored via ^{1}H-NMR spectrum. [b] Conversion calculated after addition of DMSO. [c]Below the limit of detection. Error in ^{1}H-NMR signal integration was ±5%.

12. Control experiments on concentration influence

In order to exclude the effect of the concentration on the measurements of the DCL components ratios by NMR spectra, we prepared a reaction mixture of **2a**, **2b** and **A** according to procedure reported in *par. 2.2* and, at interval reaction time=3h, we took two aliquots of volume 0.03 mL and 0.5 mL, respectively. The 0.03 mL aliquot was diluted with 0.470 mL of $CDCl_3$ before monitoring by ^{1}H-NMR spectroscopy (Figure S77a and c), instead 0.5 mL aliquot was used as it was (Figure S77b and d). As reported in Figure S77, both aliquots showed the same A2a/A2b ratio, determined by integration of the corresponding resonance signals in the spectra by comparison with the internal standard TCE and after addition of DMSO (2 μL) to the reaction aliquot.

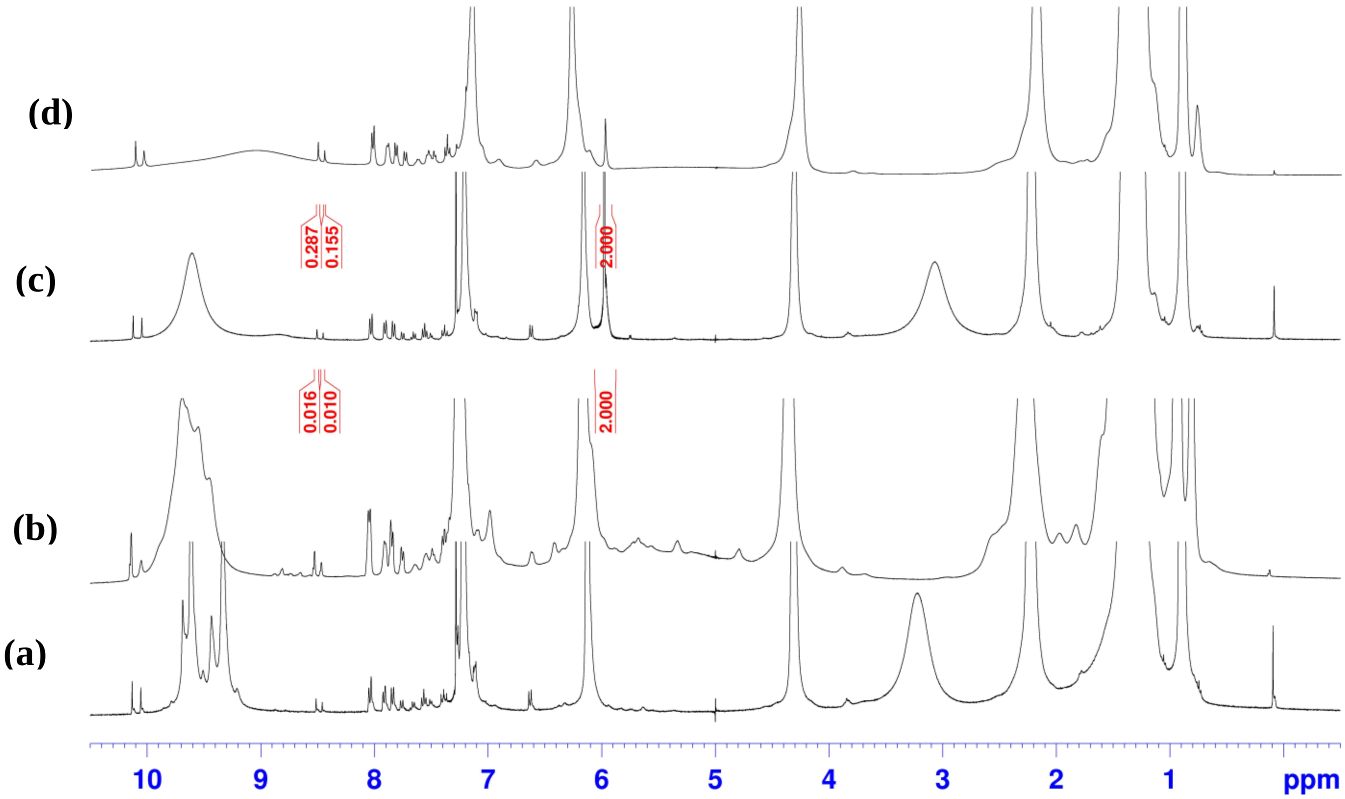

Figure S77. ^{1}H NMR (400 MHz, $CDCl_3$, 298 K) spectra of an equimolar mixture of **A, 2a, 2b** (0.0423 mmol each, water saturated $CDCl_3$, r.t.) and **CR**$_6$ (0.0423 mmol) after 3 h. (a) ^{1}H NMR spectra of **A, 2a** and **2b** at 2.5 mM. (b) ^{1}H NMR spectrum of **A, 2a** and **2b** at 42.3 mM**.** (c) ^{1}H NMR spectra of **A, 2a** and **2b** at 2.5 mM after addition of DMSO. (d) ^{1}H NMR spectrum of **A, 2a** and **2b** at 42.3 mM after addition of DMSO. TCE (tetrachloroethane, 0.0192 mmol) used as internal standard.

13. Computational studies

All calculations were performed using the ONIOM method implemented in the Gaussian16 package. M062X/dgdzvp level of theory was used for guests inside the capsule, OH groups of the resorcinarene units and for the 8 water molecules, while the semiempirical method PM6 was employed for all the other atoms.

The electronic zero-point corrected energies, enthalpies, and Gibbs free energies, expressed in Hartree, are reported in Tables S20 and S21. Enthalpy and Gibbs free energy in Table S21 are expressed in Kcal/mol.

Thermodynamic corrections were calculated at 298.15 K and 1 atm for all optimized geometries.

Table S20. Electronic zero-point corrected energies, enthalpies, and Gibbs free energies, expressed in Hartree

	**E(0)**	**E**	**H**	**G**	**Oniom total energy<br>Or<br>EE**
**CR_6**	-4273.909243	-4273.668128	-4273.667184	-4274.182345	-4277.467861
**A2a**	-1015.877148	-1015.865698	-1015.864754	-1015.916145	-1016.072092
**A2b**	-1352.884161	-1352.869104	-1352.868159	-1352.929597	-1353.084142
**A2a$\subset$$CR_6$**	-5289.822501	-5289.568164	-5289.567220	-5290.111374	-5293.578003
**A2b$\subset$$CR_6$**	-5626.775674	-5626.515751	-5626.514807	-5627.072559	-5630.534705

E(0)= EE + Zero–point Energy
E= EE + Thermal Energy Correction
H= EE + Thermal Enthalpy Correction
G= EE + Thermal Free Energy Correction
EE= Electronic energy

Table S21. Relative enthalpies (ΔH) and Gibbs free energies (ΔG) (in kcal mol^{-1}) for the encapsulation processes of imines **A2a** and **A2b** inside the capsule.

	**ΔH_r**	**ΔG_r**
**A2a$\subset$$CR_6$**	-22.14	-8.08
**A2b$\subset$$CR_6$**	12.87	24.71

Referred to those of the capsule **CR_6** and the corresponding non-encapsulated imines

Cartesian coordinates and frequencies for **A2a⊂CR$_6$** .

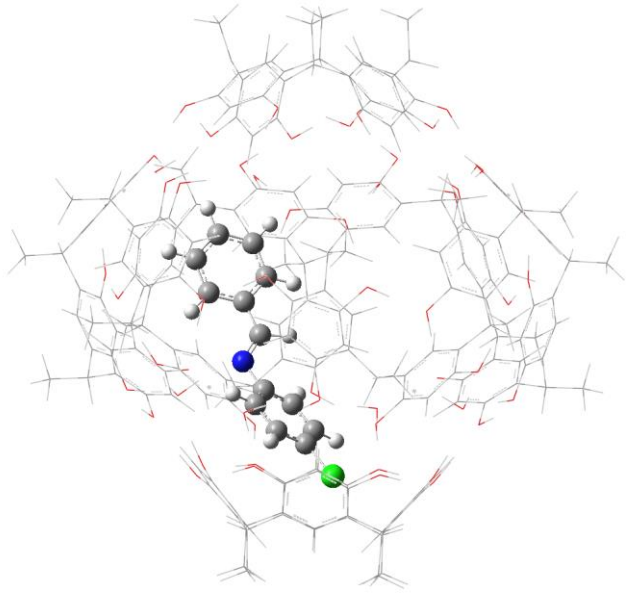

O	6.10753700	6.11387800	1.68171800
O	3.68731200	5.38351900	5.83158300
C	5.96384300	5.53238500	2.93444100
C	4.85806500	5.83539600	3.74524200
C	4.78350900	5.20880600	4.98895400
C	5.78615600	4.33923100	5.46684500
C	6.84394700	4.05038200	4.60694200
C	6.95031700	4.60960800	3.31578000
C	5.69237100	3.70359300	6.83667100
O	6.00705200	-3.59565900	5.44872000
O	8.27099600	-3.03664200	1.19887800
C	6.83675700	-2.89731800	4.57717700
C	7.11728000	-3.40044500	3.30386300
C	7.96146100	-2.63361600	2.49846300
C	8.51707000	-1.40683600	2.90970000
C	8.18134200	-0.94440100	4.19099600
C	7.35335300	-1.67340800	5.05415900
C	9.41525600	-0.62693300	1.96497700
O	2.95604100	2.77134800	6.70984600
O	4.13604100	-1.75664900	6.40158000
C	3.99628100	1.84471100	6.64045900
C	3.59410700	0.51219900	6.54514200
C	4.60658900	-0.45128300	6.47058400
C	5.98123300	-0.12710200	6.48606400
C	6.31629600	1.22999100	6.57832200
C	5.34826800	2.23687900	6.68435500
C	7.02856700	-1.22195700	6.46466400
O	7.39956500	-0.74495200	-0.16967500
O	6.18253000	3.73967700	0.22419100
C	7.63419700	0.44969900	0.51247400
C	6.80496400	1.50299000	0.12018000
C	6.98452000	2.73301000	0.74413700
C	7.98631100	2.96444000	1.74533700
C	8.87353900	1.86086000	1.99943700
C	8.66306500	0.59743800	1.48007600

C	8.09798500	4.11274200	2.54399800
O	-5.50262400	-6.21819100	-2.15347300
O	-7.84965400	-3.30257400	0.91404700
C	-6.40285800	-5.19329100	-1.88098300
C	-6.61708300	-4.76707200	-0.56669300
C	-7.56821300	-3.76179300	-0.37231900
C	-8.29090000	-3.17390600	-1.42960100
C	-8.00473700	-3.62001900	-2.72886100
C	-7.07789600	-4.63753100	-2.98723200
C	-9.34553100	-2.12374500	-1.14539600
O	-6.28661600	3.72868100	-4.31672400
O	-3.51823500	0.77268700	-6.95827100
C	-6.06485200	2.52911400	-4.98648000
C	-4.84837400	2.29623900	-5.63343600
C	-4.71257600	1.08229100	-6.31059600
C	-5.75454600	0.13583000	-6.39225800
C	-6.92665100	0.40004000	-5.67126300
C	-7.11341200	1.58824800	-4.95506300
C	-5.60475200	-1.09526800	-7.25729600
O	-2.85106600	-1.90507000	-6.92665100
O	-3.92310400	-5.73285800	-4.41011800
C	-3.85668400	-2.67930600	-6.35181700
C	-3.42521000	-3.83701000	-5.70266000
C	-4.40688700	-4.60496400	-5.06719000
C	-5.77475900	-4.26490300	-5.07706300
C	-6.14623800	-3.10133400	-5.76426400
C	-5.21253100	-2.30244700	-6.43373200
C	-6.78843000	-5.15057300	-4.38223500
O	-8.19969400	-0.52644400	0.99660600
O	-7.25513800	3.14371400	-1.77842300
C	-8.25898400	0.00120800	-0.29278800
C	-7.76398900	1.30048700	-0.42035800
C	-7.79747000	1.86562400	-1.69878900
C	-8.32874100	1.19510300	-2.82133000
C	-8.80454700	-0.10790100	-2.62730900
C	-8.79640700	-0.73050900	-1.37099500
C	-8.37459000	1.88334700	-4.17055500
O	-7.86132200	1.18611700	3.14330900
O	-5.82937300	-2.63978200	5.16455200
C	-7.25544900	0.56309200	4.22904800
C	-6.83574400	-0.76803600	4.14755900
C	-6.25915300	-1.31912800	5.29570700
C	-6.13218900	-0.61166900	6.50646300
C	-6.52610600	0.73337900	6.51086700
C	-7.09343700	1.34824200	5.39000400
C	-5.62173700	-1.30543400	7.75194200
O	0.74575300	2.12731200	8.23952400
O	-1.00790300	5.85664800	5.82345500
C	-0.31498200	2.95802300	7.89263700
C	-0.12626500	4.01547900	6.99774600
C	-1.23604200	4.82132700	6.72993000
C	-2.49096400	4.62616200	7.33889100
C	-2.62899200	3.52256800	8.19184900
C	-1.55891700	2.67273600	8.49221300
C	-3.62402400	5.59954400	7.08825000
O	-3.76220300	-3.20336400	6.94191500
O	0.00748100	-0.58904800	8.26932300
C	-3.21867300	-2.04804700	7.49869300
C	-1.83533200	-1.89309100	7.60996000
C	-1.37629800	-0.70810700	8.19296700

C	-2.24456400	0.28659000	8.69032700
C	-3.61955900	0.09216500	8.51054300
C	-4.13834600	-1.06803700	7.92379200
C	-1.68908700	1.48867400	9.42491800
O	-3.33686900	6.53449000	4.49016000
O	-6.83299500	3.78046200	2.74675200
C	-4.33914800	5.58249400	4.66130500
C	-5.10702000	5.14530300	3.58031500
C	-6.10103400	4.19959200	3.85260000
C	-6.36742900	3.71875800	5.15134700
C	-5.53463700	4.16202500	6.18610500
C	-4.51493900	5.09715400	5.97391500
C	-7.54117800	2.79335700	5.39385800
O	7.75824700	-0.41010200	-2.86327300
O	6.26599000	-4.94241300	-2.61732700
C	7.35407000	-1.59550500	-3.46311600
C	7.02800700	-2.72329600	-2.70938500
C	6.59107500	-3.84788000	-3.41654500
C	6.49352500	-3.88956100	-4.82250800
C	6.79870300	-2.71860100	-5.52971000
C	7.24441300	-1.56184300	-4.87787500
C	6.09309200	-5.17852100	-5.51221900
O	-0.42114500	-2.94581700	-7.59805000
O	1.12293200	1.57489600	-7.43571900
C	0.58459600	-1.99669900	-7.74462500
C	0.34316200	-0.64426900	-7.49098200
C	1.40629400	0.23896600	-7.70835000
C	2.66067600	-0.17908000	-8.19555100
C	2.85875600	-1.55418000	-8.37622300
C	1.84025700	-2.48765200	-8.16093300
C	3.71462300	0.84372500	-8.55266300
O	3.45391800	3.18650500	-7.09790400
O	7.18682200	2.23160300	-4.24503000
C	4.51866300	2.38247100	-6.70741000
C	5.40146000	2.78125400	-5.70129900
C	6.38814200	1.86810300	-5.32784700
C	6.53043800	0.59102600	-5.93937500
C	5.59642700	0.24470200	-6.94440400
C	4.62297400	1.13733500	-7.37794800
C	7.57062200	-0.28340300	-5.48777500
O	4.32981200	-6.50952000	-3.79831900
O	0.44455400	-5.25622200	-6.28430400
C	3.73453800	-5.86469800	-4.88084700
C	2.34743500	-5.88785800	-5.04559800
C	1.83110900	-5.22742800	-6.16399300
C	2.64910700	-4.58878100	-7.11952800
C	4.02737200	-4.56583000	-6.87877400
C	4.60231200	-5.19645700	-5.76848600
C	2.03780100	-3.97384600	-8.36125200
O	2.48709100	-7.63768300	-2.09254700
O	5.32558100	-6.72619700	1.64479400
C	2.79234800	-7.86896600	-0.75372400
C	3.90153900	-7.17378100	-0.26366500
C	4.22804700	-7.37483700	1.08052800
C	3.48836800	-8.21802200	1.93268100
C	2.39318400	-8.89453500	1.37992500
C	2.03742600	-8.76337400	0.03237300
C	3.88795000	-8.36883600	3.38452300
O	-1.84936400	-5.06684100	6.32611700
O	-4.72934600	-6.44461500	2.77231000

C	-2.16122200	-6.02097000	5.36270600
C	-3.27923200	-5.72460400	4.57625600
C	-3.61802100	-6.65222100	3.58836400
C	-2.87646300	-7.82740700	3.35342100
C	-1.77654000	-8.07513100	4.18486600
C	-1.41232000	-7.20634200	5.22022000
C	-3.26927000	-8.76615800	2.23289900
O	-4.11231600	-6.97080400	0.10859400
O	-0.21468900	-7.85363100	-2.49565300
C	-2.88860400	-7.63035500	-0.00038300
C	-2.18661400	-7.38189500	-1.18109200
C	-0.95857600	-8.03213100	-1.33692400
C	-0.42080300	-8.89303500	-0.35540100
C	-1.16980600	-9.09544800	0.80881500
C	-2.42166900	-8.49730000	1.00679000
C	0.91372300	-9.56418300	-0.59340600
O	4.73899900	-5.71639900	4.20230100
O	0.83807400	-5.07779600	6.89992600
C	3.51569700	-6.20786800	4.65909900
C	2.82326500	-5.36990100	5.53683800
C	1.59259300	-5.83207500	6.01335900
C	1.04407000	-7.07675300	5.62896100
C	1.78545900	-7.86825900	4.74697000
C	3.04129100	-7.47423700	4.26526100
C	-0.29025000	-7.51717700	6.18907000
O	-3.09761900	7.49979000	1.93755400
O	1.58814000	8.11758400	2.61251600
C	-1.90529800	8.05698100	1.48504700
C	-0.78692300	7.78757700	2.27993400
C	0.43294700	8.32605300	1.86035400
C	0.56975100	9.09466800	0.68743900
C	-0.58848500	9.33413600	-0.06342500
C	-1.84524900	8.85421000	0.32383000
C	1.92348600	9.63824100	0.28540100
O	2.99875000	5.26048500	-5.31775900
O	-1.70798500	4.79489900	-5.95368300
C	1.73554900	5.84518800	-5.25283400
C	0.67883600	5.01278500	-5.63131100
C	-0.60656600	5.56065200	-5.58444200
C	-0.85821400	6.88778000	-5.17822600
C	0.24154000	7.66818100	-4.80350600
C	1.55465900	7.18309900	-4.84980100
C	-2.27206800	7.42564900	-5.16792400
O	3.88060800	7.64787300	1.06870400
O	4.69152100	6.34633600	-3.45892700
C	3.56620800	7.79541400	-0.28188700
C	4.29355800	6.98232000	-1.15454900
C	4.00652200	7.10450000	-2.51630700
C	3.02231200	7.98605300	-3.01429600
C	2.33018500	8.77541100	-2.08931500
C	2.59901000	8.72311800	-0.71492900
C	2.74958800	8.04657500	-4.50196300
O	-3.97419300	5.20570200	-4.42481100
O	-4.87607600	7.05127000	-0.11228000
C	-3.74879500	6.22356700	-3.49995800
C	-4.44493300	6.09942300	-2.29618600
C	-4.22359200	7.09139300	-1.33703100
C	-3.33001100	8.16562000	-1.54173700
C	-2.67576300	8.24223000	-2.77598800
C	-2.88962100	7.30076400	-3.79110300

C	-3.11049400	9.18121400	-0.44239700
O	1.34042000	6.03216200	4.44441400
O	-5.73455900	-3.84056700	2.71113500
O	6.23096200	-4.60118100	0.09383200
O	-1.24138200	2.13152300	-6.09661900
O	-1.11786300	-5.92952700	-4.02805900
O	6.12472100	4.30941500	-2.56201500
O	1.76380500	-2.72708400	7.60357700
O	-6.52387700	5.02176300	0.23442700
H	5.33375400	6.71875400	1.46958100
H	2.91469200	5.80838900	5.35941400
H	5.59511300	-4.40169800	5.01262100
H	7.67929700	-3.78034800	0.88999000
H	3.26694400	3.70560200	6.52048900
H	4.84989900	-2.41064700	6.14192700
H	7.84750700	-1.52445200	0.26949400
H	6.28926000	4.60806500	0.70936000
H	-5.02356100	-6.52327900	-1.32454700
H	-7.18442600	-3.63843700	1.58170900
H	-5.47985800	4.32604500	-4.37219000
H	-2.75871800	1.36201900	-6.66035300
H	-3.12758400	-0.93855100	-6.99408100
H	-4.53909900	-6.01003400	-3.66817800
H	-8.18495900	-1.52940000	0.98104900
H	-7.03995200	3.40389600	-2.72300900
H	-7.98547200	0.55475500	2.37111400
H	-5.14372000	-2.87733800	5.86293200
H	1.57535600	2.36392500	7.72655200
H	-1.86540700	6.16998400	5.39683900
H	-3.04892200	-3.88375600	6.73488600
H	0.28730200	0.36877000	8.36579000
H	-3.28785300	6.87220100	3.54270700
H	-7.28167100	2.89866200	2.91002200
H	7.69024700	-0.48587600	-1.86248100
H	5.63028600	-5.57136300	-3.08394300
H	-1.30645500	-2.53291600	-7.35764900
H	1.95362700	2.13629100	-7.37970400
H	3.34826600	3.98447900	-6.49655600
H	7.54855900	1.41365600	-3.79609400
H	1.50219000	-7.78434500	-2.27182700
H	0.11739200	-4.49755700	-6.85375000
H	-0.61583000	-7.12692000	-3.10329100
H	5.72269300	-6.05852300	1.01235800
H	-0.87439100	-5.10778600	6.59259800
H	-5.12723300	-5.53346700	2.89685600
H	-4.40150900	-6.88505400	1.06582000
H	-0.59786500	-5.71561700	-4.83054100
H	5.01314700	-6.15896300	3.34504400
H	1.26871200	-4.17668200	7.14139300
H	-3.77727400	7.39928500	1.19632300
H	1.43238500	7.45930400	3.35193100
H	-1.47432500	3.81481300	-6.05187800
H	3.11034500	7.92210000	1.65083900
H	5.28488200	5.63335500	-3.03898600
H	-3.20170100	5.11771900	-5.06438800
H	-5.51892700	6.25609500	-0.03581200
H	0.52396200	6.00406200	5.00641800
H	1.27893600	5.25025200	3.87006000
H	-5.02833600	-3.33891900	2.27019600
H	-5.84442700	-3.40972100	3.59702900

H	6.31161700	-4.75405400	-0.88224500
H	5.51126900	-3.95517300	0.19307600
H	-1.01900900	1.75875000	-5.20150400
H	-0.45511100	1.93176000	-6.65690000
H	-2.07050500	-5.87564000	-4.24952100
H	6.17801000	4.01008900	-1.62996600
H	6.59488800	3.66693100	-3.12823900
H	1.14139900	-2.01117300	7.84481300
H	2.55704300	-2.31263100	7.20860300
H	-6.62922200	4.62046000	1.12101000
H	-6.80645900	4.35186800	-0.41905700
H	3.64673200	-6.94726400	-3.20124400
C	2.60322800	9.48515300	-5.00908100
H	3.51043600	10.06440400	-4.79508300
H	2.44735800	9.50153500	-6.09485300
H	1.76305800	10.01438000	-4.54913900
C	-2.35639000	8.85841400	-5.70661900
H	-3.39891100	9.19914100	-5.74029000
H	-1.79975300	9.57517700	-5.09532200
H	-1.95546000	8.91458300	-6.72623800
C	-3.12450600	10.62060000	-0.96797900
H	-4.08058100	10.84161800	-1.45975100
H	-3.00109700	11.33696800	-0.14648100
H	-2.33169100	10.81517200	-1.69652300
C	1.84577100	11.08357700	-0.21910900
H	2.84712400	11.46835500	-0.45002600
H	1.24142800	11.18472300	-1.12577300
H	1.40593700	11.73846000	0.54334900
C	-8.31463800	3.15737200	6.66619300
H	-8.66554700	4.19621100	6.62333300
H	-9.19609300	2.51421700	6.78139800
H	-7.71295600	3.05307700	7.57404600
C	-6.42136600	-0.91806700	9.00100500
H	-6.07237500	-1.48411900	9.87402800
H	-6.33683900	0.14473000	9.24715900
H	-7.48718000	-1.14012400	8.86589200
C	-4.41042800	5.91871800	8.36445300
H	-5.18245900	6.67256000	8.16406400
H	-4.91269900	5.04218200	8.78484700
H	-3.74738500	6.32104600	9.14036000
C	-2.49931900	1.82501900	10.68187800
H	-2.53866300	0.96352600	11.36042900
H	-2.03909700	2.65735500	11.22884400
H	-3.53136900	2.11146500	10.45826400
C	8.30298500	-0.84429800	7.22933000
H	8.07107800	-0.56815200	8.26530600
H	8.99849000	-1.69286600	7.26237700
H	8.83781200	-0.00537100	6.77341300
C	10.76728500	-0.31752100	2.61202700
H	11.42154900	0.22494300	1.91891900
H	10.67161700	0.28487000	3.52144200
H	11.28079200	-1.24624200	2.89343300
C	9.42010400	4.68202700	2.91118000
H	10.23856700	4.29627900	2.28946800
H	9.40199000	5.77781500	2.77264200
H	9.68278400	4.50920600	3.96475800
C	6.95831700	3.94304900	7.66450200
H	6.87064700	3.48205000	8.65598400
H	7.85806100	3.53865800	7.19034900
H	7.12611300	5.01782400	7.81158900

C	8.97492600	0.17493900	-5.44222300
H	9.44809200	0.00688900	-4.45746800
H	9.58015200	-0.40360800	-6.16424300
H	9.09920900	1.23675400	-5.70135500
C	2.84214600	-4.29558400	-9.62513300
H	2.92905000	-5.38052600	-9.76454500
H	2.34683500	-3.88567700	-10.51438000
H	3.85715000	-3.88776000	-9.59914200
C	6.90775800	-5.43598700	-6.78409500
H	6.63827200	-6.40381900	-7.22634500
H	6.75020800	-4.67294800	-7.55258700
H	7.98169000	-5.46130600	-6.56142400
C	4.52463300	0.45000800	-9.79398400
H	5.21084500	1.25749600	-10.07983000
H	5.13003600	-0.44806600	-9.63861000
H	3.86291000	0.26023700	-10.64815000
C	-8.06541800	-5.33447500	-5.20923500
H	-7.83227600	-5.75244000	-6.19663700
H	-8.75432900	-6.02761600	-4.71028800
H	-8.60563000	-4.39659800	-5.37063000
C	-6.85526100	-1.35778600	-8.10513100
H	-6.70335800	-2.22057000	-8.76545400
H	-7.74372600	-1.56125000	-7.49982400
H	-7.08306000	-0.49109800	-8.73844900
C	-10.63531400	-2.37773400	-1.93318700
H	-11.40126300	-1.63577600	-1.67473300
H	-10.48737200	-2.33100400	-3.01644600
H	-11.04302400	-3.36974600	-1.70153400
C	-9.64089700	1.54499100	-4.96402900
H	-10.54039200	1.80321200	-4.39149500
H	-9.67151300	2.11233500	-5.90299400
H	-9.70870200	0.48391500	-5.22277600
C	0.92434500	-11.02675600	-0.13481200
H	0.13951700	-11.59824100	-0.64639900
H	1.88555500	-11.50093700	-0.36903900
H	0.75935600	-11.13622500	0.94129700
C	3.86313000	-9.83072800	3.84411500
H	4.19758900	-9.91618800	4.88571100
H	2.86610500	-10.27775900	3.78430200
H	4.53386700	-10.44286400	3.22837800
C	-3.22964300	-10.23617800	2.66310300
H	-3.55714800	-10.88972200	1.84484400
H	-2.22961700	-10.56429500	2.96291000
H	-3.89952400	-10.40922500	3.51480000
C	-0.28533700	-8.99003800	6.61424000
H	0.49509400	-9.17277200	7.36392100
H	-1.24694400	-9.26895500	7.06262500
H	-0.10224000	-9.67321700	5.77954300
H	-4.94652100	5.51211600	2.56979500
H	-5.69328600	3.77517400	7.19478200
H	-4.31152000	0.86509000	8.85114700
H	-1.13913600	-2.65065000	7.25921000
H	-5.11678600	5.26559700	-2.11298100
H	-1.98286900	9.06617800	-2.95700600
H	0.83656800	4.20431200	6.53440100
H	-3.60279100	3.32513300	8.64540500
H	-0.64879000	1.22077800	9.78240100
H	-5.77566700	-2.41822700	7.61210400
H	-3.16745200	6.57739700	6.74683600
H	-8.26631600	2.92793300	4.53465300

H	-9.62133600	-2.20846400	-0.05105900
H	-8.40792800	2.99905700	-3.98266700
H	-4.76511300	-0.89310300	-7.98935800
H	-6.32886200	-6.17974100	-4.27675700
H	-6.07907900	-5.21111800	0.26549900
H	-8.52657200	-3.15640500	-3.56918700
H	-7.72416400	-0.34613900	-5.67070900
H	-4.04588600	3.02906300	-5.61311600
H	-9.19825200	-0.65862000	-3.48428300
H	-7.35089000	1.84063100	0.42604700
H	-2.37723000	-4.11639000	-5.67699200
H	-7.19925300	-2.81260900	-5.78359400
H	-0.50840800	9.92545000	-0.97858400
H	-0.87609200	7.19133900	3.18166900
H	2.56675900	9.66306000	1.21661300
H	-3.97899100	9.10022400	0.27893700
H	3.65089000	7.61492500	-5.03293200
H	-2.88029800	6.78572900	-5.87662500
H	0.06979200	8.69465000	-4.47142900
H	0.85772700	3.99012400	-5.94780900
H	1.56029000	9.46005800	-2.45077600
H	5.04374600	6.28701300	-0.78659600
H	-6.95760700	-1.34885400	3.23949200
H	-6.39633500	1.31799100	7.42437300
H	9.63216300	-1.27447600	1.06519600
H	6.70708100	-4.34686500	2.96576300
H	8.57258200	0.02124800	4.52038000
H	7.37137900	1.51414800	6.58329700
H	6.58656500	-2.10903600	7.01307100
H	4.84513600	4.20055800	7.39704800
H	2.54486100	0.23401000	6.50421300
H	6.02809100	1.35986800	-0.62760900
H	9.68558400	2.02655500	2.70807000
H	4.09683200	6.53391600	3.41070800
H	7.61074400	3.34217200	4.93361500
H	5.67051700	-0.74039000	-7.40679700
H	5.31723600	3.74665200	-5.20664800
H	-0.62336600	-0.28754500	-7.14689100
H	3.83572100	-1.90837200	-8.71237400
H	-2.57338900	-6.70288200	-1.93656800
H	-0.77000200	-9.75024100	1.58595700
H	1.80237500	-9.55259100	2.02094700
H	4.47513800	-6.51537000	-0.90658200
H	1.37850600	-8.83146100	4.43223400
H	3.22415000	-4.40346400	5.82943800
H	-4.34543300	-8.54502600	1.96216000
H	4.96249800	-8.02628100	3.47983700
H	1.08732600	-9.58299100	-1.71193800
H	6.70675100	-2.70890400	-6.61693200
H	7.10402700	-2.72985900	-1.62667500
H	6.32895000	-6.03255100	-4.80800600
H	1.01592800	-4.43777100	-8.51004800
H	3.17522400	1.80266700	-8.82143000
H	4.67816600	-4.04510900	-7.58450000
H	1.69049200	-6.39139900	-4.34066900
H	-3.84918000	-4.81612400	4.73906100
H	-1.18853700	-8.98142100	4.02372800
H	-0.47647900	-6.91798700	7.13180700
H	3.65399100	5.73058200	-4.70961100
C	1.89692300	1.90083200	-3.54171700

C	3.20827700	2.33831700	-3.39194000
C	4.16749100	1.49619300	-2.81748900
C	3.81873200	0.21572300	-2.39055000
C	2.49847500	-0.21058600	-2.51001600
C	1.53272600	0.62584700	-3.08543800
H	1.14097800	2.55501000	-3.96806200
H	3.48255900	3.34018600	-3.70489700
H	4.56872100	-0.43465200	-1.94974700
H	2.20723300	-1.19564500	-2.15171600
C	0.14328100	0.16089500	-3.08747200
N	-0.82626900	0.74476600	-3.68702600
H	-0.05315000	-0.72467200	-2.46921500
C	-4.63812200	-0.78797200	-2.86551300
C	-4.41935000	0.58764800	-2.83296300
C	-3.15626100	1.07762200	-3.15505500
C	-2.11895600	0.19584600	-3.47240200
C	-2.36524700	-1.17894600	-3.53670300
C	-3.62633200	-1.67460300	-3.22143300
Cl	-6.20451500	-1.41898600	-2.44374300
H	-5.21813200	1.26626300	-2.54796100
H	-2.96093300	2.14480900	-3.12348300
H	-1.58180100	-1.86391900	-3.84344200
H	-3.82111800	-2.74279000	-3.24808800
H	5.18595300	1.84798700	-2.70543700

0 imaginary frequency

Cartesian coordinates and frequencies for **A2b⊂CR$_6$**.

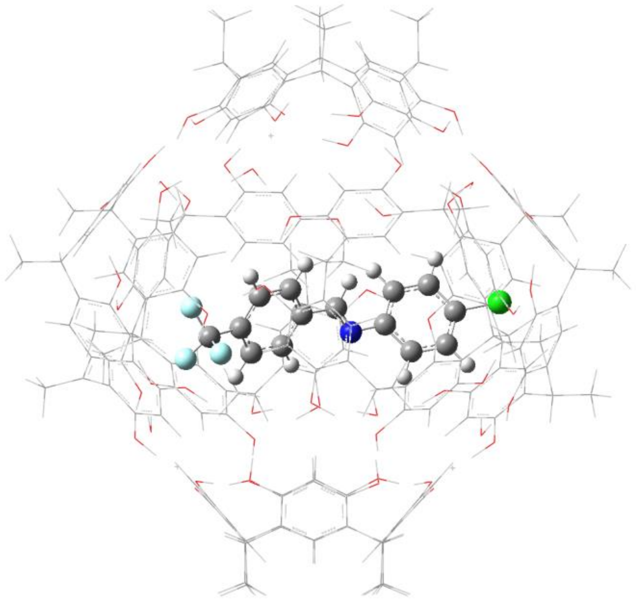

O	-7.22976400	-4.85647700	-0.94208700
O	-6.11134200	-4.88981000	3.78547900
C	-7.38621300	-4.40445400	0.36066800
C	-6.64707200	-4.96725600	1.41377200
C	-6.85136800	-4.45065500	2.69237800
C	-7.80184500	-3.43832600	2.96262000
C	-8.49210500	-2.90489500	1.88031300
C	-8.28756300	-3.34798800	0.55238400
C	-8.01565000	-2.91765200	4.36784300
O	-6.54607800	4.32818600	3.69433200

O	-7.60147900	4.49012800	-1.03347500
C	-7.21255100	3.86274200	2.56528000
C	-7.02302200	4.47918500	1.32514400
C	-7.73796500	3.95485200	0.24699800
C	-8.60492500	2.85038100	0.36268200
C	-8.73870000	2.26344500	1.63011400
C	-8.06793500	2.75649700	2.75621900
C	-9.33183500	2.32416700	-0.86238800
O	-5.27370800	-2.55755700	5.22338700
O	-5.43607400	2.12178300	4.98785000
C	-6.04696600	-1.43717600	4.92292700
C	-5.40241200	-0.21170800	5.09694300
C	-6.14046300	0.93915900	4.79898500
C	-7.48289900	0.90296200	4.36137100
C	-8.06839000	-0.35996700	4.20298900
C	-7.38476700	-1.54713200	4.49522700
C	-8.25696000	2.18803100	4.14893600
O	-6.84965400	2.16897200	-2.41032800
O	-6.55057800	-2.47952100	-2.17647100
C	-7.44550900	1.00691800	-1.94246800
C	-6.74201800	-0.16402100	-2.22573700
C	-7.27623800	-1.37144800	-1.77947500
C	-8.52878100	-1.45800700	-1.08571300
C	-9.25287600	-0.21775200	-0.94988000
C	-8.70339200	1.00468800	-1.26770300
C	-9.05743200	-2.59087100	-0.44463100
O	6.83935100	5.22583500	0.19452000
O	7.73320400	1.73174300	3.41146100
C	7.45950600	4.03710100	0.56793100
C	7.22769500	3.49674300	1.83648200
C	7.90641100	2.31950000	2.15724500
C	8.78384900	1.67433100	1.26571200
C	8.95135700	2.24894500	-0.00300500
C	8.31654700	3.43957900	-0.37803600
C	9.50020100	0.40727800	1.68076900
O	6.73147200	-4.48675600	-3.02886800
O	5.70625600	-0.97624300	-6.20218800
C	6.96999100	-3.24482400	-3.60982400
C	6.14670700	-2.76215600	-4.63014800
C	6.47545200	-1.51600100	-5.17187100
C	7.57664700	-0.75526100	-4.73316200
C	8.33427500	-1.27102400	-3.67238100
C	8.06638700	-2.52091000	-3.10021000
C	7.91198700	0.56473400	-5.39541800
O	5.29935400	1.76809000	-5.82375100
O	5.79516100	4.92633900	-2.40650700
C	6.12877800	2.26439400	-4.81979900
C	5.60373900	3.34454800	-4.11211000
C	6.40105700	3.88435100	-3.09776000
C	7.69308100	3.40577300	-2.79829700
C	8.15676400	2.30737200	-3.53575700
C	7.40568300	1.72391300	-4.56451400
C	8.52580600	4.08592600	-1.73209800
O	7.41851700	-1.02845100	3.11895100
O	6.83311100	-4.24641800	-0.25335200
C	7.80897700	-1.47168300	1.85569500
C	7.15722100	-2.62381500	1.41118200
C	7.52259700	-3.10807100	0.15146300
C	8.50974500	-2.49587300	-0.64917400
C	9.10905800	-1.33045000	-0.15481200

C	8.80224100	-0.80852300	1.10871600
C	8.90138500	-3.10372400	-1.97996700
O	6.31263300	-2.77099100	4.93355100
O	4.40647000	1.23182300	6.72257100
C	5.51884300	-2.11455200	5.86705300
C	5.35321500	-0.72715400	5.81867200
C	4.56594800	-0.15073400	6.81958400
C	3.98760200	-0.89472400	7.86588000
C	4.14592800	-2.28721300	7.82992600
C	4.90637200	-2.92608200	6.84574400
C	3.26049300	-0.19603500	8.99532800
O	-3.45036900	-2.36885900	7.30225800
O	-1.68105500	-6.14942100	4.98545800
C	-2.47962500	-3.35566600	7.15502000
C	-2.56361400	-4.28156100	6.11125600
C	-1.56813100	-5.26095600	6.05373800
C	-0.53790400	-5.36296900	7.00931200
C	-0.47885100	-4.37545200	8.00155300
C	-1.43633000	-3.35977600	8.10266900
C	0.43812500	-6.52025700	6.96600700
O	2.05407800	2.07759400	7.95962200
O	-2.31449300	0.14058700	7.86736400
C	1.18769100	1.01340300	8.19795800
C	-0.17443400	1.11893200	7.90863800
C	-0.96788200	0.00212500	8.18991500
C	-0.45631700	-1.17352400	8.77711200
C	0.92458500	-1.23416500	9.00240300
C	1.77242000	-0.15487700	8.72816200
C	-1.39053400	-2.29636200	9.17732600
O	0.79100000	-7.19860400	4.29892400
O	5.06907500	-5.09900700	3.96650800
C	1.84206600	-6.48083200	4.86437000
C	2.96378500	-6.13852600	4.10677900
C	3.97626000	-5.42954700	4.76058700
C	3.91390900	-5.09204900	6.12906000
C	2.74567500	-5.42749100	6.82428000
C	1.69409200	-6.12733800	6.22120200
C	5.09453300	-4.42680700	6.80481300
O	-6.50573800	2.15700200	-5.17280400
O	-4.61175100	6.42742300	-4.13718700
C	-5.74341800	3.27079800	-5.51566800
C	-5.60091000	4.35026800	-4.64492700
C	-4.75377800	5.38341300	-5.06004400
C	-4.08454100	5.38533800	-6.29830600
C	-4.22151100	4.25410200	-7.11268600
C	-5.06313200	3.18367800	-6.76410400
C	-3.27821700	6.59466300	-6.72888500
O	3.30569300	3.43887600	-6.83171800
O	1.28305500	-0.77299700	-7.83406900
C	2.29519200	2.68572200	-7.41763100
C	2.29684200	1.29002200	-7.33035800
C	1.25112900	0.61511700	-7.96708700
C	0.24956900	1.27410300	-8.70610400
C	0.27656600	2.67515300	-8.71988000
C	1.28813600	3.40801100	-8.09135800
C	-0.79035900	0.48447900	-9.47228900
O	-1.24058900	-1.91378100	-8.15637600
O	-5.32845300	-0.43171500	-6.15046700
C	-2.23118100	-0.94788200	-7.97419300
C	-3.33968900	-1.21791700	-7.16326800

C	-4.27852300	-0.19797800	-7.03505100
C	-4.16658100	1.04900800	-7.71444600
C	-3.00751900	1.27323000	-8.47104800
C	-2.02921600	0.28289700	-8.62767300
C	-5.25071100	1.98483900	-7.53317000
O	-2.06346500	7.56895800	-4.40966000
O	2.20426500	5.72913300	-5.44151800
C	-1.23038900	6.84501900	-5.26890000
C	0.10387100	6.62771900	-4.92493700
C	0.89682600	5.93509000	-5.84823700
C	0.39937900	5.51494700	-7.10304800
C	-0.96103200	5.70878100	-7.36127400
C	-1.80723400	6.37331000	-6.46138300
C	1.34101700	4.91841600	-8.12698300
O	-0.43283400	8.35417300	-2.20826600
O	-4.27493900	7.42440400	0.50277000
C	-1.06244100	8.41911600	-0.95479700
C	-2.35489400	7.89695000	-0.89988300
C	-2.99223900	7.93777200	0.34449800
C	-2.39200100	8.50228800	1.49242100
C	-1.07285700	8.95164600	1.38247000
C	-0.37892300	8.91983800	0.16343500
C	-3.17918200	8.62586200	2.77999100
O	0.80872000	4.40889300	7.21505200
O	4.88783800	5.46839200	4.95455100
C	1.54899100	5.33061500	6.48029900
C	2.83259600	4.91089800	6.11731100
C	3.59148500	5.79706600	5.34906800
C	3.10449400	7.04763100	4.91744100
C	1.82347000	7.42467500	5.34094300
C	1.03275700	6.60013000	6.14975700
C	3.93434400	7.91665300	3.99368000
O	4.95818900	5.84835400	2.19911100
O	2.02275900	6.88587000	-1.28096600
C	3.92478900	6.68246700	1.77997800
C	3.43881600	6.40310900	0.50565800
C	2.45688500	7.25402100	-0.01161300
C	1.99397200	8.38743900	0.68986700
C	2.46931900	8.57407300	1.99772000
C	3.43177400	7.74058100	2.57484600
C	1.05802200	9.37862500	0.03445300
O	-4.51997700	6.06600200	2.97727800
O	-1.88636600	4.74430600	6.73971700
C	-3.47811800	6.35787900	3.85740200
C	-3.22270500	5.38148300	4.82287800
C	-2.19007600	5.64282000	5.72908400
C	-1.40820200	6.82024100	5.67714500
C	-1.71220000	7.75669000	4.68582400
C	-2.76483400	7.56906900	3.77837500
C	-0.31049100	7.03888500	6.69483300
O	1.23311800	-7.85858000	1.68737900
O	-3.47233000	-7.53010200	0.95368900
C	0.14691500	-8.14334100	0.86610800
C	-1.08520100	-7.67662200	1.33528700
C	-2.20544100	-7.94553800	0.54473700
C	-2.13023000	-8.63929800	-0.67929200
C	-0.86741100	-9.08218600	-1.09433300
C	0.28720800	-8.87463100	-0.33075000
C	-3.38191900	-8.88604300	-1.49250200
O	-1.86407000	-4.18199700	-6.71969300

O	2.82942200	-4.72685000	-6.06051500
C	-0.81674700	-5.02576300	-6.36556900
C	0.44832700	-4.42970300	-6.38442200
C	1.53412100	-5.24086700	-6.04786900
C	1.39328900	-6.59702400	-5.68878600
C	0.10033700	-7.13525300	-5.68654500
C	-1.02417300	-6.38248800	-6.04582100
C	2.61130100	-7.41812100	-5.32774600
O	-5.08544300	-6.57452800	-1.11260700
O	-4.22754600	-4.94878700	-5.52621500
C	-4.41571600	-6.72944700	-2.32791200
C	-4.68328800	-5.75234700	-3.28848100
C	-4.01877000	-5.87293300	-4.51322500
C	-3.10518300	-6.91572400	-4.78666000
C	-2.88518200	-7.86542800	-3.78437900
C	-3.54853000	-7.81536400	-2.55029500
C	-2.41352400	-6.97941400	-6.13123100
O	4.40203500	-5.61339100	-3.94185200
O	3.57975300	-7.70468900	0.28016100
C	3.72033700	-6.58361400	-3.20850600
C	4.02554900	-6.63173400	-1.84663500
C	3.33903900	-7.57856300	-1.08094400
C	2.36530300	-8.44382700	-1.62753700
C	2.11651900	-8.35622500	-3.00111400
C	2.80270700	-7.45566700	-3.82618600
C	1.64047900	-9.42497600	-0.73256200
O	-3.54372000	-5.68062700	3.02506100
O	5.37544600	2.73357700	4.64752500
O	-5.12517400	5.74266800	-1.53529100
O	3.05736000	-1.95643400	-6.11795000
O	3.13342000	4.14242300	-1.56404600
O	-5.78028900	-2.91100900	-4.90880900
O	-3.33150700	2.56311200	6.83273700
O	5.35783400	-6.08169800	1.32530200
H	-6.49436200	-5.54195100	-1.01083600
H	-5.27886800	-5.37074500	3.50132400
H	-5.85950800	5.02204400	3.45976600
H	-6.83540200	5.12859000	-1.09944600
H	-5.63709300	-3.38886500	4.79609100
H	-5.90127400	2.91333000	4.58522200
H	-7.25603900	2.99381100	-2.01299100
H	-6.89701900	-3.33472100	-1.78407800
H	6.18840400	5.51918500	0.89670400
H	7.00770600	2.18420200	3.93014900
H	5.90268700	-4.92013800	-3.39737900
H	4.82878500	-1.44824900	-6.29148300
H	5.51989000	0.81608800	-6.04758400
H	6.25759300	5.11967100	-1.54085200
H	7.62882300	-0.05732100	3.25648400
H	6.91300500	-4.40283300	-1.24094900
H	6.72528800	-2.13102800	4.27638000
H	3.60165500	1.54956900	7.23582600
H	-4.14515400	-2.43597500	6.58163600
H	-0.80138400	-6.59703300	4.78088700
H	1.56058900	2.91173800	7.68762000
H	-2.76133400	-0.75135800	7.76499500
H	0.98220200	-7.44888400	3.34171200
H	5.58407500	-4.32863500	4.34997500
H	-6.70078700	2.15635400	-4.19399900
H	-3.76251700	6.93257300	-4.30186400

H	4.02240300	2.85103500	-6.44747300
H	0.38782700	-1.19025800	-8.03061600
H	-1.47500400	-2.76144800	-7.67143200
H	-5.78575200	0.42495900	-5.89755800
H	0.17834500	9.12188700	-2.33592200
H	2.64062700	5.00141600	-5.96882900
H	1.14639800	7.27382000	-1.52425400
H	-4.58034200	6.91472100	-0.30849900
H	-0.18361700	4.56696900	7.11282000
H	5.08252700	4.49250300	5.06826000
H	5.05638400	5.84977500	3.19687100
H	2.53975300	4.90551900	-1.67362300
H	-4.47788600	6.62558200	2.14754000
H	-2.49284400	3.91407000	6.73575900
H	2.10864000	-7.86577100	1.18028800
H	-3.43074400	-6.93460400	1.76005000
H	2.84110900	-3.73009300	-6.18052400
H	-4.57388100	-7.00685500	-0.36404100
H	-4.86086400	-4.19096400	-5.24640300
H	3.89481900	-5.36083900	-4.77149900
H	4.29674300	-7.05434100	0.62395300
H	-2.90582000	-5.85825300	3.76221200
H	-3.28878800	-4.81823000	2.65249300
H	4.75725700	2.55120500	3.91956700
H	5.08533700	2.15177600	5.39425600
H	-5.01850300	6.03373000	-2.47953100
H	-4.59382000	4.93146600	-1.46195200
H	2.80397400	-1.63042600	-5.23704000
H	2.44905200	-1.50428600	-6.75710200
H	3.95234900	4.40914500	-2.02065100
H	-6.02476800	-2.67889200	-3.99202100
H	-5.62359700	-2.06968900	-5.38728100
H	-2.97617200	1.73225900	7.21056100
H	-4.06423500	2.33277600	6.22733800
H	5.24752800	-5.76194000	2.24375800
H	5.88660800	-5.41597500	0.84159100
H	-1.55003100	7.89140300	-3.62146300
C	-2.40586200	-8.39778600	-6.71218600
H	-3.43024800	-8.77540900	-6.82279300
H	-1.93976000	-8.40860700	-7.70534100
H	-1.86096400	-9.11066400	-6.08612000
C	2.57273200	-8.82298000	-5.93960600
H	3.49140200	-9.37437600	-5.70304300
H	1.73145000	-9.42112600	-5.57664900
H	2.48966400	-8.76841800	-7.03221800
C	1.54324400	-10.82029700	-1.35914700
H	2.54405000	-11.21503900	-1.57679800
H	1.05198700	-11.52216500	-0.67422600
H	0.98032900	-10.82673800	-2.29738600
C	-3.42488200	-10.29707900	-2.08941500
H	-4.36654300	-10.46202100	-2.62796500
H	-2.61003200	-10.48398000	-2.79569000
H	-3.35551900	-11.05572600	-1.30004800
C	5.37904800	-5.01585800	8.19093000
H	5.55479100	-6.09702900	8.12420800
H	6.27615900	-4.56066200	8.62886900
H	4.55638900	-4.86172900	8.89553400
C	3.58695000	-0.80674800	10.36291100
H	3.10060100	-0.23888900	11.16612500
H	3.25791200	-1.84637600	10.45368000

H	4.66782400	-0.78728800	10.55014500
C	0.73967100	-7.07936700	8.36098100
H	1.39651000	-7.95594200	8.29135000
H	1.23511700	-6.35298200	9.01222700
H	-0.18326700	-7.39544500	8.86251300
C	-1.04538200	-2.87399800	10.55442800
H	-1.06369600	-2.08894800	11.32084400
H	-1.77374200	-3.64060100	10.84711300
H	-0.05417800	-3.33655400	10.58497800
C	-9.74600900	2.04588300	4.48790900
H	-9.88077300	1.68670200	5.51578900
H	-10.25424400	3.01562300	4.40913800
H	-10.26820000	1.35146900	3.82287300
C	-10.84253000	2.25236000	-0.63056900
H	-11.36360200	1.89443000	-1.52704900
H	-11.11378500	1.59478600	0.20140100
H	-11.24213800	3.24824200	-0.39510700
C	-10.50475900	-2.90843700	-0.51229400
H	-11.06988000	-2.25525900	-1.19100700
H	-10.62906500	-3.94119800	-0.89453700
H	-11.00099200	-2.89517600	0.46962700
C	-9.49381800	-2.93789900	4.76743900
H	-9.62486100	-2.57287000	5.79405600
H	-10.11951200	-2.31952200	4.11694100
H	-9.89223800	-3.95988100	4.72697100
C	-6.61427700	1.60650500	-7.97653300
H	-7.39178400	1.86203800	-7.23827300
H	-6.86695600	2.15972400	-8.89777000
H	-6.71001400	0.53775900	-8.21219500
C	1.09045400	5.47181400	-9.53499100
H	1.17579500	6.56512900	-9.54414600
H	1.82758100	5.07389500	-10.24345700
H	0.09824500	5.21738500	-9.91992200
C	-3.56091000	6.98178300	-8.18476400
H	-3.00588100	7.88571800	-8.46232600
H	-3.28179700	6.19439800	-8.89255900
H	-4.62890200	7.18491000	-8.33242100
C	-1.11708900	1.10916400	-10.83356300
H	-1.81721600	0.47331500	-11.39029400
H	-1.57943400	2.09733300	-10.74567400
H	-0.21093500	1.21984600	-11.44081600
C	10.01132700	4.15757600	-2.10410900
H	10.14643100	4.66721300	-3.06628200
H	10.57394400	4.71953500	-1.34849300
H	10.47455100	3.16984300	-2.18856500
C	9.40607900	0.67926300	-5.71821000
H	9.62014400	1.62241100	-6.23606300
H	10.03626600	0.64535600	-4.82436300
H	9.72494500	-0.14013500	-6.37439600
C	10.99159500	0.45015000	1.33020500
H	11.49863300	-0.46051900	1.67230700
H	11.17175000	0.54175400	0.25463200
H	11.47920900	1.30460900	1.81606800
C	10.40285200	-2.98532200	-2.26410300
H	10.98961600	-3.44588400	-1.45986500
H	10.66042600	-3.49917600	-3.19897600
H	10.73525900	-1.94735900	-2.36192800
C	1.23357100	10.81260100	0.54517100
H	2.27235900	11.14737300	0.42431200
H	0.59290100	11.51041600	-0.00714500

H	0.97955100	10.91626700	1.60573200
C	-3.09629900	10.04418800	3.35566400
H	-3.69932300	10.12767400	4.26925800
H	-2.07469500	10.34058100	3.61208100
H	-3.48058500	10.77995000	2.63825400
C	3.97378900	9.37759500	4.44878800
H	4.60384800	9.97815500	3.78100900
H	2.98319100	9.84171000	4.47589000
H	4.39598200	9.45524000	5.45940100
C	-0.27385900	8.48030500	7.21541400
H	-1.23835900	8.75622000	7.66016600
H	0.49251700	8.59268300	7.99287200
H	-0.05277400	9.21078800	6.43174700
H	3.05416400	-6.40182300	3.05575100
H	2.65635000	-5.14482600	7.87505200
H	1.35287400	-2.14878700	9.41770700
H	-0.60807500	2.01996300	7.48381500
H	4.75562700	-5.95844600	-1.40625400
H	1.36962300	-9.01640000	-3.44634100
H	-3.36505100	-4.24385400	5.38048400
H	0.33648800	-4.40571200	8.72783200
H	-2.43113100	-1.86016800	9.26966200
H	3.63117000	0.87294400	9.03159900
H	-0.05293200	-7.36135800	6.38919900
H	6.01294900	-4.63870200	6.17747000
H	9.44244000	0.33435600	2.80837900
H	8.67714400	-4.21203500	-1.92576200
H	7.37047900	0.59497600	-6.38868000
H	8.16633500	5.15666800	-1.65622100
H	6.55696300	3.98091400	2.54024900
H	9.60744500	1.75280700	-0.72220600
H	9.16506600	-0.67901200	-3.28222900
H	5.30048800	-3.33602300	-4.99552600
H	9.85151400	-0.81788900	-0.77033000
H	6.38733100	-3.10840000	2.00327100
H	4.61464800	3.74532500	-4.32386400
H	9.14134200	1.89612700	-3.30500200
H	-0.78381100	-9.61756700	-2.04297900
H	-1.15391400	-7.13291900	2.27146700
H	-4.26357600	-8.81446800	-0.78677900
H	2.25425500	-9.54524800	0.21103600
H	-3.01328800	-6.34615400	-6.85319200
H	3.51303200	-6.90264300	-5.77751900
H	-0.03288700	-8.18207400	-5.40479600
H	0.56622100	-3.38387000	-6.65046100
H	-2.17935000	-8.67735900	-3.97114400
H	-5.37352700	-4.93752500	-3.09169800
H	5.81552400	-0.12542300	5.04317300
H	3.66868600	-2.89247100	8.60377400
H	-9.17347900	3.05418000	-1.71075900
H	-6.35946100	5.33088200	1.21244200
H	-9.38394400	1.38877700	1.73904000
H	-9.10156800	-0.42172300	3.85356500
H	-7.84139700	2.94478900	4.88260100
H	-7.48194900	-3.61245700	5.08314200
H	-4.36889600	-0.15257900	5.42437000
H	-5.79398700	-0.13308700	-2.76337200
H	-10.23985900	-0.26250600	-0.48497400
H	-5.93944300	-5.77198200	1.23028300
H	-9.20060300	-2.09055900	2.05694900

H	-2.88114300	2.23578800	-8.96640800
H	-3.45409700	-2.16453500	-6.64351300
H	3.07460000	0.75612500	-6.79459400
H	-0.51293300	3.21265000	-9.24995800
H	3.78457700	5.52961000	-0.05923600
H	2.08262300	9.41141400	2.58313000
H	-0.56449400	9.33580600	2.26988800
H	-2.83690100	7.48819700	-1.78420100
H	-1.12180800	8.67343800	4.62718300
H	-3.79965500	4.46122700	4.86257100
H	5.00138800	7.54336600	4.02908500
H	-4.27001700	8.45016700	2.53167700
H	1.32518700	9.40364400	-1.06570700
H	-3.67974100	4.20396700	-8.05747600
H	-6.11725900	4.39335400	-3.69370300
H	-3.60970000	7.47270600	-6.09721100
H	2.39292100	5.22987200	-7.84675500
H	-0.35168900	-0.53542900	-9.69106000
H	-1.37880000	5.34334300	-8.30190700
H	0.52727300	6.95724000	-3.98112800
H	3.21293600	3.94408500	6.43173600
H	1.42987900	8.39403400	5.02815200
H	-0.54712800	6.38660200	7.59019300
H	-2.74808100	-4.50020900	-6.34961000
C	-1.38209900	-1.39354400	-0.66883100
C	-2.64758100	-1.80541100	-0.27438000
C	-3.19500500	-1.29933100	0.90804000
C	-2.51091800	-0.36338500	1.67934900
C	-1.24701600	0.05293000	1.26974300
C	-0.66817900	-0.47324200	0.11063300
H	-0.92079400	-1.78422900	-1.57038800
H	-3.21189000	-2.51190500	-0.87446500
H	-2.95663800	0.03064100	2.58736200
H	-0.69688900	0.77463900	1.86782300
C	0.71979800	-0.10368300	-0.22659900
N	1.36697100	-0.70156800	-1.14749700
H	1.18751700	0.66323300	0.40599900
C	5.44115400	0.17837000	-1.47580600
C	5.00269400	-1.14328100	-1.51925000
C	3.63759900	-1.40077100	-1.46313700
C	2.72511800	-0.34807900	-1.32331000
C	3.17999300	0.97683000	-1.33010800
C	4.54553600	1.24138100	-1.40665700
Cl	7.16044900	0.50172100	-1.48892300
H	5.71607700	-1.96048700	-1.57481600
H	3.26994300	-2.42134400	-1.47476500
H	2.49961800	1.82316300	-1.29788700
H	4.89274600	2.26999500	-1.39953800
C	-4.50563800	-1.83325500	1.39895500
F	-5.21033700	-0.92636600	2.09170700
F	-5.28312900	-2.28008700	0.40563700
F	-4.31500400	-2.89038300	2.23935800

0 imaginary frequency

14. Non-covalent interactions

The reduced density gradient (RDG) analysis-based NCI results for **A2a** ⊂ **CR$_6$** complex is illustrated in Figure S78. The strong interaction associated with the hydrogen bond between the water and imine nitrogen atom appear evident in the NCI 2D-plot (Figure S78, blue color) so as the Van der Waals interactions appear in green color.

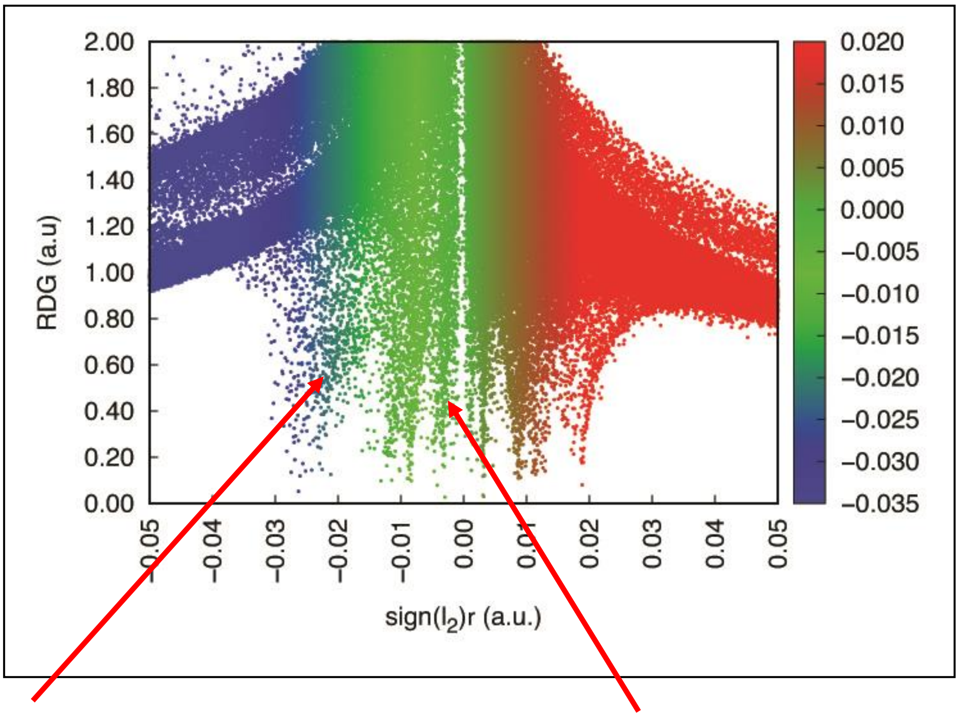

H-Bond interactions

van deer Waals interactions

Figure S78. Plots of the RDG versus the electron density multiplied by the sign of the second Hessian eigenvalue (s = 0.5 a.u.; left) and gradient isosurfaces (s = 0.4 a.u.; right) for the **A2a** ⊂ **CR$_6$**. The coloring scheme was chosen to assist in distinguishing the amplitude of the electron density corresponding to different types of interactions. Blue and green colors represent strong (hydrogen-bond) and medium-strong (Van der Waals) interactions, whereas the red color represents the repulsive ones.

15. References

(1) Osypenko, A.; Dhers, S.; Lehn, J.-M. *J. Am. Chem. Soc*. **2019**, *141*, 12724−12737

(2) Zhang, Q.; Tiefenbacher, K. *J. Am. Chem. Soc.* **2013**, *135*, 16213-16219.

(3) (a) Tunstad, L. M.; Tucker, J. A.; Dalcanale, E.; Weiser, J.; Bryant, J. A.; Sherman, J. C.; Helgeson, R. C.; Knobler, C. B.; Cram, D. J. *J. Org. Chem.* **1989**, *54*, 1305-1312; (b) Elidrisi, I.; Negin, S.; Bhatt, P. V.; Govender, T.; Kruger, H. G.; Gokel, G. W.; Maguire, G. E. M. *Org. Biomol. Chem.* **2011**, *9*, 4498- 4506.

(4) Yeap, G.-Y.; Al-Taifi, E. A.; Ong, C.-H.; Mahmood, W. A. K.; Takeuchi, D.; Ito, M. M. *Phase Transitions* **2012**, *85*, 483-496.

(5) Liu, L.; Zhang, S.; Fu, X.; Yan, C.-H. *Chem. Commun.* **2011**, *47*, 10148-10150.

(6) Iqbal, A.; Siddiqui, H. L.; Ashraf, C. M. *Chem. Pharm. Bull.* **2007**, *55*, 1070-1072.

(7) Miyamura, H.; Morita, M.; Inaasaki, T.; Kobayashi, S. *Bull. Chem. Soc. Jpn.* **2011**, *84*, 588-599.

(8) Hasegawa, A.; Naganawa, Y.; Fushimi, M.; Ishihara, K.; Yamamoto, H. *Org. Lett.* **2006**, *8*, 3175-3178.

(9) Torregrosa, R.; Pastor, I. M.; Yus, M. *Tetrahedron* **2005**, *61*, 11148-11155.

(10) Lai, J.-T.; Yang, Y.-J.; Lin, J.-H. ; Yang, D.-Y. *Synlett.* **2010**, *1*, 111-114.

(11) Bennett, J. S.; Charles, K. L.; Miner, M. R.; Heuberger, C. F.; Spina, E. J.; Bartels, M. F.; Foreman, T. *Green Chem.* **2009**, *11*, 166-168.

(12) Bruer, T.M.; Zhang, Q.; Tiefenbacher, K. *Angew. Chem. Int. Ed.* **2016**, *55*, 7698-7701.

(13) L. Avram, Y. Cohen, *Org. Lett*. **2006**, *8*, 219-222

(14) La Manna, P.; Talotta, C.; Floresta, G.; De Rosa, M.; Soriente, A.; Rescifina, A.; Gaeta, C.; Neri, P. *Angew. Chem. Int. Ed.* **2018**, *57*, 5423-5428.

(15) The ^{1}H NMR signals of the encapsulated aldehyde **2a** are very complex, and in accord with the data previously reported by Cohen[13] and by us,[14] which indicates that the molecules of **2a** are encapsulated in slightly different nanocontainers.

(16) Zhou, P.; Jiang, L.; Wang, S.; Hu, X.; Wang, H.; Yuan, Z.; Zhang, Z. *ACS Catal.* **2019**, *9*, 8413-8423.

(17) L. Avram, Y. Cohen *Org. Lett* **2003**, *5*, 3329-3332.
